# Synthesis of Small Libraries of Natural Products—Part III: Identification of New Esters from *Pelargonium graveolens* L’Her. (Geraniaceae) Essential Oil

**DOI:** 10.3390/molecules30244741

**Published:** 2025-12-11

**Authors:** Šejla F. Gusinac Avdović, Marko Z. Mladenović, Niko S. Radulović

**Affiliations:** 1 Department of Chemistry, Faculty of Sciences and Mathematics, University of Niš, Višegradska 33, 18000 Niš, Serbia; sejla.gusinac@gmail.com (Š.F.G.A.); markohem87@gmail.com (M.Z.M.); 2 Department of Sciences and Mathematics, State University of Novi Pazar, Vuka Karadžića 9, 36300 Novi Pazar, Serbia

**Keywords:** *Pelargonium graveolens*, essential oil, esters, synthetic library, NMR, GC-MS, structure elucidation

## Abstract

*Pelargonium graveolens* (rose geranium) essential oil contains numerous aroma-active esters that are challenging to identify at low abundance. We obtained by preparative chromatography an ester-rich fraction of the essential oil and constructed a synthetic reference library of 159 structurally related esters (spectral/GC data provided; 102 newly synthesized). This enabled dereplication and detection of constituents not apparent in direct GC–MS of the unfractionated oil. Nine esters (5-methylhexyl formate, (*Z*)-hex-3-en-1-yl 3-methylpentanoate, 3-methylbutyl 3-methylpentanoate, 3-methylpentyl 4-methylpentanoate, 5-methylhexyl hexanoate, 3-methylbutyl 6-methylheptanoate, 2-phenylethyl 6-methylheptanoate, 5-methylhexyl tiglate, and 6-methylheptyl tiglate) were confirmed as new natural products (eight of them new compounds overall), by combined evidence from retention indices, EI mass spectra, co-injections with synthesized references, and, in selected cases, by 1D/2D NMR. Systematic RI trends across acid and alcohol isomers were delineated, aiding rapid differentiation of regio-isomeric esters that share similar EI patterns. This library-guided workflow offers a robust path to differentiate structurally close volatiles in complex matrices and provides transferable RI/spectral benchmarks for future natural product identification.

## 1. Introduction

The genus *Pelargonium* (Geraniaceae), encompassing approximately 230 perennial species and numerous hybrids, has long intrigued botanists and horticulturists due to its diverse morphology and wide-ranging adaptability [1]. These plants, widely grown for ornamental purposes, have become a common feature in gardens, balconies, and windowsills worldwide [1]. *Pelargonium* species have demonstrated considerable economic significance, with applications spanning the production of aromatic essential oils, traditional medicine, and culinary uses [1].

*Pelargonium graveolens* L’Her. (Geraniaceae), commonly known as ‘rose geranium’, is an aromatic plant distributed across Mozambique, Zimbabwe, and several provinces of South Africa [1,2]. In South Africa, the extract of *P. graveolens* is incorporated in *ca*. fifty health-related patents and is utilized in the treatment of a wide range of conditions [2]. Numerous recent studies have substantiated the broad pharmacological potential of *P. graveolens*, which includes hypoglycemic, anti-tumor, anti-inflammatory, hepatoprotective, antioxidant, and antibacterial activities [3]. Furthermore, its aromatic flavor is strongly associated with its therapeutic applications, such as relaxant, sedative, anxiolytic, antidepressant, and tension-alleviating effects, underscoring its multifaceted medicinal significance.

*Pelargonium graveolens* is most widely recognized as a source of the essential oil commonly known as rose geranium oil, which has been extensively chemically studied. Essential oil analysis has revealed variations in its chemical composition, with citronellol, geraniol, 10-*epi*-γ-eudesmol, citronellyl formate, linalool, and menthol identified as predominant constituents [1,4]. The essential oil of *P. graveolens* is distinguished by a complex and characteristic odor profile comprising sweet, floral, and mildly citrus-like notes reminiscent of rose. For that reason, *P. graveolens* essential oil is particularly notable for its role as an economical alternative to rose oil, which is one of the most expensive essential oils used in perfumery. The ability of *P. graveolens* essential oil to mimic the olfactory characteristics of rose oil has positioned it as a cost-effective substitute, particularly in products that aim to deliver premium fragrance profiles without incurring the high costs associated with authentic rose oil. This unique olfactory signature is, in general, attributed to the balanced combination of citronellol, geraniol, linalool, and their derivatives [2].

Despite extensive studies of the major constituents, numerous low-abundance branched and isomeric esters in *P. graveolens* essential oil remain poorly characterized, largely due to overlapping EI mass spectra and small ΔRI values between regio-isomers. Although many of these esters occur only at trace levels, their sensory impact can be disproportionately high due to low odor thresholds, making them key contributors to the characteristic aroma of *P. graveolens*. However, their low abundance often results in “weak” or “noisy” mass spectra and poor GC signal-to-noise ratios, which further complicate their detection and identification. Reliable identification of such esters requires comprehensive reference data sets rather than single-compound comparisons. Building on our ‘small synthetic libraries of natural products’ approach (latest Part II [5]), here we construct a targeted library of 159 esters and apply it to an ester-rich fraction of commercial *P. graveolens* essential oil to (i) resolve closely related isomeric esters by GC–MS/RI, and (ii) identify previously undescribed natural esters. Specifically, our goal was to examine previously undetected trace components that were not identified in the unfractionated *P. graveolens* essential oil sample. Based on mass fragmentation patterns observed in the MS spectra and the obtained RI data, these components were identified as esters of various isomeric alcohols and acids, including (iso)heptyl formate, (iso)pentyl, (iso)hexyl, (*Z*)-hex-3-en-1-yl, and (iso)heptyl esters of one of the isomeric hexanoic acids, (iso)pentyl (iso)octanoate, 2-phenylethyl (iso)heptanoate and (iso)octanoate, as well as (iso)heptyl and (iso)octyl tiglate, angelate, or senecioate.

## 2. Results and Discussion

### 2.1. Composition of the Ester-Rich Fraction of P. graveolens Essential Oil

**Figure 1:**
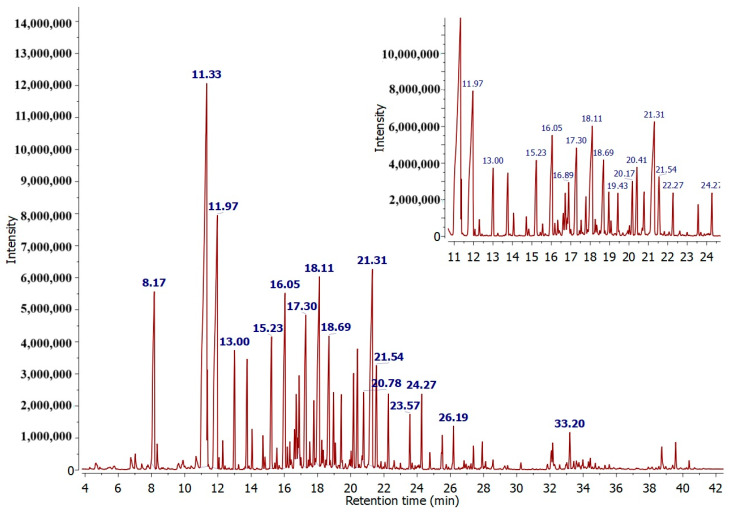
Typical TIC (total ion current) chromatogram of *P. graveolens* essential oil fraction (fraction VI), with a chromatogram expansion of the ester-rich region of the chromatogram (*ca*. 11–24 min).

The chromatographic fractionation of a commercially available *P. graveolens* essential oil was performed on SiO_2_, using *n*-hexane/diethyl ether (Et_2_O) mixtures as the mobile phase. Employing a shallow gradient with low Et_2_O content facilitated the isolation of several fractions predominantly containing citronellyl, neryl, and geranyl esters. Our focus was directed towards the initial ester-rich fraction (fraction VI), which, upon analysis, revealed the presence of citronellyl formate (19.7%), geranyl formate (10.1%), geranyl butanoate (6.8%), and geranyl tiglate (7.2%) as the major constituents, and numerous minor esters that were not detectable in direct GC-MS analyses of the unfractionated oil (Figure 1 and Table S1). In addition to esters of monoterpene alcohols, a significant number of esters of linear and branched aliphatic alcohols and fatty acids, such as heptyl propanoate, 2-methylpropyl tiglate, and 2-phenylethyl isobutyrate, were also detected and initially identified by comparison of their RI values and mass spectra with literature data, and subsequently confirmed by GC co-injection with authentic standards.

**Figure 2:**
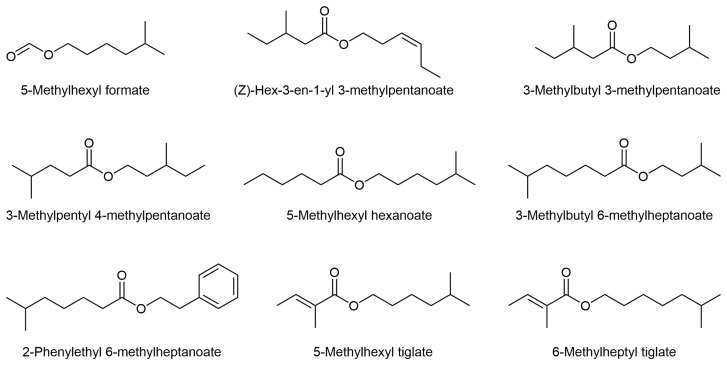
Chemical structures of newly identified esters from *P. graveolens* essential oil: 5-methylhexyl formate (**15a**), (*Z*)-hex-3-en-1-yl 3-methylpentanoate (**10f**), 3-methylbutyl 3-methylpentanoate (**3f**), 3-methylpentyl 4-methylpentanoate (**7g**), 5-methylhexyl hexanoate (**15h**), 3-methylbutyl 6-methylheptanoate (**3m**), 2-phenylethyl 6-methylheptanoate (**19m**), 5-methylhexyl tiglate (**15p**), and 6-methylheptyl tiglate (**17p**).

For one of the ester series comprising twelve constituents, the analysis of mass spectra and retention indices suggested that they represent formate esters of various alcohols. The final identification of these compounds was achieved through the comparison of their retention index values and mass spectral fragmentation patterns with literature data and further confirmed by GC co-injection with authentic standards. The identified esters included 3-methylpentyl, (*Z*)-hex-3-en-1-yl, hexyl, heptyl, benzyl, 2-phenylethyl, linalyl, *neo*-menthyl, *neoiso*-menthyl, citronellyl, and neryl formate. However, an additional detected compound displayed an identical fragmentation pattern in its mass spectrum to that of heptyl formate but had a slightly lower retention index compared to the n-chain homolog. This implied the existence of a branched-chain in the alcohol moiety. The specific branching patterns, i.e., most likely esters of 2-, 3-, 4-, and 5-methylhexanols, were inferred from the characteristic decreases in retention index (RI) values associated with a single methyl branch when compared to their straight-chain counterparts, thereby excluding other isomers, such as those with multiple branching [5]. Unfortunately, for direct identification through retention index (RI) and mass spectrometry (MS) comparison, the literature lacks data on formates of isomeric heptanols. GC-MS co-injection of the synthesized series of isomeric heptyl formates (**11a**–**16a**, see Experimental part) with the essential oil fraction confirmed the presence of 5-methylhexyl formate (*syn*. isoheptyl formate) in *P. graveolens* essential oil. To the best of our knowledge, this ester represents a new natural product (Figure 2). Compounds were classified as (i) new compounds if not previously reported at all, and (ii) new natural products if not previously described from natural sources, based on SciFinder searches (26 October 2025) and primary literature inspection (Table S2).

The additional four detected, yet unidentified, constituents were found to possess quite similar fragmentation patterns in the mass spectra compared to the esters of isomeric hexanoic acids (the ions formed by cleavage next to the carbonyl group appeared in mass spectra of detected esters as the signals at *m*/*z* 71 and 99 corresponding to [C_5_H_11_]^+^ and [C_6_H_11_O]^+^, respectively [5]). Analysis of the additional ions in the mass spectra and calculated retention index (RI) values suggests that three of the mentioned esters are likely formed from isomeric hexanoic acids and various isomers of pentanol, hexanol, and heptanol. Fragmentation patterns in MS of the remaining GC peak at RI = 1341 were consistent with hexanoates (*m*/*z* 99) or some other long-chain acid esters of hexenol (base peaks at *m*/*z* 82 (C_6_H_10_^+^, McLafferty rearrangement at the alkyloxy side)). Based on biosynthetic considerations, we hypothesized that the unidentified ester is most likely derived from (*Z*)-hex-3-en-1-ol, commonly known as leaf alcohol, whose dominance among hexenols reflects a combination of biosynthetic predisposition, enzymatic selectivity, and biological function. This hypothesis was supported by the presence of (*Z*)-hex-3-en-1-ol in the essential oil and the identification of seven additional esters of this alcohol within the analyzed fraction. The identification of these esters was confirmed through a comparison of their MS and RI data with ones from the literature. Additionally, esters of regio-isomers and/or diastereomers of (*Z*)-hex-3-en-1-ol were not detected in the essential oil fraction, further supporting our hypothesis.

**Scheme 1:**
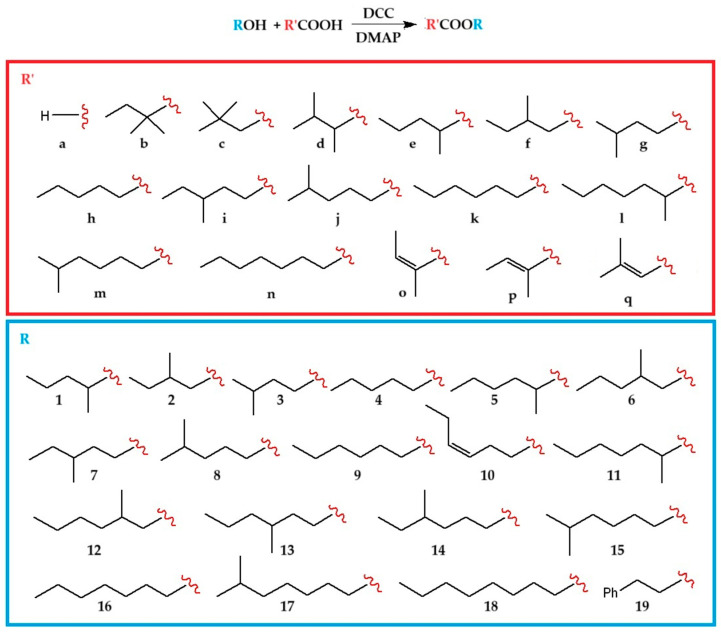
Synthesis of esters.

An approach to identify all above-mentioned constituents involved creating a small synthetic library of MS and RI data for possible esters formed from isomeric pentanols, hexanols, heptanols, and (*Z*)-hex-3-en-1-ol with various isomeric hexanoic acids, followed by multiple co-injection of the synthesized esters with the essential-oil fraction to achieve accurate identification. Esters of isomeric pentanols (**1**–**4**), hexanols (**5**–**9**), heptanols (**11**–**16**), and (*Z*)-hex-3-en-1-ol (**10**) with all constitutional isomers of hexanoic acid (2,2-dimethylbutanoic (**b**), 3,3-dimethylbutanoic (**c**), 2,3-dimethylbutanoic (**d**), 2-methylpentanoic (**e**), 3-methylpentanoic (**f**), 4-methylpentanoic (**g**), and hexanoic acid (**h**)) were synthesized using the standard Steglich procedure (Scheme 1). On 26 October 2025, a SciFinder search of the Chemical Abstracts Service (CAS) database revealed that 102 of the 159 synthesized esters were previously unreported in the literature. Through multiple co-injection procedures of esters from the synthetic library with essential oil fraction samples, we confirmed that 3-methylbutyl 3-methylpentanoate, 3-methylpentyl 4-methylpentanoate, 5-methylhexyl hexanoate, and (*Z*)-hex-3-en-1-yl 3-methylpentanoate are constituents of the essential oil fraction (evidence summarized in Table S2). The facile and reliable identification of these isomeric esters underscores the efficacy of the ‘small synthetic libraries’ approach for the unambiguous identification of natural products with multiple possible regio-isomers.

The identification of (*Z*)-hex-3-en-1-yl 3-methylpentanoate and 5-methylhexyl hexanoate is particularly significant, as they represent previously unreported natural products, thereby contributing novel insights into the chemical profile of *P. graveolens* essential oil. At the time of this investigation, 3-methylbutyl 3-methylpentanoate represented a new natural product, as it appears only in a 1974 patent as a synthetic derivative, without MS and RI data, added to tobacco as a flavoring agent. The novelty status of the remaining identified component of the fraction, 3-methylpentyl 4-methylpentanoate, needs deliberation. A detailed search of the SciFinder database revealed that this ester (registered under CAS Registry Number: 1215128-04-3) is a constituent of the volatile fraction of *Capsicum* fruits from the *annuum*–*chinense*–*frutescens* complex [6]. However, a thorough examination of the cited study indicated that this ester is not among the 107 identified esters, which include 2-methylpropyl, 3-methylbutyl, pentyl, 4-methylpentyl, hexyl, 5-methylhexyl, 4-methylhexyl, heptyl, and methyloctyl 4-methylpentanoate. This means that 3-methylpentyl 4-methylpentanoate also represents a completely new natural product.

Two additional esters were straightforwardly identified through the analysis of fragmentation patterns observed in mass spectra, followed by the synthesis of corresponding reference standards and their co-injection with essential oil fraction samples. One of the detected esters exhibited the same fragmentation pattern in the mass spectrum as another constituent of the essential oil fraction, namely 3-methylbutyl octanoate (base ion *m*/*z* 70 that is indicative for (iso)pentyl moiety, along with characteristic fragment ions suggesting the presence of octanoic acid (*m*/*z* 127)), but with a slightly lower retention index (RI = 1410 compared to 1448 for 3-methylbutyl octanoate). The difference in retention index values and the presence of an additional series of 3-methylbutyl esters suggested that the compound is 3-methylbutyl 6-methylheptanoate rather than a 2-methylbutyl or pentyl ester, or a 3-methylbutyl ester of another isomeric octanoic acid. The final confirmation of this tentative identification was achieved through the synthesis of reference standards for isomeric pentyl octanoates and 6-methylheptanoates, followed by their co-injection with the essential oil fraction. Using the same analytical approach, it was confirmed that the second detected ester (RI = 1812) is 2-phenylethyl 6-methylheptanoate. Both identified esters represent new natural products and new compounds in general.

For the final two mentioned compounds, mass fragmentation analysis suggested that they were possibly esters of angelic, tiglic, or senecioic acid with aliphatic alcohols. The presence of additional tiglic acid esters in the fraction, with similar fragmentation pattern in their mass spectra, further supported our initial hypothesis. Mass fragmentation patterns and the obtained retention index values indicated that these esters were derived from certain isomeric heptanols and octanols. Heptyl tiglate, which was present in the essential oil fraction and finally identified by GC co-injection of the essential-oil fraction sample with synthesized heptyl esters of angelic, tiglic, and senecioic acids, exhibited an RI value 38 indices higher than the detected isomeric ester. This suggested the presence of an *iso*-branch in the alcohol moiety. Final confirmation of the proposed structure was achieved through the synthesis of a series of tiglates, senecioates, and angelates with all isomeric heptanols, which confirmed that the essential oil constituent at RI = 1393 is a new natural product—5-methylhexyl (*syn*. isoheptyl) tiglate. As the majority of isomeric octanols are not commercially available, the comprehensive synthesis of esters for all isomeric octyl tiglates, angelates, and senecioates presents a significant challenge in terms of both feasibility and complexity. The retention index (RI = 1496) and an observed RI increment of approximately 100 RI units, when compared to previously characterized fraction’s constituents such as isohexyl tiglate (RI = 1295) and isoheptyl tiglate (RI = 1393), strongly suggested that the detected compound is another tiglate featuring an *iso*-branch in its alcohol moiety. Final structural confirmation was achieved through the synthesis and GC co-injection of reference standards for 6-methylheptyl and octyl tiglates, angelates, and senecioates, ultimately verifying the presence of a previously unreported natural product in the fraction (6-methylheptyl tiglate).

### 2.2. Chromatographic (RI) and NMR Data

A survey of the natural occurrence of the esters presented in Figure 2, along with structurally related compounds (Scheme 1), revealed that these esters (only 57 esters from 159 chemical entities from the created library) have been reported only sporadically as plant metabolites or scarcely mentioned in the literature. One possible explanation for this limited identification is the absence of comprehensive chromatographic data (e.g., GC retention indices) and spectral characterization (e.g., mass spectra). Additionally, their reported distribution in nature should be interpreted with caution, as some studies have claimed the identification of these compounds without providing conclusive analytical evidence. One of the potential reasons for the uncertain GC-MS identification of such regio-isomeric compounds is their highly similar mass spectral fragmentation patterns, coupled with the fact that some exhibit closely related RI values.

From a broader phytochemical perspective, it is reasonable to assume that some of the newly identified esters may occur more widely in nature than in the analyzed *P. graveolens* EO sample. Plants sharing similar metabolic profiles, ecological niches, or taxonomic proximity to *Pelargonium* species may biosynthesize analogous ester sets, particularly when derived from common pools of branched-chain precursors [7]. Moreover, plants known to produce significant amounts of branched-chain alcohols and acids, such as species within the Apiaceae, Asteraceae, or Lamiaceae families, represent plausible natural sources where these compounds may also be present but remain analytically overlooked due to their trace abundance and the lack of suitable reference data [8]. Branched aliphatic acids commonly arise from the metabolism of branched-chain amino acids (valine, leucine, and isoleucine) via α-keto acid pathways or through fatty acid biosynthesis involving methylmalonyl–CoA extension [9]. These metabolic routes naturally generate sets of regio-isomeric acids that can subsequently esterify available alcohols through the action of alcohol acyltransferases (AATs), producing structurally diverse ester mixtures [10]. The same biosynthetic principles govern the branched alcohol moieties detected in this study [11]. Their co-occurrence with branched acids is therefore expected, as both derive from shared precursors and parallel metabolic transformations. This metabolic linkage provides additional support for the natural origin of the newly identified esters.

Herein, we report mass spectra and retention indices for 159 synthesized compounds. A comparative analysis of the obtained RI data (Table 1) reveals a consistent elution trend: esters derived from the same alcohol elute in the following order based on their corresponding acids: 2,2-dimethylbutanoates, 3,3-dimethylbutanoates, 2,3-dimethylbutanoates, 2-methylpentanoates, 3-methylpentanoates, 4-methylpentanoates, and hexanoates. This elution pattern aligns with the observations previously reported by Radulović and co-workers [5]. Notably, certain isomeric esters exhibit near GC co-elution; for instance, the retention index difference (ΔRI) between 2,2-dimethylbutanoates and 3,3-dimethylbutanoates, with the same moiety in the alcoholic part, ranges from 2 to 10 units, while that between 3-methylpentanoates and 4-methylpentanoates varies from 2 to 7 units. Esters of the same acid but with different alcoholic moieties also elute in a specific order, e.g., in the case of the isomeric heptanols, i.e., the isomer that elutes first is the one with a methyl branch at position C-1, then methyl branch at positions C-2, C-3, C-5, C-4 while the isomer with the highest RI value is one with a linear alcohol moiety (Table 1, column f, rows 11–16). The analysis of RI data presented in Table 2 reveals a consistent elution order for isomeric esters derived from the same alcohol: angelates elute first, followed by senecioates, and finally tiglates. Notably, tiglates and senecioates exhibit near co-elution, with a ΔRI ranging from 6 to 9 units (Table 2). These findings may contribute to the preliminary prediction of the chromatographic behavior of structurally related esters.

**Figure 3:**
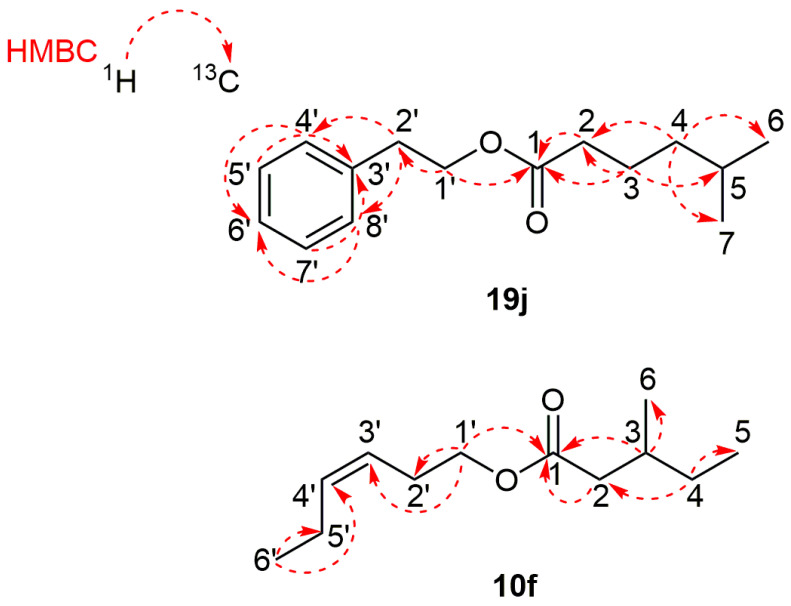
The structure of 2-phenylethyl 5-methylhexanoate (**19j**) and (*Z*)-hex-3-en-1-yl 3-methylpentanoate (**10f**) with the carbon atom numbering scheme and crucial HMBC correlations (marked with arrows) used for analysis.

Detailed NMR analyses (1D: ^1^H and ^13^C, including ^1^H spectra with homonuclear decoupling and ^13^C spectra without heteronuclear decoupling, as well as DEPT-90 and DEPT-135; 2D: HSQC and HMBC) are presented there for the two representative esters from the library, 2-phenylethyl 5-methylhexanoate (**19j**) and (*Z*)-hex-3-en-1-yl 3-methylpentanoate (**10f**) (Figure 3, Tables S3 and S4). The ^1^H and ^13^C NMR spectra of both compounds displayed the expected number of signals, consistent with their proposed ester structures. Comprehensive 2D NMR experiments enabled unambiguous assignment of all carbon and proton signals, confirming the connectivity between the corresponding alcohol and acid fragments. Observed correlations were fully consistent with structural features deduced from MS and RI data. Altogether, these results provide conclusive structural evidence supporting the identification of both compounds as previously undescribed natural esters.

## 3. Materials and Methods

### 3.1. General Experimental Procedures

All solvents and most of the used chemicals (dimethyl malonate, dimethyl 2-methylmalonate, cyclohexane-1,3-dione, methyl iodide, 4-(dimethylamino)pyridine (DMAP), *N*,*N*′-dicyclohexylcarbodiimide (DCC), alcohols ((*Z*)-hex-3-en-1-ol, 2-methylbutan-1-ol, 3-methylbutan-1-ol, pentan-1-ol, 2-methylpentan-1-ol, 3-methylpentan-1-ol, 4-methylpentan-1-ol, hexan-1-ol, octan-1-ol, pentan-2-ol, hexan-2-ol, heptan-2-ol, 2-phenylethan-1-ol), and corresponding acids (formic, 2,2-dimethylbutanoic, 3,3-dimethylbutanoic, 2-methylpentanoic, 3-methylpentanoic, 4-methylpentanoic, hexanoic, 2-methylhexanoic, 3-methylhexanoic, 5-methylhexanoic, heptanoic, 2-methylheptanoic, octanoic, angelic, tiglic, and senecioic acids) were obtained from commercial sources, including Sigma-Aldrich (St. Louis, MO, USA), Merck (Darmstadt, Germany), and Fisher Scientific (Waltham, MA, USA), and were used as received, except for the solvents (*n*-hexane, diethyl ether (Et_2_O), tetrahydrofuran (THF), dichloromethane (DCM), methanol, ethanol, and acetone; HPLC grade), which were distilled and dried prior to use. Silica gel 60 with a particle size distribution of 40–63 µm (Acros Organics, Geel, Belgium) was used for dry-flash column chromatography, while precoated aluminum silica gel plates (Kieselgel 60 F_254_, 0.2 mm; Merck, Darmstadt, Germany) were used for analytical TLC analyses. The TLC plates were visualized by spraying with 50% (*v*/*v*) aqueous H_2_SO_4_, followed by brief heating. ATR-IR measurements (attenuated total reflectance) were conducted using a Thermo Nicolet 6700 FTIR instrument (Waltham, MA, USA). UV spectra (in acetonitrile) were recorded using a UV-1800 PC Shimadzu spectrophotometer (Tokyo, Japan).

### 3.2. Gas Chromatography–Mass Spectrometry (GC-MS) Analyses

GC-MS analyses (performed in triplicate) of the obtained samples were conducted using a Hewlett–Packard 6890N gas chromatograph (Agilent Technologies, Palo Alto, CA, USA) equipped with a fused silica capillary column (DB-5MS, 5% diphenyl-(95% dimethyl)polysiloxane, 30 m × 0.25 mm, film thickness 0.25 µm) coupled with a 5975B mass selective detector from the same manufacturer. The injector and interface temperatures were set to 250 °C and 300 °C, respectively. The oven temperature was programmed to increase from 70 °C to 290 °C at a rate of 5 °C/min, followed by an isothermal hold for 10 min. Helium was used as the carrier gas at a flow rate of 1.0 mL/min. Samples of 1.0 µL, consisting of Et_2_O solutions of the essential oil, the ester fraction of the essential oil, pure esters, and co-injection mixtures were injected in split mode (with a flow rate of 1.5 mL/min for the first 0.5 min, reduced to 1.0 mL/min for the remainder of the analysis; split ratio 40:1). MS conditions were as follows: ionization voltage of 70 eV, acquisition mass range of 35–650 amu, and a scan time of 0.32 s. The linear retention indices were determined relative to the retention times of a series of *n*-alkanes on the column.

### 3.3. NMR Measurements

The ^1^H (including ^1^H NMR selective homonuclear decoupling experiments), ^13^C (with and without heteronuclear decoupling) nuclear magnetic resonance (NMR) spectra, distortionless enhancement by polarization transfer (DEPT-90 and DEPT-135), and 2D (NOESY, and gradient ^1^H–^1^H COSY, HSQC, and HMBC) NMR spectra were recorded on a Bruker Avance III 400 MHz NMR spectrometer (Fällanden, Switzerland; ^1^H at 400 MHz, ^13^C at 101 MHz) equipped with a 5–11 mm dual ^13^C/^1^H probe head. All NMR spectra were measured at 25 °C in CDCl_3_ with tetramethylsilane (TMS) as an internal standard. Chemical shifts are in δ (ppm). ^1^H referenced to TMS (δ_H_ 0.00) or residual CHCl_3_ (δ_H_ 7.26). ^13^C and heteronuclear 2D referenced to ^13^CDCl_3_ (δ_C_ 77.16). The samples were dissolved in 1 mL of the solvent, and 0.7 mL of the solutions were transferred into a 5 mm Wilmad, 528-TR-7 NMR tube. The acquired NMR experiments, both 1D and 2D, were recorded using standard Bruker built-in pulse sequences.

### 3.4. Synthesis of 2,3-Dimethylbutanoic Acid

A solution of dimethyl 2-methylmalonate (5 g; 34.2 mmol), isopropyl iodide (11.5 g; 67.6 mmol), and anhydrous K_2_CO_3_ (18.8 g; 136.2 mmol) in dry acetone (75 mL) was vigorously stirred and refluxed for 8 h, then left overnight at ambient temperature [5]. The solvent evaporated, and the resulting slurry was diluted with H_2_O (150 mL) and extracted with Et_2_O (4 × 50 mL). The organic layers were dried over anhydrous MgSO_4_ and concentrated under reduced pressure to yield a crude mixture (7.2 g). Subsequently, the crude dialkylated dimethyl malonate, an aqueous solution of NaOH (130 mmol in 60 mL), and EtOH (40 mL) were stirred and refluxed for 4 h. The EtOH was evaporated, the aqueous layer was washed with Et_2_O (2 × 50 mL), acidified with 1 M HCl, and extracted with Et_2_O (5 × 70 mL). The combined ether extracts were concentrated under reduced pressure to obtain crude 2-isopropyl-2-methylmalonic acid, which was then decarboxylated by heating at 210 °C for 2 h under nitrogen. The resulting dark oil was subjected to dry-flash chromatography on SiO_2_ using hexane/EtOAc mixtures of gradually increasing polarity, yielding 700 mg of pure 2,3-dimethylbutanoic acid, whose mass spectral data and retention index values were consistent with those reported in the literature [5].

### 3.5. Synthesis of 4-Methylhexanoic Acid

The workflow, using dimethyl malonate (10 g, 75.7 mmol), 2-methylbutyl iodide (18 g, 90.9 mmol), and anhydrous K_2_CO_3_ (50 g, 362.3 mmol) in dry acetone (100 mL), is the same as that used for the synthesis of 2,3-dimethylbutanoic acid. The organic layers were dried over anhydrous MgSO_4_ and concentrated under reduced pressure to give a crude dimethyl 2-(2-methylbutyl)malonate (18.2 g) that was further fractionated by distillation under reduced pressure (in total 4 fractions; 1 (500 mbar, 80 °C, m = 6.15 g), 2 (350 mbar, 110 °C, m = 6.97 g), 3 (300 mbar, 120 °C, m = 1.66 g), and fraction 4 (residue in the flask; m = 3.32 g)). GC-MS analyses of the obtained fractions confirmed that fraction 4 represented pure dimethyl 2-(2-methylbutyl)malonate. Afterward, a mixture of dimethyl 2-(2-methylbutyl)malonate (16.4 mmol), an aqueous solution of NaOH (65 mmol in 60 mL), and EtOH (20 mL) was stirred and refluxed for 4 h. EtOH was evaporated, the water layer was washed with Et_2_O (2 × 50 mL), then acidified with 1 M HCl and extracted with Et_2_O (5 × 50 mL). Combined ether extracts were concentrated under reduced pressure to yield crude 2-(2-methylbutyl)malonic acid that was subsequently decarboxylated by heating at 210 °C for 2 h under nitrogen. The obtained dark oil was purified by dry-flash chromatography on SiO_2_ using hexane/EtOAc mixtures of increasing polarity as the eluent, affording pure 4-methylhexanoic acid (1.6 g), whose mass spectral data and retention index values are in agreement with those reported in the literature [12].

### 3.6. Synthesis of 6-Methylheptanoic Acid

In a 100 mL two-necked flask, 2 g (17.85 mmol) of 1,3-cyclohexanedione and 9.85 g (4 eq.) of anhydrous K_2_CO_3_ were dissolved in 60 mL of HPLC-grade acetone. The mixture was brought to reflux and heated for 10 min. Subsequently, under an inert atmosphere, 5.6 g (38.2 mmol) of MeI was added dropwise through a septum. The reaction mixture was refluxed for an additional 3 h and stirred overnight. Water was then added, and the product was extracted twice with diethyl ether (2 × 100 mL). The combined organic layers were dried over anhydrous MgSO_4_, and the solvent was evaporated under reduced pressure. GC-MS analysis revealed that the product mixture contained C-dialkylated and O-monoalkylated derivatives. The obtained mixture (2 g) was separated by dry-flash chromatography on SiO_2_ using a hexane: Et_2_O (1:1, *v*/*v*) eluent, yielding fraction 1, containing pure 2,2-dimethylcyclohexane-1,3-dione (1.3 g; 9.27 mmol). The isolated 2,2-dimethylcyclohexane-1,3-dione was further reacted by dissolving it in 8.6 mL of ethylene glycol containing 1.17 g (29.3 mmol) of NaOH. Methanol (1.3 mL) and hydrazine hydrate (0.82 mL; prepared by mixing 0.676 mL hydrazine hydrate and 0.144 mL water) were added [13]. The reaction mixture was heated in an oil bath at 120 °C for 6 h and then stirred overnight at ambient temperature. Water and methanol were evaporated under reduced pressure, and the residue was refluxed in an oil bath at 190 °C for 4 h, followed by overnight stirring at room temperature. The reaction mixture was subsequently acidified with 1 M HCl, and the product was extracted three times with diethyl ether (3 × 50 mL). The combined organic layers were dried over anhydrous MgSO_4_, and the solvent was evaporated under reduced pressure. The crude reaction mixture was purified by dry-flash chromatography on silica gel, yielding 0.65 g of pure 6-methylheptanoic acid, whose MS and ^1^H NMR spectral data were consistent with those reported in the literature [14].

### 3.7. Synthesis of Esters

A solution of the appropriate alcohol (pentan-2-ol (**1**), 2-methylbutan-1-ol (**2**), 3-methylbutan-1-ol (**3**), pentan-1-ol (**4**), hexan-2-ol (**5**), 2-methylpentan-1-ol (**6**), 3-methylpentan-1-ol (**7**), 4-methylpentan-1-ol (**8**), hexan-1-ol (**9**), (*Z*)-hex-3-en-1-ol (**10**), heptan-2-ol (**11**), 2-methylhexan-1-ol (**12**), 3-methylhexan-1-ol (**13**), 4-methylhexan-1-ol (**14**), 5-methylhexan-1-ol (**15**), heptan-1-ol (**16**), 6-methylheptan-1-ol (**17**), octan-1-ol (**18**), 2-phenylethan-1-ol (**19**)), carboxylic acid (1.1 eq; formic (**a**), 2,2-dimethylbutanoic (**b**), 3,3-dimethylbutanoic (**c**), 2,3-dimethylbutanoic (**d**), 2-methylpentanoic (**e**), 3-methylpentanoic (**f**), 4-methylpentanoic (**g**), hexanoic (**h**), 4-methylhexanoic (**i**), 5-methylhexanoic (**j**), heptanoic (**k**), 2-methylheptanoic (**l**), 6-methylheptanoic (**m**), octanoic (**n**), angelic (**o**), tiglic (**p**), and senecioic (**q**) acid), DMAP (0.3 eq) and DCC (1.1 eq) in 1.5 mL of dry DCM was left overnight at room temperature, and after filtration of the precipitated *N*,*N*′-dicyclohexylurea, the remaining solution was immediately analyzed by GC–MS [5]. In the case of the esters detected in *P. graveolens* essential oil fraction and several other esters from the library, the mentioned synthetic procedures were repeated on a somewhat larger scale to provide appropriate amounts of the compounds for full spectral characterization. A solution of the appropriate alcohol, carboxylic acid (1.1 eq), DMAP (0.3 eq) and DCC (1.1 eq) in 20 mL of dry DCM was stirred overnight, at room temperature, in a round bottom flask equipped with a CaCl_2_ guard tube. The resulting residue was purified by gradient dry-flash column chromatography; mixtures of hexane and diethyl ether of increasing polarity were used for elution. Esters were washed from the column with 5% (*v*/*v*) diethyl ether in hexane [5]. The purity of the ester fractions was checked by GC-MS. Spectral data (NMR, IR, and/or MS), and assignments of ^1^H and ^13^C signals for the synthesized esters are given below and in the Supplementary Materials (Figures S1–S159 and Tables S3–S9).

*1-Methylhexyl formate* (**11a**): RI = 955 (DB-5MS column); MS (EI), *m*/*z* (%) 99 (6), 98 (23), 88 (8), 86 (51), 84 (79), 83 (20), 73 (15), 71 (5), 70 (39), 69 (33), 57 (28), 56 (65), 55 (47), 51 (29), 49 (89), 47 (19), 45 (100), 43 (35), 42 (16), 41 (50), 39 (17).

*2-Methylhexyl formate* (**12a**): RI = 980 (DB-5MS column); MS (EI), *m*/*z* (%) 98 (5), 83 (13), 74 (11), 71 (13), 70 (84), 69 (71), 57 (14), 56 (63), 55 (100), 54 (6), 44 (5), 43 (78), 42 (17), 41 (57), 40 (6), 39 (20).

*3-Methylhexyl formate* (**13a**): RI = 986 (DB-5MS column); MS (EI), *m*/*z* (%) 87 (5), 83 (12), 74 (9), 71 (12), 70 (100), 69 (84), 68 (7), 59 (6), 57 (26), 56 (59), 55 (81), 53 (7), 44 (14), 43 (61), 42 (29), 41 (82), 40 (23), 39 (22).

*4-Methylhexyl formate* (**14a**): RI = 1007 (DB-5MS column); MS (EI), *m*/*z* (%) 98 (6), 85 (5), 83 (11), 74 (17), 73 (7), 71 (14), 70 (94), 69 (76), 67 (7), 59 (11), 57 (23), 56 (68), 55 (100), 53 (8), 45 (13), 44 (30), 43 (82), 42 (25), 41 (79), 40 (50), 39 (24).

*5-Methylhexyl formate* (**15a**): RI = 994 (DB-5MS column); MS (EI), *m*/*z* (%) 101 (5), 83 (24), 70 (36), 69 (35), 57 (24), 56 (100), 55 (66), 43 (43), 42 (15), 41 (50), 39 (14).

*Heptyl formate* (**16a**): RI = 1026 (DB-5MS column); MS (EI), *m*/*z* (%) 98 (6), 86 (9), 84 (16), 83 (11), 71 (5), 70 (100), 69 (57), 68 (16), 67 (5), 57 (23), 56 (90), 55 (63), 54 (7), 51 (6), 49 (16), 47 (6), 43 (35), 42 (36), 41 (69), 39 (20).

*1-Methylbutyl 2,2-dimethylbutanoate* (**1b**): MS (EI), *m*/*z* (%) 117 (16), 116 (19), 101 (24), 100 (8), 99 (95), 84 (11), 71 (33), 70 (36), 61 (7), 60 (15), 59 (22), 58 (22), 57 (100), 56 (15), 55 (24), 49 (11), 45 (5), 43 (73), 42 (13), 41 (41), 39 (16).

*2-Methylbutyl 2,2-dimethylbutanoate* (**2b**): MS (EI), *m*/*z* (%) 117 (8), 99 (18), 88 (9), 72 (6), 71 (100), 70 (53), 55 (15), 43 (54), 42 (8), 41 (19).

*3-Methylbutyl 2,2-dimethylbutanoate* (**3b**): MS (EI), *m*/*z* (%) 158 (7), 117 (11), 99 (9), 88 (8), 72 (6), 71 (100), 70 (54), 55 (19), 43 (59), 42 (7), 41 (23), 39 (9).

*Pentyl 2,2-dimethylbutanoate* (**4b**): MS (EI), *m*/*z* (%) 158 (10), 117 (40), 101 (5), 99 (14), 88 (11), 72 (5), 71 (100), 70 (27), 55 (16), 43 (59), 42 (9), 41 (25), 39 (10).

*1-Methylbutyl 3,3-dimethylbutanoate* (**1c**): MS (EI), *m*/*z* (%) 143 (5), 117 (16), 116 (7), 101 (22), 100 (5), 99 (78), 87 (5), 71 (27), 70 (37), 61 (6), 60 (17), 59 (18), 57 (100), 56 (12), 55 (26), 45 (5), 44 (18), 43 (52), 42 (13), 41 (35), 40 (35), 39 (11).

*2-Methylbutyl 3,3-dimethylbutanoate* (**2c**): MS (EI), *m*/*z* (%) 117 (6), 101 (16), 100 (7), 99 (100), 71 (57), 70 (78), 59 (14), 57 (73), 56 (18), 55 (25), 43 (44), 42 (11), 41 (43), 39 (16).

*3-Methylbutyl 3,3-dimethylbutanoate* (**3c**): MS (EI), *m*/*z* (%) 117 (9), 101 (7), 100 (5), 99 (50), 72 (5), 71 (58), 70 (100), 69 (5), 59 (10), 57 (54), 56 (12), 55 (31), 43 (51), 42 (11), 41 (45), 39 (16).

*Pentyl 3,3-dimethylbutanoate* (**4c**): MS (EI), *m*/*z* (%) 130 (11), 117 (51), 115 (8), 102 (5), 101 (33), 100 (9), 99 (95), 83 (5), 72 (5), 71 (34), 70 (100), 69 (8), 61 (23), 60 (20), 59 (29), 57 (96), 56 (16), 55 (35), 53 (6), 43 (70), 42 (24), 41 (71), 40 (5), 39 (25).

*1-Methylbutyl 2,3-dimethylbutanoate* (**1d**): MS (EI), *m*/*z* (%) 101 (22), 99 (6), 98 (19), 87 (5), 81 (8), 74 (11), 70 (16), 69 (17), 67 (6), 59 (7), 58 (6), 57 (100), 56 (35), 55 (33), 54 (7), 45 (15), 44 (48), 43 (35), 42 (13), 41 (32), 40 (91), 39 (14).

*2-Methylbutyl 2,3-dimethylbutanoate* (**2d**): MS (EI), *m*/*z* (%) 144 (5), 117 (11), 101 (8), 99 (55), 74 (38), 72 (5), 71 (100), 70 (73), 57 (8), 56 (14), 55 (36), 53 (5), 43 (76), 42 (13), 41 (31), 40 (24), 39 (14).

*3-Methylbutyl 2,3-dimethylbutanoate* (**3d**): MS (EI), *m*/*z* (%) 117 (19), 101 (8), 99 (27), 74 (23), 71 (97), 70 (100), 69 (6), 59 (7), 56 (11), 55 (31), 43 (77), 42 (9), 41 (30), 39 (10).

*Pentyl 2,3-dimethylbutanoate* (**4d**): MS (EI), *m*/*z* (%) 144 (6), 118 (5), 117 (66), 115 (5), 101 (12), 99 (37), 75 (12), 74 (69), 73 (5), 72 (5), 71 (70), 70 (45), 69 (6), 57 (7), 56 (14), 55 (34), 53 (5), 44 (15), 43 (100), 42 (20), 41 (40), 40 (24), 39 (16).

*1-Methylbutyl 2-methylpentanoate* (**1e epimer I**): MS (EI), *m*/*z* (%) 144 (5), 117 (19), 99 (44), 87 (6), 74 (31), 71 (74), 70 (23), 56 (6), 55 (13), 45 (8), 44 (46), 43 (67), 42 (9), 41 (21), 40 (100).

*1-Methylbutyl 2-methylpentanoate* (**1e epimer II**): MS (EI), *m*/*z* (%) 117 (28), 116 (6), 100 (5), 99 (50), 87 (7), 74 (35), 71 (100), 70 (29), 69 (6), 56 (6), 55 (19), 45 (11), 44 (47), 43 (78), 42 (12), 41 (28), 40 (99), 39 (9).

*2-Methylbutyl 2-methylpentanoate* (**2e**): MS (EI), *m*/*z* (%) 144 (6), 117 (17), 99 (65), 87 (9), 74 (30), 71 (100), 70 (71), 69 (5), 56 (8), 55 (27), 43 (73), 41 (35), 42 (13), 39 (13).

*3-Methylbutyl 2-methylpentanoate* (**3e**): MS (EI), *m*/*z* (%) 117 (25), 99 (31), 87 (7), 74 (14), 71 (86), 70 (100), 69 (8), 56 (8), 55 (35), 43 (83), 42 (13), 41 (40), 39 (15).

*Pentyl 2-methylpentanoate* (**4e**): MS (EI), *m*/*z* (%) 144 (8), 118 (7), 117 (100), 115 (11), 99 (42), 87 (15), 75 (7), 74 (50), 71 (65), 70 (39), 69 (9), 56 (9), 55 (27), 43 (86), 42 (19), 41 (43), 39 (16).

*1-Methylbutyl 3-methylpentanoate* (**1f**): MS (EI), *m*/*z* (%) 117 (22), 116 (7), 99 (60), 87 (13), 71 (37), 70 (30), 69 (6), 61 (10), 60 (23), 56 (12), 55 (19), 45 (8), 44 (45), 43 (47), 42 (13), 41 (29), 40 (100), 39 (9).

*2-Methylbutyl 3-methylpentanoate* (**2f**): MS (EI), *m*/*z* (%) 117 (11), 100 (7), 99 (100), 87 (11), 72 (5), 71 (72), 70 (91), 69 (7), 61 (5), 60 (14), 57 (12), 56 (6), 55 (25), 43 (55), 42 (16), 41 (34), 39 (12).

*3-Methylbutyl 3-methylpentanoate* (**3f**): Yield 75%; IR (cm^−1^) 2961, 2877, 1734, 1463, 1382, 1368, 1286, 1245, 1181, 1125, 1097, 1052, 987, 774, 738, 706; ^1^H NMR (400 MHz, CDCl_3_) 4.10 (triplet, *J* = 6.9 Hz, 2H, H-1′), 2.31 (doublet of doublets, *J* = 14.6, 8.1 Hz, 1H, H-2a), 2.09 (doublet of doublets, *J* = 14.6, 8.1 Hz, 1H, H-2b), 1.94–1.82 (multiplet, 1H, H-3), 1.68 (nonet, *J* = 6.9 Hz, 1H, H-3′), 1.52 (pseudo quartet, *J* = 6.9 Hz, 2H, H-2′), 1.47–1.31 (multiplet, 1H, H-4a), 1.29–1.13 (multiplet, 1H, H-4b), 0.93 (doublet, *J* = 6.7 Hz, 3H, H-6), 0.92 (doublet, *J* = 6.9 Hz, 6H, H-4′ and H-5′), 0.88 (triplet, *J* = 7.4 Hz, 3H, H-5); ^13^C NMR (101 MHz, CDCl_3_) 173.53 (C-1), 62.82 (C-1′), 41.61 (C-2), 37.39 (C-2′), 31.97 (C-3), 29.34 (C-4), 25.08 (C-3′), 22.46 (C-4′ and C-5′), 19.28 (C-6), 11.28 (C-5); MS (EI), *m*/*z* (%) 117 (15), 99 (46), 87 (7), 71 (57), 70 (100), 69 (8), 57 (7), 56 (5), 55 (29), 43 (54), 42 (14), 41 (33), 39 (13).

*Pentyl 3-methylpentanoate* (**4f**): MS (EI), *m*/*z* (%) 130 (6), 118 (7), 117 (95), 115 (7), 100 (7), 99 (92), 87 (26), 73 (9), 71 (64), 70 (100), 69 (15), 61 (22), 60 (24), 57 (14), 56 (8), 55 (36), 43 (81), 42 (31), 41 (57), 40 (5), 39 (20).

*1-Methylbutyl 4-methylpentanoate* (**1g**): MS (EI), *m*/*z* (%) 143 (7), 117 (31), 116 (10), 101 (11), 100 (7), 99 (100), 87 (12), 81 (37), 74 (6), 73 (16), 71 (39), 70 (54), 69 (9), 60 (7), 57 (23), 56 (12), 55 (45), 53 (6), 45 (7), 44 (17), 43 (95), 42 (19), 41 (42), 40 (25), 39 (13).

*2-Methylbutyl 4-methylpentanoate* (**2g**): MS (EI), *m*/*z* (%) 117 (13), 101 (11), 100 (9), 99 (100), 83 (6), 81 (45), 73 (12), 71 (46), 70 (95), 69 (8), 60 (5), 57 (14), 56 (13), 55 (37), 53 (5), 43 (72), 42 (13), 41 (44), 39 (16).

*3-Methylbutyl 4-methylpentanoate* (**3g**): MS (EI), *m*/*z* (%) 143 (5), 117 (15), 101 (9), 99 (49), 83 (5), 81 (30), 73 (7), 71 (42), 70 (100), 69 (9), 56 (9), 55 (40), 43 (68), 42 (12), 41 (39), 39 (14).

*Pentyl 4-methylpentanoate* (**4g**): MS (EI), *m/z* (%) 143 (5), 118 (6), 117 (100), 115 (6), 101 (18), 100 (6), 99 (71), 87 (5), 83 (12), 81 (45), 74 (7), 73 (25), 71 (27), 70 (74), 69 (14), 56 (12), 55 (43), 53 (5), 43 (83), 42 (23), 41 (55), 39 (20).

*1-Methylbutyl hexanoate* (**1h**): MS (EI), *m*/*z* (%) 117 (18), 116 (7), 100 (5), 99 (48), 87 (5), 71 (23), 70 (25), 60 (10), 56 (7), 55 (15), 45 (5), 44 (41), 43 (44), 42 (10), 41 (18), 40 (100), 39 (8).

*2-Methylbutyl hexanoate* (**2h**): MS (EI), *m*/*z* (%) 117 (12), 100 (7), 99 (100), 87 (7), 73 (7), 71 (49), 70 (81), 69 (5), 60 (9), 56 (5), 55 (27), 43 (55), 42 (16), 41 (31), 39 (12).

*3-Methylbutyl hexanoate* (**3h**): MS (EI), *m*/*z* (%) 117 (17), 99 (51), 73 (5), 71 (44), 70 (100), 69 (8), 60 (5), 55 (35), 43 (59), 42 (16), 41 (32), 39 (13).

*Pentyl hexanoate* (**4h**): MS (EI), *m*/*z* (%) 118 (6), 117 (100), 115 (5), 100 (6), 99 (80), 87 (13), 73 (15), 71 (38), 70 (77), 69 (13), 60 (14), 56 (5), 55 (35), 43 (72), 42 (28), 41 (46), 39 (17).

*1-Methylpentyl 2,2-dimethylbutanoate* (**5b**): MS (EI), *m*/*z* (%) 117 (15), 101 (24), 100 (8), 99 (95), 85 (15), 84 (40), 83 (7), 71 (13), 69 (19), 67 (6), 61 (5), 60 (13), 59 (17), 57 (100), 56 (32), 55 (49), 53 (6), 44 (10), 43 (56), 42 (22), 41 (49), 40 (18), 39 (17).

*2-Methylpentyl 2,2-dimethylbutanoate* (**6b**): MS (EI), *m*/*z* (%) 117 (11), 99 (20), 88 (10), 85 (12), 84 (33), 72 (6), 71 (100), 70 (17), 69 (9), 57 (6), 56 (19), 55 (15), 43 (66), 42 (9), 41 (26), 39 (9).

*3-Methylpentyl 2,2-dimethylbutanoate* (**7b**): MS (EI), *m*/*z* (%) 172 (7), 117 (16), 99 (8), 88 (8), 85 (15), 84 (47), 72 (6), 71 (100), 70 (20), 69 (23), 57 (11), 56 (10), 55 (22), 43 (65), 42 (8), 41 (31), 39 (10).

*4-Methylpentyl 2,2-dimethylbutanoate* (**8b**): MS (EI), *m/z* (%) 172 (7), 117 (42), 99 (11), 88 (9), 85 (11), 84 (21), 72 (6), 71 (100), 70 (20), 69 (11), 57 (5), 56 (12), 55 (16), 43 (73), 42 (10), 41 (30), 39 (10).

*Hexyl 2,2-dimethylbutanoate* (**9b**): MS (EI), *m*/*z* (%) 172 (7), 117 (34), 99 (23), 88 (9), 72 (6), 71 (100), 70 (15), 69 (5), 56 (7), 55 (15), 43 (53), 42 (8), 41 (24), 39 (9).

*1-Methylpentyl 3,3-dimethylbutanoate* (**5c**): MS (EI), *m*/*z* (%) 143 (5), 117 (13), 101 (23), 100 (7), 99 (86), 85 (13), 84 (41), 83 (6), 71 (9), 69 (19), 60 (13), 59 (22), 57 (100), 56 (32), 55 (52), 53 (7), 45 (6), 44 (18), 43 (68), 42 (28), 41 (58), 40 (44), 39 (25).

*2-Methylpentyl 3,3-dimethylbutanoate* (**6c**): MS (EI), *m*/*z* (%) 129 (5), 117 (9), 101 (16), 100 (7), 99 (100), 85 (41), 84 (62), 71 (14), 69 (13), 59 (13), 57 (73), 56 (40), 55 (21), 53 (5), 43 (58), 42 (12), 41 (42), 39 (16).

*3-Methylpentyl 3,3-dimethylbutanoate* (**7c**): MS (EI), *m*/*z* (%) 117 (16), 101 (6), 100 (5), 99 (49), 85 (53), 84 (100), 83 (6), 71 (10), 69 (44), 59 (10), 57 (76), 56 (32), 55 (33), 53 (6), 43 (45), 42 (9), 41 (55), 39 (16).

*4-Methylpentyl 3,3-dimethylbutanoate* (**8c**): MS (EI), *m*/*z* (%) 157 (5), 117 (49), 116 (8), 101 (13), 100 (7), 99 (67), 85 (63), 84 (81), 83 (9), 71 (13), 69 (32), 61 (7), 59 (14), 57 (100), 56 (71), 55 (30), 53 (6), 43 (83), 42 (17), 41 (80), 40 (5), 39 (22).

*Hexyl 3,3-dimethylbutanoate* (**9c**): MS (EI), *m*/*z* (%) 117 (58), 101 (34), 100 (8), 99 (83), 85 (17), 84 (62), 83 (8), 71 (13), 69 (22), 61 (40), 60 (12), 59 (23), 57 (100), 56 (52), 55 (35), 53 (6), 43 (73), 42 (19), 41 (72), 40 (5), 39 (22).

*1-Methylpentyl 2,3-dimethylbutanoate* (**5d**): MS (EI), *m*/*z* (%) 117 (19), 112 (11), 101 (26), 99 (48), 85 (25), 84 (27), 83 (12), 75 (10), 74 (43), 72 (5), 71 (57), 70 (23), 69 (19), 68 (5), 67 (6), 59 (5), 58 (6), 57 (100), 56 (40), 55 (53), 53 (6), 45 (10), 44 (31), 43 (85), 42 (21), 41 (57), 40 (69), 39 (17).

*2-Methylpentyl 2,3-dimethylbutanoate* (**6d**): MS (EI), *m*/*z* (%) 158 (5), 141 (5), 129 (5), 117 (21), 101 (11), 100 (5), 99 (79), 85 (39), 84 (62), 83 (8), 75 (7), 74 (49), 72 (6), 71 (92), 70 (8), 69 (19), 59 (8), 57 (23), 56 (63), 55 (31), 43 (100), 42 (11), 41 (46), 39 (15).

*3-Methylpentyl 2,3-dimethylbutanoate* (**7d**): MS (EI), *m*/*z* (%) 117 (19), 101 (5), 99 (22), 85 (39), 84 (93), 74 (14), 71 (51), 70 (10), 69 (55), 67 (9), 57 (25), 56 (32), 55 (36), 53 (7), 49 (6), 44 (22), 43 (100), 42 (13), 41 (57), 40 (57), 39 (17).

*4-Methylpentyl 2,3-dimethylbutanoate* (**8d**): MS (EI), *m*/*z* (%) 141 (5), 117 (27), 101 (7), 99 (34), 86 (5), 85 (50), 84 (100), 83 (8), 75 (8), 74 (25), 71 (63), 70 (10), 69 (50), 59 (10), 57 (35), 56 (36), 55 (39), 45 (7), 43 (86), 42 (8), 41 (44), 39 (11).

*Hexyl 2,3-dimethylbutanoate* (**9d**): MS (EI), *m*/*z* (%) 158 (5), 118 (6), 117 (62), 101 (9), 99 (29), 85 (10), 84 (35), 83 (8), 82 (6), 75 (12), 74 (57), 71 (54), 70 (9), 69 (22), 67 (10), 57 (16), 56 (40), 55 (48), 54 (7), 53 (9), 51 (7), 49 (9), 45 (5), 44 (47), 43 (100), 42 (26), 41 (62), 40 (97), 39 (23).

*1-Methylpentyl 2-methylpentanoate* (**5e epimer I**): MS (EI), *m*/*z* (%) 117 (28), 100 (5), 99 (72), 97 (6), 87 (7), 85 (41), 84 (29), 83 (16), 81 (6), 74 (38), 71 (68), 69 (23), 67 (8), 57 (24), 56 (24), 55 (43), 53 (5), 44 (9), 43 (100), 42 (17), 41 (46), 40 (10), 39 (15).

*1-Methylpentyl 2-methylpentanoate* (**5e epimer II**): MS (EI), *m*/*z* (%) 143 (5), 117 (28), 100 (5), 99 (74), 97 (5), 87 (7), 85 (38), 84 (28), 83 (9), 74 (37), 71 (63), 70 (7), 69 (20), 57 (17), 56 (23), 55 (34), 44 (8), 43 (100), 42 (15), 41 (38), 40 (10), 39 (11).

*2-Methylpentyl 2-methylpentanoate* (**6e**): MS (EI), *m*/*z* (%) 129 (6), 117 (25), 100 (5), 99 (77), 87 (9), 85 (32), 84 (58), 74 (33), 71 (84), 69 (21), 57 (11), 56 (48), 55 (24), 43 (100), 42 (16), 41 (49), 39 (15).

*3-Methylpentyl 2-methylpentanoate* (**7e**): MS (EI), *m*/*z* (%) 117 (35), 99 (28), 87 (6), 85 (37), 84 (100), 74 (12), 71 (55), 70 (6), 69 (56), 57 (20), 56 (27), 55 (34), 53 (5), 43 (80), 41 (50), 39 (15).

*4-Methylpentyl 2-methylpentanoate* (**8e**): MS (EI), *m*/*z* (%) 118 (6), 117 (90), 99 (32), 87 (7), 85 (29), 84 (53), 83 (5), 75 (6), 74 (16), 71 (54), 69 (28), 57 (13), 56 (39), 55 (22), 43 (100), 42 (15), 41 (50), 39 (14).

*Hexyl 2-methylpentanoate* (**9e**): MS (EI), *m*/*z* (%) 158 (5), 129 (6), 118 (7), 117 (100), 99 (35), 87 (13), 85 (7), 84 (30), 75 (12), 74 (45), 71 (52), 69 (17), 57 (10), 56 (29), 55 (27), 43 (86), 42 (16), 41 (44), 39 (14).

*1-Methylpentyl 3-methylpentanoate* (**5f**): MS (EI), *m*/*z* (%) 143 (6), 117 (24), 116 (13), 100 (6), 99 (100), 87 (10), 85 (15), 84 (32), 71 (33), 69 (12), 61 (8), 60 (22), 57 (13), 56 (26), 55 (21), 45 (5), 44 (10), 43 (65), 42 (15), 41 (32), 40 (21), 39 (9).

*2-Methylpentyl 3-methylpentanoate* (**6f**): MS (EI), *m*/*z* (%) 117 (14), 100 (7), 99 (100), 87 (10), 85 (27), 84 (60), 71 (42), 69 (18), 57 (15), 56 (36), 55 (19), 43 (67), 42 (17), 41 (42), 39 (13).

*3-Methylpentyl 3-methylpentanoate* (**7f**): MS (EI), *m*/*z* (%) 117 (26), 99 (51), 87 (7), 85 (32), 84 (100), 83 (5), 71 (33), 70 (6), 69 (57), 60 (5), 57 (25), 56 (28), 55 (33), 53 (5), 43 (58), 42 (14), 41 (49), 39 (15).

*4-Methylpentyl 3-methylpentanoate* (**8f**): MS (EI), *m*/*z* (%) 157 (8), 118 (6), 117 (89), 116 (9), 100 (5), 99 (69), 87 (12), 85 (36), 84 (72), 83 (9), 73 (6), 71 (41), 70 (5), 69 (38), 61 (8), 60 (8), 57 (25), 56 (71), 55 (27), 53 (5), 43 (100), 42 (23), 41 (68), 40 (5), 39 (20).

*Hexyl 3-methylpentanoate* (**9f**): MS (EI), *m*/*z* (%) 118 (7), 117 (100), 116 (6), 101 (6), 99 (80), 87 (23), 85 (14), 84 (59), 83 (6), 73 (8), 71 (43), 70 (6), 69 (31), 61 (33), 60 (16), 57 (22), 56 (52), 55 (36), 53 (5), 43 (88), 42 (25), 41 (63), 39 (18).

*1-Methylpentyl 4-methylpentanoate* (**5g**): MS (EI), *m*/*z* (%) 143 (8), 117 (25), 116 (12), 101 (16), 100 (9), 99 (100), 85 (17), 84 (37), 81 (32), 73 (12), 71 (13), 69 (16), 57 (25), 56 (25), 55 (28), 44 (5), 43 (75), 42 (13), 41 (32), 39 (9).

*2-Methylpentyl 4-methylpentanoate* (**6g**): MS (EI), *m*/*z* (%) 117 (18), 101 (12), 100 (7), 99 (100), 85 (26), 84 (65), 81 (45), 73 (10), 71 (17), 70 (5), 69 (22), 57 (14), 56 (48), 55 (29), 43 (86), 42 (14), 41 (49), 39 (16).

*3-Methylpentyl 4-methylpentanoate* (**7g**): Yield 78%; IR (cm^−1^) 2959, 2931, 2874, 1737, 1464, 1368, 1329, 1265, 1176, 1104, 1060, 974, 775; ^1^H NMR (400 MHz, CDCl_3_) 4.16–4.03 (multiplet, 2H, H-1′), 2.32–2.27 (multiplet, 2H, H-2), 1.72–1.61 (multiplet, 1H, H-2′a), 1.61–1.48 (overlapping peaks, 3H, H-3 and H-4), 1.48–1.41 (overlapping peaks, 2H, H-2′b and H-3′), 1.41–1.31 (multiplet, 1H, H-4′a), 1.19 (pseudo doublet of quintets, *J* = 13.5, 7.3 Hz, 1H, H-4′b), 0.90 (doublet, *J* = 6.4 Hz, 9H, H-5, H-6 and H-6′), 0.88 (triplet, *J* = 7.4 Hz, 3H, H-5′); ^13^C NMR (101 MHz, CDCl_3_) 174.19 (C-1), 62.87 (C-1′), 35.13 (C-2′), 33.82 (C-3), 32.48 (C-2), 31.41 (C-3′), 29.38 (C-4′), 27.68 (C-4), 22.23 (C-5 and C-6), 19.02 (C-6′), 11.22 (C-5′); MS (EI), *m*/*z* (%) 143 (6), 117 (30), 101 (11), 99 (44), 85 (34), 84 (100), 83 (10), 81 (34), 73 (6), 71 (13), 70 (7), 69 (65), 57 (22), 56 (33), 55 (44), 53 (6), 43 (72), 42 (10), 41 (54), 39 (16).

*4-Methylpentyl 4-methylpentanoate* (**8g**): MS (EI), *m*/*z* (%) 157 (10), 118 (5), 117 (73), 101 (14), 99 (51), 85 (33), 84 (71), 83 (15), 81 (39), 71 (14), 70 (6), 69 (45), 57 (19), 56 (59), 55 (37), 53 (6), 43 (100), 42 (15), 41 (64), 39 (18).

*Hexyl 4-methylpentanoate* (**9g**): MS (EI), *m*/*z* (%) 118 (6), 117 (100), 101 (19), 100 (5), 99 (61), 85 (10), 84 (48), 83 (14), 81 (41), 74 (6), 73 (20), 71 (14), 70 (5), 69 (29), 61 (12), 60 (5), 57 (15), 56 (50), 55 (42), 53 (5), 43 (89), 42 (18), 41 (59), 39 (17).

*1-Methylpentyl hexanoate* (**5h**): MS (EI), *m*/*z* (%) 143 (8), 117 (21), 116 (13), 100 (7), 99 (100), 85 (14), 84 (32), 73 (7), 71 (23), 69 (13), 60 (16), 57 (8), 56 (23), 55 (23), 44 (9), 43 (68), 42 (17), 41 (26), 40 (17), 39 (8).

*2-Methylpentyl hexanoate* (**6h**): MS (EI), *m*/*z* (%) 117 (17), 100 (7), 99 (100), 87 (6), 85 (19), 84 (52), 73 (6), 71 (29), 69 (18), 60 (7), 57 (7), 56 (36), 55 (20), 43 (66), 42 (16), 41 (36), 39 (12).

*3-Methylpentyl hexanoate* (**7h**): MS (EI), *m*/*z* (%) 143 (6), 117 (32), 99 (55), 85 (25), 84 (100), 83 (5), 73 (5), 71 (25), 70 (6), 69 (66), 60 (5), 57 (17), 56 (28), 55 (38), 43 (61), 42 (15), 41 (47), 39 (14).

*4-Methylpentyl hexanoate* (**8h**): MS (EI), *m*/*z* (%) 157 (11), 118 (6), 117 (98), 116 (6), 100 (5), 99 (70), 87 (7), 85 (27), 84 (76), 83 (9), 73 (8), 71 (29), 70 (6), 69 (48), 60 (6), 57 (15), 56 (62), 55 (32), 53 (5), 43 (100), 42 (24), 41 (64), 39 (17).

*Hexyl hexanoate* (**9h**): MS (EI), *m*/*z* (%) 118 (6), 117 (100), 116 (5), 100 (5), 99 (71), 87 (11), 85 (9), 84 (50), 73 (14), 71 (27), 70 (5), 69 (28), 61 (14), 60 (10), 57 (9), 56 (45), 55 (35), 43 (79), 42 (22), 41 (50), 39 (15).

*(Z)-Hex-3-en-1-yl 2,2-dimethylbutanoate* (**10b**): MS (EI), *m*/*z* (%) 99 (29), 72 (6), 71 (100), 70 (10), 55 (23), 53 (7), 43 (46), 42 (12), 41 (32), 40 (5), 39 (25).

*(Z)-Hex-3-en-1-yl 3,3-dimethylbutanoate* (**10c**): MS (EI), *m*/*z* (%) 101 (5), 99 (27), 83 (26), 82 (69), 81 (7), 71 (10), 67 (81), 59 (9), 57 (76), 56 (19), 55 (69), 54 (12), 53 (18), 51 (6), 43 (22), 42 (17), 41 (100), 40 (11), 39 (55).

*(Z)-Hex-3-en-1-yl 2,3-dimethylbutanoate* (**10d**): MS (EI), *m*/*z* (%) 107 (6), 106, (6), 105 (7), 99 (16), 94 (6), 83 (16), 82 (100), 81 (7), 80 (5), 79 (8), 78 (14), 74 (6), 71 (56), 68 (7), 67 (80), 56 (10), 55 (31), 54 (7), 53 (10), 52 (7), 51 (18), 50 (6), 44 (18), 43 (46), 42 (15), 41 (36), 40 (25), 39 (18).

*(Z)-Hex-3-en-1-yl 2-methylpentanoate* (**10e**): MS (EI), *m*/*z* (%) 99 (15), 83 (8), 82 (86), 81 (7), 79 (8), 74 (5), 71 (56), 69 (9), 68 (8), 67 (81), 65 (8), 56 (9), 55 (51), 54 (16), 53 (24), 51 (9), 44 (7), 43 (87), 42 (25), 41 (100), 40 (20), 39 (67), 38 (7).

*(Z)-Hex-3-en-1-yl 3-methylpentanoate* (**10f**): Yield: 68%; IR (cm^−1^) 2963, 2934, 2877, 1736, 1461, 1382, 1286, 1243, 1178, 1125, 1097, 1045, 1007, 720; ^1^H NMR (400 MHz, CDCl_3_) 5.54–5.46 (multiplet, 1H, H-4′), 5.36–5.28 (multiplet, 1H, H-3′), 4.07 (triplet, *J* = 6.9 Hz, 2H, H-1′), 2.38 (pseudo quartet of doublets of triplets, *J* = 7.0, 1.4, 0.7 Hz, 2H, H-2′), 2.30 (doublet of doublets, *J* = 14.7, 6.1 Hz, 1H, H-2a), 2.10 (doublet of doublets, *J* = 14.7, 8.2 Hz, 1H, H-2b), 2.10–2.02 (multiplet, 1H, H-5′), 1.89 (doublet of doublets of quartets of doublets of doublets, *J* = 8.2, 7.4, 6.7, 6.1, 5.7 Hz, 1H, H-3), 1.36 (doublet of quartets of doublets, *J* = 13.4, 7.4, 5.7 Hz, 1H, H-4a), 1.22 (pseudo doublet of quintets, *J* = 13.4, 7.4 Hz, 1H, H-4b), 0.97 (pseudo triplet, *J* = 7.5 Hz, 3H, H-6′), 0.93 (doublet, *J* = 6.7 Hz, 3H, H-6), 0.89 (pseudo triplet, *J* = 7.4 Hz, 3H, H-5); ^13^C NMR (101 MHz, CDCl_3_) 173.42 (C-1), 134.47 (C-4′), 123.81 (C-3′), 63.70 (C-1′), 41.52 (C-2), 31.92 (C-3), 29.33 (C-4), 26.79 (C-2′), 20.61 (C-5′), 19.27 (C-6), 14.23 (C-6′), 11.28 (C-5); MS (EI), *m*/*z* (%) 99 (41), 83 (16), 82 (100), 81 (8), 71 (32), 68 (6), 67 (80), 57 (6), 55 (27), 54 (6), 42 (8), 41 (29), 39 (13).

*(Z)-Hex-3-en-1-yl 4-methylpentanoate* (**10g**): MS (EI), *m*/*z* (%) 101 (5), 99 (24), 83 (15), 82 (87), 81 (36), 71 (12), 67 (100), 56 (11), 55 (58), 54 (12), 53 (16), 51 (5), 43 (78), 42 (15), 41 (92), 40 (9), 39 (45).

*(Z)-Hex-3-en-1-yl hexanoate* (**10h**): MS (EI), *m*/*z* (%) 99 (38), 83 (12), 82 (100), 81 (7), 71 (24), 68 (7), 67 (93), 55 (30), 54 (7), 43 (35), 42 (10), 41 (36), 39 (15).

*1-Methylhexyl angelate* (**11o**): MS (EI), *m*/*z* (%) 100 (8), 84 (7), 83 (100), 55 (59), 53 (7), 39 (10).

*2-Methylhexyl angelate* (**12o**): MS (EI), *m*/*z* (%) 101 (7), 100 (35), 99 (5), 98 (15), 91 (6), 84 (5), 83 (27), 81 (9), 79 (9), 70 (13), 69 (25), 67 (10), 57 (29), 56 (65), 55 (54), 53 (11), 44 (31), 43 (19), 42 (11), 41 (51), 40 (100), 39 (27).

*3-Methylhexyl angelate* (**13o**): MS (EI), *m*/*z* (%) 101 (27), 100 (64), 99 (7), 98 (19), 85 (5), 83 (60), 82 (11), 71 (5), 70 (26), 69 (34), 58 (5), 57 (100), 56 (22), 55 (79), 53 (11), 44 (10), 43 (39), 41 (32), 40 (19), 39 (17).

*4-Methylhexyl angelate* (**14o**): MS (EI), *m*/*z* (%) 101 (6), 100 (14), 83 (13), 70 (8), 69 (15), 57 (23), 56 (7), 55 (24), 54 (5), 44 (30), 43 (10), 41 (30), 40 (100), 39 (10).

*5-Methylhexyl angelate* (**15o**): MS (EI), *m*/*z* (%) 101 (11), 100 (39), 84 (10), 83 (100), 82 (9), 69 (6), 57 (27), 56 (13), 55 (73), 54 (9), 53 (9), 51 (5), 49 (7), 43 (13), 41 (13), 39 (15).

*Heptyl angelate* (**16o**): MS (EI), *m*/*z* (%) 101 (20), 100 (100), 85 (7), 83 (53), 82 (14), 69 (5), 57 (28), 56 (10), 55 (54), 43 (15), 42 (5), 41 (18), 39 (12).

*1-Methylhexyl tiglate* (**11p**): MS (EI), *m*/*z* (%) 101 (16), 98 (6), 86 (5), 84 (13), 83 (100), 82 (6), 69 (6), 56 (9), 55 (58), 53 (8), 49 (9), 43 (6), 39 (12).

*2-Methylhexyl tiglate* (**12p**): MS (EI), *m*/*z* (%) 101 (35), 100 (9), 99 (7), 98 (44), 84 (6), 83 (99), 81 (8), 70 (24), 69 (42), 67 (6), 58 (5), 57 (29), 56 (100), 55 (83), 54 (10), 53 (14), 51 (5), 44 (11), 43 (28), 42 (13), 41 (67), 40 (30). 39 (33).

*3-Methylhexyl tiglate* (**13p**): MS (EI), *m*/*z* (%) 101 (65), 100 (8). 99 (6), 98 (65), 84 (7), 83 (100), 82 (5), 71 (6), 70 (58), 69 (66), 57 (23), 56 (35), 55 (93), 54 (8), 53 (11), 43 (25), 42 (6), 41 (30), 40 (8), 39 (16).

*4-Methylhexyl tiglate* (**14p**): MS (EI), *m*/*z* (%) 102 (6), 101 (89), 100 (14), 99 (5), 98 (40), 84 (7), 83 (81), 82 (7), 81 (8), 71 (7), 70 (77), 69 (53), 67 (9), 57 (53), 56 (37), 55 (100), 54 (9), 53 (19), 44 (16), 43 (28), 42 (15), 41 (77), 40 (39), 39 (34).

*5-Methylhexyl tiglate* (**15p**): Yield: 75%; IR (cm^−1^) 2955, 2870, 1712, 1654, 1467, 1385, 1367, 1255, 1136, 1079, 734, 656; UV (MeCN, 0.05 M) λ_max_ nm (log ε): 199 (2.87), 216 (1.65), 274 (0.21); ^1^H NMR (400 MHz, CDCl_3_) 6.85 (quartet of quartets, *J* = 7.0, 1.4 Hz, 1H, H-3), 4.12 (triplet, *J* = 6.7 Hz, 2H, H-1′), 1.83 (pseudo quintet, *J* = 1.4 Hz, 3H, H-5), 1.79 (doublet of quartets, *J* = 7.0, 1.4 Hz, 3H, H-4), 1.65 (pseudo quintet, *J* = 6.7 Hz, 2H, H-2′), 1.54 (nonet, *J* = 6.6 Hz, 1H, H-5′), 1.41–1.33 (multiplet, 2H, H-3′), 1.27–1.17 (multiplet, 2H, H-4′), 0.88 (doublet, *J* = 6.6 Hz, 6H, H-6′ and H-7′); ^13^C NMR (101 MHz, CDCl_3_) 168.25 (C-1), 136.81 (C-3), 128.79 (C-2), 64.57 (C-1′), 38.54 (C-4′), 28.92 (C-2′), 27.86 (C-5′), 23.80 (C-3′), 22.55 (C-6′ and C-7′), 14.32 (C-4), 12.03 (C-5); MS (EI), *m*/*z* (%) 102 (6), 101 (100), 100 (11), 84 (6), 83 (76), 82 (5), 70 (19), 69 (18), 57 (22), 56 (35), 55 (59), 53 (7), 43 (18), 41 (19), 39 (10).

*Heptyl tiglate* (**16p**): MS (EI), *m*/*z* (%) 102 (5), 101 (87), 100 (17), 84 (9), 83 (100), 82 (8), 70 (12), 69 (9), 57 (12), 56 (19), 55 (76), 54 (8), 53 (10), 43 (11), 42 (6), 41 (19), 39 (14).

*1-Methylhexyl senecioate* (**11q**): MS (EI), *m*/*z* (%) 101 (28), 100 (44), 98 (8), 84 (6), 83 (100), 82 (7), 70 (5), 69 (8), 57 (17), 56 (17), 55 (27), 53 (5), 43 (13), 42 (5), 41 (18), 39 (11).

*2-Methylhexyl senecioate* (**12q**): MS (EI), *m*/*z* (%) 101 (22), 100 (36), 98 (12), 84 (6), 83 (100), 82 (6), 69 (5), 57 (19), 56 (17), 55 (21), 43 (8), 41 (11), 39 (7).

*3-Methylhexyl senecioate* (**13q**): MS (EI), *m*/*z* (%) 101 (36), 100 (38). 99 (5), 98 (20), 84 (6), 83 (100), 82 (8), 70 (16), 69 (19), 57 (42), 56 (11), 55 (34), 53 (7), 43 (18), 41 (18), 39 (11).

*4-Methylhexyl senecioate* (**14q**): MS (EI), *m*/*z* (%) 101 (52), 100 (37), 98 (9), 84 (6), 83 (100), 82 (9), 70 (24), 69 (17), 57 (64), 56 (12), 55 (40), 53 (9), 43 (17), 42 (7), 41 (34), 40 (10), 39 (17).

*5-Methylhexyl senecioate* (**15q**): MS (EI), *m*/*z* (%) 101 (54), 100 (61), 85 (5), 84 (6), 83 (100), 82 (11), 70 (6), 69 (7), 57 (37), 56 (13), 55 (33), 53 (6), 43 (19), 41 (18), 39 (11).

*Heptyl senecioate* (**16q**): MS (EI), *m*/*z* (%) 198 (5), 101 (59), 100 (92), 85 (8), 84 (6), 83 (100), 82 (15), 70 (5), 57 (23), 56 (9), 55 (30), 53 (6), 43 (13), 42 (5), 41 (17), 39 (11).

*1-Methylbutyl 2-methylheptanoate* (**1l epimer I**): RI = 1337 (DB-5MS column); MS (EI), *m*/*z* (%) 146 (5), 145 (43), 144 (17), 127 (56), 99 (13), 87 (15), 74 (74), 71 (58), 70 (44), 69 (9), 57 (84), 56 (13), 55 (49), 53 (6), 45 (9), 44 (35), 43 (100), 42 (25), 41 (48), 40 (65), 39 (17).

*1-Methylbutyl 2-methylheptanoate* (**1l epimer II**): RI = 1341 (DB-5MS column); MS (EI), *m*/*z* (%) 146 (6), 145 (46), 144 (14), 128 (5), 127 (57), 99 (13), 87 (21), 74 (85), 71 (62), 70 (46), 69 (10), 57 (88), 56 (11), 55 (43), 45 (8), 44 (32), 43 (100), 42 (21), 41 (48), 40 (46), 39 (19).

*2-Methylbutyl 2-methylheptanoate* (**2l**): RI = 1379 (DB-5MS column); MS (EI), *m*/*z* (%) 145 (25), 144 (18), 128 (5), 127 (53), 101 (7), 99 (17), 87 (16), 74 (50), 71 (52), 70 (100), 69 (9), 58 (5), 57 (92), 56 (14), 55 (37), 43 (59), 42 (16), 41 (52), 39 (14).

*3-Methylbutyl 2-methylheptanoate* (**3l**): RI = 1372 (DB-5MS column); MS (EI), *m*/*z* (%) 145 (17), 144 (6), 127 (14), 99 (6), 87 (8), 74 (15), 71 (39), 70 (100), 69 (7), 57 (44), 56 (8), 55 (27), 43 (46), 42 (9), 41 (30).

*Pentyl 2-methylheptanoate* (**4l**): RI = 1412 (DB-5MS column); MS (EI), *m*/*z* (%) 146 (9), 145 (100), 144 (19), 127 (30), 115 (15), 103 (5), 101 (9), 99 (10), 87 (24), 75 (12), 74 (80), 73 (5), 71 (15), 70 (55), 69 (13), 57 (76), 56 (13), 55 (34), 43 (64), 42 (20), 41 (48), 39 (11).

*1-Methylbutyl 6-methylheptanoate* (**1m**): RI = 1390 (DB-5MS column); MS (EI), *m*/*z* (%) 145 (45), 128 (7), 127 (86), 126 (12), 117 (33), 111 (14), 109 (99), 104 (76), 101 (13), 99 (24), 98 (6), 89 (5), 87 (13), 86 (37), 84 (24), 83 (58), 82 (62), 74 (9), 69 (31), 67 (20), 61 (44), 60 (21), 59 (8), 57 (68), 56 (31), 55 (98), 53 (6), 45 (47), 44 (44), 43 (100), 42 (25), 41 (78), 40 (7), 39 (21).

*2-Methylbutyl 6-methylheptanoate* (**2m**): RI = 1413 (DB-5MS column); MS (EI), *m*/*z* (%) 145 (6), 127 (30), 126 (5), 109 (40), 101 (6), 85 (9), 82 (16), 73 (7), 71 (37), 70 (100), 69 (6), 60 (9), 57 (22), 56 (9), 55 (34), 44 (10), 43 (49), 42 (11), 41 (31), 40 (19), 39 (8).

*3-Methylbutyl 6-methylheptanoate* (**3m**): RI = 1410 (DB-5MS column); MS (EI), *m*/*z* (%) 145 (5), 127 (9), 109 (18), 82 (10), 71 (31), 70 (100), 67 (6), 57 (14), 56 (6), 55 (39), 44 (24), 43 (51), 42 (11), 41 (30), 40 (42), 39 (11).

*Pentyl 6-methylheptanoate* (**4m**): RI = 1449 (DB-5MS column); MS (EI), *m*/*z* (%) 146 (5), 145 (54), 127 (24), 111 (6), 109 (35), 101 (9), 85 (8), 83 (17), 82 (26), 73 (17), 71 (19), 70 (100), 69 (11), 67 (8), 61 (13), 60 (16), 57 (24), 56 (8), 55 (44), 44 (8), 43 (71), 42 (21), 41 (46), 40 (11), 39 (10).

*1-Methylbutyl octanoate* (**1n**): RI = 1414 (DB-5MS column); MS (EI), *m*/*z* (%) 145 (49), 144 (28), 128 (8), 127 (100), 115 (6), 101 (17), 87 (31), 85 (10), 84 (19), 73 (12), 71 (33), 70 (76), 69 (11), 61 (7), 60 (26), 57 (62), 56 (10), 55 (73), 45 (9), 44 (29), 43 (84), 42 (36), 41 (56), 40 (44), 39 (20).

*2-Methylbutyl octanoate* (**2n**): RI = 1451 (DB-5MS column); MS (EI), *m*/*z* (%) 145 (15), 144 (9), 128 (9), 127 (100), 115 (5), 109 (5), 101 (9), 87 (7), 73 (8), 71 (32), 70 (97), 69 (7), 60 (9), 57 (57), 56 (7), 55 (39), 43 (44), 42 (16), 41 (46), 39 (14).

*3-Methylbutyl octanoate* (**3n**): RI = 1448 (DB-5MS column); MS (EI), *m*/*z* (%) 145 (11), 127 (29), 73 (5), 71 (28), 70 (100), 69 (6), 57 (26), 55 (32), 43 (40), 42 (10), 41 (27), 39 (7).

*Pentyl octanoate* (**4n**): RI = 1486 (DB-5MS column); MS (EI), *m*/*z* (%) 146 (9), 145 (100), 144 (9), 128 (6), 127 (65), 115 (10), 103 (5), 101 (13), 97 (5), 89 (9), 87 (9), 73 (19), 71 (16), 70 (90), 69 (13), 67 (5), 61 (11), 60 (17), 57 (47), 56 (7), 55 (48), 43 (57), 42 (27), 41 (53), 39 (14).

*1-Methylhexyl 2,2-dimethylbutanoate* (**11b**): MS (EI), *m*/*z* (%) 117 (16), 99 (26), 98 (12), 88 (6), 84 (5), 72 (5), 71 (100), 70 (14), 69 (5), 57 (57), 56 (7), 55 (20), 49 (9), 44 (26), 43 (61), 42 (11), 41 (30), 40 (47), 39 (11).

*2-Methylhexyl 2,2-dimethylbutanoate* (**12b**): MS (EI), *m*/*z* (%) 117 (15), 99 (24), 98 (33), 88 (11), 81 (9), 72 (7), 71 (100), 70 (47), 69 (39), 67 (9), 58 (5), 57 (48), 56 (83), 55 (64), 53 (16), 44 (33), 43 (70), 42 (23), 41 (78), 40 (62), 39 (31).

*3-Methylhexyl 2,2-dimethylbutanoate* (**13b**): MS (EI), *m*/*z* (%) 117 (16), 99 (14), 98 (40), 88 (6), 81 (10), 72 (6), 71 (100), 70 (64), 69 (50), 67 (10), 57 (39), 56 (34), 55 (68), 54 (5), 53 (12), 44 (29), 43 (81), 42 (21), 41 (64), 40 (49), 39 (27).

*4-Methylhexyl 2,2-dimethylbutanoate* (**14b**): MS (EI), *m*/*z* (%) 117 (7), 98 (8), 81 (6), 71 (25), 70 (26), 69 (16), 67 (7), 57 (15), 56 (13), 55 (30), 44 (47), 43 (22), 42 (13). 41 (37), 40 (100), 39 (17).

*5-Methylhexyl 2,2-dimethylbutanoate* (**15b**): MS (EI), *m*/*z* (%) 117 (41), 99 (10), 98 (8), 88 (9), 83 (6), 72 (6), 71 (100), 70 (41), 69 (11), 57 (34), 56 (30), 55 (29), 44 (12), 43 (74), 42 (10), 41 (36), 40 (22), 39 (11).

*Heptyl 2,2-dimethylbutanoate* (**16b**): MS (EI), *m*/*z* (%) 186 (7), 122 (34), 121 (42), 117 (52), 101 (6), 99 (17), 98 (10), 97 (10), 96 (7), 88 (14), 83 (5), 82 (7), 81 (12), 78 (7), 72 (6), 71 (100), 70 (32), 69 (13), 68 (7), 66 (13), 57 (28), 56 (17), 55 (29), 54 (9), 51 (9), 44 (5), 43 (61), 42 (16), 41 (40), 39 (14).

*1-Methylhexyl 3,3-dimethylbutanoate* (**11c**): MS (EI), *m*/*z* (%) 143 (6), 117 (15), 116 (10), 101 (21), 100 (7), 99 (87), 98 (26), 71 (10), 70 (10), 69 (10), 60 (8), 59 (13), 58 (5), 57 (100), 56 (22), 55 (20), 43 (25), 42 (9), 41 (32), 39 (9).

*2-Methylhexyl 3,3-dimethylbutanoate* (**12c**): MS (EI), *m*/*z* (%) 117 (7), 101 (9), 99 (65), 98 (26), 71 (8), 70 (16), 69 (10), 61 (9), 59 (8), 57 (100), 56 (41), 55 (17), 43 (28), 42 (8), 41 (34), 40 (12), 39 (9).

*3-Methylhexyl 3,3-dimethylbutanoate* (**13c**): MS (EI), *m*/*z* (%) 117 (10), 99 (36), 98 (43), 83 (8), 71 (12), 70 (46), 69 (41), 61 (8), 57 (100), 56 (37), 55 (39), 53 (6), 44 (15), 43 (37), 42 (11), 41 (54), 40 (27), 39 (15).

*4-Methylhexyl 3,3-dimethylbutanoate* (**14c**): MS (EI), *m*/*z* (%) 117 (15), 99 (29), 98 (32), 96 (6), 83 (11), 81 (9), 79 (8), 71 (8), 70 (60), 69 (53), 67 (9), 59 (6), 57 (100), 56 (36), 55 (52), 53 (9), 44 (31), 43 (25), 42 (12), 41 (77), 40 (69), 39 (27).

*5-Methylhexyl 3,3-dimethylbutanoate* (**15c**): MS (EI), *m*/*z* (%) 117 (21), 101 (7), 99 (38), 98 (28), 83 (15), 71 (8), 70 (30), 69 (16), 61 (7), 57 (100), 56 (41), 55 (21), 43 (28), 42 (5), 41 (30), 39 (7).

*Heptyl 3,3-dimethylbutanoate* (**16c**): MS (EI), *m*/*z* (%) 117 (40), 101 (22), 100 (5), 99 (51), 98 (35), 83 (9), 71 (11), 70 (30), 69 (16), 61 (27), 60 (6), 59 (14), 57 (100), 56 (30), 55 (29), 53 (5), 43 (35), 42 (12), 41 (59), 39 (15).

*1-Methylhexyl 2,3-dimethylbutanoate* (**11d**): MS (EI), *m*/*z* (%) 117 (9), 99 (22), 98 (16), 85 (16), 84 (10), 83 (6), 81 (6), 74 (16), 73 (9), 71 (46), 70 (21), 69 (32), 67 (5), 57 (64), 56 (45), 55 (48), 54 (9), 53 (7), 49 (5), 44 (37), 43 (66), 42 (15), 41 (62), 40 (100), 39 (19).

*2-Methylhexyl 2,3-dimethylbutanoate* (**12d**): MS (EI), *m*/*z* (%) 117 (5), 99 (19), 98 (11), 84 (6), 81 (5), 74 (7), 71 (23), 70 (9), 69 (8), 57 (24), 56 (23), 55 (17), 49 (8), 44 (42), 43 (27), 42 (6), 41 (27), 40 (100), 39 (9).

*3-Methylhexyl 2,3-dimethylbutanoate* (**13d**): MS (EI), *m*/*z* (%) 117 (8), 99 (11), 98 (28), 86 (5), 84 (8), 81 (8), 79 (5), 74 (5), 71 (24), 70 (23), 69 (25), 68 (6), 57 (26), 56 (24), 55 (27), 53 (6), 51 (5), 49 (7), 44 (50), 43 (33), 42 (9), 41 (32), 40 (100), 39 (11).

*4-Methylhexyl 2,3-dimethylbutanoate* (**14d**): MS (EI), *m*/*z* (%) 84 (5), 57 (6), 49 (9), 44 (50), 43 (7), 41 (6), 40 (100).

*5-Methylhexyl 2,3-dimethylbutanoate* (**15d**): MS (EI), *m*/*z* (%) 118 (5), 117 (72), 101 (8), 99 (35), 98 (28), 83 (17), 81 (6), 75 (11), 74 (32), 71 (66), 70 (46), 69 (28), 58 (5), 57 (100), 56 (78), 55 (49), 53 (6), 44 (11), 43 (96), 42 (10), 41 (59), 40 (21), 39 (17).

*Heptyl 2,3-dimethylbutanoate* (**16d**): MS (EI), *m*/*z* (%) 118 (6), 117 (100), 116 (5), 101 (14), 99 (42), 98 (35), 83 (7), 75 (20), 74 (78), 71 (74), 70 (37), 69 (25), 68 (7), 57 (76), 56 (60), 55 (22), 54 (7), 44 (16), 43 (96), 42 (22), 41 (75), 40 (31), 39 (21).

*1-Methylhexyl 2-methylpentanoate* (**11e epimer I**): MS (EI), *m*/*z* (%) 143 (7), 117 (33), 116 (5), 100 (6), 99 (90), 98 (28), 87 (7), 74 (48), 71 (72), 70 (16), 69 (16), 58 (5), 57 (100), 56 (32), 55 (29), 45 (7), 43 (69), 42 (13), 41 (43), 39 (11).

*1-Methylhexyl 2-methylpentanoate* (**11e epimer II**): MS (EI), *m*/*z* (%) 143 (10), 117 (41), 116 (6), 100 (7), 99 (100), 98 (30), 87 (8), 74 (44), 72 (5), 71 (74), 70 (15), 69 (14), 58 (5), 57 (93), 56 (27), 55 (26), 45 (6), 43 (64), 42 (13), 41 (41), 39 (12).

*2-Methylhexyl 2-methylpentanoate* (**12e**): MS (EI), *m*/*z* (%) 117 (31), 100 (6), 99 (91), 98 (44), 87 (8), 83 (6), 74 (41), 71 (92), 70 (38), 69 (31), 58 (5), 57 (86), 56 (100), 55 (35), 53 (5), 44 (5), 43 (89), 42 (17), 41 (62), 40 (5), 39 (17).

*3-Methylhexyl 2-methylpentanoate* (**13e**): MS (EI), *m*/*z* (%) 143 (8), 117 (48), 99 (45), 98 (93), 87 (5), 83 (16), 74 (16), 72 (5), 71 (82). 70 (87), 69 (85), 68 (5), 57 (97), 56 (57), 55 (53), 53 (5), 44 (5), 43 (100), 42 (16), 41 (58), 39 (14).

*4-Methylhexyl 2-methylpentanoate* (**14e**): MS (EI), *m*/*z* (%) 118 (6), 117 (80), 99 (33), 98 (48), 87 (5), 83 (8), 74 (14), 71 (62), 70 (97), 69 (54), 67 (6), 58 (5), 57 (100), 56 (45), 55 (44), 53 (7), 44 (8), 43 (76), 42 (20), 41 (76), 40 (11), 39 (19).

*5-Methylhexyl 2-methylpentanoate* (**15e**): MS (EI), *m*/*z* (%) 118 (7), 117 (96), 99 (43), 98 (36), 87 (8), 83 (20), 75 (9), 74 (29), 71 (71), 70 (51), 69 (33), 58 (5), 57 (100), 56 (84), 55 (44), 44 (5), 43 (98), 42 (13), 41 (54), 39 (13).

*Heptyl 2-methylpentanoate* (**16e**): MS (EI), *m*/*z* (%) 143 (7), 118 (6), 117 (100), 99 (31), 98 (28), 87 (11), 75 (12), 74 (38), 71 (48), 70 (24), 69 (16), 57 (38), 56 (26), 55 (29), 43 (65), 42 (15), 41 (52), 39 (14).

*1-Methylhexyl 3-methylpentanoate* (**11f**): MS (EI), *m*/*z* (%) 143 (7), 117 (23), 116 (14), 100 (7), 99 (100), 98 (28), 87 (10), 71 (30), 70 (13), 69 (15), 61 (7), 60 (18), 57 (40), 56 (29), 55 (21), 43 (34), 42 (11), 41 (33), 39 (10).

*2-Methylhexyl 3-methylpentanoate* (**12f**): MS (EI), *m*/*z* (%) 117 (18), 100 (7), 99 (100), 98 (38), 87 (7), 83 (5), 71 (37), 70 (30), 69 (21), 61 (12), 60 (12), 57 (73), 56 (72), 55 (26), 44 (6), 43 (61), 42 (16), 41 (51), 40 (11), 39 (13).

*3-Methylhexyl 3-methylpentanoate* (**13f**): MS (EI), *m*/*z* (%) 143 (5), 117 (35), 100 (5), 99 (63), 98 (80), 97 (5), 87 (6), 83 (17), 81 (7), 71 (47), 70 (93), 69 (100), 68 (7), 61 (12), 60 (7), 57 (91), 56 (65), 55 (71), 53 (9), 44 (16), 43 (89), 42 (25), 41 (90), 40 (27), 39 (27).

*4-Methylhexyl 3-methylpentanoate* (**14f**): MS (EI), *m*/*z* (%) 117 (35), 99 (32), 98 (51), 96 (6), 87 (5), 83 (13), 81 (8), 79 (7), 71 (26), 70 (87), 69 (79), 68 (7), 67 (12), 61 (6), 60 (5), 58 (5), 57 (74), 56 (42), 55 (62), 54 (6), 53 (12), 44 (26), 43 (41), 42 (24), 41 (100), 40 (50), 39 (29).

*5-Methylhexyl 3-methylpentanoate* (**15f**): MS (EI), *m*/*z* (%) 118 (5), 117 (69), 100 (5), 99 (69), 98 (46), 87 (11), 83 (28), 71 (39), 70 (63), 69 (41), 61 (11), 57 (100), 56 (87), 55 (43), 43 (69), 42 (13), 41 (54), 39 (12).

*Heptyl 3-methylpentanoate* (**16f**): MS (EI), *m*/*z* (%) 118 (7), 117 (100), 116 (8), 101 (5), 99 (71), 98 (46), 87 (21), 83 (11), 73 (8), 71 (42), 70 (46), 69 (30), 68 (8), 61 (27), 60 (12), 57 (62), 56 (41), 55 (40), 53 (6), 43 (61), 42 (24), 41 (75), 39 (20).

*1-Methylhexyl 4-methylpentanoate* (**11g**): MS (EI), *m*/*z* (%) 143 (8), 117 (22), 116 (10), 101 (10), 100 (7), 99 (100), 98 (33), 83 (6), 81 (30), 73 (11), 71 (13), 70 (17), 69 (21), 57 (59), 56 (39), 55 (32), 43 (52), 42 (10), 41 (39), 39 (10).

*2-Methylhexyl 4-methylpentanoate* (**12g**): MS (EI), *m*/*z* (%) 117 (23), 101 (10), 100 (7), 99 (100), 98 (43), 83 (9), 81 (39), 73 (10), 71 (18), 70 (39), 69 (24), 61 (6), 57 (76), 56 (89), 55 (32), 43 (76), 42 (12), 41 (49), 40 (5), 39 (12).

*3-Methylhexyl 4-methylpentanoate* (**13g**): MS (EI), *m*/*z* (%) 143 (7), 117 (32), 101 (11), 99 (46), 98 (73), 97 (7), 83 (22), 81 (33), 73 (7), 71 (23), 70 (95), 69 (96), 67 (6), 61 (5), 57 (82), 56 (65), 55 (76), 54 (5), 53 (7), 43 (100), 42 (20), 41 (77), 40 (38), 39 (21).

*4-Methylhexyl 4-methylpentanoate* (**14g**): MS (EI), *m*/*z* (%) 157 (6), 117 (55), 115 (5), 101 (8), 99 (36). 98 (40), 97 (6), 83 (12), 81 (26), 73 (6), 71 (16), 70 (100), 69 (53), 67 (5), 57 (76), 56 (41), 55 (43), 53 (6), 44 (5), 43 (53), 42 (15), 41 (61), 40 (7), 39 (15).

*5-Methylhexyl 4-methylpentanoate* (**15g**): MS (EI), *m*/*z* (%) 118 (5), 117 (80), 101 (18), 100 (5), 99 (66), 98 (42), 97 (7), 83 (39), 81 (42), 73 (12), 71 (19), 70 (70), 69 (47), 58 (5), 57 (97), 56 (100), 55 (60), 53 (5), 44 (5), 43 (90), 42 (12), 41 (62), 39 (14).

*Heptyl 4-methylpentanoate* (**16g**): MS (EI), *m*/*z* (%) 118 (7), 117 (100), 116 (8), 101 (19), 100 (5), 99 (57), 98 (43), 97 (7), 83 (17), 81 (40), 74 (5), 73 (17), 71 (15), 70 (43), 69 (28), 68 (7), 61 (10), 57 (45), 56 (40), 55 (41), 53 (5), 43 (67), 42 (15), 41 (64), 39 (16).

*1-Methylhexyl hexanoate* (**11h**): MS (EI), *m*/*z* (%) 143 (7), 117 (20), 116 (11), 100 (6), 99 (100), 98 (26), 73 (7), 71 (21), 70 (15), 69 (15), 60 (15), 57 (33), 56 (29), 55 (22), 43 (39), 42 (11), 41 (27), 39 (8).

*2-Methylhexyl hexanoate* (**12h**): MS (EI), *m*/*z* (%) 117 (22), 100 (7), 99 (100), 98 (37), 87 (6), 83 (5), 73 (6), 71 (27), 70 (33), 69 (20), 61 (6), 60 (9), 57 (55), 56 (76), 55 (26), 43 (68), 42 (15), 41 (44), 40 (5), 39 (10).

*3-Methylhexyl hexanoate* (**13h**): MS (EI), *m*/*z* (%) 143 (7), 117 (36), 99 (57), 98 (73), 97 (6), 83 (17), 81 (6), 73 (5), 71 (34), 70 (95), 69 (100), 68 (7), 61 (7), 57 (67), 56 (65), 55 (72), 53 (8), 44 (11), 43 (95), 42 (25), 41 (79), 40 (16), 39 (22).

*4-Methylhexyl hexanoate* (**14h**): MS (EI), *m*/*z* (%) 157 (5), 117 (51), 99 (38), 98 (37), 97 (5), 83 (11), 71 (22), 70 (100), 69 (68), 67 (8), 57 (63), 56 (38), 55 (47), 53 (7), 44 (11), 43 (44), 42 (21), 41 (68), 40 (20), 39 (21).

*5-Methylhexyl hexanoate* (**15h**): Yield 77%; IR (cm^−1^) 2956, 2932, 2870, 1737, 1467, 1385, 1367, 1245, 1170, 1099, 1009, 734; H NMR (400 MHz, CDCl_3_) 4.06 (triplet, *J* = 6.7 Hz, 2H, H-1′), 2.29 (triplet, *J* = 7.5 Hz, 2H, H-2), 1.69–1.57 (overlapping peaks, 4H, H-2′ and H-3), 1.55 (nonet, *J* = 6.6 Hz, 1H, H-5′), 1.39–1.24 (overlapping peaks, 6H, H-3′, H-4 and H-5), 1.23–1.15 (multiplet, 2H, H-4′), 0.90 (triplet, *J* = 6.9 Hz, 3H, H-6), 0.87 (doublet, *J* = 6.6 Hz, 6H, H-6′ and H-7′); ^13^C NMR (101 MHz, CDCl_3_) 174.03 (C-1), 64.40 (C-1′), 38.53 (C-4′), 34.39 (C-2), 31.34 (C-4), 28.89 (C-2′), 27.89 (C-5′), 24.73 (C-3), 23.73 (C-3′), 22.55 (C-6′ and C-7′), 22.34 (C-5), 13.92 (C-6); MS (EI), *m*/*z* (%) 117 (63), 99 (57), 98 (36), 87 (5), 83 (26), 73 (8), 71 (25), 70 (62), 69 (42), 68 (5), 60 (9), 57 (78), 56 (100), 55 (53), 44 (14), 43 (86), 42 (20), 41 (59), 40 (13), 39 (15).

*Heptyl hexanoate* (**16h**): MS (EI), *m*/*z* (%) 118 (6), 117 (100), 99 (65), 98 (41), 97 (5), 89 (6), 87 (11), 83 (8), 73 (12), 71 (29), 70 (44), 69 (29), 68 (7), 61 (13), 60 (8), 57 (39), 56 (39), 55 (40), 53 (5), 43 (63), 42 (22), 41 (63), 39 (17).

*6-Methylheptyl angelate* (**17o**): MS (EI), *m*/*z* (%) 101 (16), 100 (42), 95 (9), 91 (7), 84 (7), 83 (23), 82 (9), 81 (8), 79 (6), 71 (16), 70 (11), 69 (32), 68 (6), 67 (12), 57 (34), 56 (39), 55 (52), 54 (10), 53 (13), 51 (6), 49 (7), 44 (40), 43 (35), 42 (14), 41 (52), 40 (100), 39 (24).

*Octyl angelate* (**18o**): MS (EI), *m*/*z* (%) 101 (29), 100 (100), 85 (5), 83 (31), 82 (10), 71 (12), 70 (5), 57 (19), 56 (8), 55 (37), 43 (19), 42 (5), 41 (15), 39 (7).

*6-Methylheptyl tiglate* (**17p**): MS (EI), *m*/*z* (%) 102 (9), 101 (100), 100 (10), 97 (8), 84 (12), 83 (59), 82 (5), 71 (5), 70 (10), 69 (23), 57 (18), 56 (27), 55 (48), 53 (5), 43 (20), 42 (5), 41 (18), 39 (8).

*Octyl tiglate* (**18p**): MS (EI), *m*/*z* (%) 102 (6), 101 (100), 100 (12), 84 (9), 83 (57), 82 (6), 70 (11), 69 (9), 57 (9), 56 (12), 55 (45), 53 (6), 43 (12), 42 (5), 41 (16), 39 (8).

*6-Methylheptyl senecioate* (**17q**): MS (EI), *m*/*z* (%) 101 (60), 100 (69), 85 (6), 84 (9), 83 (100), 82 (12), 71 (20), 70 (6), 69 (17), 57 (33), 56 (20), 55 (40), 53 (8), 43 (35), 42 (8), 41 (29), 40 (5), 39 (14).

*Octyl senecioate* (**18q**): MS (EI), *m*/*z* (%) 212 (5), 101 (67), 100 (98), 84 (8), 83 (100), 82 (15), 71 (10), 70 (7), 69 (7), 57 (15), 56 (8), 55 (31), 53 (7), 43 (18), 42 (6), 41 (19), 39 (10).

*2-Phenylethyl 4-methylhexanoate* (**19i**): Yield 80%; RI = 1720 (DB-5MS column); IR (cm^−1^) 3030, 2960, 2875, 1735, 1605, 1498, 1455, 1381, 1364, 1237, 1169, 1104, 1031, 748, 698; UV (MeCN, 0.05 M) λ_max_ nm (log ε): 204 (4.50), 224 (4.20), 275 (3.67), 281 (3.69); ^1^H NMR (400 MHz, CDCl_3_) 7.34–7.27 (multiplet, 2H, H-5′ and H-7′), 7.25–7.20 (overlapping peaks, 3H, H-4′, H-6′, and H-8′), 4.28 (triplet, *J* = 7.1 Hz, 2H, H-1′), 2.94 (triplet, *J* = 7.1 Hz, 2H, H-2′), 2.37–2.20 (multiplet, 2H, H-2), 1.69–1.58 (multiplet, 1H, H-3a), 1.45–1.36 (multiplet, 1H, H-3b), 1.36–1.25 (overlapping peaks, 2H, H-4 and H-5a), 1.20–1.08 (multiplet, 1H, H-5b), 0.86 (triplet, *J* = 7.1 Hz, 3H, H-6), 0.85 (doublet, *J* = 6.4 Hz, 3H, H-7); ^13^C NMR (101 MHz, CDCl_3_) 174.09 (C-1), 137.90 (C-3′), 128.91 (C-4′ and C-8′), 128.48 (C-5′ and C-7′), 126.53 (C-6′), 64,74 (C-1′), 35.16 (C-2′), 33.97 (C-4), 32.14 (C-2), 31.49 (C-3), 29.13 (C-5), 18.79 (C-7), 11.28 (C-6); MS (EI), *m*/*z* (%) 105 (33), 104 (100), 103 (5), 95 (9), 83 (5), 77 (6), 55 (10), 43 (10).

*2-Phenylethyl 5-methylhexanoate* (**19j**): Yield 82%; RI = 1711 (DB-5MS column); IR (cm^−1^) 3030, 2955, 2871, 1735, 1605, 1498, 1455, 1386, 1367, 1311, 1246, 1168, 1107, 1056, 1016, 747, 698; MS (EI), *m/z* (%) 105 (31), 104 (100), 103 (5), 95 (11), 77 (6), 43 (11); UV (MeCN, 0.05 M) λ_max_ nm (log ε): 205 (4.55), 223 (4.22), 275 (3.71), 280 (3.71); ^1^H NMR (400 MHz, CDCl_3_) 7.33–7.27 (multiplet, 2H, H-5′ and H-7′), 7.25–7.19 (overlapping peaks, 3H, H-4′, H-6′, and H-8′), 4.29 (triplet, *J* = 7.1 Hz, 2H, H-1′), 2.93 (triplet, *J* = 7.1 Hz, 2H, H-2′), 2.26 (triplet, *J* = 7.6 Hz, 2H, H-2), 1.63–1.55 (multiplet, 2H, H-3), 1.53 (nonet, *J* = 6.6 Hz, 1H, H-5), 1.19–1.12 (multiplet, 2H, H-4), 0.87 (doublet, 6H, *J* = 6.6 Hz, H-6 and H-7); ^13^C NMR (101 MHz, CDCl_3_) 173.82 (C-1), 137.90 (C-3′), 128.92 (C-4′ and C-8′), 128.49 (C-5′ and C-7′), 126.54 (C-6′), 64.72 (C-1′), 38.35 (C-4), 35.17 (C-2′), 34.57 (C-2), 27.78 (C-5), 22.86 (C-3), 22.50 (C-6 and C-7); MS (EI), *m*/*z* (%) 105 (31), 104 (100), 103 (5), 95 (11), 77 (6), 43 (11).

*2-Phenylethyl heptanoate* (**19k**): RI = 1749 (DB-5MS column); MS (EI), *m*/*z* (%) 113 (5), 105 (27), 104 (100), 103 (7), 91 (9), 77 (7), 43 (10).

*2-Phenylethyl 6-methylheptanoate* (**19m**): RI = 1812 (DB-5MS column); MS (EI), *m*/*z* (%) 109 (6), 105 (32), 104 (100), 103 (5), 91 (6), 77 (5), 57 (6), 43 (7).

*2-Phenylethyl octanoate* (**19n**): RI = 1853 (DB-5MS column); MS (EI), *m*/*z* (%) 105 (27), 104 (100), 103 (6), 91 (7), 77 (6), 57 (11), 41 (7).

## 4. Conclusions

The creation of a small synthetic library consisting of a series of esters (a total of 159 chemical entities, among them 102 new compounds) enabled the easy, fast, and unambiguous identification of potentially olfactory-interesting esters that were undetectable through direct GC-MS analyses of the unfractionated *Pelargonium graveolens* essential oil (Figure S160). Nine esters (5-methylhexyl formate, (*Z*)-hex-3-en-1-yl 3-methylpentanoate, 3-methylbutyl 3-methylpentanoate, 3-methylpentyl 4-methylpentanoate, 5-methylhexyl hexanoate, 3-methylbutyl 6-methylheptanoate, 2-phenylethyl 6-methylheptanoate, 5-methylhexyl tiglate, and 6-methylheptyl tiglate) were identified as new natural products, with eight of them representing entirely new compounds. Additionally, several other identified esters exhibit a rather limited distribution within the Plant Kingdom. The synthesized esters were comprehensively characterized via mass spectrometry (MS) and gas chromatography (RI), while selected ones that were identified as new natural products underwent additional structural elucidation using IR and NMR (1D and 2D NMR) spectroscopy.

## Figures and Tables

**Table 1:** A list of synthesized esters of isomeric hexanoic acids and isomeric pentanols, hexanols, heptanols, and (*Z*)-hex-3-en-1-ol, and their retention indices on a DB-5MS column.

Compound ^a^	Hexanoic Acid Isomer ^b^						
	b	c	d	e	f	g	h
Pentan-2-ol (**1**)	1098	1117	1142	1151/1152 ^c^	1177	1183	1216
2-Methylbutan-1-ol (**2**)	1143	1147	1176	1188	1212	1218	1255
3-Methylbutan-1-ol (**3**)	1138	1143	1171	1185	1209	1215	1252
Pentan-1-ol (**4**)	1176	1182	1211	1224	1248	1253	1289
Hexan-2-ol (**5**)	1207	1209	1234	1244/1246 ^c^	1271	1273	1309
2-Methylpentan-1-ol (**6**)	1230	1234	1261	1275	1298	1303	1340
3-Methylpentan-1-ol (**7**)	1242	1248	1279	1289	1313	1319	1356
4-Methylpentan-1-ol (**8**)	1238	1244	1276	1284	1310	1315	1351
Hexan-1-ol (**9**)	1275	1280	1309	1321	1346	1351	1387
(*Z*)-Hex-3-en-1-ol (**10**)	1272	1275	1305	1318	1341	1348	1382
Heptan-2-ol (**11**)	1293	1303	1330	1337/1341 ^c^	1365	1368	1404
2-Methylhexan-1-ol (**12**)	1323	1328	1358	1370	1392	1398	1434
3-Methylhexan-1-ol (**13**)	1328	1335	1364	1376	1400	1406	1442
4-Methylhexan-1-ol (**14**)	1345	1349	1379	1390	1415	1421	1457
5-Methylhexan-1-ol (**15**)	1337	1343	1370	1383	1407	1413	1449
Heptan-1-ol (**16**)	1373	1379	1407	1419	1445	1450	1484

[^a^] Experimental retention indices determined relative to a homologous series of *n*-alkanes (C_10_–C_15_) on a non-polar DB-5MS column; The precision of the RI values was confirmed by triplicate analysis with differences of <1 RI unit for all samples noted. [^b^] Isomeric hexanoic acids: 2,2-dimethylbutanoic (**b**), 3,3-dimethylbutanoic (**c**), 2,3-dimethylbutanoic (**d**), 2-methylpentanoic (**e**), 3-methylpentanoic (**f**), 4-methylpentanoic (**g**), and hexanoic acid (**h**). [^c^] Retention indices of two epimers are reported; all other epimers co-eluted under the applied GC conditions on the DB-5MS column.

**Table 2:** Retention indices of synthesized esters of angelic, tiglic, and senecioic acids with isomeric heptanols and selected isomeric octanols on a DB-5MS column.

Compound ^a^	Angelic Acid (o)	Tiglic Acid (p)	Senecioic Acid (q)
Heptan-2-ol (**11**)	1299	1343	1337
2-Methylhexan-1-ol (**12**)	1337	1378	1369
3-Methylhexan-1-ol (**13**)	1342	1387	1378
4-Methylhexan-1-ol (**14**)	1358	1400	1393
5-Methylhexan-1-ol (**15**)	1349	1393	1384
Heptan-1-ol (**16**)	1385	1431	1422
6-Methylheptan-1-ol (**17**)	1449	1493	1484
Octan-1-ol (**18**)	1484	1531	1522

[^a^] Experimental retention indices determined relative to a homologous series of *n*-alkanes (C_10_–C_15_) on a non-polar DB-5MS column; The precision of the RI values was confirmed by triplicate analysis with differences of <1 RI unit for all samples noted.

## Data Availability

Data are contained within the article and Supplementary Materials.

## Supplementary Materials

The following supporting information can be downloaded at: https://www.mdpi.com/article/10.3390/molecules30244741/s1, Table S1: Composition of the studied ester-containing chromatographic fraction of P. graveolens essential oil; Table S2: Criteria and evidence for assigning new natural product status, supported by SciFinder database searches accessed on 26 October 2025; Table S3: ^1^H and ^13^C NMR assignments data of 2-phenylethyl 5-methylhexanoate, along with the observed HMBC correlations; Table S4: ^1^H and ^13^C NMR assignments data of (*Z*)-hex-3-en-1-yl 3-methylpentanoate, along with the observed HMBC correlations; Table S5: ^1^H and ^13^C NMR assignments data of 3-methylbutyl 3-methylpentanoate, along with the observed HMBC correlations; Table S6: ^1^H and ^13^C NMR assignments data of 3-methylpentyl 4-methylpentanoate, along with the observed HMBC correlations; Table S7: ^1^H and ^13^C NMR assignments data of 5-methylhexyl tiglate, along with the observed HMBC correlations; Table S8: ^1^H and ^13^C NMR assignments data of 5-methylhexyl hexanoate, along with the observed HMBC correlations; Table S9: ^1^H and ^13^C NMR assignments data of 2-phenylethyl 4-methylhexanoate, along with the observed HMBC correlations; Figure S1: Mass spectrum of 1-methylhexyl formate (**11a**); Figure S2: Mass spectrum of 2-methylhexyl formate (**12a**); Figure S3: Mass spectrum of 3-methylhexyl formate (**13a**); Figure S4: Mass spectrum of 4-methylhexyl formate (**14a**); Figure S5: Mass spectrum of 5-methylhexyl formate (**15a**); Figure S6: Mass spectrum of heptyl formate (**16a**); Figure S7: Mass spectrum of 1-methylbutyl 2,2-dimethylbutanoate (**1b**); Figure S8: Mass spectrum of 2-methylbutyl 2,2-dimethylbutanoate (**2b**); Figure S9: Mass spectrum of 3-methylbutyl 2,2-dimethylbutanoate (**3b**); Figure S10: Mass spectrum of pentyl 2,2-dimethylbutanoate (**4b**); Figure S11: Mass spectrum of 1-methylbutyl 3,3-dimethylbutanoate (**1c**); Figure S12: Mass spectrum of 2-methylbutyl 3,3-dimethylbutanoate (**2c**); Figure S13: Mass spectrum of 3-methylbutyl 3,3-dimethylbutanoate (**3c**); Figure S14: Mass spectrum of pentyl 3,3-dimethylbutanoate (**4c**); Figure S15: Mass spectrum of 1-methylbutyl 2,3-dimethylbutanoate (**1d**); Figure S16: Mass spectrum of 2-methylbutyl 2,3-dimethylbutanoate (**2d**); Figure S17: Mass spectrum of 3-methylbutyl 2,3-dimethylbutanoate (**3d**); Figure S18: Mass spectrum of pentyl 2,3-dimethylbutanoate (**4d**); Figure S19a: Mass spectrum of 1-methylbutyl 2-methylpentanoate (**1e** epimer I); Figure S19b: Mass spectrum of 1-methylbutyl 2-methylpentanoate (**1e** epimer II); Figure S20: Mass spectrum of 2-methylbutyl 2-methylpentanoate (**2e**); Figure S21: Mass spectrum of 3-methylbutyl 2-methylpentanoate (**3e**); Figure S22: Mass spectrum of pentyl 2-methylpentanoate (**4e**); Figure S23: Mass spectrum of 1-methylbutyl 3-methylpentanoate (**1f**); Figure S24: Mass spectrum of 2-methylbutyl 3-methylpentanoate (**2f**); Figure S25a: Mass spectrum of 3-methylbutyl 3-methylpentanoate (**3f**); Figure S25b: ^1^H NMR spectrum of 3-methylbutyl 3-methylpentanoate (**3f**) recorded in CDCl_3_; Figure S25c: ^13^C NMR spectrum of 3-methylbutyl 3-methylpentanoate (**3f**) recorded in CDCl_3_; Figure S26: Mass spectrum of pentyl 3-methylpentanoate (**4f**); Figure S27: Mass spectrum of 1-methylbutyl 4-methylpentanoate (**1g**); Figure S28: Mass spectrum of 2-methylbutyl 4-methylpentanoate (**2g**); Figure S29: Mass spectrum of 3-methylbutyl 4-methylpentanoate (**3g**); Figure S30: Mass spectrum of pentyl 4-methylpentanoate (**4g**); Figure S31: Mass spectrum of 1-methylbutyl hexanoate (**1h**); Figure S32: Mass spectrum of 2-methylbutyl hexanoate (**2h**); Figure S33: Mass spectrum of 3-methylbutyl hexanoate (**3h**); Figure S34: Mass spectrum of pentyl hexanoate (**4h**); Figure S35: Mass spectrum of 1-methylpentyl 2,2-dimethylbutanoate (**5b**); Figure S36: Mass spectrum of 2-methylpentyl 2,2-dimethylbutanoate (**6b**); Figure S37: Mass spectrum of 3-methylpentyl 2,2-dimethylbutanoate (**7b**); Figure S38: Mass spectrum of 4-methylpentyl 2,2-dimethylbutanoate (**8b**); Figure S39: Mass spectrum of hexyl 2,2-dimethylbutanoate (**9b**); Figure S40: Mass spectrum of 1-methylpentyl 3,3-dimethylbutanoate (**5c**); Figure S41: Mass spectrum of 2-methylpentyl 3,3-dimethylbutanoate (**6c**); Figure S42: Mass spectrum of 3-methylpentyl 3,3-dimethylbutanoate (**7c**); Figure S43: Mass spectrum of 4-methylpentyl 3,3-dimethylbutanoate (**8c**); Figure S44: Mass spectrum of hexyl 3,3-dimethylbutanoate (**9c**); Figure S45: Mass spectrum of 1-methylpentyl 2,3-dimethylbutanoate (**5d**); Figure S46: Mass spectrum of 2-methylpentyl 2,3-dimethylbutanoate (**6d**); Figure S47: Mass spectrum of 3-methylpentyl 2,3-dimethylbutanoate (**7d**); Figure S48: Mass spectrum of 4-methylpentyl 2,3-dimethylbutanoate (**8d**); Figure S49: Mass spectrum of hexyl 2,3-dimethylbutanoate (**9d**); Figure S50a: Mass spectrum of 1-methylpentyl 2-methylpentanoate (**5e** epimer I); Figure S50b: Mass spectrum of 1-methylpentyl 2-methylpentanoate (**5e** epimer II); Figure S51: Mass spectrum of 2-methylpentyl 2-methylpentanoate (**6e**); Figure S52: Mass spectrum of 3-methylpentyl 2-methylpentanoate (**7e**); Figure S53: Mass spectrum of 4-methylpentyl 2-methylpentanoate (**8e**); Figure S54: Mass spectrum of hexyl 2-methylpentanoate (**9e**); Figure S55: Mass spectrum of 1-methylpentyl 3-methylpentanoate (**5f**); Figure S56: Mass spectrum of 2-methylpentyl 3-methylpentanoate (**6f**); Figure S57: Mass spectrum of 3-methylpentyl 3-methylpentanoate (**7f**); Figure S58: Mass spectrum of 4-methylpentyl 3-methylpentanoate (**8f**); Figure S59: Mass spectrum of hexyl 3-methylpentanoate (**9f**); Figure S60: Mass spectrum of 1-methylpentyl 4-methylpentanoate (**5g**); Figure S61: Mass spectrum of 2-methylpentyl 4-methylpentanoate (**6g**); Figure S62a: Mass spectrum of 3-methylpentyl 4-methylpentanoate (**7g**); Figure S62b: ^1^H NMR spectrum of 3-methylpentyl 4-methylpentanoate (**7g**) recorded in CDCl_3_; Figure S62c: ^13^C NMR spectrum of 3-methylpentyl 4-methylpentanoate (**7g**) recorded in CDCl_3_; Figure S63: Mass spectrum of 4-methylpentyl 4-methylpentanoate (**8g**); Figure S64: Mass spectrum of hexyl 4-methylpentanoate (**9g**); Figure S65: Mass spectrum of 1-methylpentyl hexanoate (**5h**); Figure S66: Mass spectrum of 2-methylpentyl hexanoate (**6h**); Figure S67: Mass spectrum of 3-methylpentyl hexanoate (**7h**); Figure S68: Mass spectrum of 4-methylpentyl hexanoate (**8h**); Figure S69: Mass spectrum of hexyl hexanoate (**9h**); Figure S70: Mass spectrum of (*Z*)-Hex-3-en-1-yl 2,2-dimethylbutanoate (**10b**); Figure S71: Mass spectrum of (*Z*)-Hex-3-en-1-yl 3,3-dimethylbutanoate (**10c**); Figure S72: Mass spectrum of (*Z*)-Hex-3-en-1-yl 2,3-dimethylbutanoate (**10d**); Figure S73: Mass spectrum of (*Z*)-Hex-3-en-1-yl 2-methylpentanoate (**10e**); Figure S74a: Mass spectrum of (*Z*)-Hex-3-en-1-yl 3-methylpentanoate (**10f**); Figure S74b: ^1^H NMR spectrum of (*Z*)-Hex-3-en-1-yl 3-methylpentanoate (**10f**) recorded in CDCl_3_; Figure S74c: ^13^C NMR spectrum of (*Z*)-Hex-3-en-1-yl 3-methylpentanoate (**10f**) recorded in CDCl_3_; Figure S74d: DEPT 90 spectrum of (*Z*)-Hex-3-en-1-yl 3-methylpentanoate (**10f**) recorded in CDCl_3_; Figure S74e: DEPT 135 spectrum of (*Z*)-Hex-3-en-1-yl 3-methylpentanoate (**10f**) recorded in CDCl_3_; Figure S74f: HSQC spectrum of (*Z*)-Hex-3-en-1-yl 3-methylpentanoate (**10f**) recorded in CDCl_3_; Figure S74g: HMBC spectrum of (*Z*)-Hex-3-en-1-yl 3-methylpentanoate (**10f**) recorded in CDCl_3_; Figure S75: Mass spectrum of (*Z*)-Hex-3-en-1-yl 4-methylpentanoate (**10g**); Figure S76: Mass spectrum of (*Z*)-Hex-3-en-1-yl hexanoate (**10h**); Figure S77: Mass spectrum of 1-methylhexyl angelate (**11o**); Figure S78: Mass spectrum of 2-methylhexyl angelate (**12o**); Figure S79: Mass spectrum of 3-methylhexyl angelate (**13o**); Figure S80: Mass spectrum of 4-methylhexyl angelate (**14o**); Figure S81: Mass spectrum of 5-methylhexyl angelate (**15o**); Figure S82: Mass spectrum of heptyl angelate (**16o**); Figure S83: Mass spectrum of 1-methylhexyl tiglate (**11p**); Figure S84: Mass spectrum of 2-methylhexyl tiglate (**12p**); Figure S85: Mass spectrum of 3-methylhexyl tiglate (**13p**); Figure S86: Mass spectrum of 4-methylhexyl tiglate (**14p**); Figure S87a: Mass spectrum of 5-methylhexyl tiglate (**15p**); Figure S87b: ^1^H NMR spectrum of 5-methylhexyl tiglate (**15p**) recorded in CDCl_3_; Figure S87c: ^13^C NMR spectrum of 5-methylhexyl tiglate (**15p**) recorded in CDCl_3_; Figure S88: Mass spectrum of heptyl tiglate (16p); Figure S89: Mass spectrum of 1-methylhexyl senecioate (**11q**); Figure S90: Mass spectrum of 2-methylhexyl senecioate (**12q**); Figure S91: Mass spectrum of 3-methylhexyl senecioate (**13q**); Figure S92: Mass spectrum of 4-methylhexyl senecioate (**14q**); Figure S93: Mass spectrum of 5-methylhexyl senecioate (**15q**); Figure S94: Mass spectrum of heptyl senecioate (**16q**); Figure S95a: Mass spectrum of 1-methylbutyl 2-methylheptanoate (**1l** epimer I); Figure S95b: Mass spectrum of 1-methylbutyl 2-methylheptanoate (**1l** epimer II); Figure S96: Mass spectrum of 2-methylbutyl 2-methylheptanoate (**2l**); Figure S97: Mass spectrum of 3-methylbutyl 2-methylheptanoate (**3l**); Figure S98: Mass spectrum of pentyl 2-methylheptanoate (**4l**); Figure S99: Mass spectrum of 1-methylbutyl 6-methylheptanoate (**1m**); Figure S100: Mass spectrum of 2-methylbutyl 6-methylheptanoate (**2m**); Figure S101: Mass spectrum of 3-methylbutyl 6-methylheptanoate (**3m**); Figure S102: Mass spectrum of pentyl 6-methylheptanoate (**4m**); Figure S103: Mass spectrum of 1-methylbutyl octanoate (**1n**); Figure S104: Mass spectrum of 2-methylbutyl octanoate (**2n**); Figure S105: Mass spectrum of 3-methylbutyl octanoate (**3n**); Figure S106: Mass spectrum of pentyl octanoate (**4n**); Figure S107: Mass spectrum of 1-methylhexyl 2,2-dimethylbutanoate (**11b**); Figure S108: Mass spectrum of 2-methylhexyl 2,2-dimethylbutanoate (**12b**); Figure S109: Mass spectrum of 3-methylhexyl 2,2-dimethylbutanoate (**13b**); Figure S110: Mass spectrum of 4-methylhexyl 2,2-dimethylbutanoate (**14b**); Figure S111: Mass spectrum of 5-methylhexyl 2,2-dimethylbutanoate (**15b**); Figure S112: Mass spectrum of heptyl 2,2-dimethylbutanoate (**16b**); Figure S113: Mass spectrum of 1-methylhexyl 3,3-dimethylbutanoate (**11c**); Figure S114: Mass spectrum of 2-methylhexyl 3,3-dimethylbutanoate (**12c**); Figure S115: Mass spectrum of 3-methylhexyl 3,3-dimethylbutanoate (**13c**); Figure S116: Mass spectrum of 4-methylhexyl 3,3-dimethylbutanoate (**14c**); Figure S117: Mass spectrum of 5-methylhexyl 3,3-dimethylbutanoate (**15c**); Figure S118: Mass spectrum of heptyl 3,3-dimethylbutanoate (**16c**); Figure S119: Mass spectrum of 1-methylhexyl 2,3-dimethylbutanoate (**11d**); Figure S120: Mass spectrum of 2-methylhexyl 2,3-dimethylbutanoate (**12d**); Figure S121: Mass spectrum of 3-methylhexyl 2,3-dimethylbutanoate (**13d**); Figure S122: Mass spectrum of 4-methylhexyl 2,3-dimethylbutanoate (**14d**); Figure S123: Mass spectrum of 5-methylhexyl 2,3-dimethylbutanoate (**15d**); Figure S124: Mass spectrum of heptyl 2,3-dimethylbutanoate (**16d**); Figure S125a: Mass spectrum of 1-methylhexyl 2-methylpentanoate (**11e** epimer I); Figure S125b: Mass spectrum of 1-methylhexyl 2-methylpentanoate (**11e** epimer II); Figure S126: Mass spectrum of 2-methylhexyl 2-methylpentanoate (**12e**); Figure S127: Mass spectrum of 3-methylhexyl 2-methylpentanoate (**13e**); Figure S128: Mass spectrum of 4-methylhexyl 2-methylpentanoate (**14e**); Figure S129: Mass spectrum of 5-methylhexyl 2-methylpentanoate (**15e**); Figure S130: Mass spectrum of heptyl 2-methylpentanoate (**16e**); Figure S131: Mass spectrum of 1-methylhexyl 3-methylpentanoate (**11f**); Figure S132: Mass spectrum of 2-methylhexyl 3-methylpentanoate (**12f**); Figure S133: Mass spectrum of 3-methylhexyl 3-methylpentanoate (**13f**); Figure S134: Mass spectrum of 4-methylhexyl 3-methylpentanoate (**14f**); Figure S135: Mass spectrum of 5-methylhexyl 3-methylpentanoate (**15f**); Figure S136: Mass spectrum of heptyl 3-methylpentanoate (**16f**); Figure S137: Mass spectrum of 1-methylhexyl 4-methylpentanoate (**11g**); Figure S138: Mass spectrum of 2-methylhexyl 4-methylpentanoate (**12g**); Figure S139: Mass spectrum of 3-methylhexyl 4-methylpentanoate (**13g**); Figure S140: Mass spectrum of 4-methylhexyl 4-methylpentanoate (**14g**); Figure S141: Mass spectrum of 5-methylhexyl 4-methylpentanoate (**15g**); Figure S142: Mass spectrum of heptyl 4-methylpentanoate (**16g**); Figure S143: Mass spectrum of 1-methylhexyl hexanoate (**11h**); Figure S144: Mass spectrum of 2-methylhexyl hexanoate (**12h**); Figure S145: Mass spectrum of 3-methylhexyl hexanoate (**13h**); Figure S146: Mass spectrum of 4-methylhexyl hexanoate (**14h**); Figure S147a: Mass spectrum of 5-methylhexyl hexanoate (**15h**); Figure S147b: ^1^H NMR spectrum of 5-methylhexyl hexanoate (15h) recorded in CDCl_3_; Figure S147c: ^13^C NMR spectrum of 5-methylhexyl hexanoate (**15h**) recorded in CDCl_3_; Figure S148: Mass spectrum of heptyl hexanoate (**16h**); Figure S149: Mass spectrum of 6-methylheptyl angelate (**17o**); Figure S150: Mass spectrum of octyl angelate (**18o**); Figure S151: Mass spectrum of 6-methylheptyl tiglate (**17p**); Figure S152: Mass spectrum of octyl tiglate (**18p**); Figure S153: Mass spectrum of 6-methylheptyl senecioate (**17q**); Figure S154: Mass spectrum of octyl senecioate (**18q**); Figure S155a: Mass spectrum of 2-phenylethyl 4-methylhexanoate (**19i**); Figure S155b: ^1^H NMR spectrum of 2-phenylethyl 4-methylhexanoate (**19i**) recorded in CDCl_3_; Figure S155c: ^13^C NMR spectrum of 2-phenylethyl 4-methylhexanoate (**19i**) recorded in CDCl_3_; Figure S156a: Mass spectrum of 2-phenylethyl 5-methylhexanoate (**19j**); Figure S156b: ^1^H NMR spectrum of 2-phenylethyl 5-methylhexanoate (**19j**) recorded in CDCl_3_; Figure S156c: ^13^C NMR spectrum of 2-phenylethyl 5-methylhexanoate (**19j**) recorded in CDCl_3_; Figure S156d: DEPT 90 spectrum of 2-phenylethyl 5-methylhexanoate (**19j**) recorded in CDCl_3_; Figure S156e: DEPT 135 spectrum of 2-phenylethyl 5-methylhexanoate (**19j**) recorded in CDCl_3_; Figure S156f: HSQC spectrum of 2-phenylethyl 5-methylhexanoate (**19j**) recorded in CDCl_3_; Figure S156g: HMBC spectrum of 2-phenylethyl 5-methylhexanoate (**19j**) recorded in CDCl_3_; Figure S157: Mass spectrum of 2-phenylethyl heptanoate (**19k**); Figure S158: Mass spectrum of 2-phenylethyl 6-methylheptanoate (**19m**); Figure S159: Mass spectrum of 2-phenylethyl octanoate (**19n**); Figure S160: Schematic overview of the identification strategy: synthesis to final confirmation.
