# Supplementary material for: Synthesis of Small Libraries of Natural Products—Part III: Identification of New Esters from Pelargonium graveolens L’Her. (Geraniaceae) Essential Oil
Source: Molecules. 2025 Dec 11;30(24):4741. doi: 10.3390/molecules30244741 (PMC12736027; doi:10.3390/molecules30244741)
Supplement: Supplementary file 1 [file molecules-30-04741-s001.zip › molecules-4006200-supplementary.pdf]

Supplementary material

for

Synthesis of Small Libraries of Natural Products—Part III: Identification of New Esters from *Pelargonium graveolens* L'Her. (Geraniaceae) Essential Oil

Šejla F. Gusinac Avdović [1], Marko Z. Mladenović [1,2], Niko S. Radulović [1,*]

[1] Department of Chemistry, Faculty of Sciences and Mathematics, University of Niš, Višegradska 33, 18000 Niš, Serbia
[2] Department of Sciences and Mathematics, State University of Novi Pazar, Vuka Karadžića 9, 36300 Novi Pazar, Serbia
* Correspondence: nikoradulovic@yahoo.com; Tel.: +381-18-533-015; Fax: +381-18-533-014

Content:

Table S1. Composition of the studied ester-containing chromatographic fraction of *P. graveolens* essential oil
Table S2. Criteria and evidence for assigning new natural product status, supported by SciFinder database searches accessed on October 26, 2025
Table S3. ^{1}H and ^{13}C NMR assignments data of 2-phenylethyl 5-methylhexanoate, along with the observed HMBC correlations
Table S4. ^{1}H and ^{13}C NMR assignments data of (*Z*)-hex-3-en-1-yl 3-methylpentanoate, along with the observed HMBC correlations
Table S5. ^{1}H and ^{13}C NMR assignments data of 3-methylbutyl 3-methylpentanoate, along with the observed HMBC correlations
Table S6. ^{1}H and ^{13}C NMR assignments data of 3-methylpentyl 4-methylpentanoate, along with the observed HMBC correlations
Table S7. ^{1}H and ^{13}C NMR assignments data of 5-methylhexyl tiglate, along with the observed HMBC correlations
Table S8. ^{1}H and ^{13}C NMR assignments data of 5-methylhexyl hexanoate, along with the observed HMBC correlations
Table S9. ^{1}H and ^{13}C NMR assignments data of 2-phenylethyl 4-methylhexanoate, along with the observed HMBC correlations
Figure S1. Mass spectrum of 1-methylhexyl formate (**11a**)
Figure S2. Mass spectrum of 2-methylhexyl formate (**12a**)
Figure S3. Mass spectrum of 3-methylhexyl formate (**13a**)
Figure S4. Mass spectrum of 4-methylhexyl formate (**14a**)
Figure S5. Mass spectrum of 5-methylhexyl formate (**15a**)
Figure S6. Mass spectrum of heptyl formate (**16a**)

Figure S7. Mass spectrum of 1-methylbutyl 2,2-dimethylbutanoate (**1b**)
Figure S8. Mass spectrum of 2-methylbutyl 2,2-dimethylbutanoate (**2b**)
Figure S9. Mass spectrum of 3-methylbutyl 2,2-dimethylbutanoate (**3b**)
Figure S10. Mass spectrum of pentyl 2,2-dimethylbutanoate (**4b**)
Figure S11. Mass spectrum of 1-methylbutyl 3,3-dimethylbutanoate (**1c**)
Figure S12. Mass spectrum of 2-methylbutyl 3,3-dimethylbutanoate (**2c**)
Figure S13. Mass spectrum of 3-methylbutyl 3,3-dimethylbutanoate (**3c**)
Figure S14. Mass spectrum of pentyl 3,3-dimethylbutanoate (**4c**)
Figure S15. Mass spectrum of 1-methylbutyl 2,3-dimethylbutanoate (**1d**)
Figure S16. Mass spectrum of 2-methylbutyl 2,3-dimethylbutanoate (**2d**)
Figure S17. Mass spectrum of 3-methylbutyl 2,3-dimethylbutanoate (**3d**)
Figure S18. Mass spectrum of pentyl 2,3-dimethylbutanoate (**4d**)
Figure S19a. Mass spectrum of 1-methylbutyl 2-methylpentanoate (**1e epimer I**)
Figure S19b. Mass spectrum of 1-methylbutyl 2-methylpentanoate (**1e epimer II**)
Figure S20. Mass spectrum of 2-methylbutyl 2-methylpentanoate (**2e**)
Figure S21. Mass spectrum of 3-methylbutyl 2-methylpentanoate (**3e**)
Figure S22. Mass spectrum of pentyl 2-methylpentanoate (**4e**)
Figure S23. Mass spectrum of 1-methylbutyl 3-methylpentanoate (**1f**)
Figure S24. Mass spectrum of 2-methylbutyl 3-methylpentanoate (**2f**)
Figure S25a. Mass spectrum of 3-methylbutyl 3-methylpentanoate (**3f**)
Figure S25b. ^{1}H NMR spectrum of 3-methylbutyl 3-methylpentanoate (**3f**) recorded in $CDCl_3$
Figure S25c. ^{13}C NMR spectrum of 3-methylbutyl 3-methylpentanoate (**3f**) recorded in $CDCl_3$
Figure S26. Mass spectrum of pentyl 3-methylpentanoate (**4f**)
Figure S27. Mass spectrum of 1-methylbutyl 4-methylpentanoate (**1g**)
Figure S28. Mass spectrum of 2-methylbutyl 4-methylpentanoate (**2g**)
Figure S29. Mass spectrum of 3-methylbutyl 4-methylpentanoate (**3g**)
Figure S30. Mass spectrum of pentyl 4-methylpentanoate (**4g**)
Figure S31. Mass spectrum of 1-methylbutyl hexanoate (**1h**)
Figure S32. Mass spectrum of 2-methylbutyl hexanoate (**2h**)
Figure S33. Mass spectrum of 3-methylbutyl hexanoate (**3h**)
Figure S34. Mass spectrum of pentyl hexanoate (**4h**)
Figure S35. Mass spectrum of 1-methylpentyl 2,2-dimethylbutanoate (**5b**)
Figure S36. Mass spectrum of 2-methylpentyl 2,2-dimethylbutanoate (**6b**)
Figure S37. Mass spectrum of 3-methylpentyl 2,2-dimethylbutanoate (**7b**)
Figure S38. Mass spectrum of 4-methylpentyl 2,2-dimethylbutanoate (**8b**)
Figure S39. Mass spectrum of hexyl 2,2-dimethylbutanoate (**9b**)
Figure S40. Mass spectrum of 1-methylpentyl 3,3-dimethylbutanoate (**5c**)
Figure S41. Mass spectrum of 2-methylpentyl 3,3-dimethylbutanoate (**6c**)
Figure S42. Mass spectrum of 3-methylpentyl 3,3-dimethylbutanoate (**7c**)
Figure S43. Mass spectrum of 4-methylpentyl 3,3-dimethylbutanoate (**8c**)
Figure S44. Mass spectrum of hexyl 3,3-dimethylbutanoate (**9c**)
Figure S45. Mass spectrum of 1-methylpentyl 2,3-dimethylbutanoate (**5d**)
Figure S46. Mass spectrum of 2-methylpentyl 2,3-dimethylbutanoate (**6d**)

Figure S47. Mass spectrum of 3-methylpentyl 2,3-dimethylbutanoate (**7d**)
Figure S48. Mass spectrum of 4-methylpentyl 2,3-dimethylbutanoate (**8d**)
Figure S49. Mass spectrum of hexyl 2,3-dimethylbutanoate (**9d**)
Figure S50a. Mass spectrum of 1-methylpentyl 2-methylpentanoate (**5e epimer I**)
Figure S50b. Mass spectrum of 1-methylpentyl 2-methylpentanoate (**5e epimer II**)
Figure S51. Mass spectrum of 2-methylpentyl 2-methylpentanoate (**6e**)
Figure S52. Mass spectrum of 3-methylpentyl 2-methylpentanoate (**7e**)
Figure S53. Mass spectrum of 4-methylpentyl 2-methylpentanoate (**8e**)
Figure S54. Mass spectrum of hexyl 2-methylpentanoate (**9e**)
Figure S55. Mass spectrum of 1-methylpentyl 3-methylpentanoate (**5f**)
Figure S56. Mass spectrum of 2-methylpentyl 3-methylpentanoate (**6f**)
Figure S57. Mass spectrum of 3-methylpentyl 3-methylpentanoate (**7f**)
Figure S58. Mass spectrum of 4-methylpentyl 3-methylpentanoate (**8f**)
Figure S59. Mass spectrum of hexyl 3-methylpentanoate (**9f**)
Figure S60. Mass spectrum of 1-methylpentyl 4-methylpentanoate (**5g**)
Figure S61. Mass spectrum of 2-methylpentyl 4-methylpentanoate (**6g**)
Figure S62a. Mass spectrum of 3-methylpentyl 4-methylpentanoate (**7g**)
Figure S62b. ^{1}H NMR spectrum of 3-methylpentyl 4-methylpentanoate (**7g**) recorded in $CDCl_3$
Figure S62c. ^{13}C NMR spectrum of 3-methylpentyl 4-methylpentanoate (**7g**) recorded in $CDCl_3$
Figure S63. Mass spectrum of 4-methylpentyl 4-methylpentanoate (**8g**)
Figure S64. Mass spectrum of hexyl 4-methylpentanoate (**9g**)
Figure S65. Mass spectrum of 1-methylpentyl hexanoate (**5h**)
Figure S66. Mass spectrum of 2-methylpentyl hexanoate (**6h**)
Figure S67. Mass spectrum of 3-methylpentyl hexanoate (**7h**)
Figure S68. Mass spectrum of 4-methylpentyl hexanoate (**8h**)
Figure S69. Mass spectrum of hexyl hexanoate (**9h**)
Figure S70. Mass spectrum of (Z)-Hex-3-en-1-yl 2,2-dimethylbutanoate (**10b**)
Figure S71. Mass spectrum of (Z)-Hex-3-en-1-yl 3,3-dimethylbutanoate (**10c**)
Figure S72. Mass spectrum of (Z)-Hex-3-en-1-yl 2,3-dimethylbutanoate (**10d**)
Figure S73. Mass spectrum of (Z)-Hex-3-en-1-yl 2-methylpentanoate (**10e**)
Figure S74a. Mass spectrum of (Z)-Hex-3-en-1-yl 3-methylpentanoate (**10f**)
Figure S74b. ^{1}H NMR spectrum of (Z)-Hex-3-en-1-yl 3-methylpentanoate (**10f**) recorded in $CDCl_3$
Figure S74c. ^{13}C NMR spectrum of (Z)-Hex-3-en-1-yl 3-methylpentanoate (**10f**) recorded in $CDCl_3$
Figure S74d. DEPT 90 spectrum of (Z)-Hex-3-en-1-yl 3-methylpentanoate (**10f**) recorded in $CDCl_3$
Figure S74e. DEPT 135 spectrum of (Z)-Hex-3-en-1-yl 3-methylpentanoate (**10f**) recorded in $CDCl_3$
Figure S74f. HSQC spectrum of (Z)-Hex-3-en-1-yl 3-methylpentanoate (**10f**) recorded in $CDCl_3$
Figure S74g. HMBC spectrum of (Z)-Hex-3-en-1-yl 3-methylpentanoate (**10f**) recorded in $CDCl_3$
Figure S75. Mass spectrum of (Z)-Hex-3-en-1-yl 4-methylpentanoate (**10g**)
Figure S76. Mass spectrum of (Z)-Hex-3-en-1-yl hexanoate (**10h**)

Figure S77. Mass spectrum of 1-methylhexyl angelate (**11o**)
Figure S78. Mass spectrum of 2-methylhexyl angelate (**12o**)
Figure S79. Mass spectrum of 3-methylhexyl angelate (**13o**)
Figure S80. Mass spectrum of 4-methylhexyl angelate (**14o**)
Figure S81. Mass spectrum of 5-methylhexyl angelate (**15o**)
Figure S82. Mass spectrum of heptyl angelate (**16o**)
Figure S83. Mass spectrum of 1-methylhexyl tiglate (**11p**)
Figure S84. Mass spectrum of 2-methylhexyl tiglate (**12p**)
Figure S85. Mass spectrum of 3-methylhexyl tiglate (**13p**)
Figure S86. Mass spectrum of 4-methylhexyl tiglate (**14p**)
Figure S87a. Mass spectrum of 5-methylhexyl tiglate (**15p**)
Figure S87b. ^{1}H NMR spectrum of 5-methylhexyl tiglate (**15p**) recorded in $CDCl_3$
Figure S87c. ^{13}C NMR spectrum of 5-methylhexyl tiglate (**15p**) recorded in $CDCl_3$
Figure S88. Mass spectrum of heptyl tiglate (**16p**)
Figure S89. Mass spectrum of 1-methylhexyl senecioate (**11q**)
Figure S90. Mass spectrum of 2-methylhexyl senecioate (**12q**)
Figure S91. Mass spectrum of 3-methylhexyl senecioate (**13q**)
Figure S92. Mass spectrum of 4-methylhexyl senecioate (**14q**)
Figure S93. Mass spectrum of 5-methylhexyl senecioate (**15q**)
Figure S94. Mass spectrum of heptyl senecioate (**16q**)
Figure S95a. Mass spectrum of 1-methylbutyl 2-methylheptanoate (**1l epimer I**)
Figure S95b. Mass spectrum of 1-methylbutyl 2-methylheptanoate (**1l epimer II**)
Figure S96. Mass spectrum of 2-methylbutyl 2-methylheptanoate (**2l**)
Figure S97. Mass spectrum of 3-methylbutyl 2-methylheptanoate (**3l**)
Figure S98. Mass spectrum of pentyl 2-methylheptanoate (**4l**)
Figure S99. Mass spectrum of 1-methylbutyl 6-methylheptanoate (**1m**)
Figure S100. Mass spectrum of 2-methylbutyl 6-methylheptanoate (**2m**)
Figure S101. Mass spectrum of 3-methylbutyl 6-methylheptanoate (**3m**)
Figure S102. Mass spectrum of pentyl 6-methylheptanoate (**4m**)
Figure S103. Mass spectrum of 1-methylbutyl octanoate (**1n**)
Figure S104. Mass spectrum of 2-methylbutyl octanoate (**2n**)
Figure S105. Mass spectrum of 3-methylbutyl octanoate (**3n**)
Figure S106. Mass spectrum of pentyl octanoate (**4n**)
Figure S107. Mass spectrum of 1-methylhexyl 2,2-dimethylbutanoate (**11b**)
Figure S108. Mass spectrum of 2-methylhexyl 2,2-dimethylbutanoate (**12b**)
Figure S109. Mass spectrum of 3-methylhexyl 2,2-dimethylbutanoate (**13b**)
Figure S110. Mass spectrum of 4-methylhexyl 2,2-dimethylbutanoate (**14b**)
Figure S111. Mass spectrum of 5-methylhexyl 2,2-dimethylbutanoate (**15b**)
Figure S112. Mass spectrum of heptyl 2,2-dimethylbutanoate (**16b**)
Figure S113. Mass spectrum of 1-methylhexyl 3,3-dimethylbutanoate (**11c**)
Figure S114. Mass spectrum of 2-methylhexyl 3,3-dimethylbutanoate (**12c**)
Figure S115. Mass spectrum of 3-methylhexyl 3,3-dimethylbutanoate (**13c**)
Figure S116. Mass spectrum of 4-methylhexyl 3,3-dimethylbutanoate (**14c**)

Figure S117. Mass spectrum of 5-methylhexyl 3,3-dimethylbutanoate (**15c**)
Figure S118. Mass spectrum of heptyl 3,3-dimethylbutanoate (**16c**)
Figure S119. Mass spectrum of 1-methylhexyl 2,3-dimethylbutanoate (**11d**)
Figure S120. Mass spectrum of 2-methylhexyl 2,3-dimethylbutanoate (**12d**)
Figure S121. Mass spectrum of 3-methylhexyl 2,3-dimethylbutanoate (**13d**)
Figure S122. Mass spectrum of 4-methylhexyl 2,3-dimethylbutanoate (**14d**)
Figure S123. Mass spectrum of 5-methylhexyl 2,3-dimethylbutanoate (**15d**)
Figure S124. Mass spectrum of heptyl 2,3-dimethylbutanoate (**16d**)
Figure S125a. Mass spectrum of 1-methylhexyl 2-methylpentanoate (**11e epimer I**)
Figure S125b. Mass spectrum of 1-methylhexyl 2-methylpentanoate (**11e epimer II**)
Figure S126. Mass spectrum of 2-methylhexyl 2-methylpentanoate (**12e**)
Figure S127. Mass spectrum of 3-methylhexyl 2-methylpentanoate (**13e**)
Figure S128. Mass spectrum of 4-methylhexyl 2-methylpentanoate (**14e**)
Figure S129. Mass spectrum of 5-methylhexyl 2-methylpentanoate (**15e**)
Figure S130. Mass spectrum of heptyl 2-methylpentanoate (**16e**)
Figure S131. Mass spectrum of 1-methylhexyl 3-methylpentanoate (**11f**)
Figure S132. Mass spectrum of 2-methylhexyl 3-methylpentanoate (**12f**)
Figure S133. Mass spectrum of 3-methylhexyl 3-methylpentanoate (**13f**)
Figure S134. Mass spectrum of 4-methylhexyl 3-methylpentanoate (**14f**)
Figure S135. Mass spectrum of 5-methylhexyl 3-methylpentanoate (**15f**)
Figure S136. Mass spectrum of heptyl 3-methylpentanoate (**16f**)
Figure S137. Mass spectrum of 1-methylhexyl 4-methylpentanoate (**11g**)
Figure S138. Mass spectrum of 2-methylhexyl 4-methylpentanoate (**12g**)
Figure S139. Mass spectrum of 3-methylhexyl 4-methylpentanoate (**13g**)
Figure S140. Mass spectrum of 4-methylhexyl 4-methylpentanoate (**14g**)
Figure S141. Mass spectrum of 5-methylhexyl 4-methylpentanoate (**15g**)
Figure S142. Mass spectrum of heptyl 4-methylpentanoate (**16g**)
Figure S143. Mass spectrum of 1-methylhexyl hexanoate (**11h**)
Figure S144. Mass spectrum of 2-methylhexyl hexanoate (**12h**)
Figure S145. Mass spectrum of 3-methylhexyl hexanoate (**13h**)
Figure S146. Mass spectrum of 4-methylhexyl hexanoate (**14h**)
Figure S147a. Mass spectrum of 5-methylhexyl hexanoate (**15h**)
Figure S147b. ^{1}H NMR spectrum of 5-methylhexyl hexanoate (**15h**) recorded in $CDCl_3$
Figure S147c. ^{13}C NMR spectrum of 5-methylhexyl hexanoate (**15h**) recorded in $CDCl_3$
Figure S148. Mass spectrum of heptyl hexanoate (**16h**)
Figure S149. Mass spectrum of 6-methylheptyl angelate (**17o**)
Figure S150. Mass spectrum of octyl angelate (**18o**)
Figure S151. Mass spectrum of 6-methylheptyl tiglate (**17p**)
Figure S152. Mass spectrum of octyl tiglate (**18p**)
Figure S153. Mass spectrum of 6-methylheptyl senecioate (**17q**)
Figure S154. Mass spectrum of octyl senecioate (**18q**)
Figure S155a. Mass spectrum of 2-phenylethyl 4-methylhexanoate (**19i**)
Figure S155b. ^{1}H NMR spectrum of 2-phenylethyl 4-methylhexanoate (**19i**) recorded in $CDCl_3$

Figure S155c. ^{13}C NMR spectrum of 2-phenylethyl 4-methylhexanoate (**19i**) recorded in $CDCl_3$
Figure S156a. Mass spectrum of 2-phenylethyl 5-methylhexanoate (**19j**)
Figure S156b. ^{1}H NMR spectrum of 2-phenylethyl 5-methylhexanoate (**19j**) recorded in $CDCl_3$
Figure S156c. ^{13}C NMR spectrum of 2-phenylethyl 5-methylhexanoate (**19j**) recorded in $CDCl_3$
Figure S156d. DEPT 90 spectrum of 2-phenylethyl 5-methylhexanoate (**19j**) recorded in $CDCl_3$
Figure S156e. DEPT 135 spectrum of 2-phenylethyl 5-methylhexanoate (**19j**) recorded in $CDCl_3$
Figure S156f. HSQC spectrum of 2-phenylethyl 5-methylhexanoate (**19j**) recorded in $CDCl_3$
Figure S156g. HMBC spectrum of 2-phenylethyl 5-methylhexanoate (**19j**) recorded in $CDCl_3$
Figure S157. Mass spectrum of 2-phenylethyl heptanoate (**19k**)
Figure S158. Mass spectrum of 2-phenylethyl 6-methylheptanoate (**19m**)
Figure S159. Mass spectrum of 2-phenylethyl octanoate (**19n**)
Figure S160. Schematic overview of the identification strategy: synthesis to final confirmation

Table S1. Composition of the studied ester-containing chromatographic fraction of *P. graveolens* essential oil

RI[a]	Constituents	Percentage [%][b]	Method of identification[c]
875	3-Methylbutyl acetate	tr	MS, RI
902	3-Methylpentyl formate	tr	MS, RI
909	Butyl propanoate	tr	MS, RI
921	(*Z*)-Hex-3-en-1-yl formate	tr	MS, RI, CoI
928	Hexyl formate	tr	MS, RI, CoI
963	Benzaldehyde	tr	MS, RI, CoI
967	3-Methylbutyl propanoate	tr	MS, RI
975	Geranic oxide (*syn*. 2,6,6-trimethyl-2-vinyltetrahydropyran)	tr	MS, RI
994	5-Methylhexyl formate	tr	CoI, **NEW**
997	*trans*-Dehydroxylinalool oxide	tr	MS, RI
998	Dehydro-1,8-cineole	tr	MS, RI
1007	(*Z*)-Hex-3-en-1-yl acetate	tr	MS, RI, CoI
1011	*cis*-Dehydroxylinalool oxide	tr	MS, RI
1026	Heptyl formate	tr	MS, RI, CoI
1035	1,8-Cineole	tr	MS, RI
1052	(*E*)-*β*-Ocimene	0.1	MS, RI
1074	3-Methylpentyl propanoate	tr	MS, CoI
1083	Benzyl formate	tr	MS, RI
1115	*cis*-Rose oxide	0.3	MS, RI
1125	Methyl octanoate	tr	MS, RI
1130	*α*-Campholenal	tr	MS, RI
1131	*trans*-Rose oxide	0.1	MS, RI
1138	*allo*-Ocimene	tr	MS, RI
1158	Menthone	5.0	MS, RI
1162	Citronellal	tr	MS, RI, CoI
1171	Isomenthone	0.4	MS, RI
1179	Citronellyl methyl ether	tr	MS, RI
1182	2-Phenylethyl formate	tr	MS, RI
1186	(*E*)-Isocitral	0.1	MS, RI
1188	(*Z*)-Hex-3-en-1-yl butanoate	tr	MS, RI, CoI
1194	Hexyl butanoate	tr	MS, RI
1195	2-Methylpropyl tiglate	tr	MS, RI
1202	Methyl salicylate	tr	MS, RI
1203	Methyl chavicol	tr	MS, RI
1206	Heptyl propanoate	tr	MS, RI
1208	Decanal	tr	MS, RI
1209	3-Methylbutyl 3-methylpentanoate	tr	CoI, **NEW**
1222	*p*-Menth-1-en-9-al	tr	MS, RI
1223	Linalyl formate	0.2	MS, RI
1226	*β*-Cyclocitral	tr	MS, RI
1232	Thymyl methyl ether	0.2	MS, RI
1233	(*Z*)-Hex-3-en-1-yl 2-methylbutanoate	tr	MS, RI, CoI
1237	Carvacryl methyl ether	0.1	MS, RI
1238	(*Z*)-Hex-3-en-1-yl 3-methylbutanoate	tr	MS, RI, CoI
1244	Neomenthyl formate	tr	MS, RI, CoI

1246	Cumin aldehyde	tr	MS, RI
1266	Neoisomenthyl formate	0.4	MS, RI, CoI
1277	Citronellyl formate	19.7	MS, RI
1291	Neryl formate	1.2	MS, RI, CoI
1306	Heptyl butanoate	tr	MS, RI
1307	4-Methylpentyl tiglate	tr	MS, RI, CoI
1307	Geranyl formate	10.1	MS, RI, CoI
1330	Methyl geranate	0.1	MS, RI
1319	3-Methylpentyl 4-methylpentanoate	tr	CoI, **NEW**
1336	(*Z*)-Hex-3-en-1-yl tiglate	0.1	MS, RI, CoI
1340	Hexyl tiglate	tr	MS, RI
1341	2-Methylpropyl benzoate	tr	MS, RI
1341	(*Z*)-Hex-3-en-1-yl 3-methylpentanoate	tr	CoI, **NEW**
1354	3-Methylbutyl heptanoate	tr	MS, RI
1354	2-Methylpropyl octanoate	tr	MS, RI
1357	Citronellyl acetate	1.8	MS, RI, CoI
1363	2-Phenylethyl propanoate	tr	MS, RI
1364	Neomenthyl propanoate	tr	MS, RI, CoI
1369	Neryl acetate	0.1	MS, RI, CoI
1388	Geranyl acetate	1.8	MS, RI, CoI
1392	Octyl butanoate	tr	MS, RI
1392	Benzyl 3-methylbutanoate	tr	MS, RI
1393	5-Methylhexyl tiglate	tr	CoI, **NEW**
1399	β-Elemene	tr	MS, RI
1402	2-Phenylethyl 2-methylpropanoate	0.4	MS, RI
1403	Neoisomenthyl propanoate	tr	MS, RI, CoI
1410	Dodecan-2-one	tr	MS, RI
1410	3-Methylbutyl 6-methylheptanoate	tr	CoI, **NEW**
1419	Isomenthyl 2-methylpropanoate	tr	MS, RI, CoI
1426	Linalyl butanoate	tr	MS, RI
1431	Heptyl tiglate	0.1	MS, CoI
1433	Unidentified constituent	0.1	
1441	γ-Elemene	tr	MS, RI
1443	3-Methylbutyl benzoate	tr	MS, RI
1445	Citronellyl propanoate	2.7	MS, RI, CoI
1448	3-Methylbutyl octanoate	tr	MS, RI
1449	5-Methylhexyl hexanoate	tr	CoI, **NEW**
1451	2-Methylbutyl octanoate	tr	MS, RI
1456	Neryl propanoate	tr	MS, RI, CoI
1465	α-Humulene	0.3	MS, RI
1476	Geranyl propanoate	4.4	MS, RI, CoI
1493	Citronellyl 2-methylpropanoate	tr	MS, RI, CoI
1493	6-Methylheptyl tiglate	tr	CoI, **NEW**
1497	2-Phenylethyl 2-methylbutanoate	0.4	MS, RI, CoI
1503	2-Phenylethyl 3-methylbutanoate	0.1	MS, RI, CoI
1507	Benzyl tiglate	tr	MS, RI
1507	Bicyclogermacrene	0.4	MS, RI
1518	Geranyl 2-methylpropanoate	1.8	MS, RI, CoI
1527	(*Z*)-Oct-5-en-1-yl tiglate	tr	MS, RI
1530	Citronellyl butanoate	3.9	MS, RI, CoI

1540	Neryl butanoate	tr	MS, RI, CoI
1545	2-Phenylethyl angelate	0.4	MS, RI, CoI
1563	Geranyl butanoate	6.8	MS, RI, CoI
1574	Citronellyl 2-methylbutanoate	0.1	MS, RI, CoI
1583	Citronellyl 3-methylbutanoate	0.3	MS, RI, CoI
1583	(*Z*)-Hex-3-en-1-yl benzoate	tr	MS, RI
1584	Neoisomenthyl pentanoate	tr	MS, RI
1585	Hexyl benzoate	tr	MS, RI
1586	2-Phenylethyl senecioate	tr	MS, RI, CoI
1594	2-Phenylethyl tiglate	3.1	MS, RI, CoI
1605	Geranyl 2-methylbutanoate	1.0	MS, RI, CoI
1615	Geranyl 3-methylbutanoate	0.3	MS, RI, CoI
1618	Tetradecanal	tr	MS, RI
1623	Citronellyl angelate	tr	MS, CoI
1626	Citronellyl pentanoate	1.1	MS, RI, CoI
1636	Neryl pentanoate	tr	MS, RI, CoI
1646	2-Phenylethyl hexanoate	tr	MS, RI
1658	Geranyl angelate	0.4	MS, RI, CoI
1660	Geranyl pentanoate	1.4	MS, RI, CoI
1671	Citronellyl tiglate	2.1	MS, RI, CoI
1681	Unidentified constituent	0.1	
1687	Unidentified constituent	1.2	
1702	Geranyl senecioate	tr	MS, CoI
1711	Geranyl tiglate	7.2	MS, RI, CoI
1711	2-Phenylethyl 5-methylhexanoate	tr	MS, CoI
1720	Pentadecanal	tr	MS, RI
1721	Unidentified constituent	1.1	
1723	Citronellyl hexanoate	1.0	MS, RI, CoI
1732	Neryl hexanoate	tr	MS, RI, CoI
1746	Unidentified constituent	0.1	
1748	Mint sulfide	0.2	MS, RI
1749	2-Phenylethyl heptanoate	tr	MS, RI
1757	Geranyl hexanoate	1.0	MS, RI, CoI
1771	Benzyl benzoate	0.1	MS, RI
1778	Unidentified constituent	0.2	
1782	Unidentified constituent	0.1	MS, RI, CoI
1787	Octyl benzoate	0.1	MS, RI
1796	Unidentified constituent	0.1	
1812	2-Phenylethyl 6-methylheptanoate	tr	CoI, **NEW**
1821	Citronellyl heptanoate	0.6	MS, RI, CoI
1828	Unidentified constituent	0.1	
1831	Neryl heptanoate	tr	MS, RI, CoI
1844	Unidentified constituent	tr	
1848	Hexahydrofarnesyl acetone	tr	MS, RI
1853	2-Phenylethyl octanoate	tr	MS, RI
1856	Geranyl heptanoate	1.2	MS, RI, CoI
1860	Isomenthyl octanoate	tr	MS, RI, CoI
1862	2-Phenylethyl benzoate	tr	MS, RI
1880	Unidentified constituent	0.2	MS, CoI
1890	Unidentified constituent	tr	

1893	Unidentified constituent	0.1	
1918	Citronellyl octanoate	0.6	MS, RI, CoI
1933	Methyl hexadecanoate	tr	MS, RI
1935	Citronellyl benzoate	0.1	MS, RI
1953	Geranyl octanoate	0.6	MS, RI, CoI
1969	Geranyl benzoate	0.1	MS, RI
1980	Unidentified constituent	tr	
1986	Unidentified constituent	tr	
1990	Unidentified constituent	tr	
2017	Citronellyl nonanoate	tr	MS, RI, CoI
2017	Unidentified constituent	0.3	
2029	Unidentified constituent	tr	
2033	Unidentified constituent	tr	
2048	Citronellyl citronellate	0.4	MS, CoI
2052	Geranyl nonanoate	tr	MS, RI, CoI
2078	Unidentified constituent	0.2	
2095	Unidentified constituent	tr	
2152	Geranyl decanoate	tr	MS, RI, CoI
2183	Unidentified constituent	tr	
2215	Unidentified constituent	tr	
2317	Citronellyl dodecanoate	tr	MS, CoI
2330	Neryl dodecanoate	tr	MS, CoI
2415	Unidentified constituent	tr	
2424	Unidentified constituent	tr	
2450	Unidentified constituent	tr	
2517	Citronellyl tetradecanoate	0.1	MS, CoI
2548	Unidentified constituent	tr	
2552	Geranyl tetradecanoate	tr	MS, CoI
2717	Citronellyl hexadecanoate	0.1	MS, CoI
2730	Neryl hexadecanoate	0.1	MS, CoI
2751	Geranyl hexadecanoate	tr	MS, CoI
2897	Citronellyl oleate	tr	MS, CoI
2917	Citronellyl octadecanoate	tr	MS, CoI
2930	Neryl octadecanoate	tr	MS, CoI
2950	Geranyl octadecanoate	tr	MS, CoI
		90.7	

[a] Retention indices on a DB-5MS column calculated against a series of co-injected *n*-alkanes (C_8–C_{30}); [b] tr – constituent in trace (< 0.05%); [c] RI - constituelnt identified by retention index matching with literature data; MS – constituent identified by mass spectra comparison with those listed in Wiley 11, NIST17, MassFinder 2.3, and a homemade mass spectral library; CoI - constituent identity confirmed by GC co-injection of an authentic sample.

Table S2. Criteria and evidence for establishing new natural product status, supported by SciFinder database searches (accessed on october 26, 2025), with illustrations of key fragmentation pathways

Natural product name	Identification route	ΔRI to closest isomer(s) (value; isomer)	Key diagnostic ions (*m*/*z*)
5-methylhexyl formate	MS/RI, GC co-inj	32, heptyl formate	41, 56, 70
(*Z*)-hex-3-en-1-yl 3-methylpentanoate	MS/RI, GC co-inj, NMR	7, (*Z*)-hex-3-en-1-yl 4-methylpentanoate	71, 82, 99
3-methylbutyl 3-methylpentanoate	MS/RI, GC co-inj, NMR	6, 3-methylbutyl 4-methylpentanoate	71, 99
3-methylpentyl 4-methylpentanoate	MS/RI, GC co-inj, NMR	4, 4-methylpentyl 4-methylpentanoate	71, 85, 99, 101
5-methylhexyl hexanoate	MS/RI, GC co-inj, NMR	35, heptyl hexanoate	71, 99
3-methylbutyl 6-methylheptanoate	MS/RI, GC co-inj	38, 3-methylbutyl octanoate	71, 127
2-phenylethyl 6-methylheptanoate	MS/RI, GC co-inj	41, 2-phenylethyl octanoate	104, 127
5-methylhexyl tiglate	MS/RI, GC co-inj, NMR	38, heptyl tiglate	55, 83, 99, 101
6-methylheptyl tiglate	MS/RI, GC co-inj	38, octyl tiglate	55, 83, 101, 113

Structures with annotated key fragment ions from mass spectra:

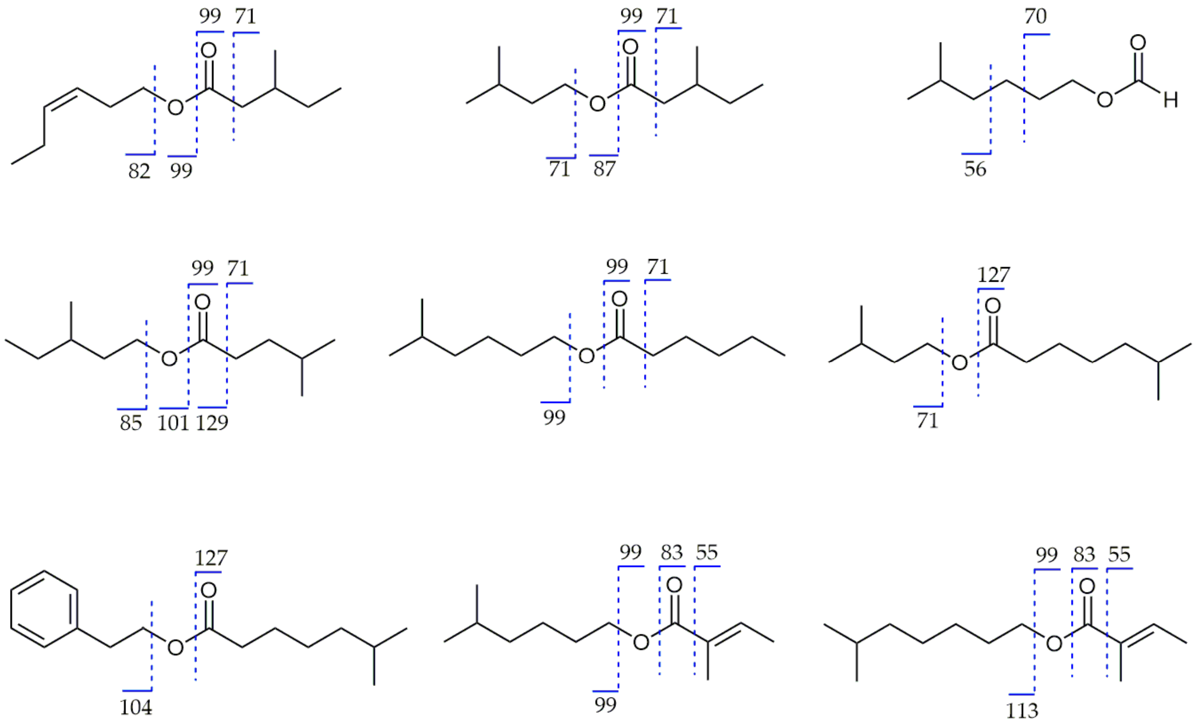

Table S3. ^{1}H and ^{13}C NMR assignments data of 2-phenylethyl 5-methylhexanoate, along with the observed HMBC correlations

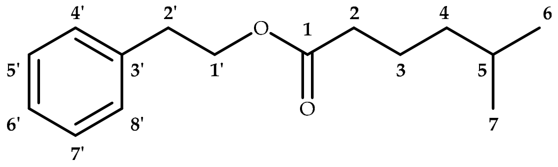

Position	δ_H (m, *J* (Hz), Integration)	δ_C	HMBC
1	/	173.82	/
2	2.26 (triplet, *J* = 7.6 Hz, 2H)	34.57	1, 3, 4
3	1.63 – 1.55 (multiplet, 2H)	22.86	1, 2, 4, 5
4	1.19 – 1.12 (multiplet, 2H)	38.35	2, 5, 6, 7
5	1.53 (nonet, *J* = 6.6 Hz, 1H)	27.78	4, 6, 7
6 and 7	0.87 (doublet, *J* = 6.6 Hz, 6H)	22.50	4, 5
1′	4.29 (triplet, *J* = 7.1 Hz, 2H)	64.72	1, 2′, 3′
2′	2.93 (triplet, *J* = 7.1 Hz, 2H)	35.17	1′, 3′, 4′, 8′
3′	/	137.90	/
4′,6′ and 8′	7.25 – 7.19 (overlapping peaks, 3H)	128.92 (4′ and 8′), 126.54 (6′)	2′, 6′ (4′ and 8′), 4′, 8′ (6′)
5′ and 7′	7.33 – 7.27 (multiplet, 2H)	128.49	3′

Table S4. ¹H and ¹³C NMR assignments data of (*Z*)-hex-3-en-1-yl 3-methylpentanoate, along with the observed HMBC correlations

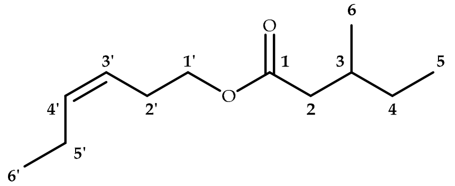

Position	δH (m, *J* (Hz), Integration)	δC	HMBC
1	/	173.42	/
2a	2.30 (doublet of doublets, *J* = 14.7, 6.1 Hz, 1H)	41.52	1, 3, 4, 6
2b	2.10 (doublet of doublets, *J* = 14.7, 8.2 Hz, 1H)	41.52	1, 3, 4, 6
3	1.89 (doublet of doublets of quartets of doublets of doublets, *J* = 8.2, 7.4, 6.7, 6.1, 5.7 Hz, 1H)	31.92	1, 2, 4, 5, 6
4a	1.36 (doublet of quartets of doublets, *J* = 13.4, 7.4, 5.7 Hz, 1H)	29.33	2, 3, 5, 6
4b	1.22 (pseudo doublet of quintets, *J* = 13.4, 7.4 Hz, 1H)	29.33	2, 3, 5, 6
5	0.89 (pseudo triplet, *J* = 7.4 Hz, 3H)	11.28	3, 4
6	0.93 (doublet, *J* = 6.7 Hz, 3H)	19.27	2, 3, 4
1′	4.07 (triplet, *J* = 6.9 Hz, 2H)	63.70	1, 2′, 3′
2′	2.38 (pseudo quartet of doublets of triplets, *J* = 7.0, 1.4, 0.7 Hz, 2H)	26.79	1′, 3′, 4′
3′	5.36 – 5.28 (multiplet, 1H)	123.81	1′, 2′, 4′, 5′
4′	5.54 – 5.46 (multiplet, 1H)	134.47	2′, 3′, 5′, 6′
5′	2.10 – 2.02 (multiplet, 1H)	20.61	3′, 4′, 6′
6′	0.97 (pseudo triplet, *J* = 7.5 Hz, 3H)	14.23	4′, 5′

Table S5. ^{1}H and ^{13}C NMR assignments data of 3-methylbutyl 3-methylpentanoate, along with the observed HMBC correlations

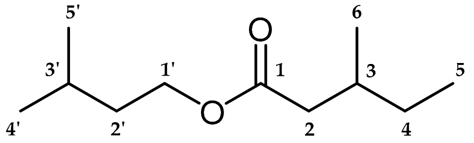

Position	δH (m, *J* (Hz), Integration)	δC	HMBC
1	/	173.53	/
2a	2.31 (doublet of doublets, *J* = 14.6, 8.1 Hz, 1H)	41.61	1, 3, 4, 6
2b	2.09 (doublet of doublets, *J* = 14.6, 8.1 Hz, 1H)	41.61	1, 3, 4, 6
3	1.94 – 1.82 (multiplet, 1H)	31.97	1, 2, 4, 5, 6
4a	1.47 – 1.31 (multiplet, 1H)	29.34	2, 3, 5, 6
4b	1.29 – 1.13 (multiplet, 1H)	29.34	2, 3, 5, 6
5	0.88 (triplet, *J* = 7.4 Hz, 3H)	11.28	3, 4
6	0.93 (doublet, *J* = 6.7 Hz, 3H)	19.28	2, 3, 4
1′	4.10 (triplet, *J* = 6.9 Hz, 2H)	62.82	1, 2′, 3′
2′	1.52 (pseudo quartet, *J* = 6.9 Hz, 2H)	37.39	1′, 3′, 4′, 5′
3′	1.68 (nonet, *J* = 6.9 Hz, 1H)	25.08	1′, 2′, 4′, 5′
4′ and 5′	0.92 (doublet, *J* = 6.9 Hz, 6H)	22.46	2′, 3′

Table S6. ^{1}H and ^{13}C NMR assignments data of 3-methylpentyl 4-methylpentanoate, along with the observed HMBC correlations

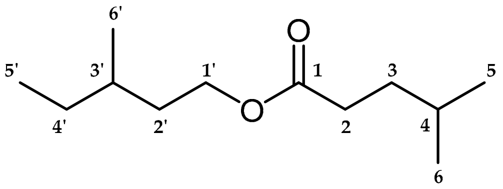

Position	δH (m, *J* (Hz), Integration)	δC	HMBC
1	/	174.19	/
2	2.32 – 2.27 (multiplet, 2H)	32.48	1, 3, 4
3 and 4	1.61 – 1.48 (overlapping peaks, 3H)	33.82 (3), 27.68 (4)	1, 2, 4, 5, 6 (3), 2, 3, 5, 6 (4)
5 and 6	0.90 (doublet, *J* = 6.4 Hz, 6H)	22.23	3, 4
1′	4.16 – 4.03 (multiplet, 2H)	62.87	1, 2′, 3′
2′a	1.72 – 1.61 (multiplet, 1H)	35.13	1′, 3′, 4′, 6′
2′b and 3′	1.48 – 1.41 (overlapping peaks, 2H)	35.13 (2′b), 31.41 (3′)	1′, 3′, 4′, 6′ (2′b), 1′, 4′, 5′, 6′ (3′)
4′a	1.41 – 1.31 (multiplet, 1H)	29.38	2′, 3′, 5′, 6′
4′b	1.19 (pseudo doublet of quintets, *J* = 13.5, 7.3 Hz, 1H)	29.38	2′, 3′, 5′, 6′
5′	0.88 (triplet, *J* = 7.4 Hz, 3H)	11.22	3′, 4′
6′	0.90 (doublet, *J* = 6.4 Hz, 3H)	19.02	2′, 4′

Table S7. ^{1}H and ^{13}C NMR assignments data of 5-methylhexyl tiglate, along with the observed HMBC correlations

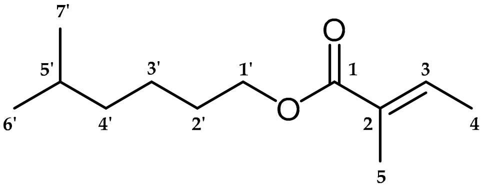

Position	δH (m, *J* (Hz), Integration)	δC	HMBC
1	/	168.25	/
2	/	128.79	/
3	6.85 (quartet of quartets, *J* = 7.0, 1.4 Hz, 1H)	136.81	1, 2, 4, 5
4	1.79 (doublet of quartets, *J* = 7.0, 1.4 Hz, 3H)	14.32	2, 3
5	1.83 (pseudo quintet, *J* = 1.4 Hz, 3H)	12.03	1, 2, 3
1′	4.12 (triplet, *J* = 6.7 Hz, 2H)	64.57	1, 2′, 3′
2′	1.65 (pseudo quintet, *J* = 6.7 Hz, 2H)	28.92	1′, 3′, 4′
3′	1.41 – 1.33 (multiplet, 2H)	23.80	1′, 2′, 4′, 5′
4′	1.27 – 1.17 (multiplet, 2H)	38.54	2′, 3′, 5′, 6′, 7′
5′	1.54 (nonet, *J* = 6.6 Hz, 1H)	27.86	4′, 6′, 7′
6′ and 7′	0.88 (doublet, *J* = 6.6 Hz, 6H)	22.55	4′, 5′

Table S8. ^{1}H and ^{13}C NMR assignments data of 5-methylhexyl hexanoate, along with the observed HMBC correlations

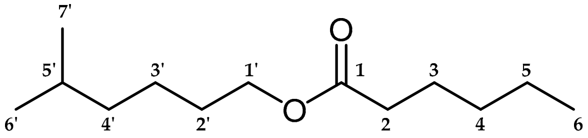

Position	δH (m, *J* (Hz), Integration)	δC	HMBC
1	/	174.03	/
2	2.29 (triplet, *J* = 7.5 Hz, 2H)	34.39	1, 3, 4
3 and 2′	1.69 – 1.57 (overlapping peaks, 4H)	24.73 (3), 28.89 (2′)	1, 2, 4 (3), 1′, 3′, 4′ (2′)
4, 5 an 3′	1.39 – 1.24 (overlapping peaks, 6H)	31.34 (4), 22.34 (5) and 23.73 (3′)	5 (4), 4 (5), 1′, 4′ (3′)
6	0.90 (triplet, *J* = 6.9 Hz, 3H)	13.92	4, 5
1′	4.06 (triplet, *J* = 6.7 Hz, 2H)	64.40	1, 2′, 3′
4′	1.23 – 1.15 (multiplet, 2H)	38.53	5′, 6′, 7′
5′	1.55 (nonet, *J* = 6.6 Hz, 1H)	27.89	4′, 6′, 7′
6′ and 7′	0.87 (doublet, *J* = 6.6 Hz, 6H)	22.55	4′, 5′

Table S9. ^{1}H and ^{13}C NMR assignments data of 2-phenylethyl 4-methylhexanoate, along with the observed HMBC correlations

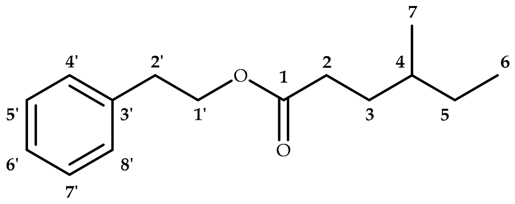

Position	δ_H (m, J (Hz), Integration)	δ_C	HMBC
1	/	174.09	/
2	2.37 – 2.20 (multiplet, 2H)	32.14	1, 3, 4
3a	1.69 – 1.58 (multiplet, 1H)	31.49	1, 4, 5, 7
3b	1.45 – 1.36 (multiplet, 1H)	31.49	1, 4, 5, 7
4 and 5a	1.36 – 1.25 (overlapping peaks, 2H)	33.97 (4), 29.13 (5a)	5, 6, 7 (4), 4, 6, 7 (5a)
5b	1.20 - 1.08 (multiplet, 1H)	29.13	3, 4, 6, 7
6	0.86 (triplet, J = 7.1 Hz, 3H)	11.28	4, 5
7	0.85 (doublet, J = 6.4 Hz, 3H)	18.79	3, 4, 5
1′	4.28 (triplet, J = 7.1 Hz, 2H)	64.74	1, 2′, 3′
2′	2.94 (triplet, J = 7.1 Hz, 2H)	35.16	1′, 3′, 4′, 8′
3′	/	137.90	/
4′, 6′ and 8′	7.25 – 7.20 (overlapping peaks, 3H)	128.91 (4′ and 8′), 126.53 (6′)	2′, 6′ (4′ and 8′), 4′, 8′ (6′)
5′ and 7′	7.34 – 7.27 (multiplet, 2H)	128.48	3′

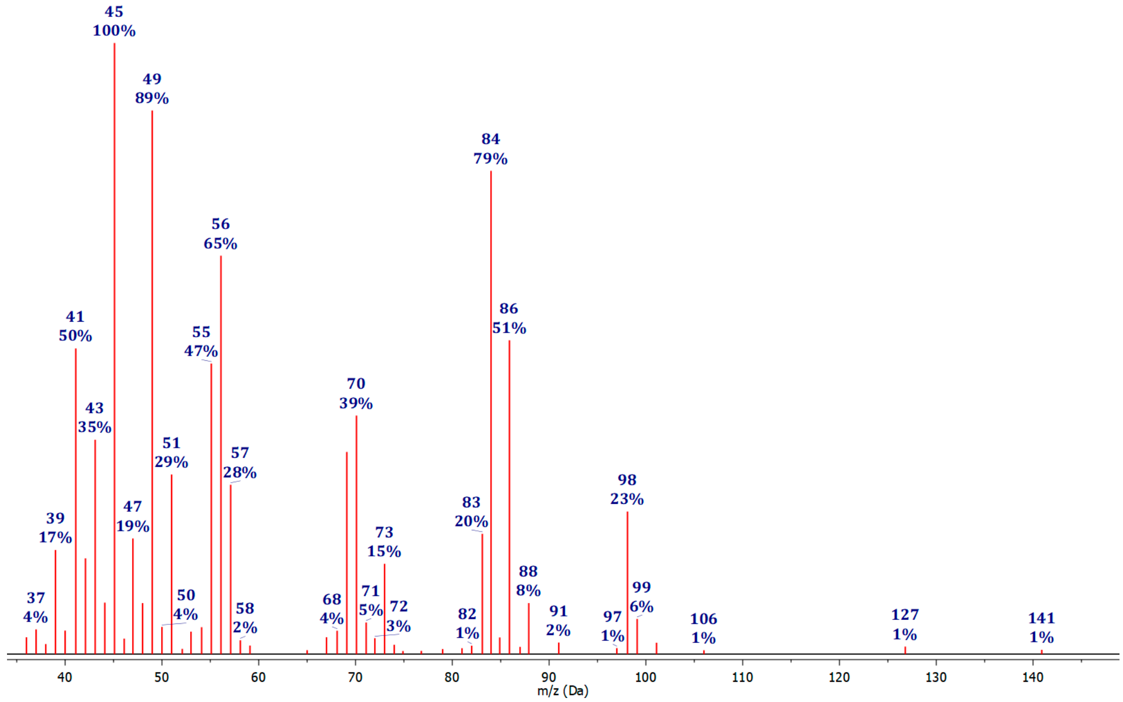

Figure S1. Mass spectrum of 1-methylhexyl formate (**11a**)

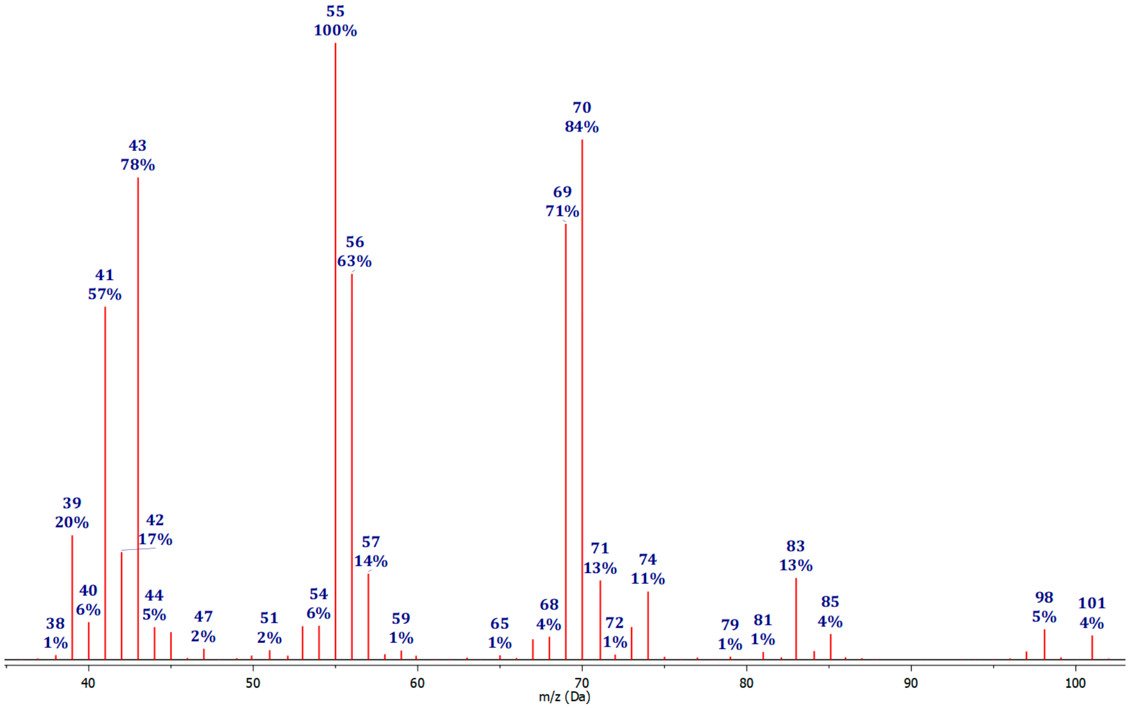

Figure S2. Mass spectrum of 2-methylhexyl formate (**12a**)

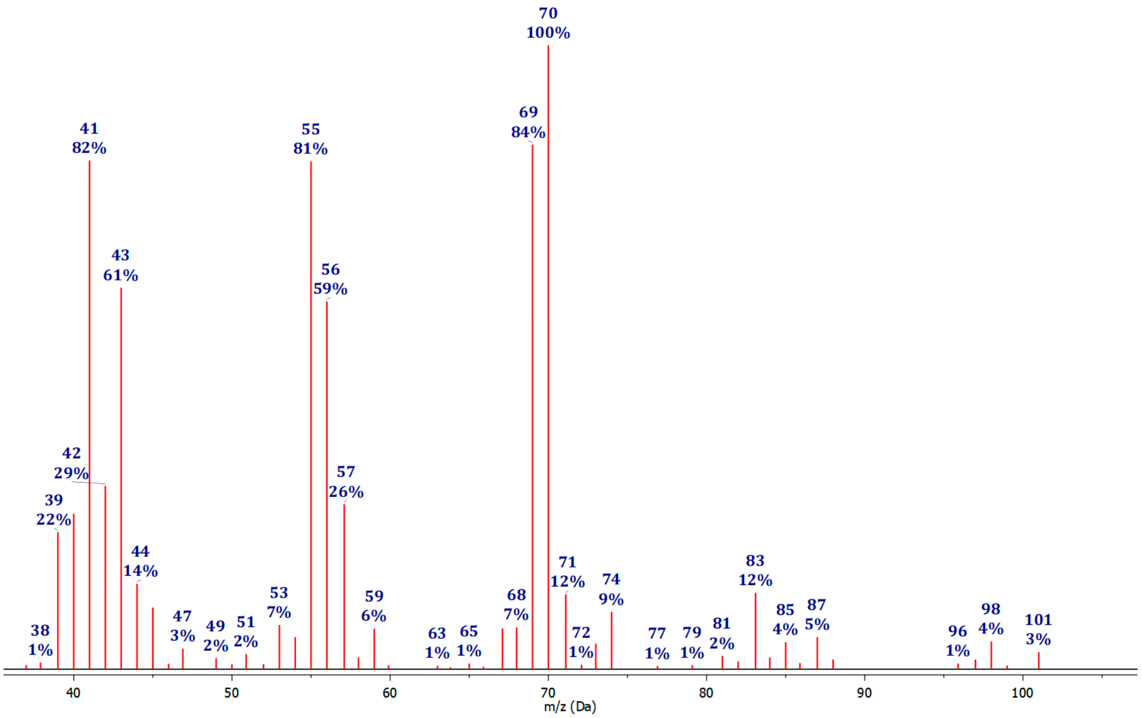

Figure S3. Mass spectrum of 3-methylhexyl formate (**13a**)

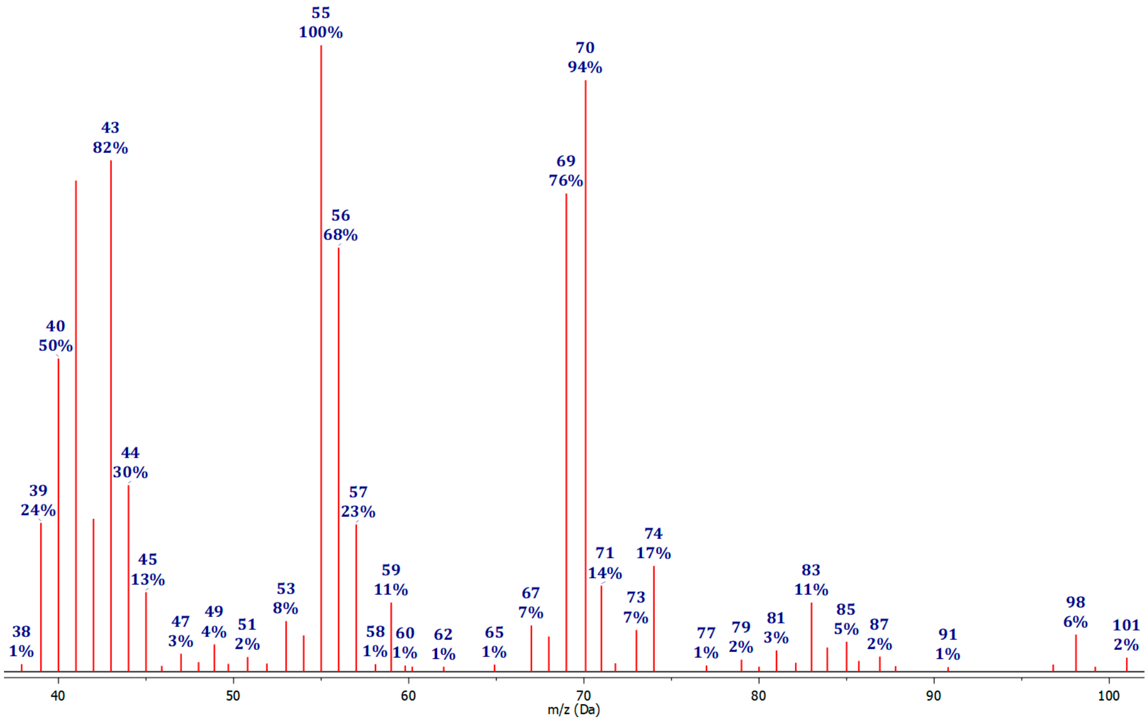

Figure S4. Mass spectrum of 4-methylhexyl formate (**14a**)

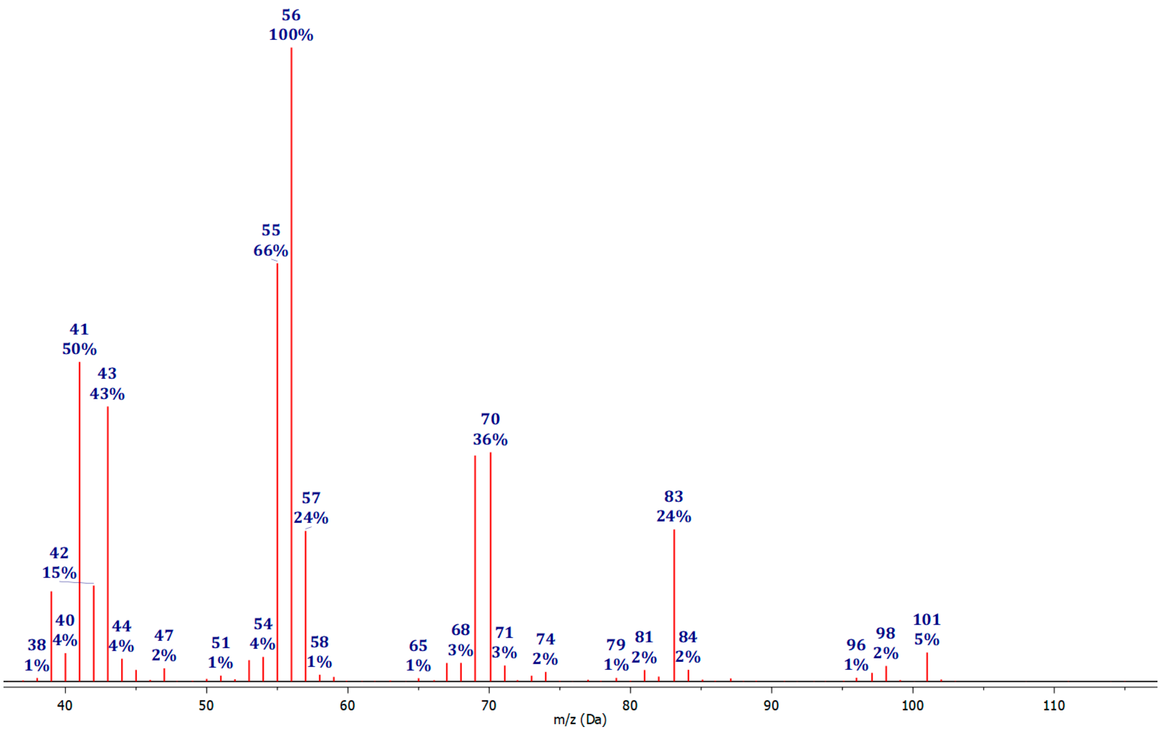

Figure S5. Mass spectrum of 5-methylhexyl formate (**15a**)

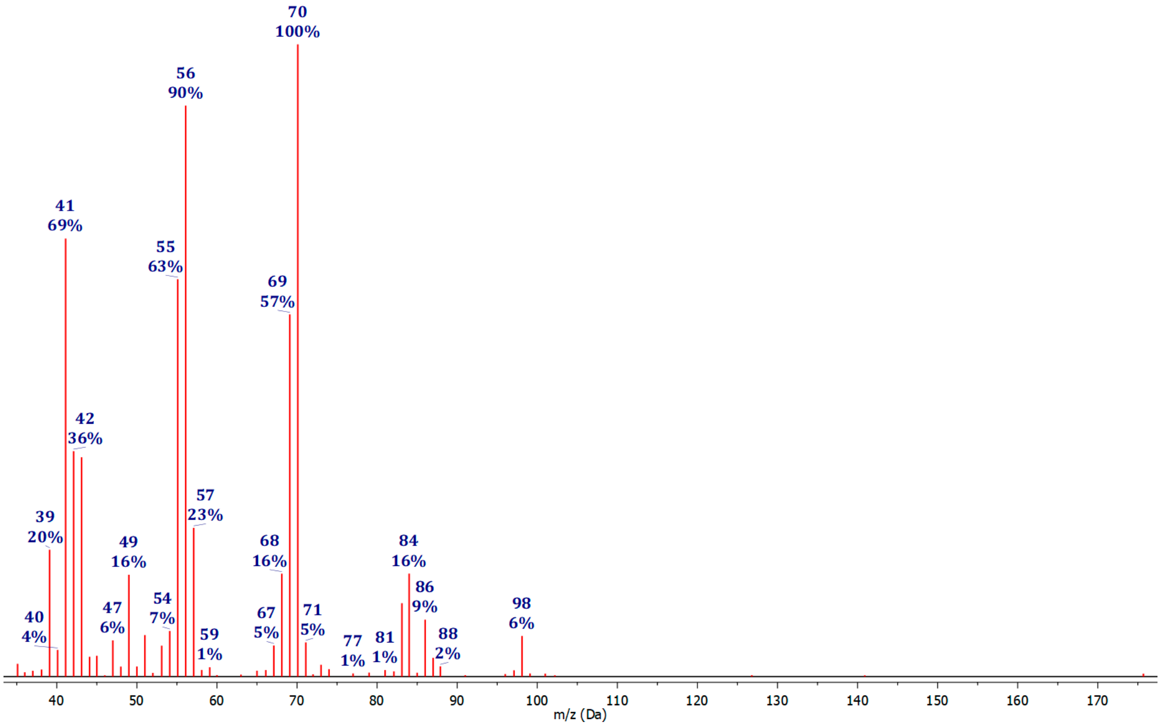

Figure S6. Mass spectrum of heptyl formate (**16a**)

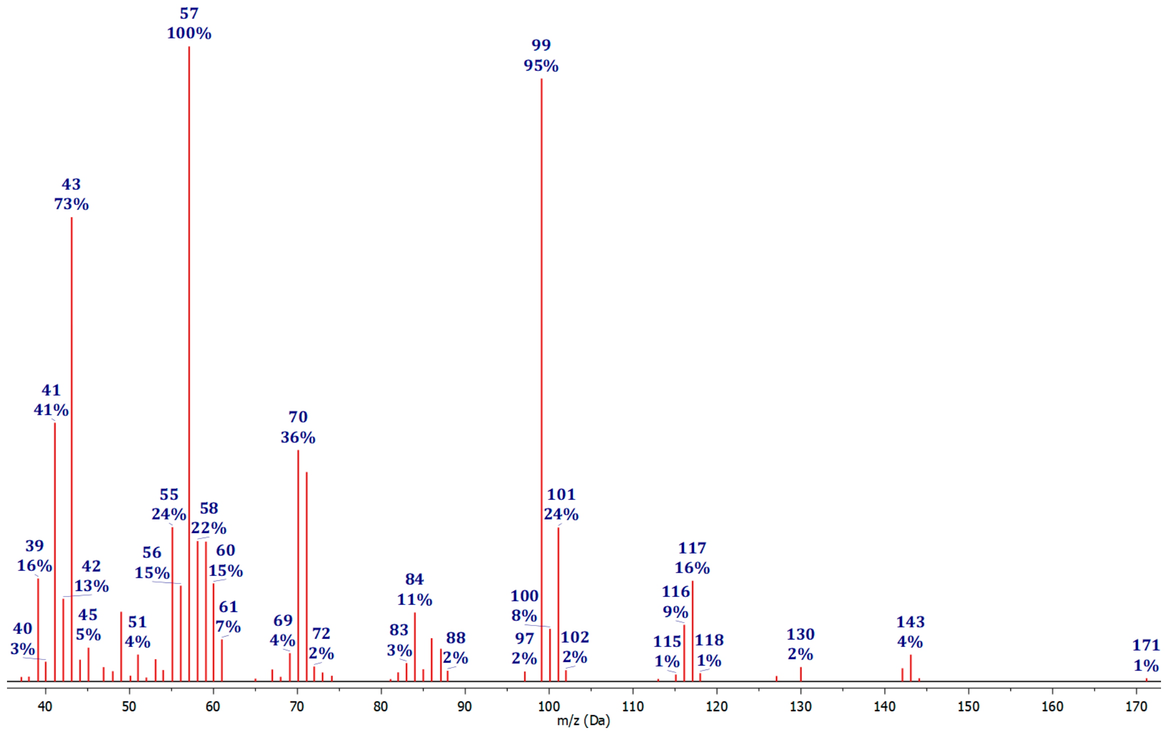

Figure S7. Mass spectrum of 1-methylbutyl 2,2-dimethylbutanoate (**1b**)

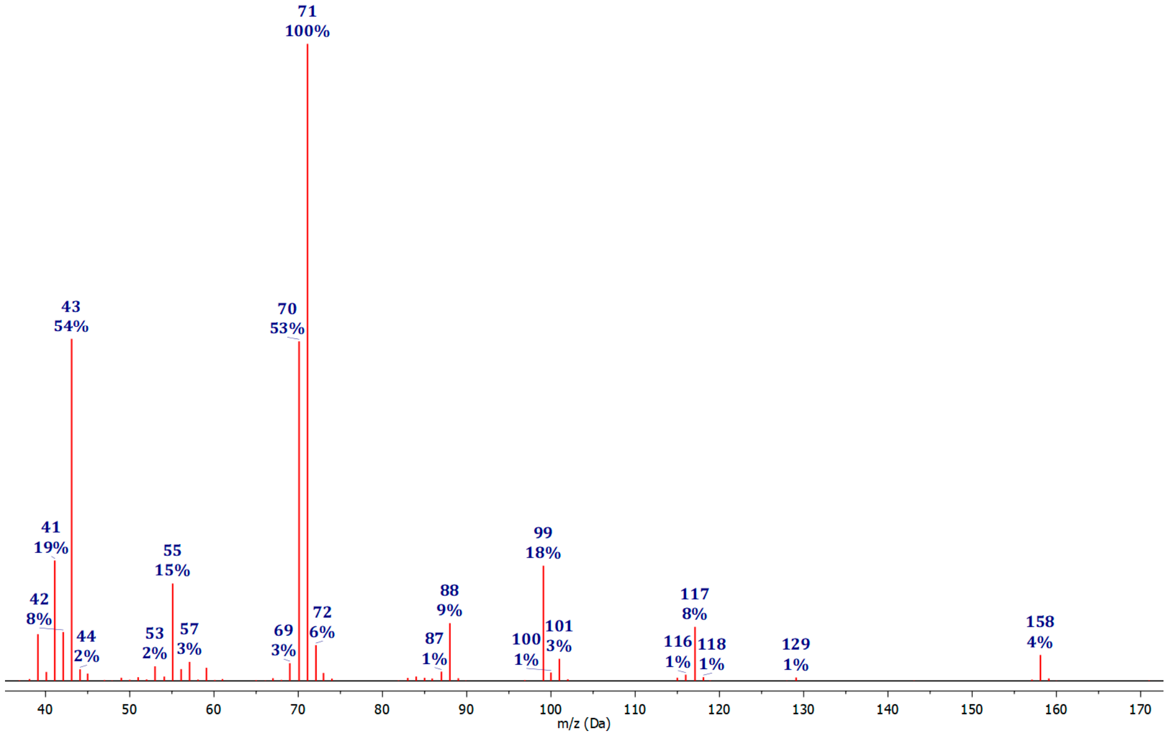

Figure S8. Mass spectrum of 2-methylbutyl 2,2-dimethylbutanoate (**2b**)

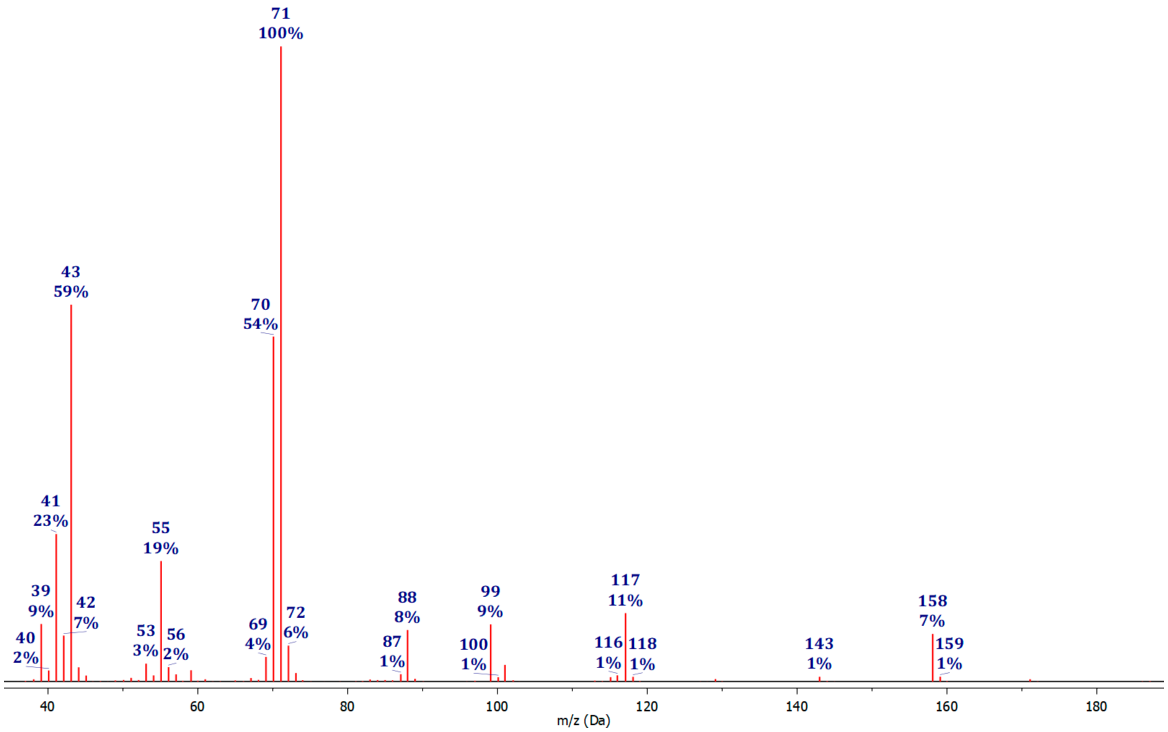

Figure S9. Mass spectrum of 3-methylbutyl 2,2-dimethylbutanoate (**3b**)

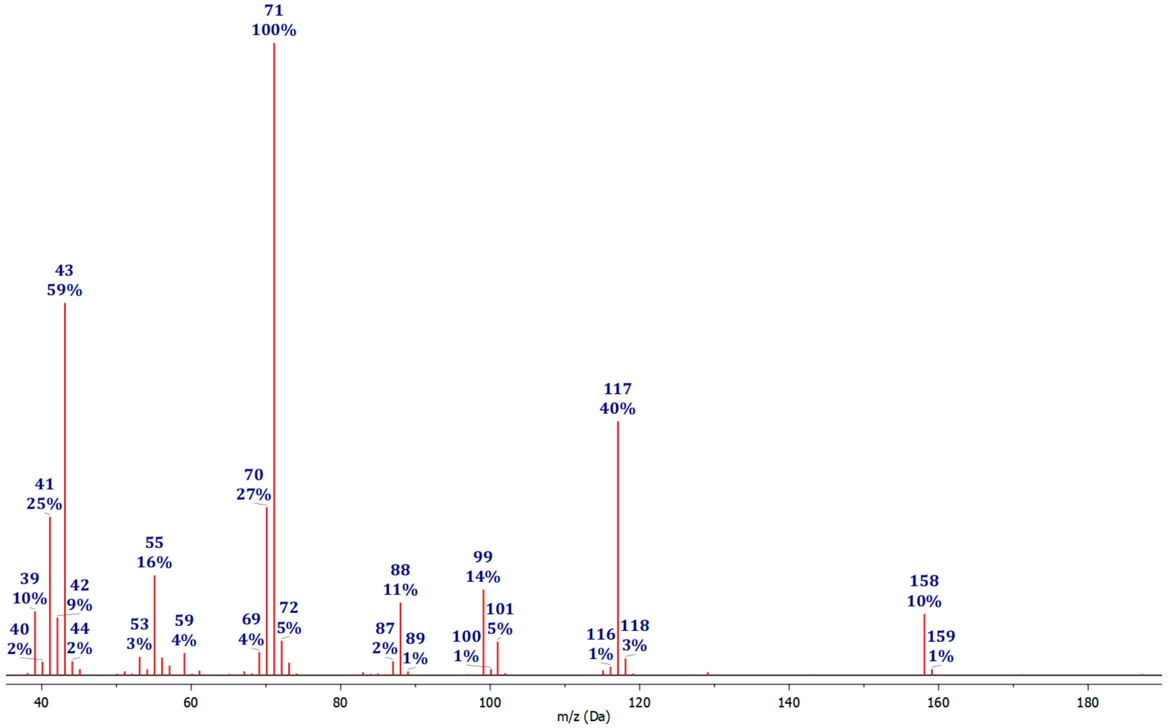

Figure S10. Mass spectrum of pentyl 2,2-dimethylbutanoate (**4b**)

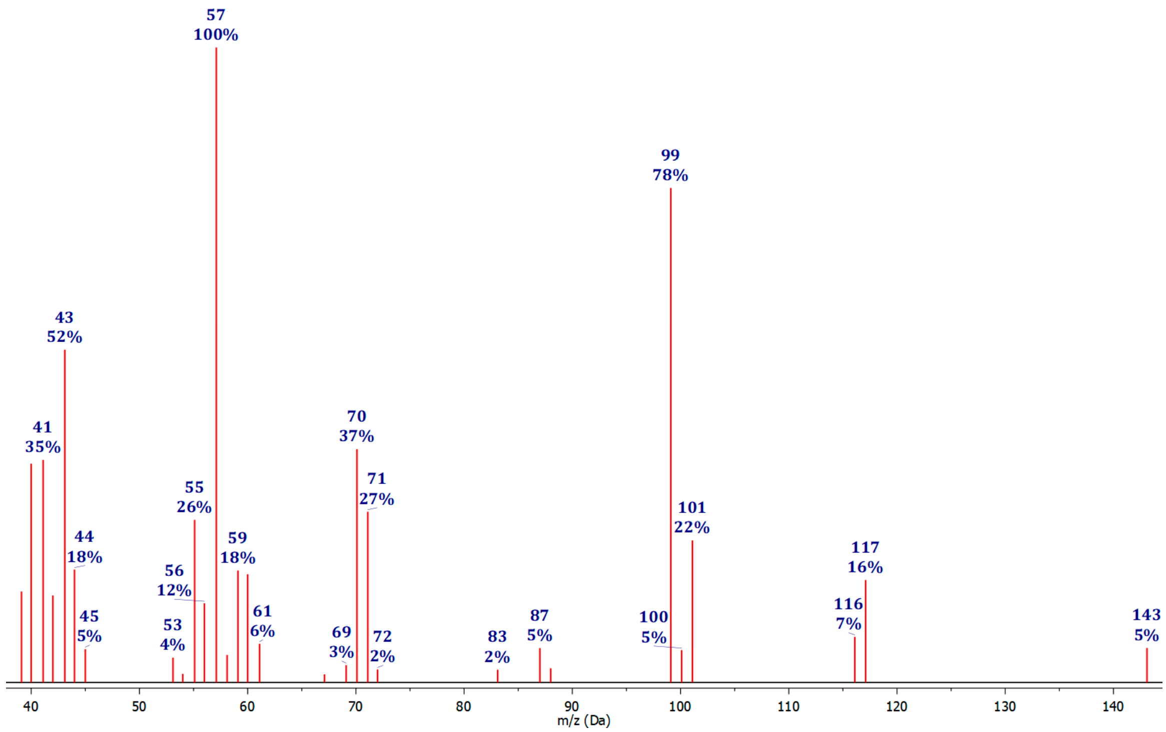

Figure S11. Mass spectrum of 1-methylbutyl 3,3-dimethylbutanoate (**1c**)

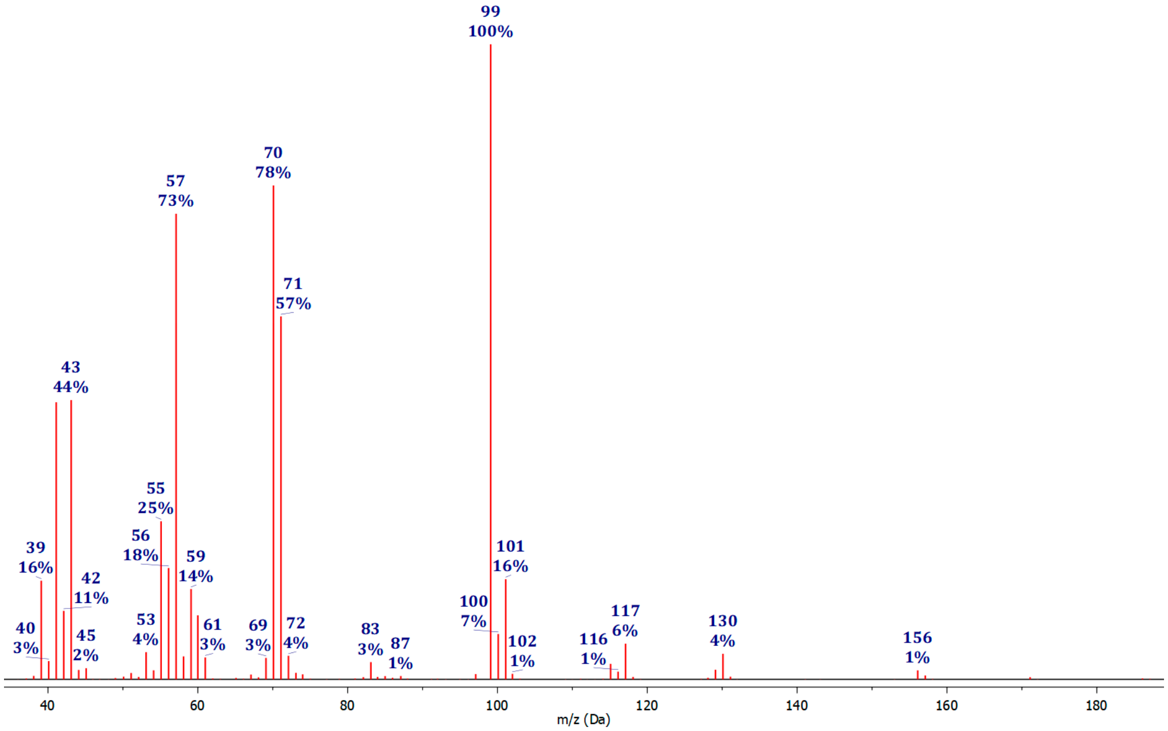

Figure S12. Mass spectrum of 2-methylbutyl 3,3-dimethylbutanoate (**2c**)

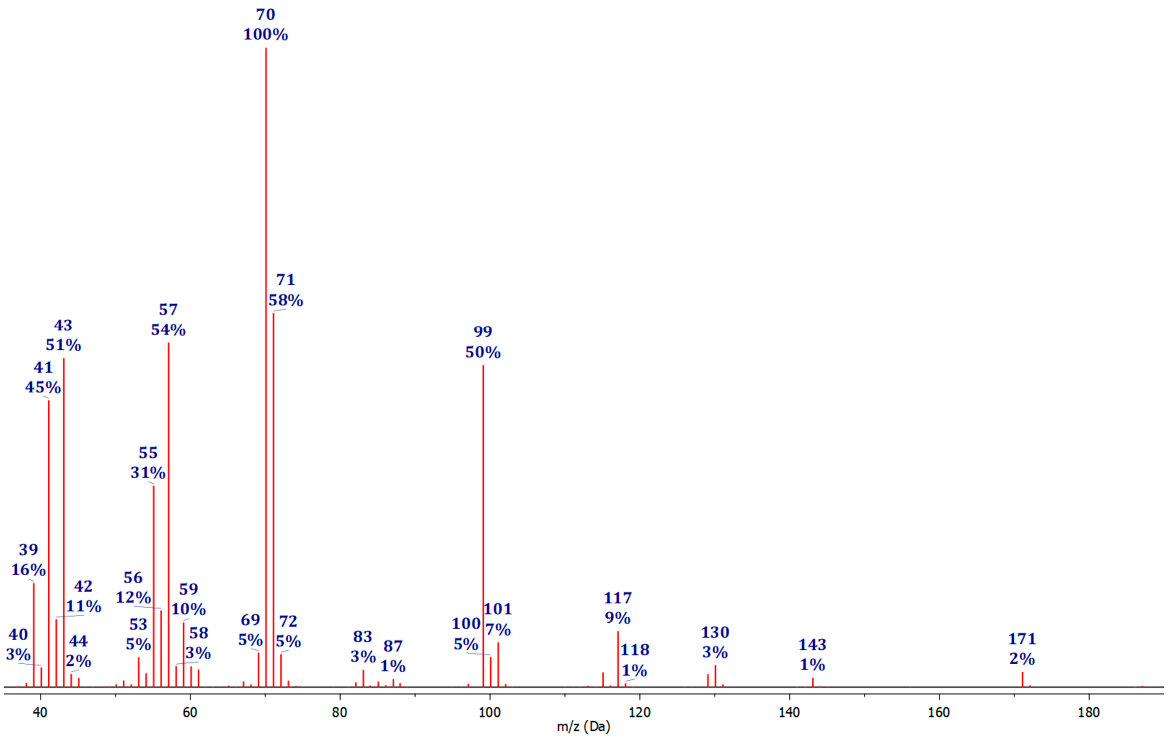

Figure S13. Mass spectrum of 3-methylbutyl 3,3-dimethylbutanoate (**3c**)

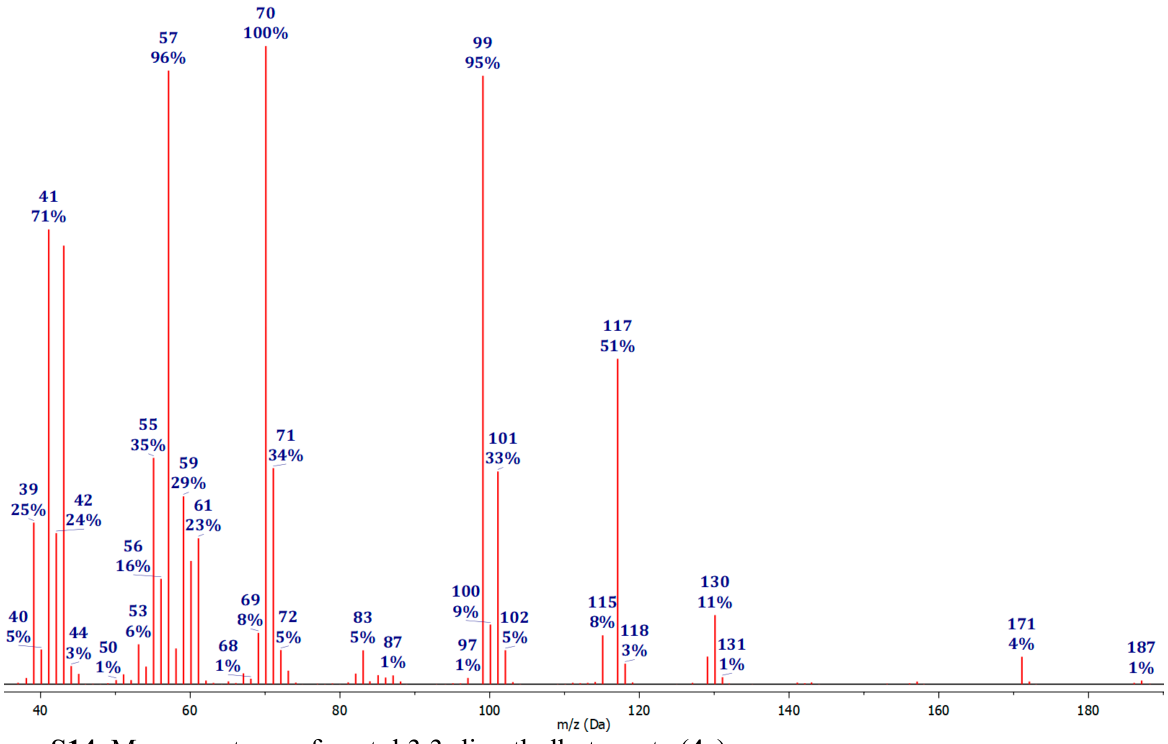

Figure S14. Mass spectrum of pentyl 3,3-dimethylbutanoate (**4c**)

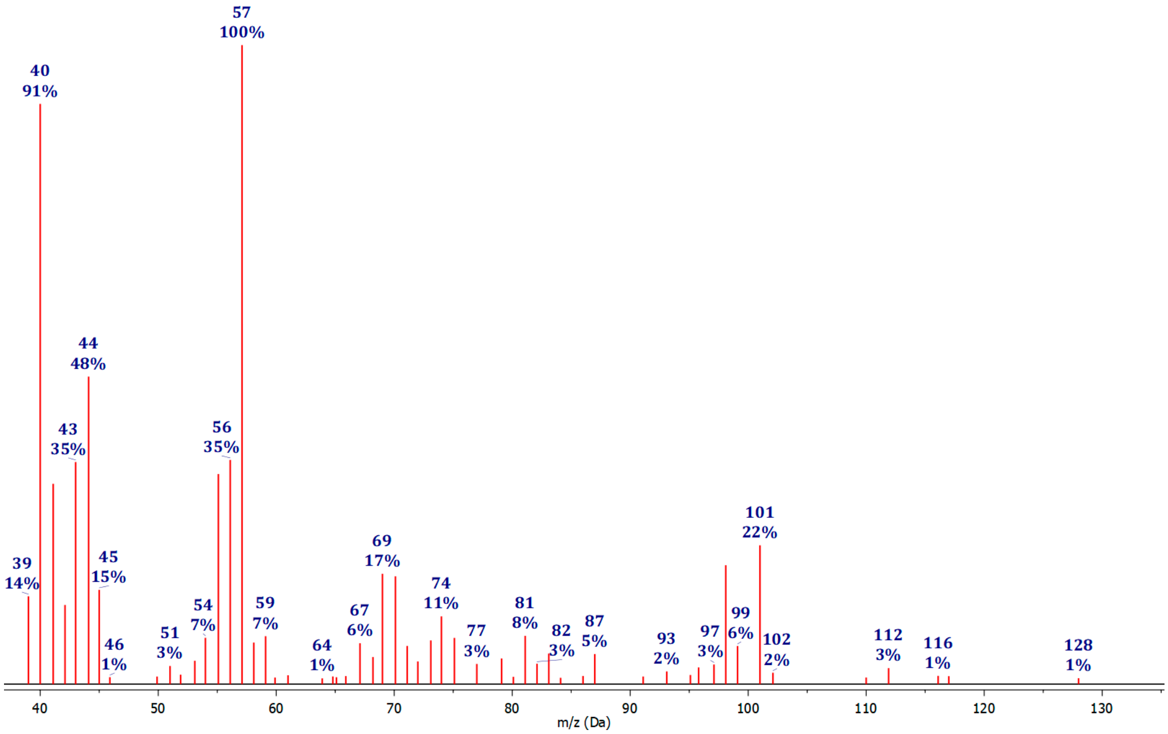

Figure S15. Mass spectrum of 1-methylbutyl 2,3-dimethylbutanoate (**1d**)

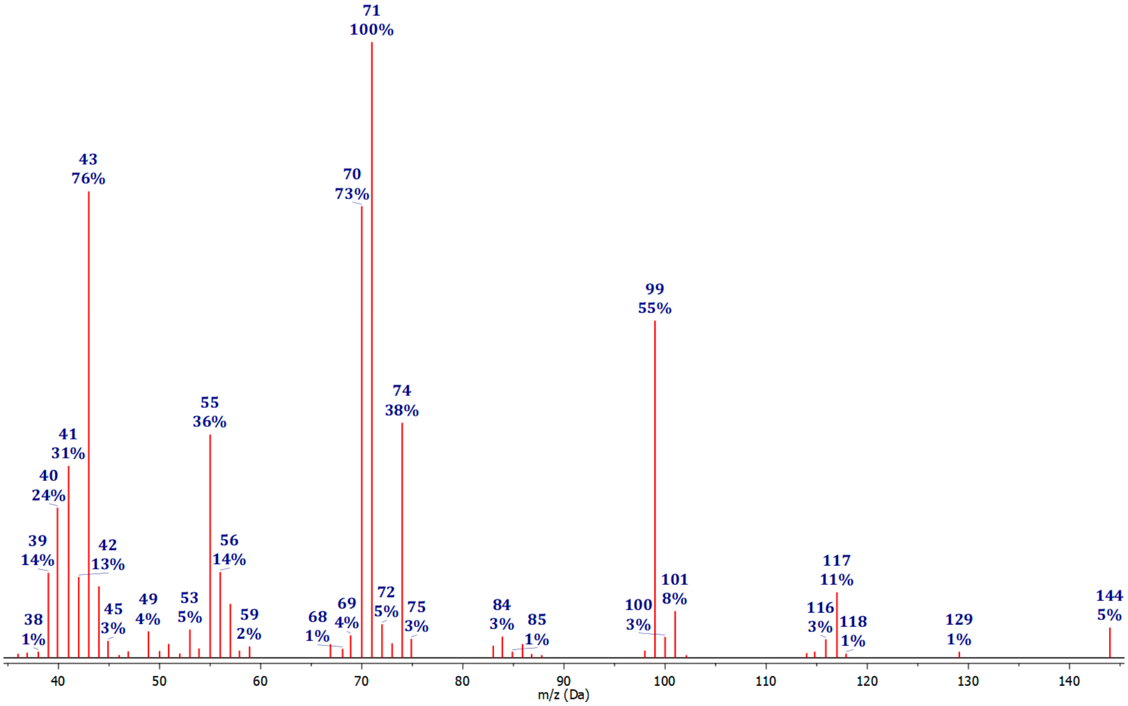

Figure S16. Mass spectrum of 2-methylbutyl 2,3-dimethylbutanoate (**2d**)

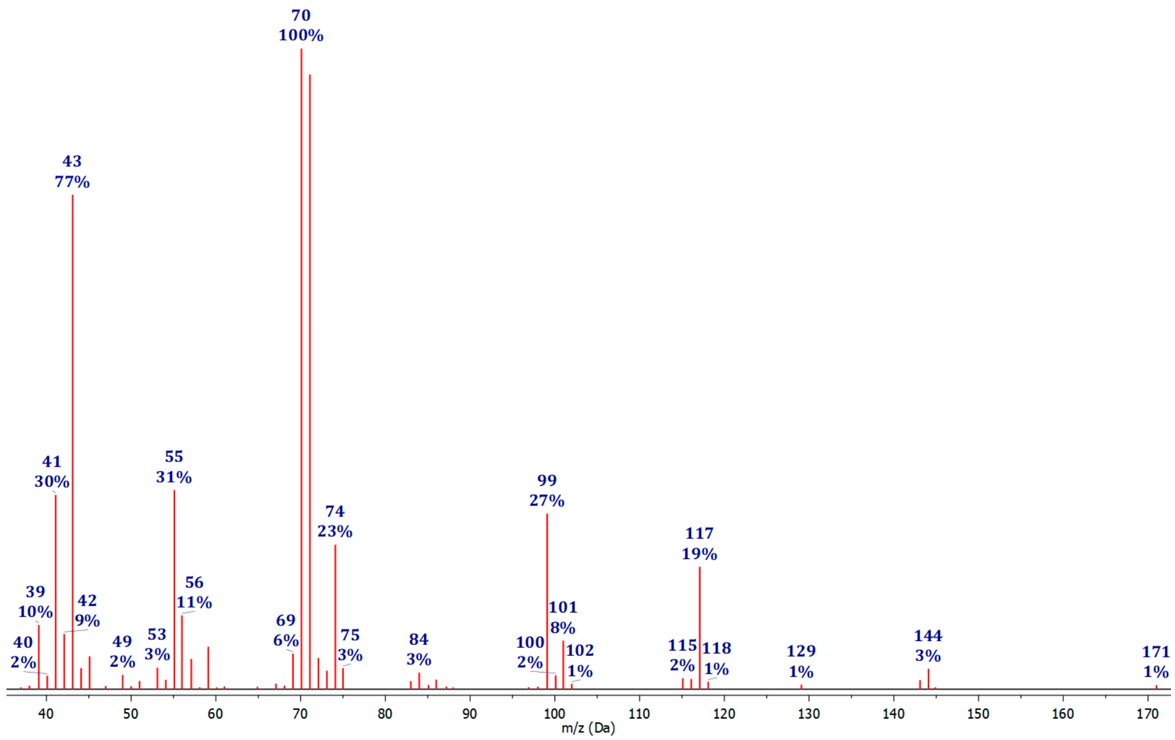

Figure S17. Mass spectrum of 3-methylbutyl 2,3-dimethylbutanoate (**3d**)

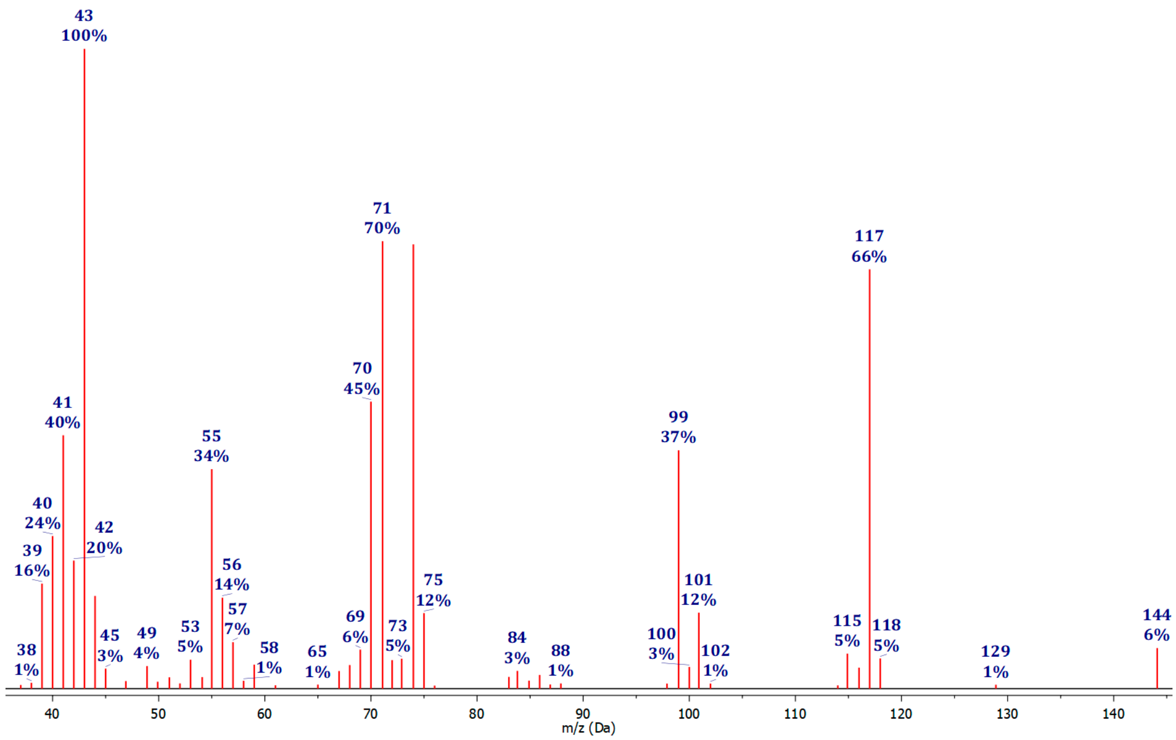

Figure S18. Mass spectrum of pentyl 2,3-dimethylbutanoate (**4d**)

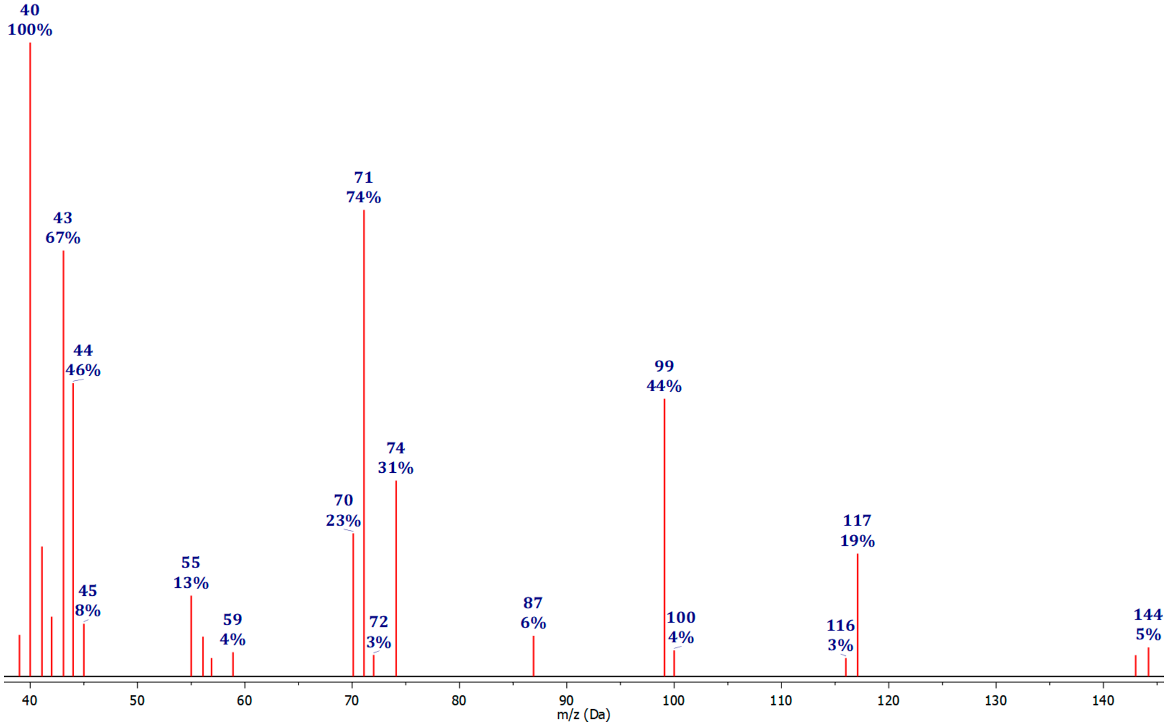

Figure S19a. Mass spectrum of 1-methylbutyl 2-methylpentanoate (**1e epimer I**)

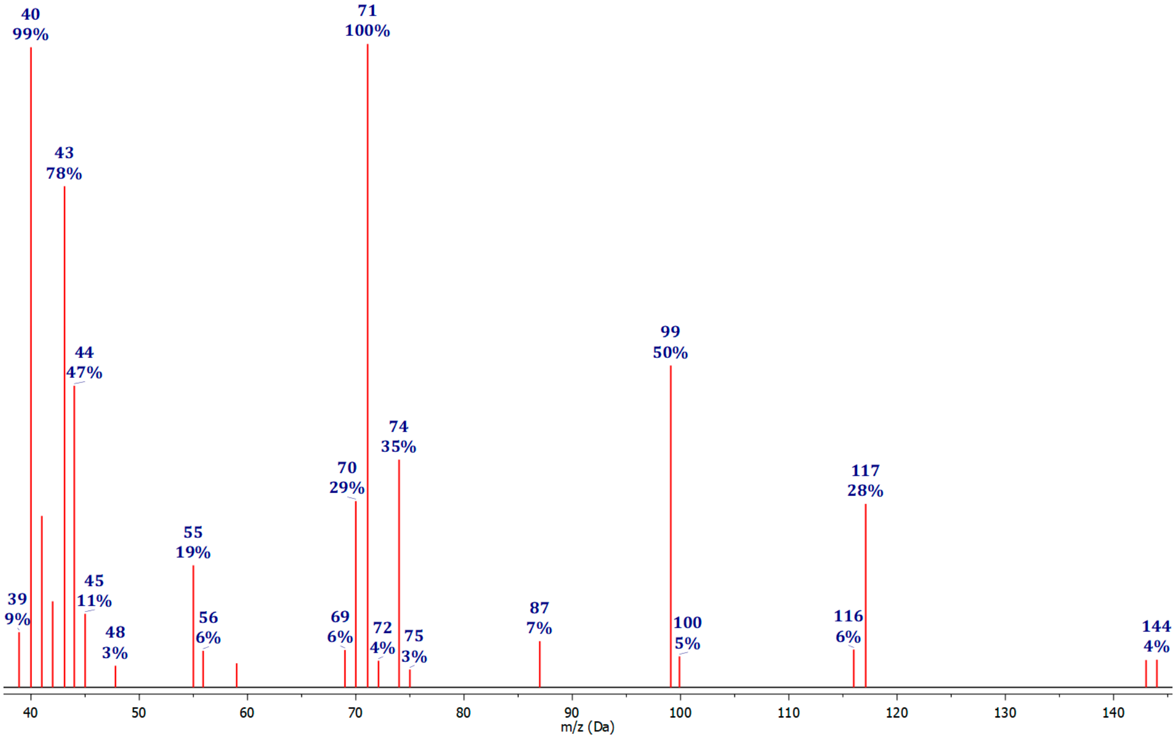

Figure S19b. Mass spectrum of 1-methylbutyl 2-methylpentanoate (**1e epimer II**)

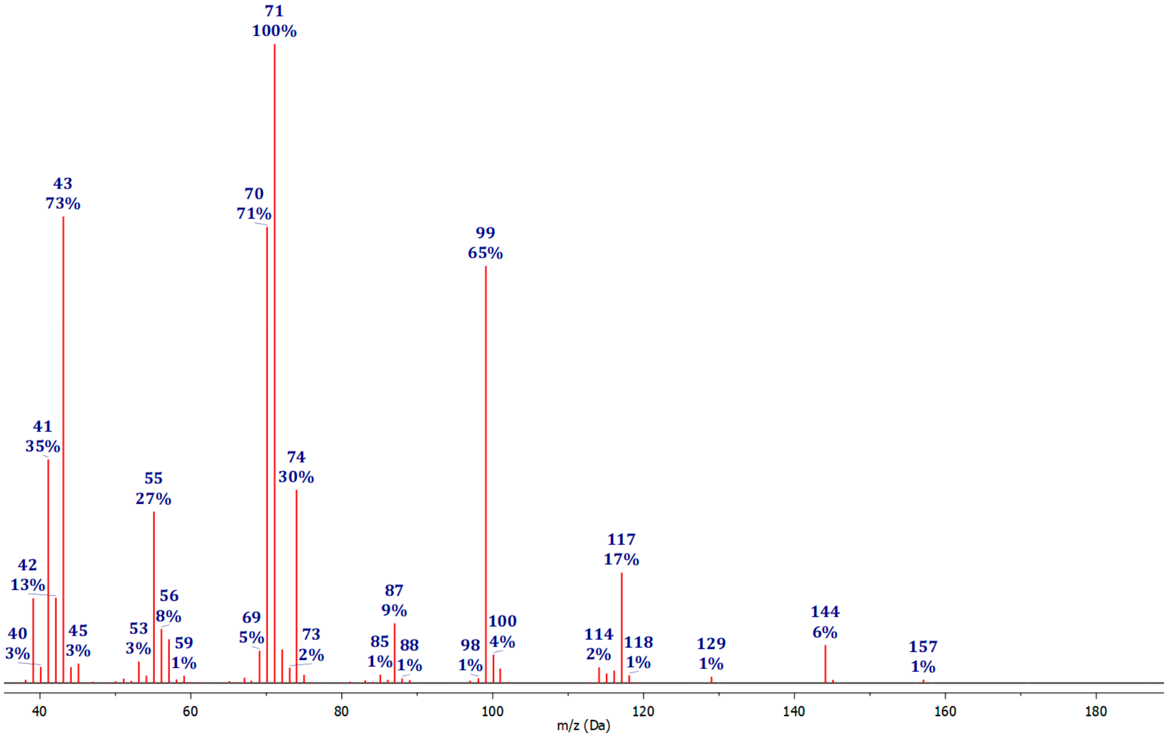

Figure S20. Mass spectrum of 2-methylbutyl 2-methylpentanoate (**2e**)

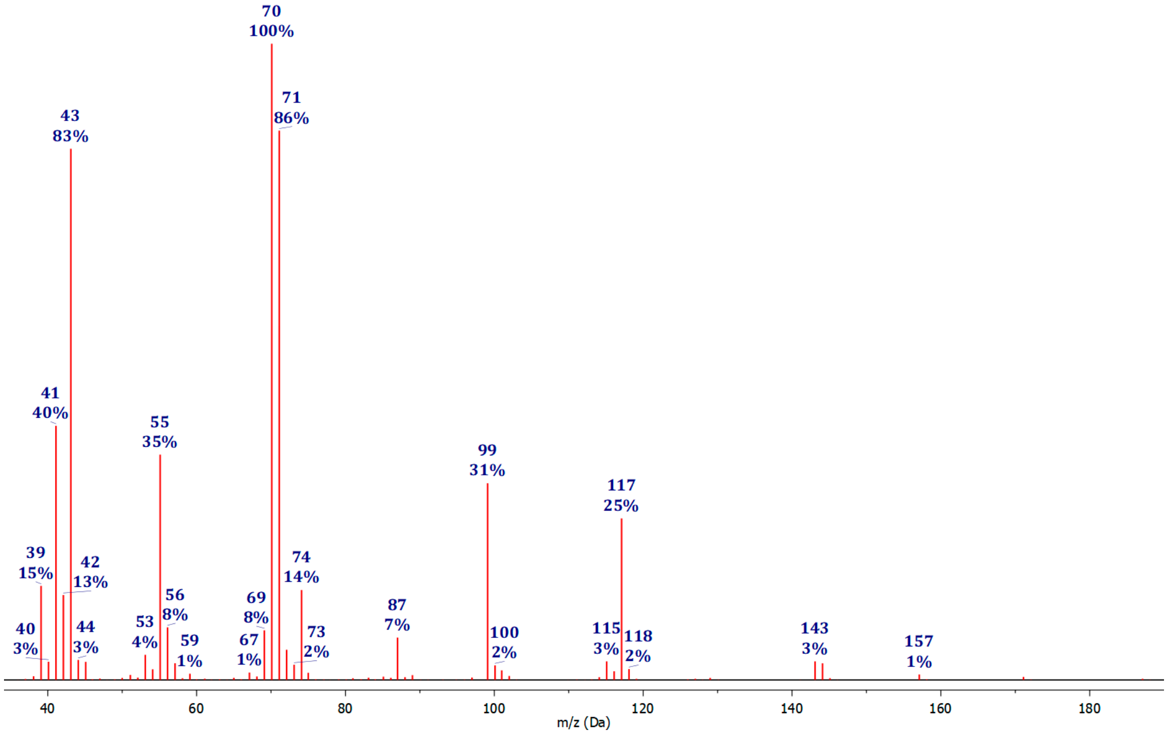

Figure S21. Mass spectrum of 3-methylbutyl 2-methylpentanoate (**3e**)

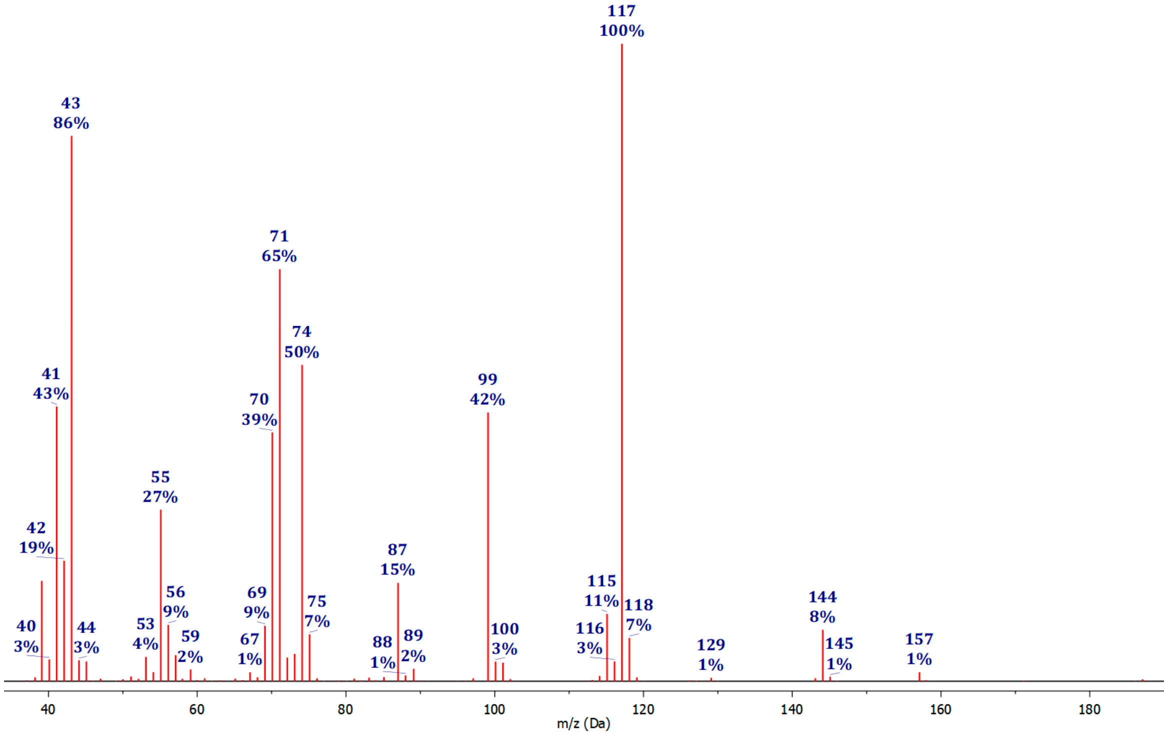

Figure S22. Mass spectrum of pentyl 2-methylpentanoate (**4e**)

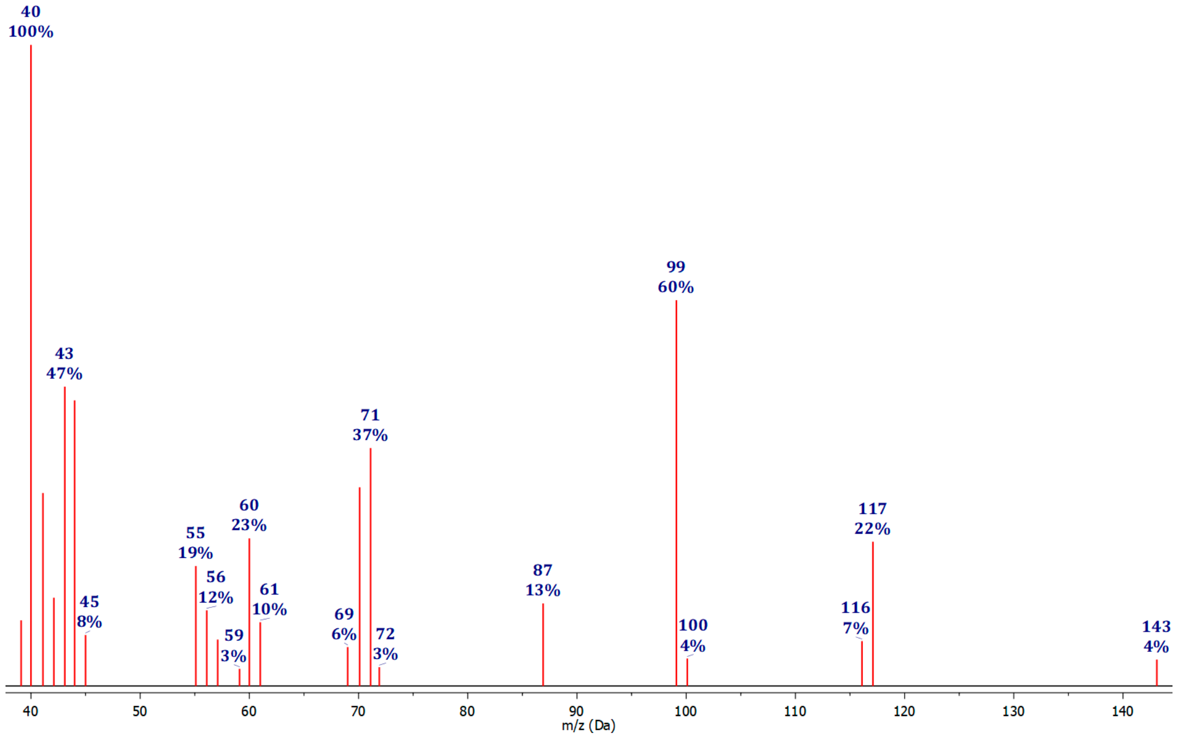

Figure S23. Mass spectrum of 1-methylbutyl 3-methylpentanoate (**1f**)

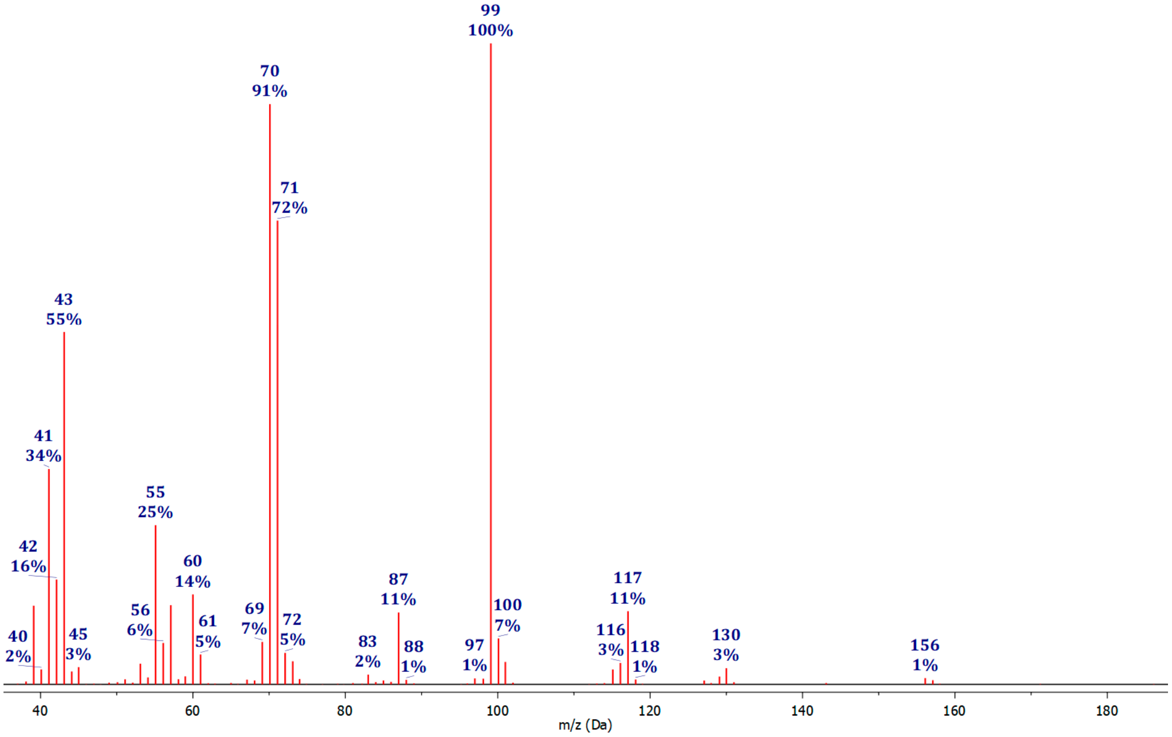

Figure S24. Mass spectrum of 2-methylbutyl 3-methylpentanoate (**2f**)

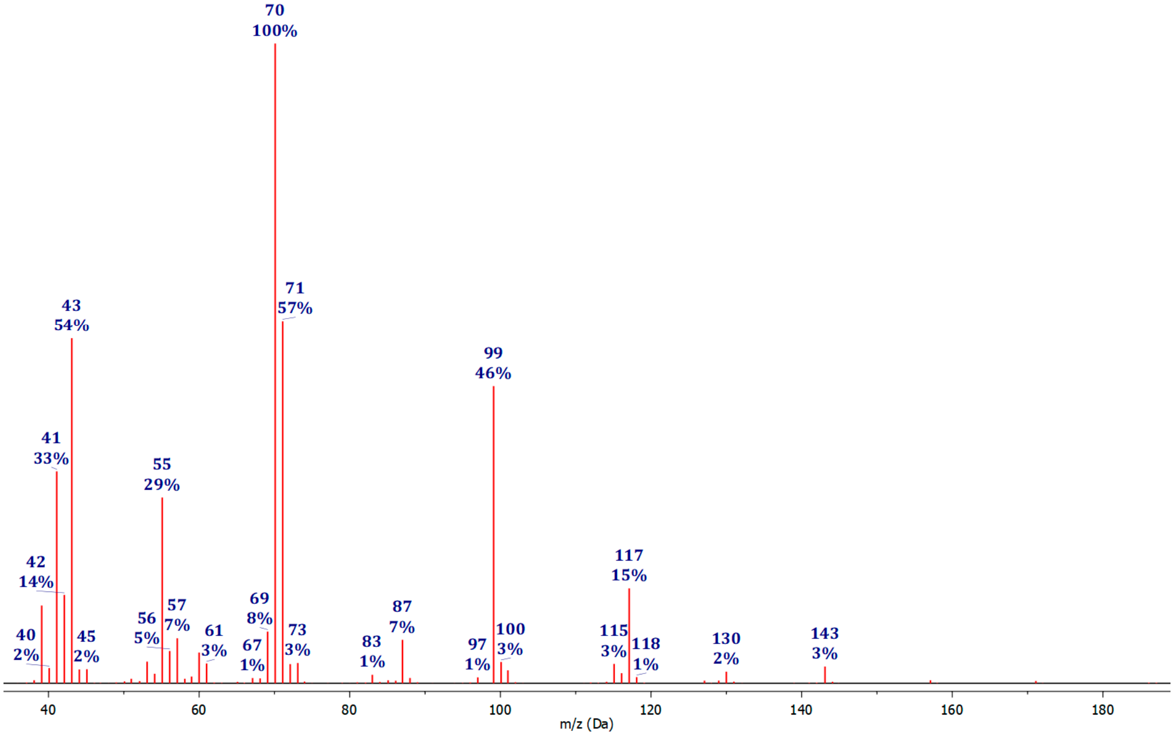

Figure S25a. Mass spectrum of 3-methylbutyl 3-methylpentanoate (**3f**)

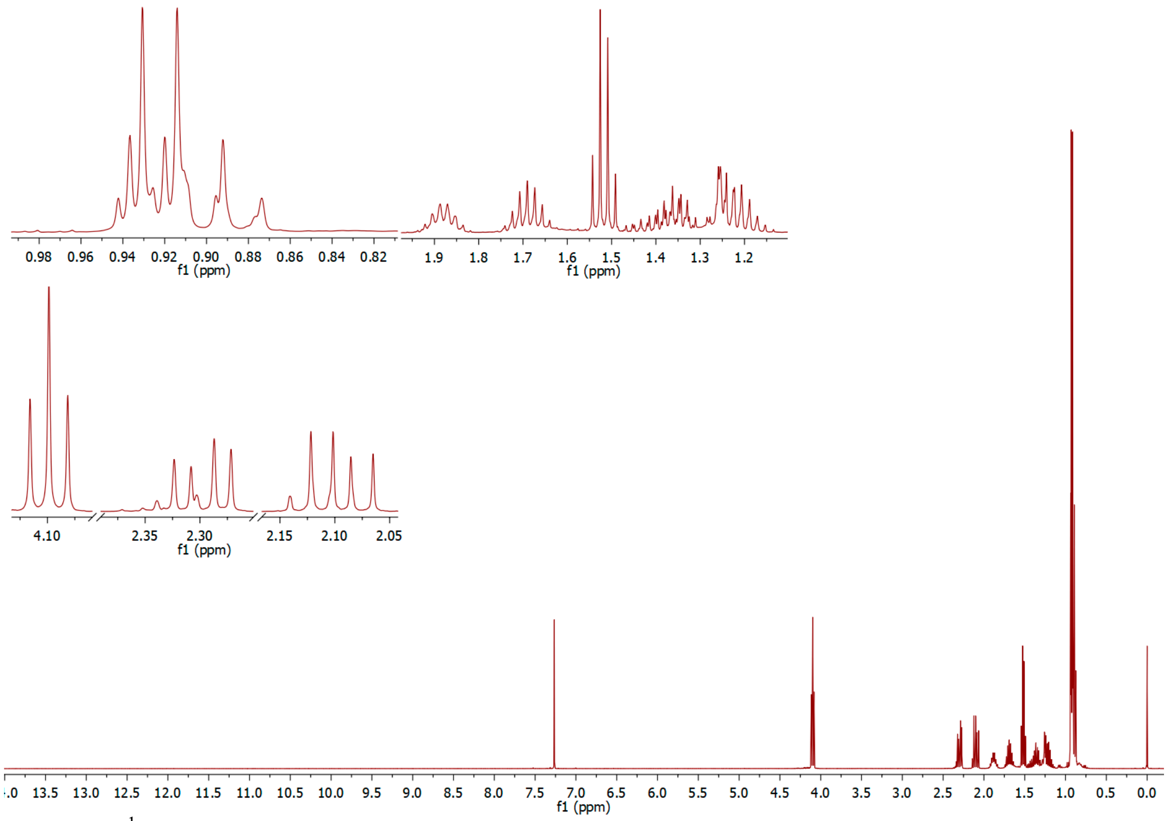

Figure S25b. ^{1}H NMR spectrum of 3-methylbutyl 3-methylpentanoate (**3f**) recorded in $CDCl_3$

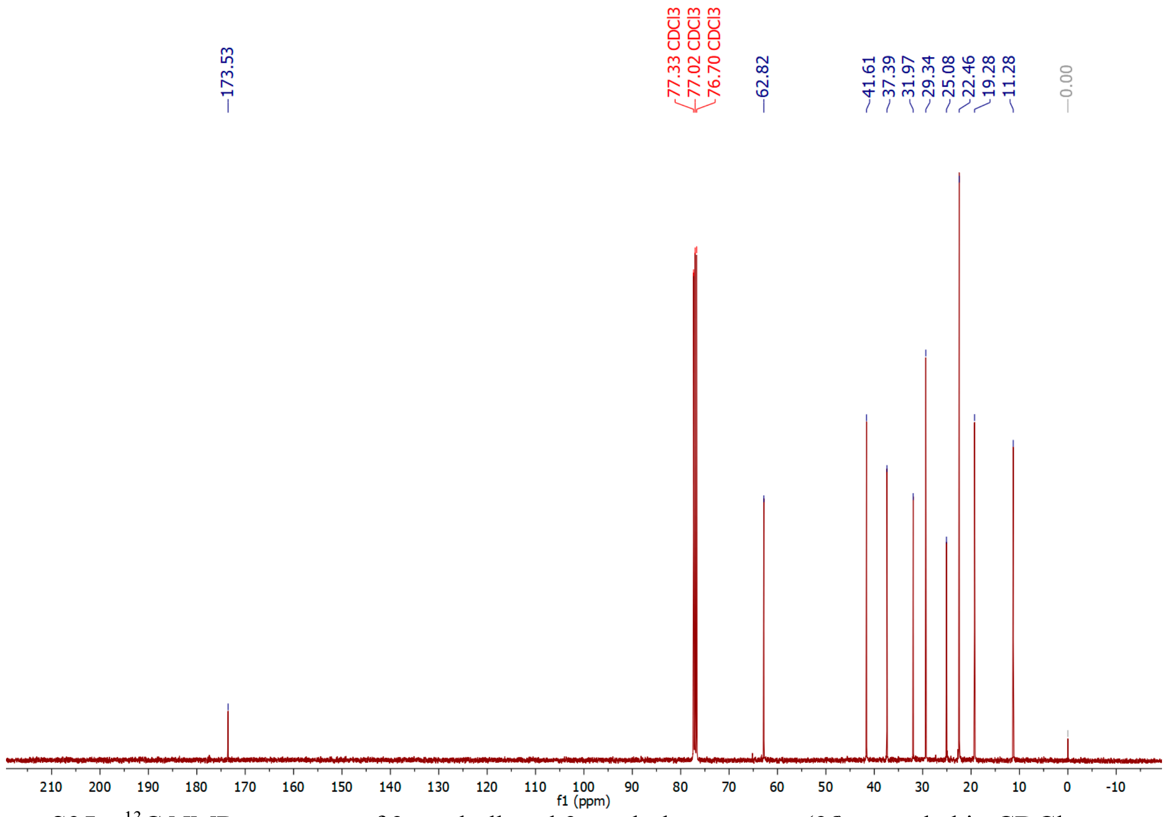

Figure S25c. ^{13}C NMR spectrum of 3-methylbutyl 3-methylpentanoate (**3f**) recorded in $CDCl_3$

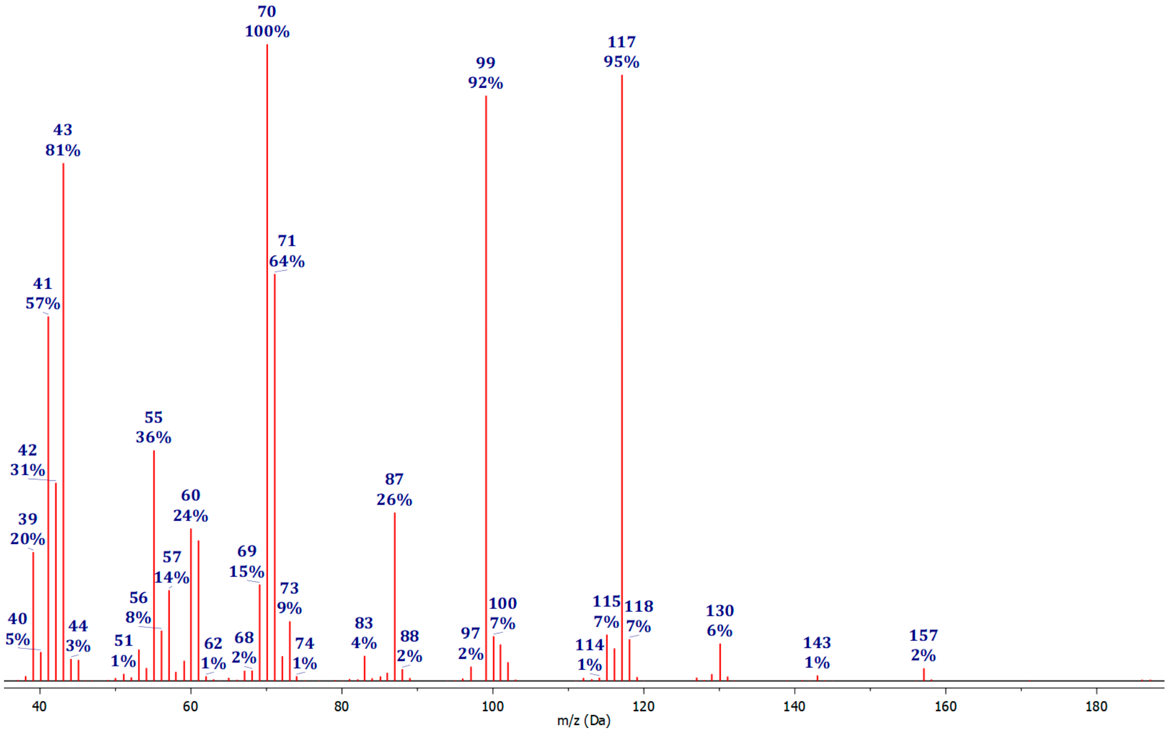

Figure S26. Mass spectrum of pentyl 3-methylpentanoate (**4f**)

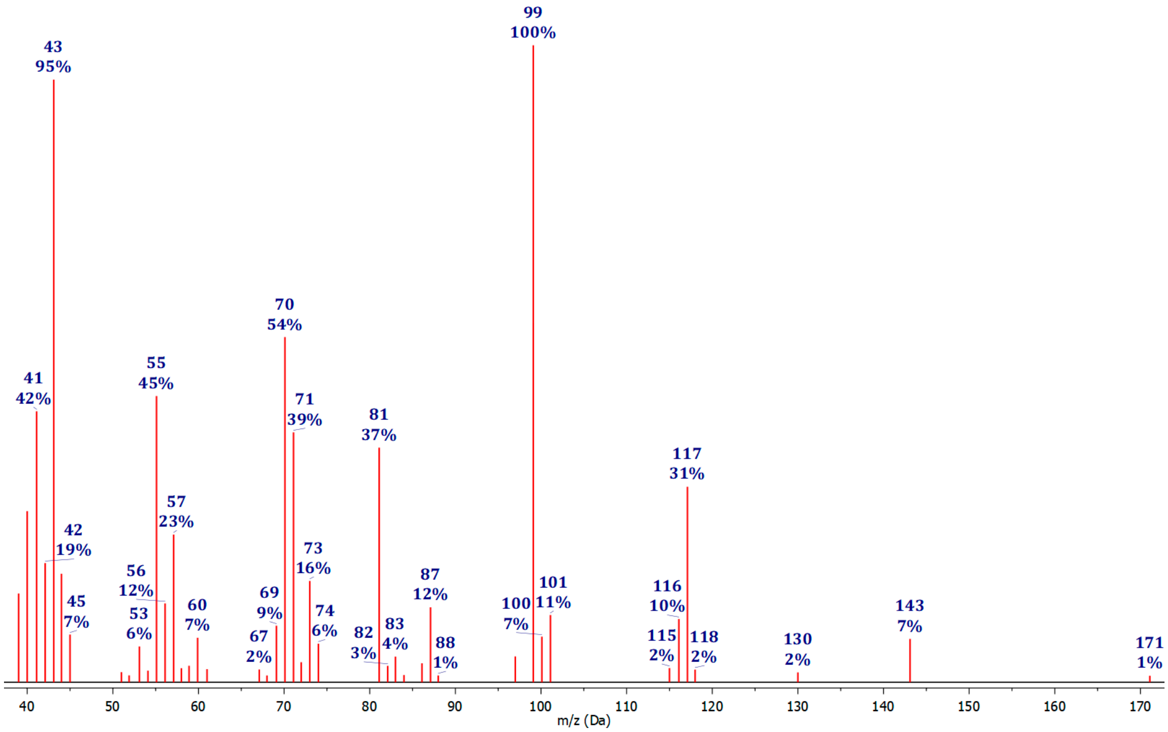

Figure S27. Mass spectrum of 1-methylbutyl 4-methylpentanoate (**1g**)

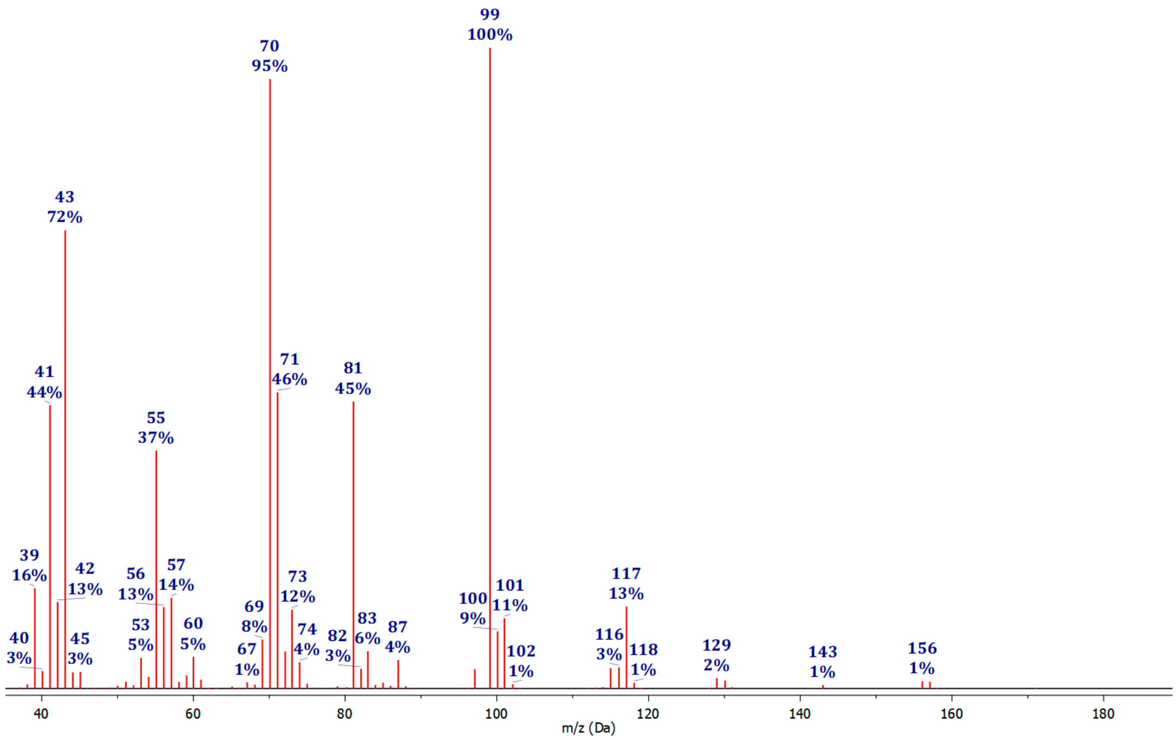

Figure S28. Mass spectrum of 2-methylbutyl 4-methylpentanoate (**2g**)

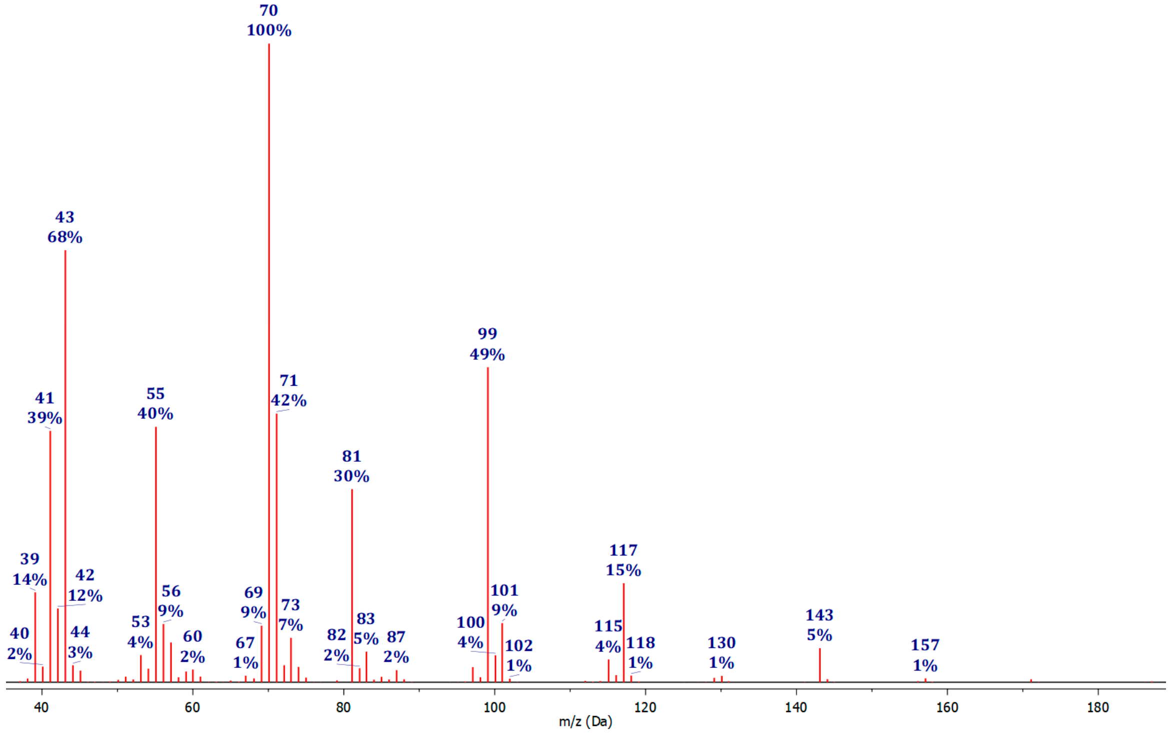

Figure S29. Mass spectrum of 3-methylbutyl 4-methylpentanoate (**3g**)

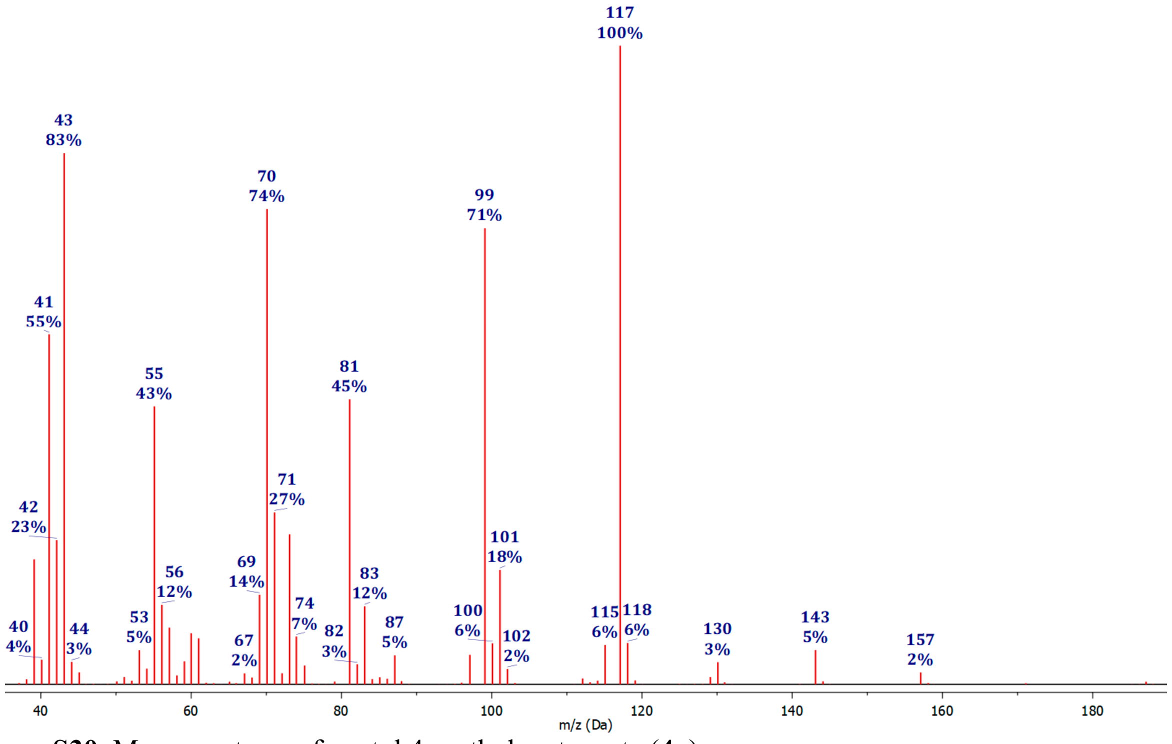

Figure S30. Mass spectrum of pentyl 4-methylpentanoate (**4g**)

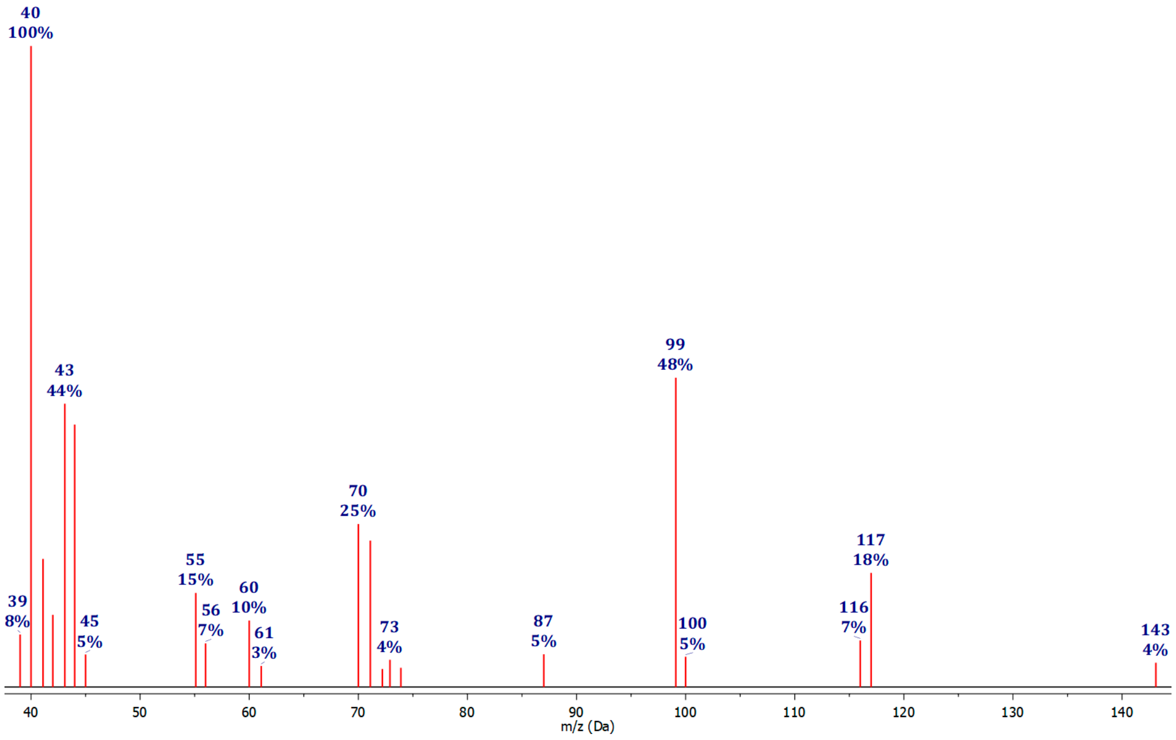

Figure S31. Mass spectrum of 1-methylbutyl hexanoate (**1h**)

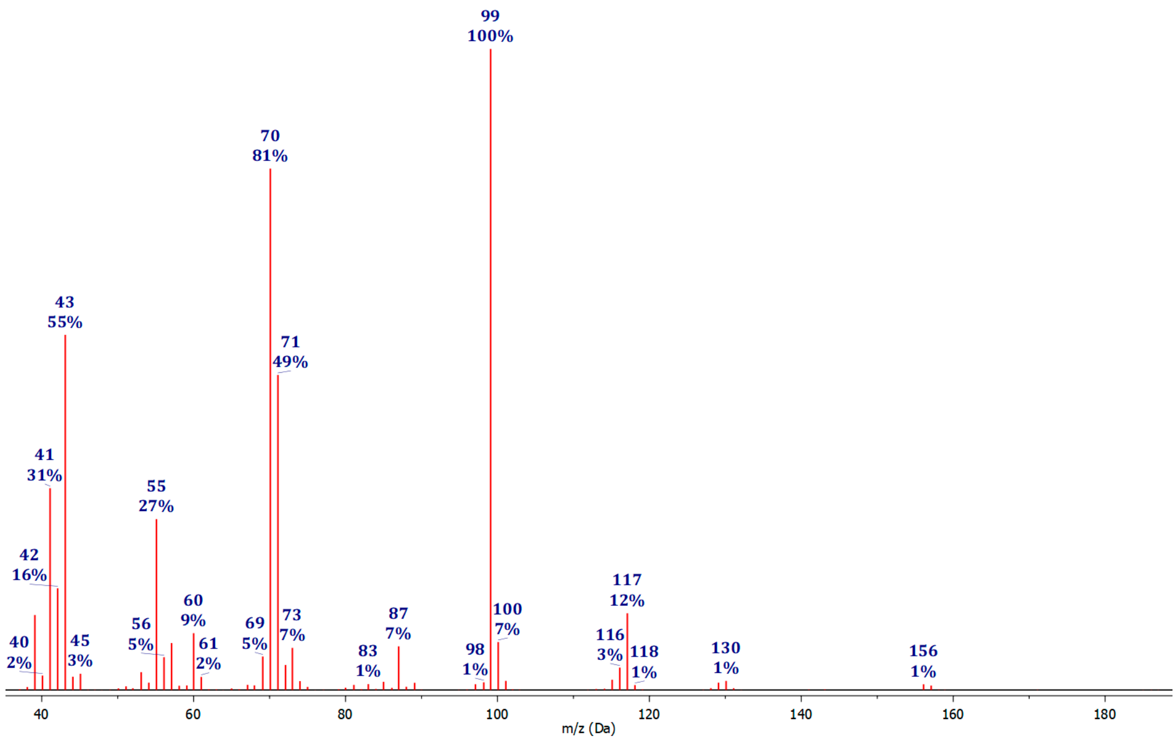

Figure S32. Mass spectrum of 2-methylbutyl hexanoate (**2h**)

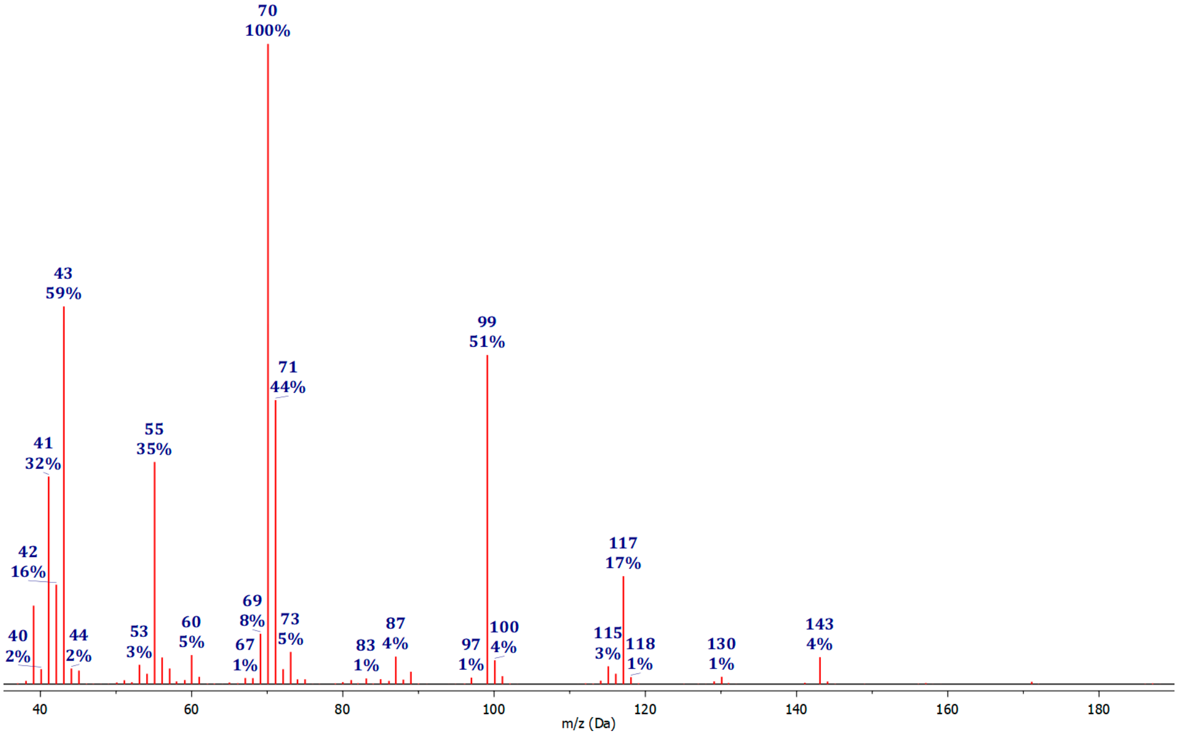

Figure S33. Mass spectrum of 3-methylbutyl hexanoate (**3h**)

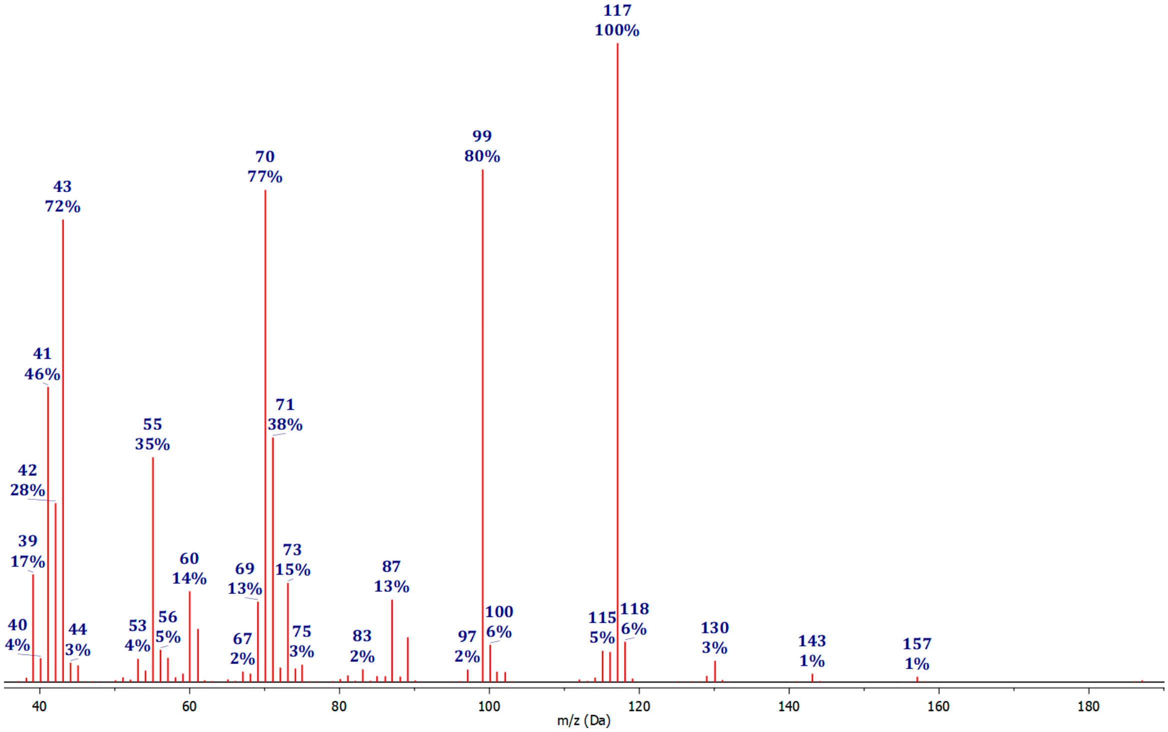

Figure S34. Mass spectrum of pentyl hexanoate (**4h**)

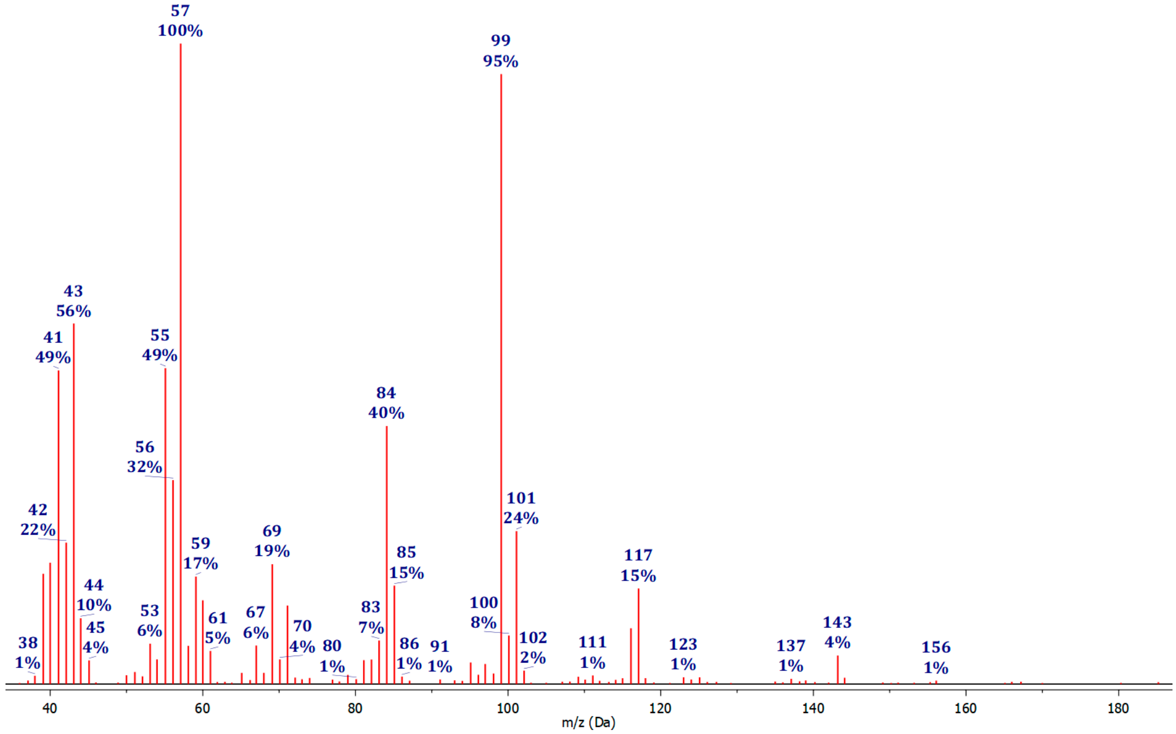

Figure S35. Mass spectrum of 1-methylpentyl 2,2-dimethylbutanoate (**5b**)

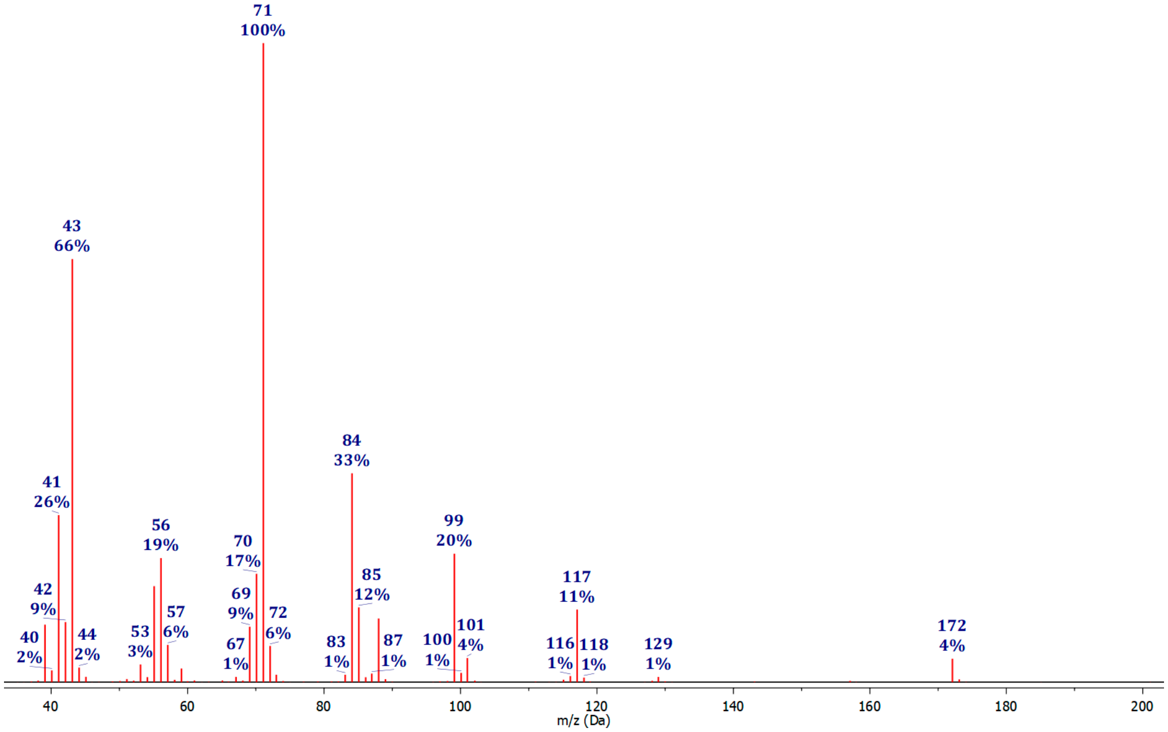

Figure S36. Mass spectrum of 2-methylpentyl 2,2-dimethylbutanoate (**6b**)

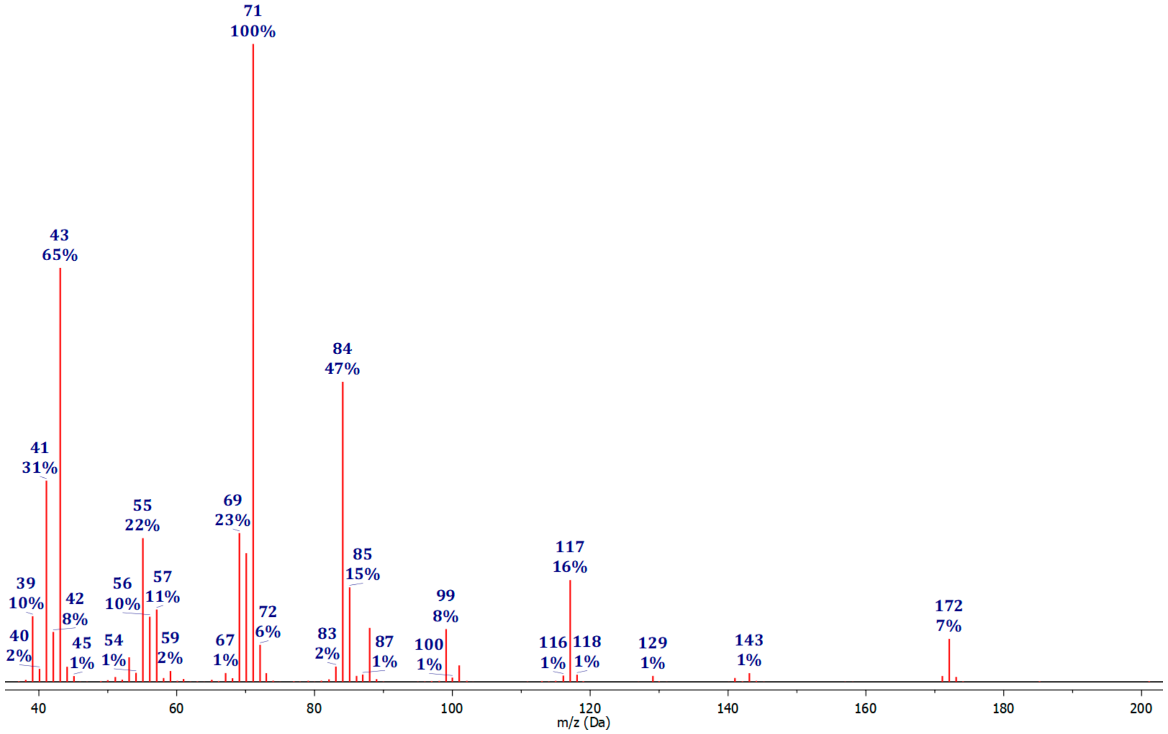

Figure S37. Mass spectrum of 3-methylpentyl 2,2-dimethylbutanoate (**7b**)

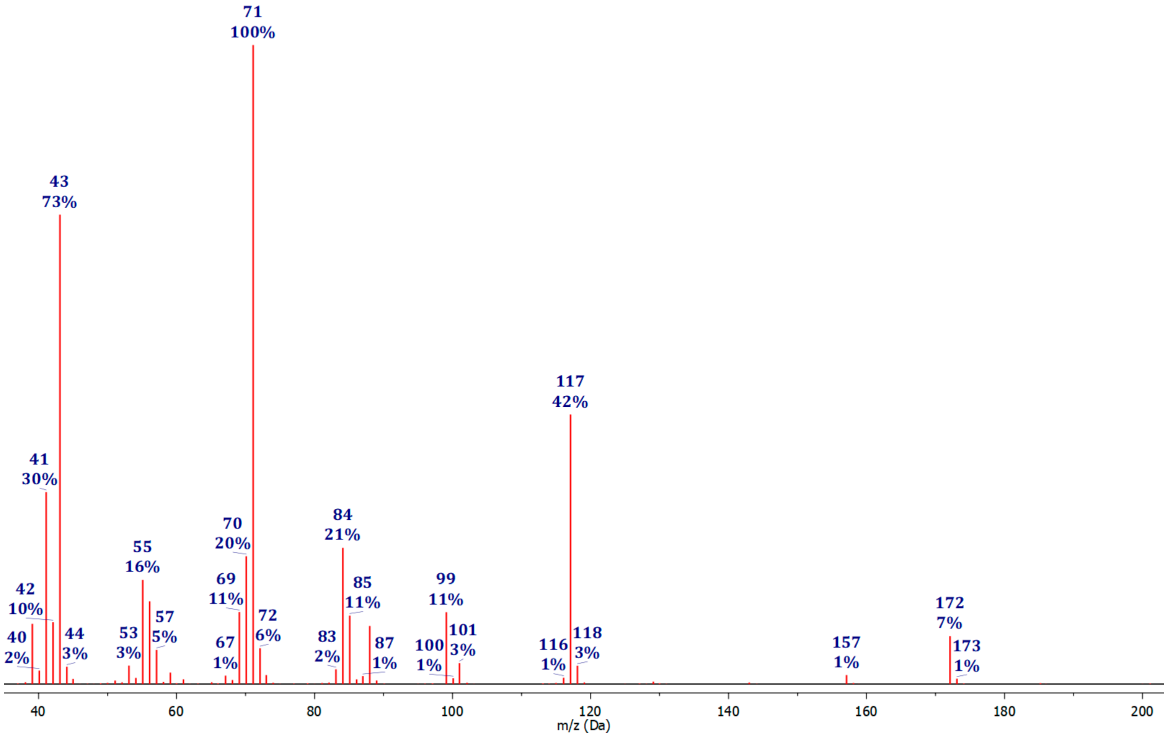

Figure S38. Mass spectrum of 4-methylpentyl 2,2-dimethylbutanoate (**8b**)

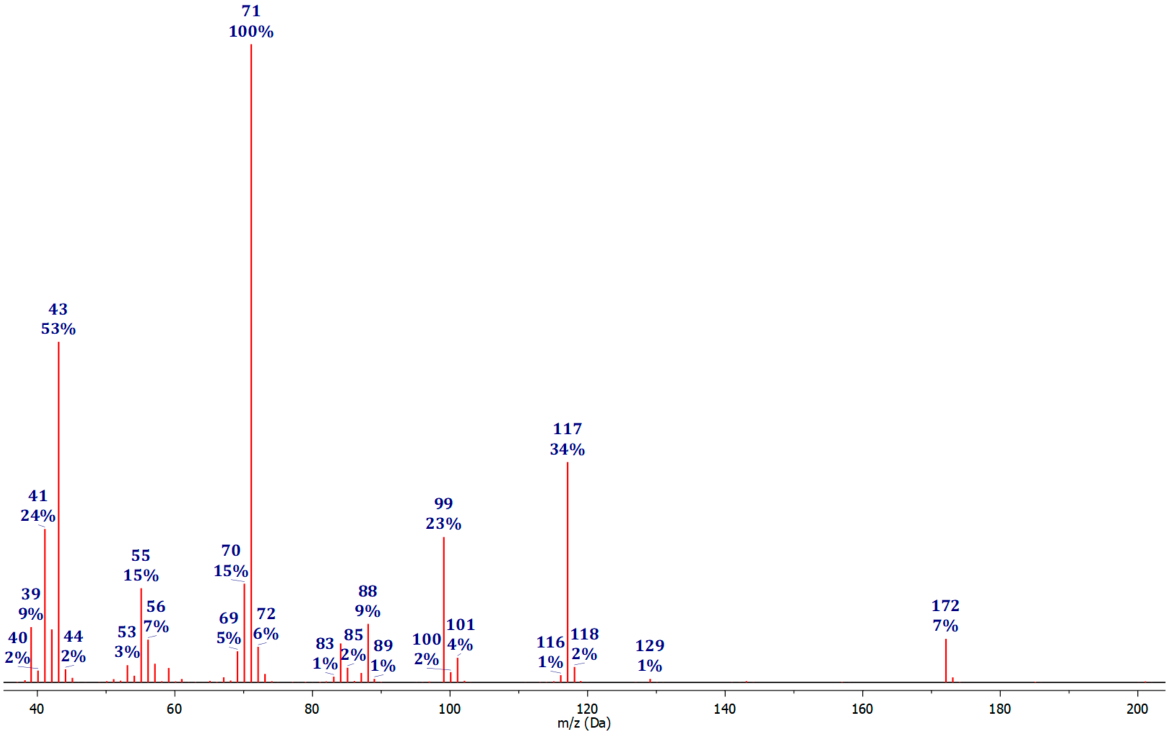

Figure S39. Mass spectrum of hexyl 2,2-dimethylbutanoate (**9b**)

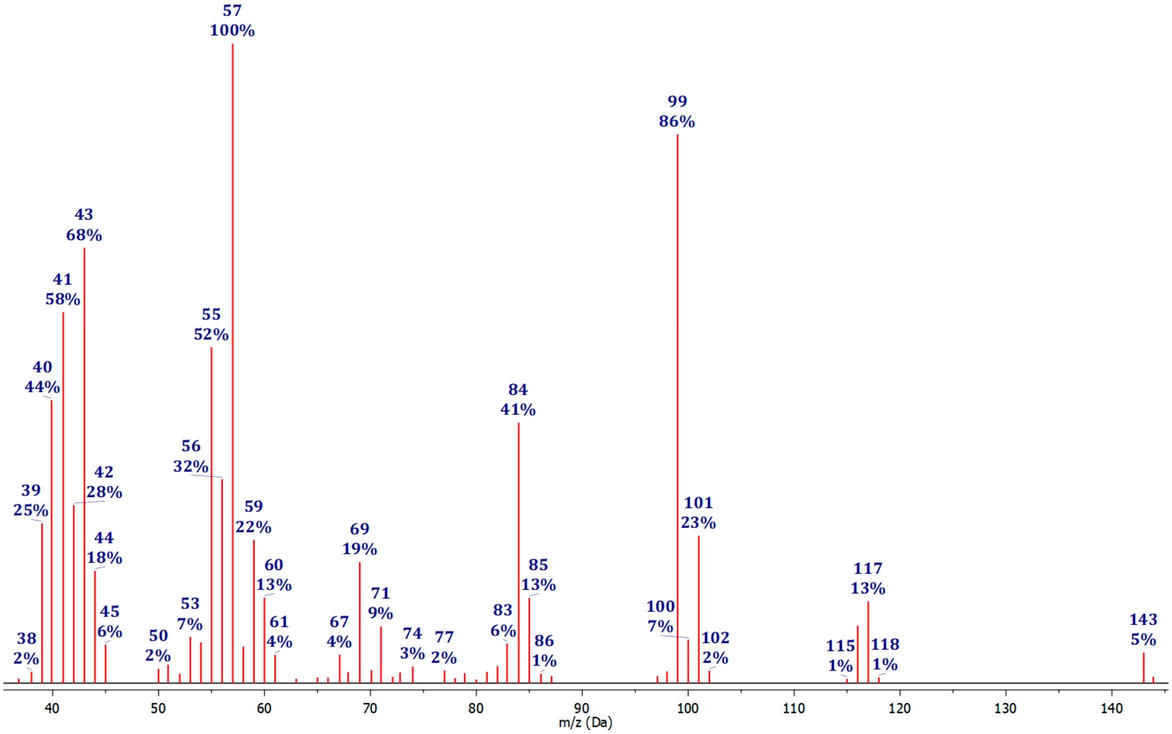

Figure S40. Mass spectrum of 1-methylpentyl 3,3-dimethylbutanoate (**5c**)

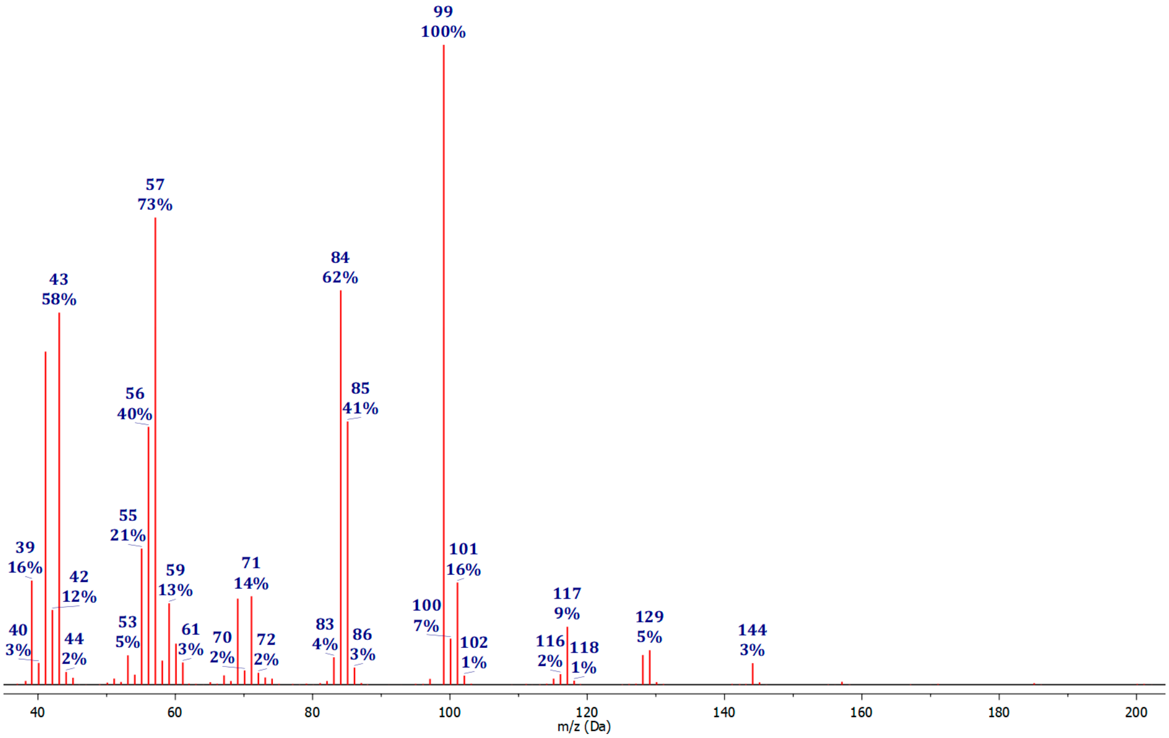

Figure S41. Mass spectrum of 2-methylpentyl 3,3-dimethylbutanoate (**6c**)

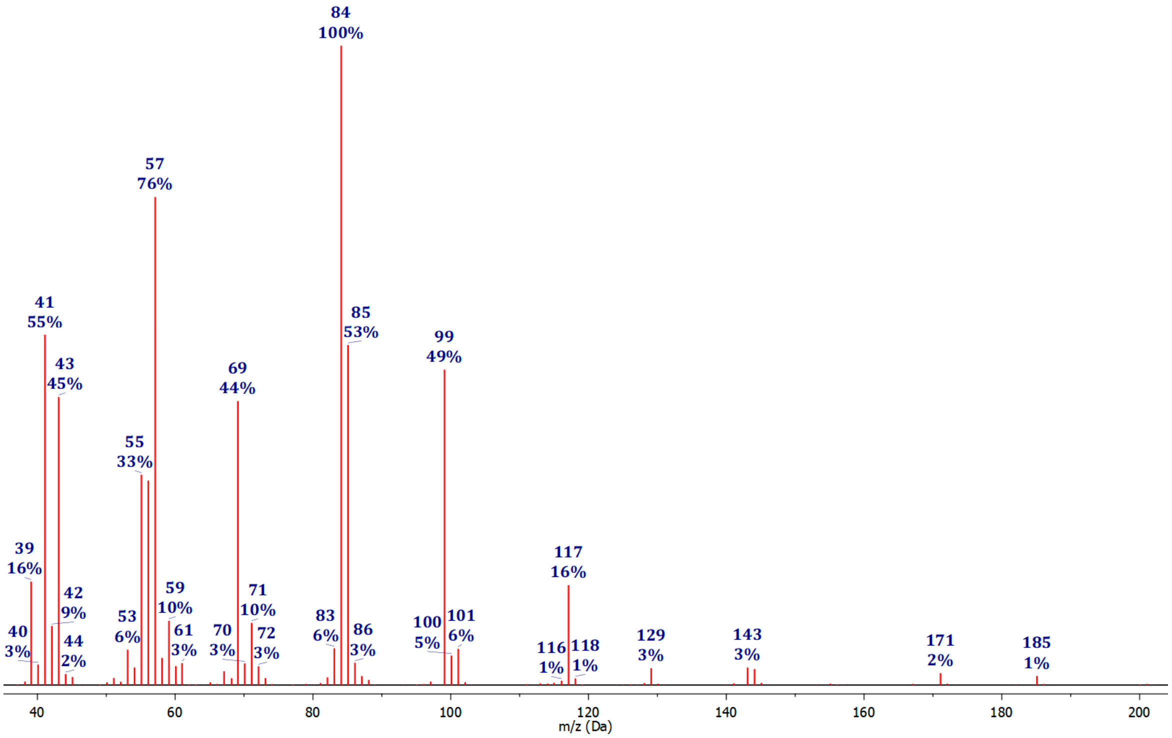

Figure S42. Mass spectrum of 3-methylpentyl 3,3-dimethylbutanoate (**7c**)

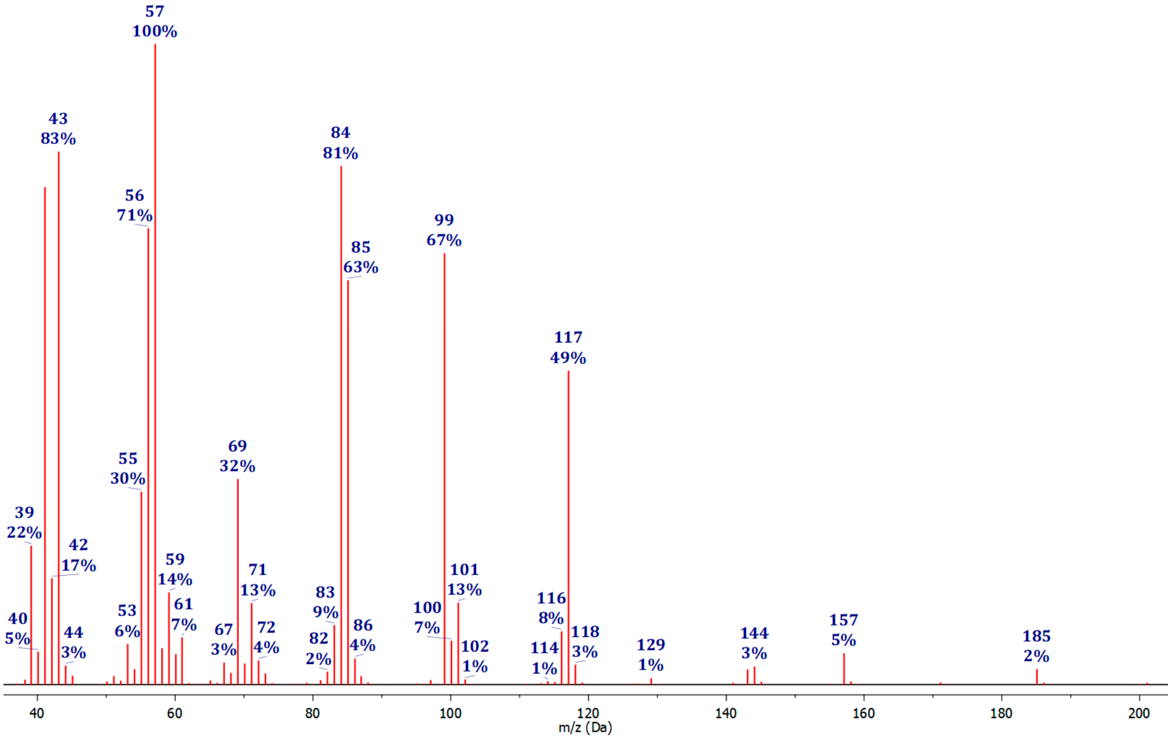

Figure S43. Mass spectrum of 4-methylpentyl 3,3-dimethylbutanoate (**8c**)

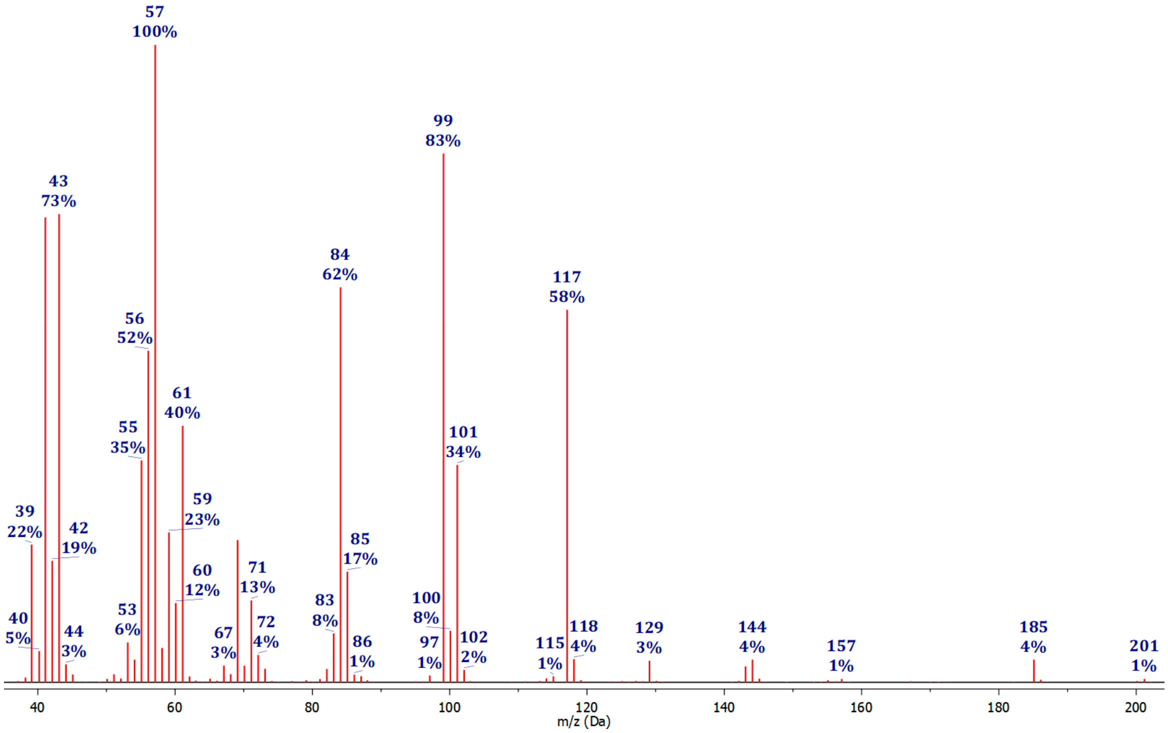

Figure S44. Mass spectrum of hexyl 3,3-dimethylbutanoate (**9c**)

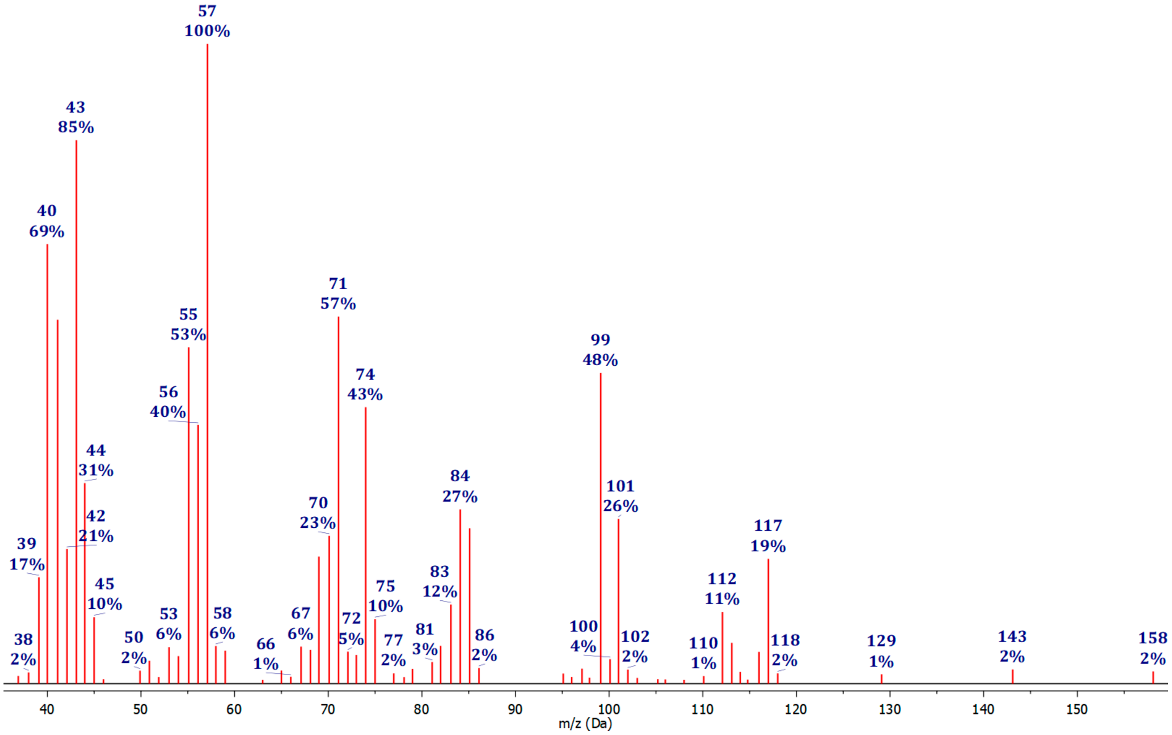

Figure S45. Mass spectrum of 1-methylpentyl 2,3-dimethylbutanoate (**5d**)

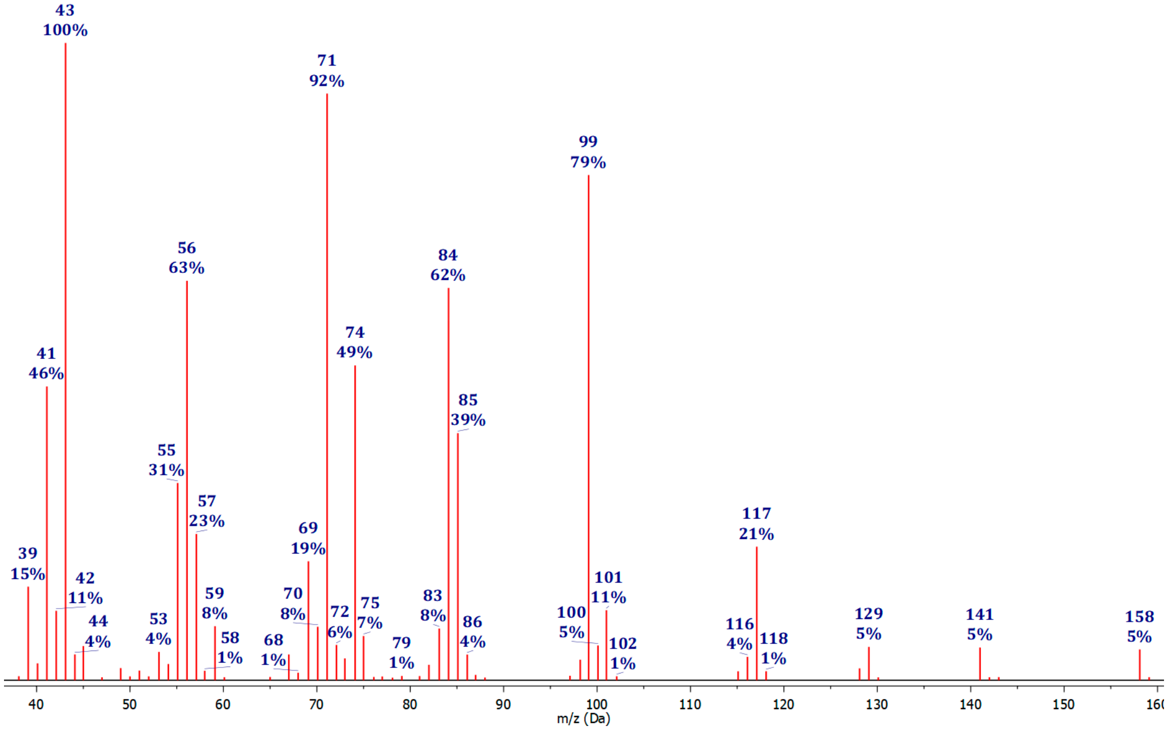

Figure S46. Mass spectrum of 2-methylpentyl 2,3-dimethylbutanoate (**6d**)

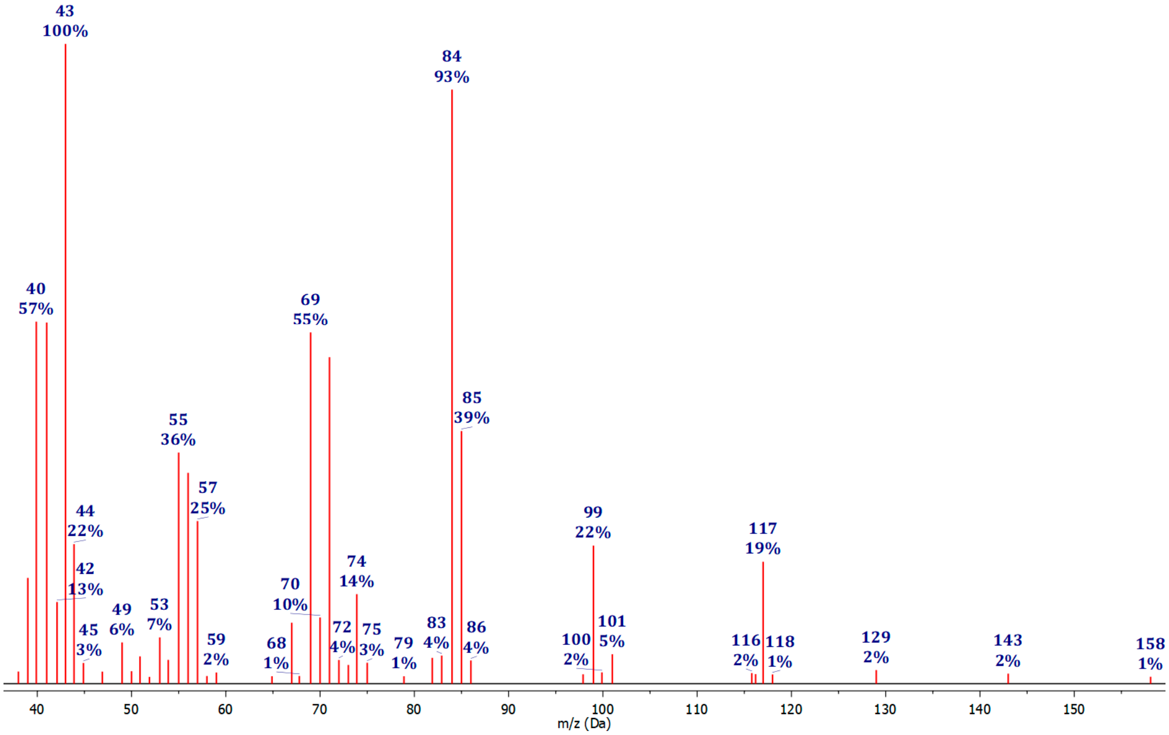

Figure S47. Mass spectrum of 3-methylpentyl 2,3-dimethylbutanoate (**7d**)

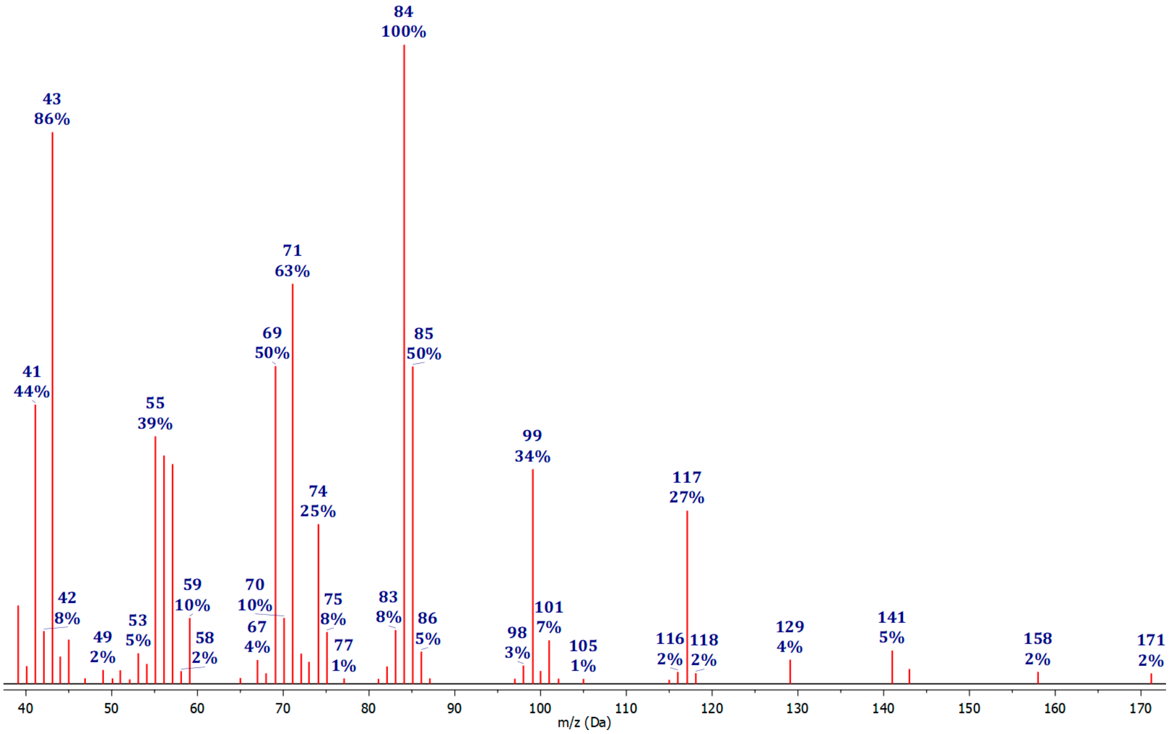

Figure S48. Mass spectrum of 4-methylpentyl 2,3-dimethylbutanoate (**8d**)

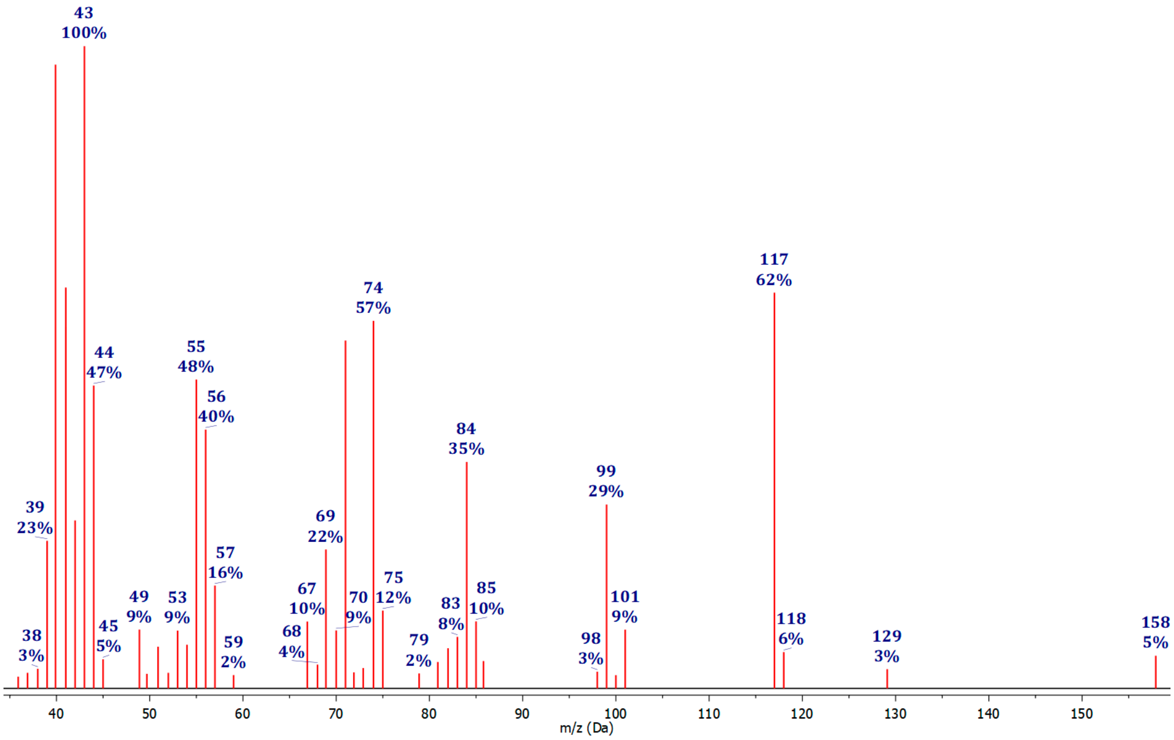

Figure S49. Mass spectrum of hexyl 2,3-dimethylbutanoate (**9d**)

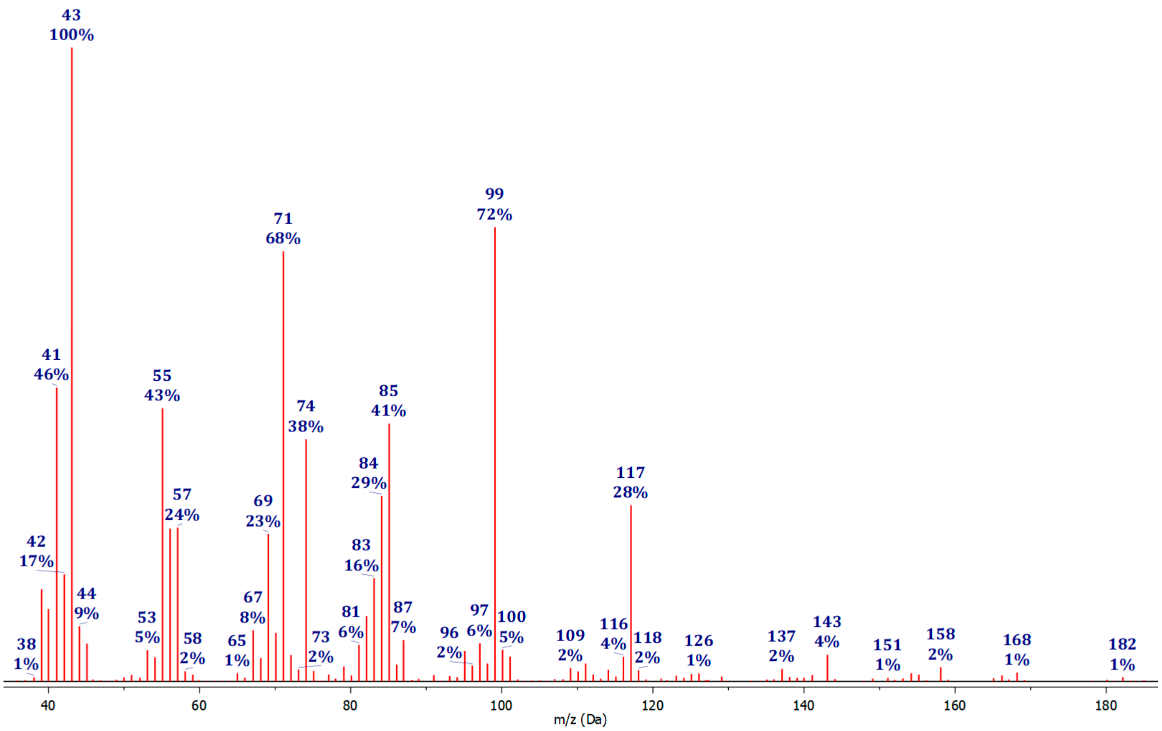

Figure S50a. Mass spectrum of 1-methylpentyl 2-methylpentanoate (**5e epimer I**)

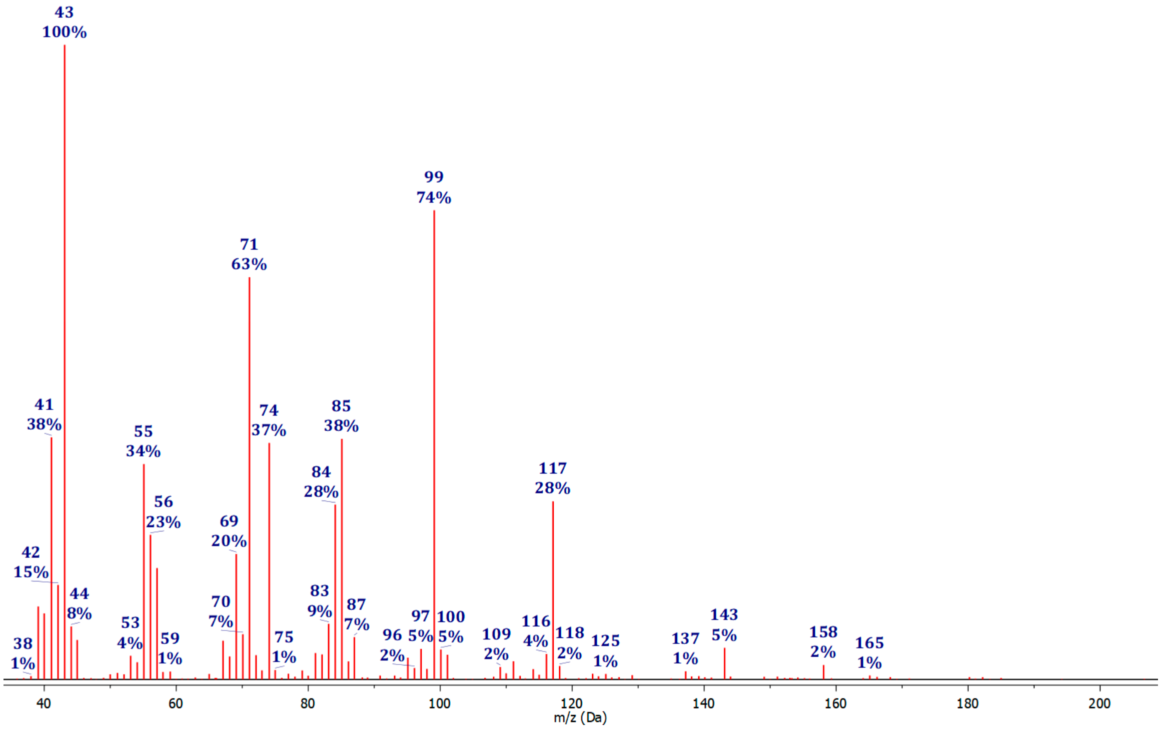

Figure S50b. Mass spectrum of 1-methylpentyl 2-methylpentanoate (**5e epimer II**)

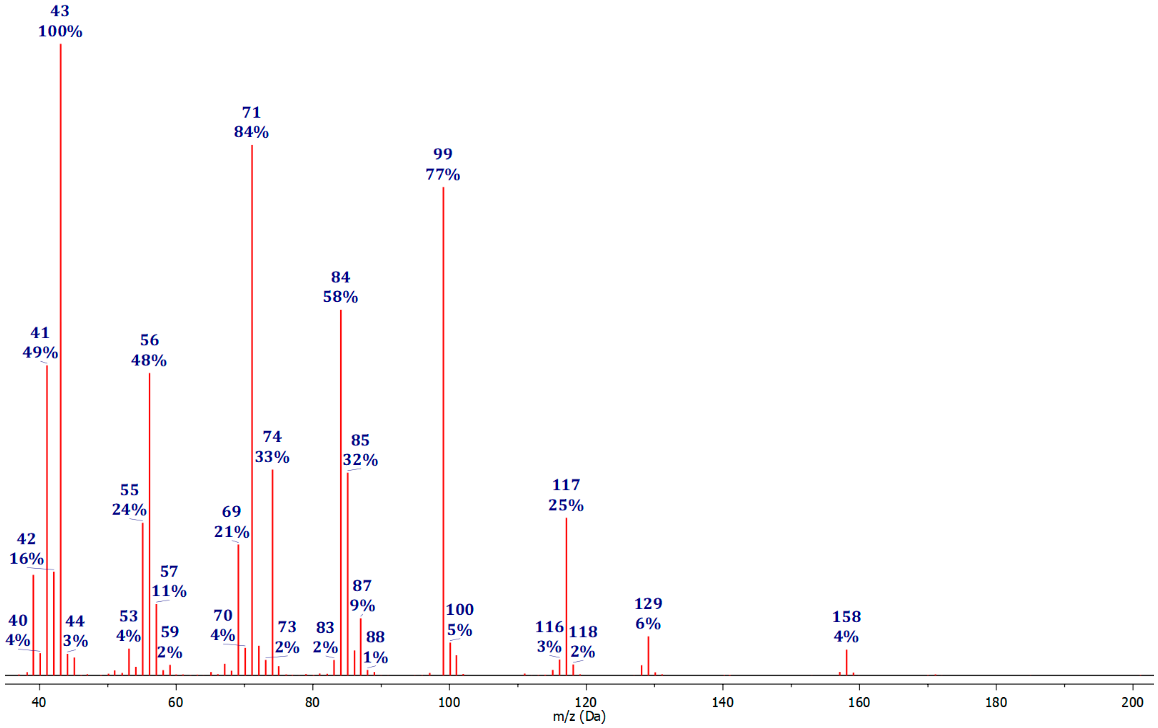

Figure S51. Mass spectrum of 2-methylpentyl 2-methylpentanoate (**6e**)

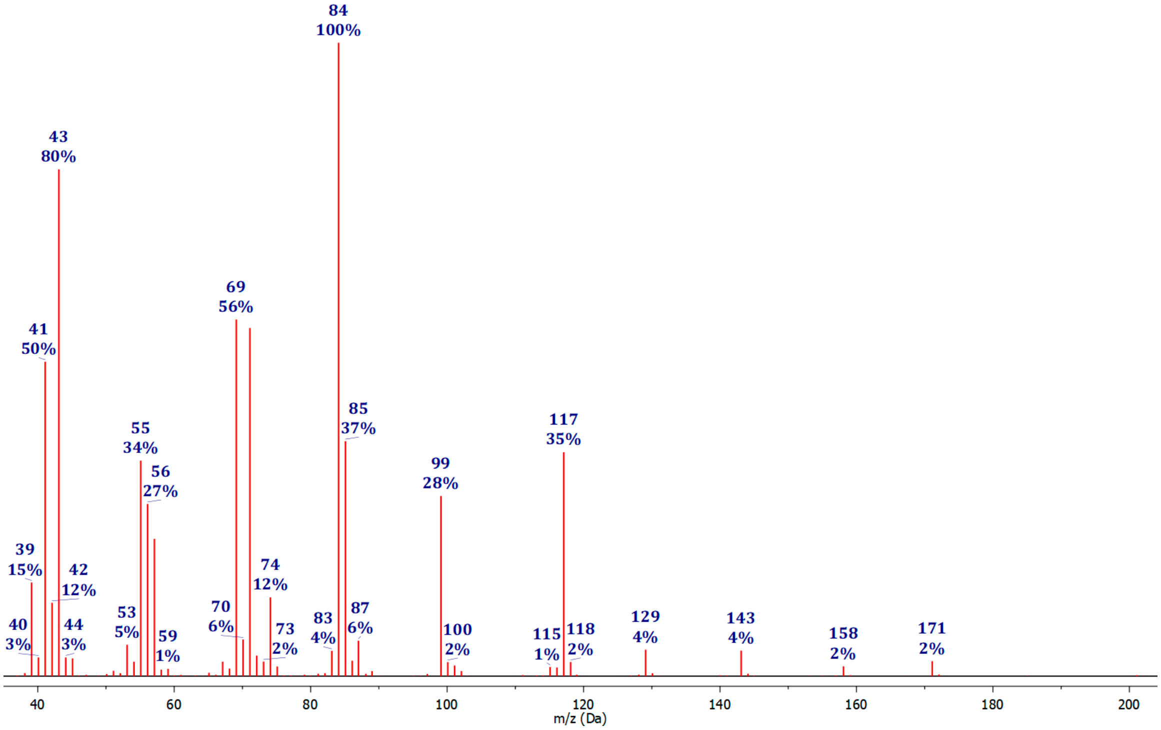

Figure S52. Mass spectrum of 3-methylpentyl 2-methylpentanoate (**7e**)

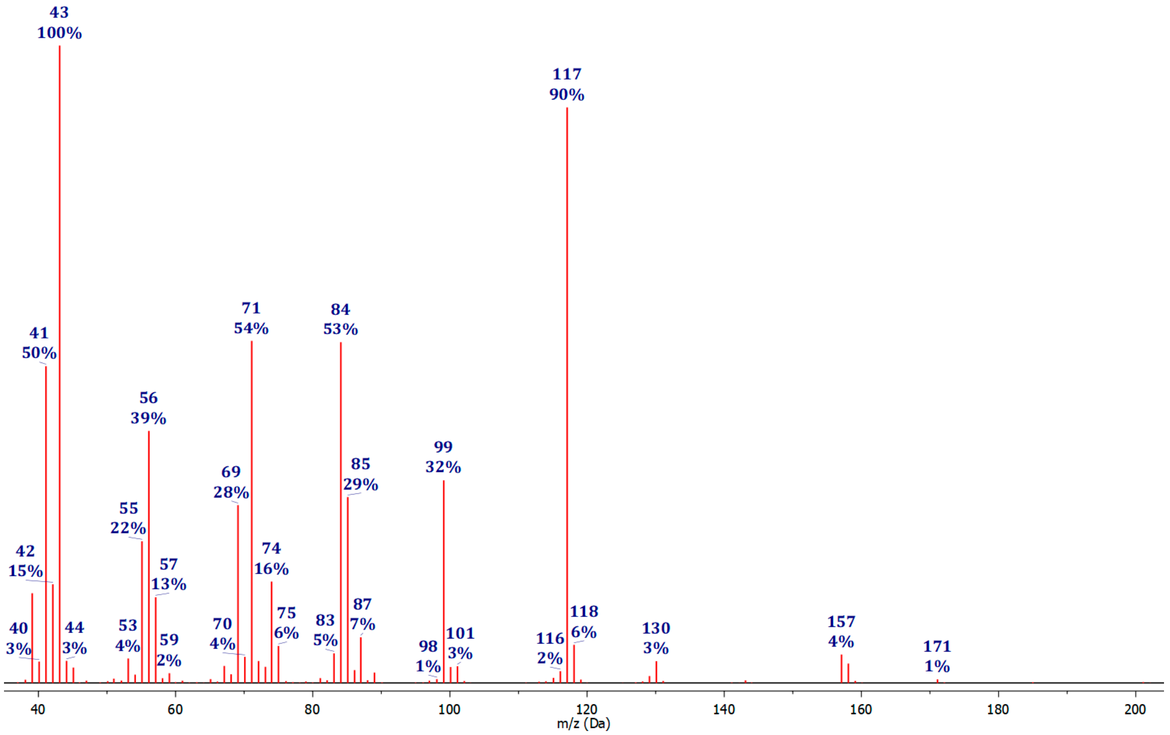

Figure S53. Mass spectrum of 4-methylpentyl 2-methylpentanoate (**8e**)

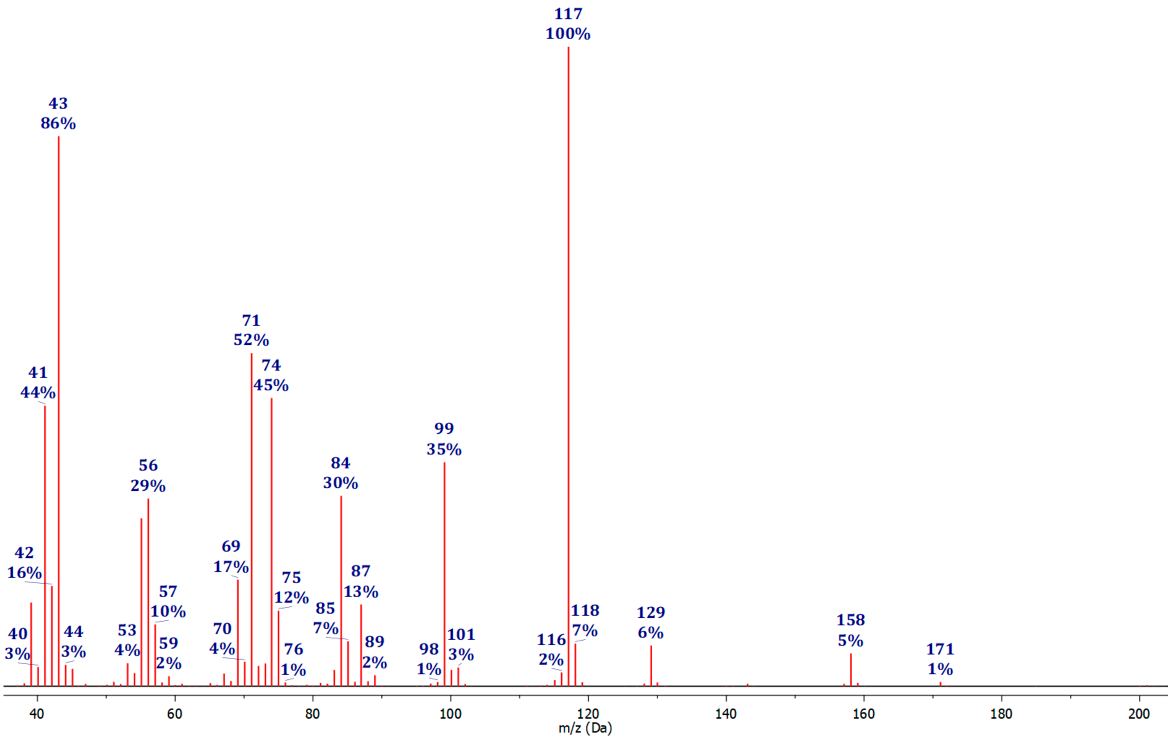

Figure S54. Mass spectrum of hexyl 2-methylpentanoate (**9e**)

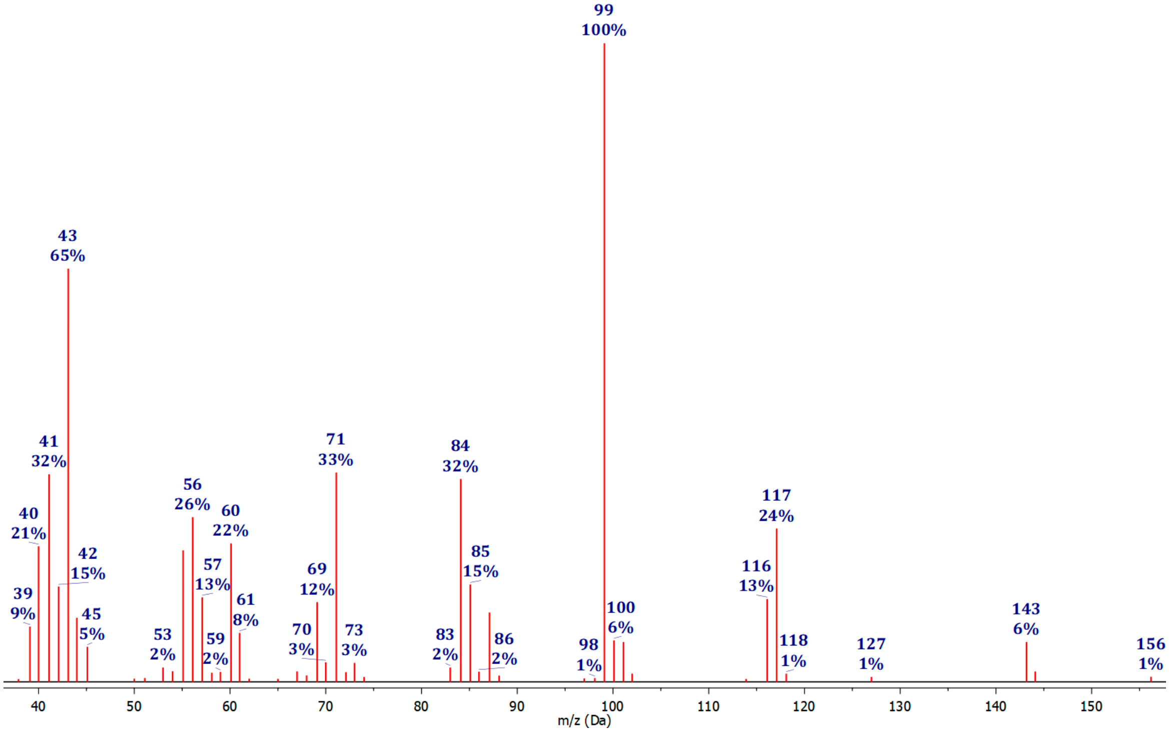

Figure S55. Mass spectrum of 1-methylpentyl 3-methylpentanoate (**5f**)

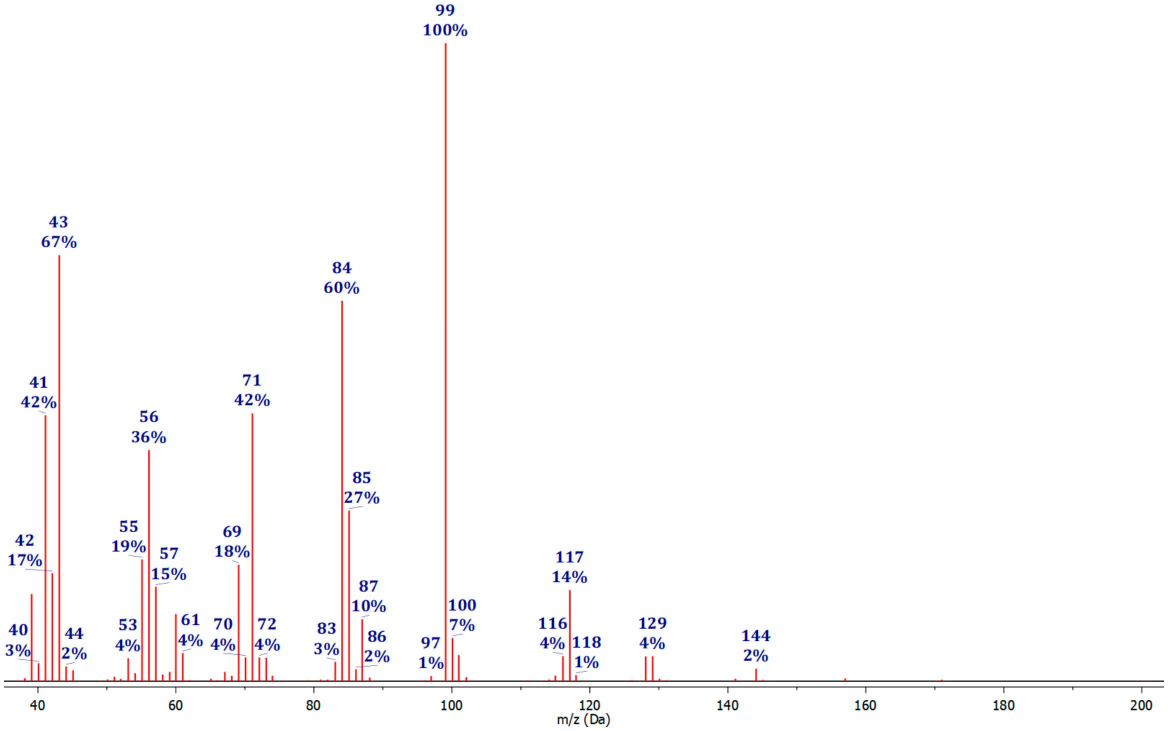

Figure S56. Mass spectrum of 2-methylpentyl 3-methylpentanoate (**6f**)

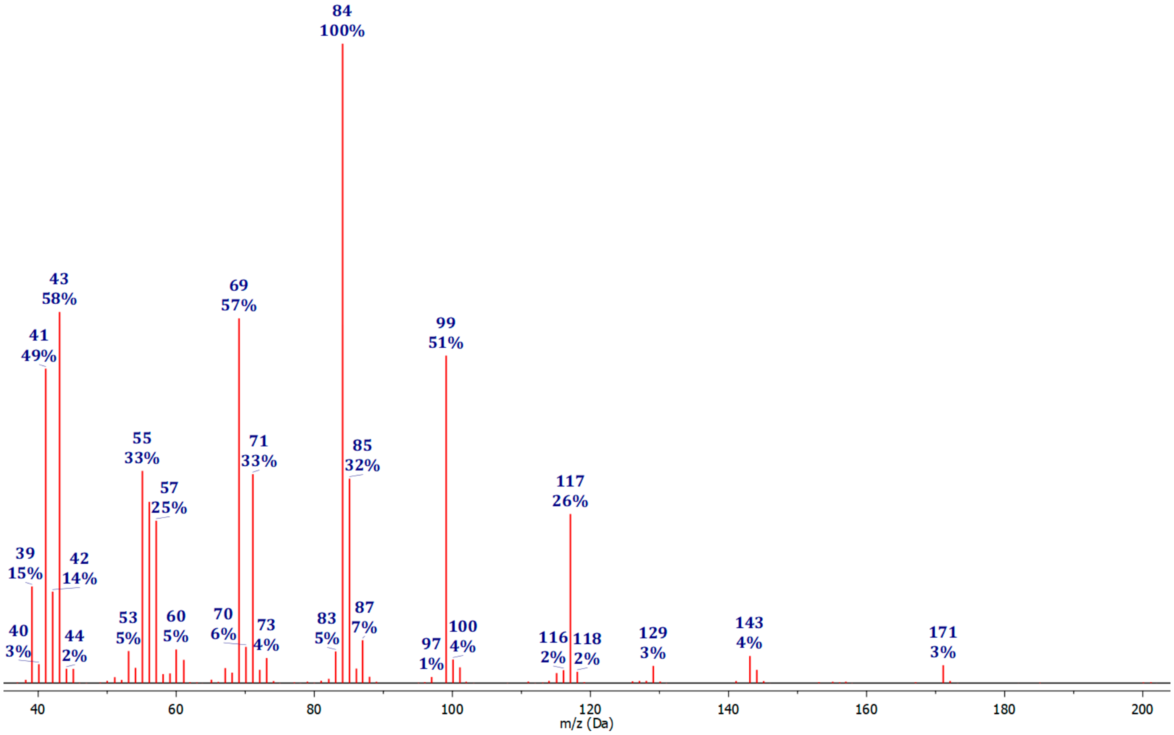

Figure S57. Mass spectrum of 3-methylpentyl 3-methylpentanoate (**7f**)

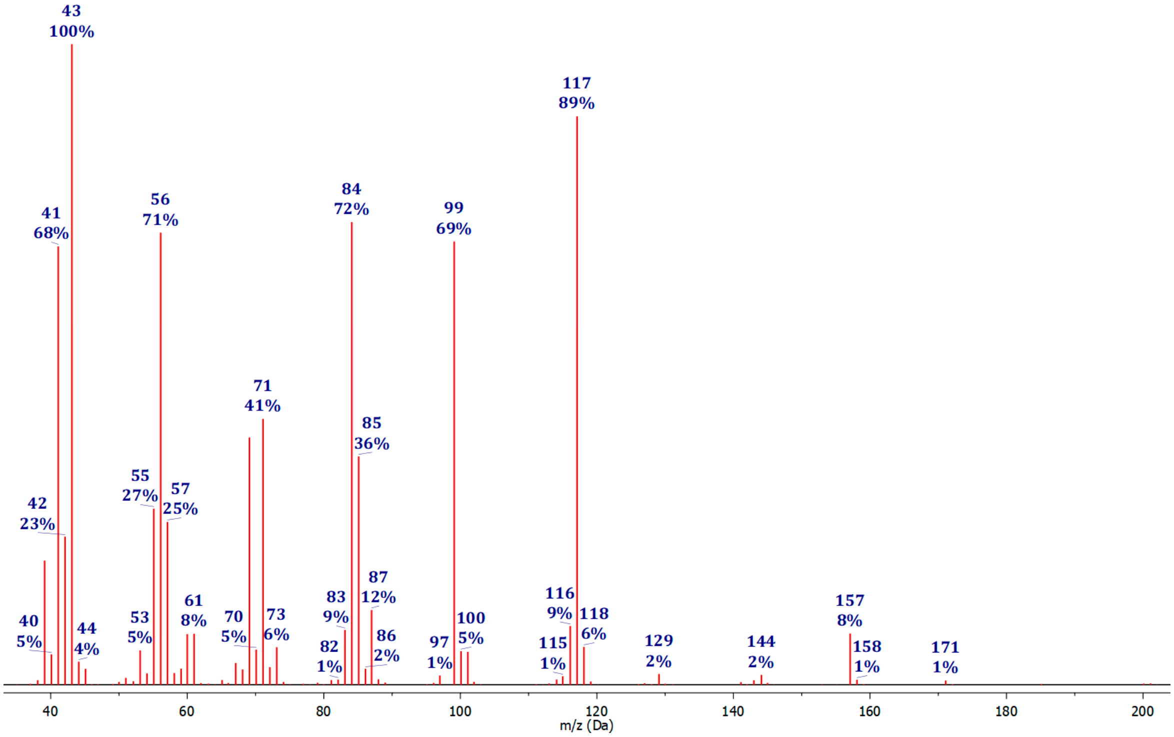

Figure S58. Mass spectrum of 4-methylpentyl 3-methylpentanoate (**8f**)

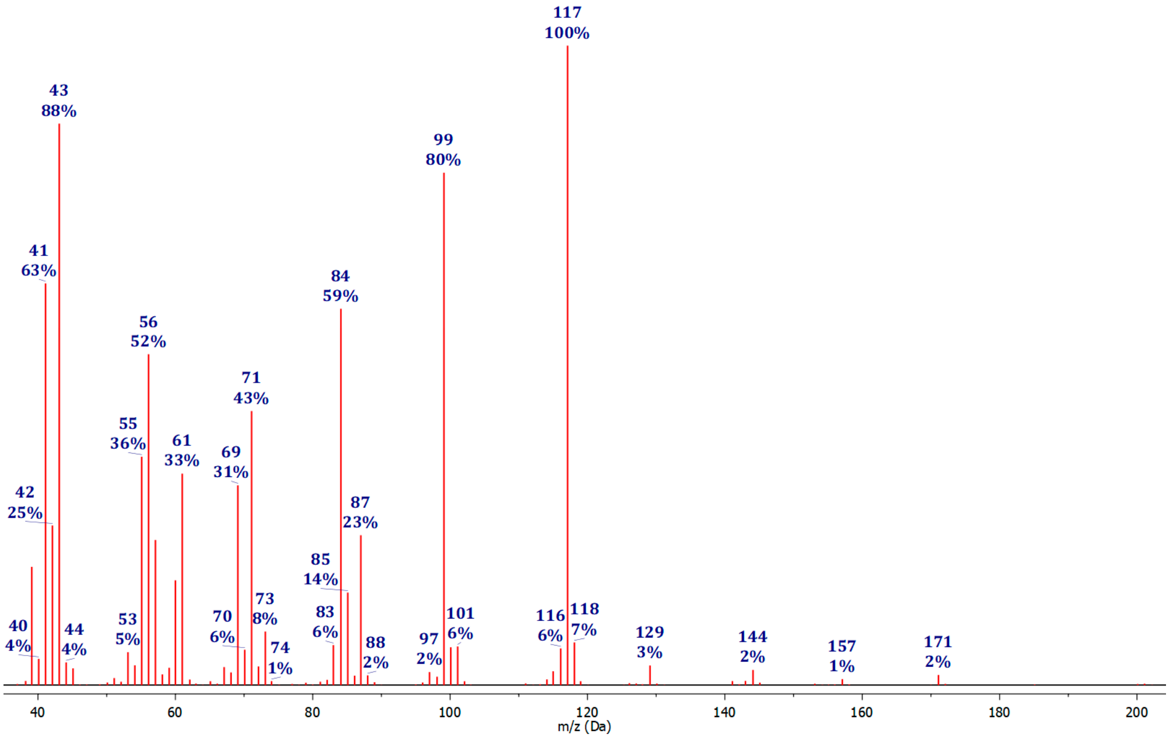

Figure S59. Mass spectrum of hexyl 3-methylpentanoate (**9f**)

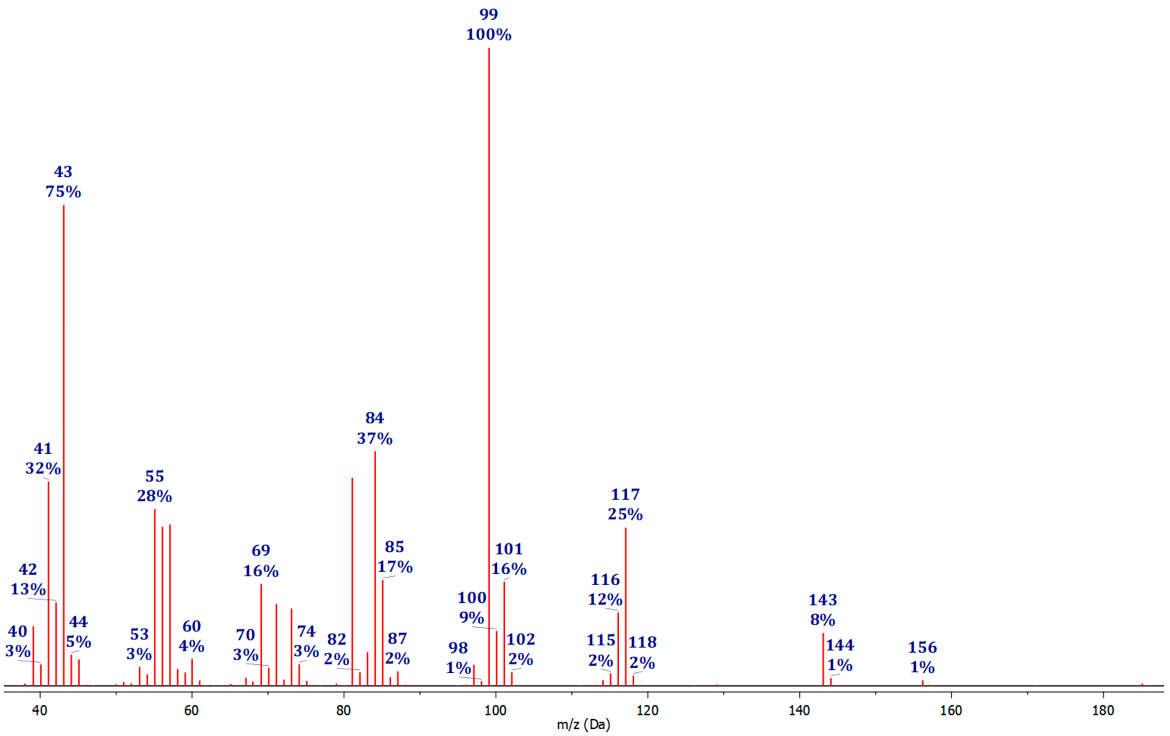

Figure S60. Mass spectrum of 1-methylpentyl 4-methylpentanoate (**5g**)

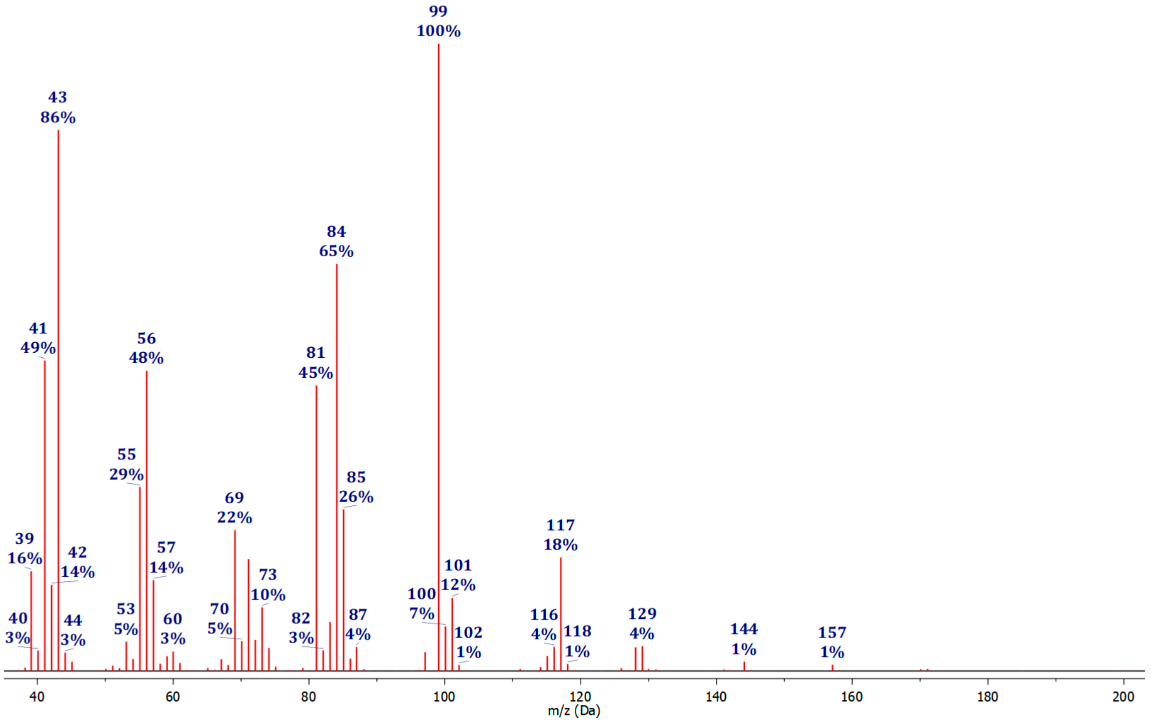

Figure S61. Mass spectrum of 2-methylpentyl 4-methylpentanoate (**6g**)

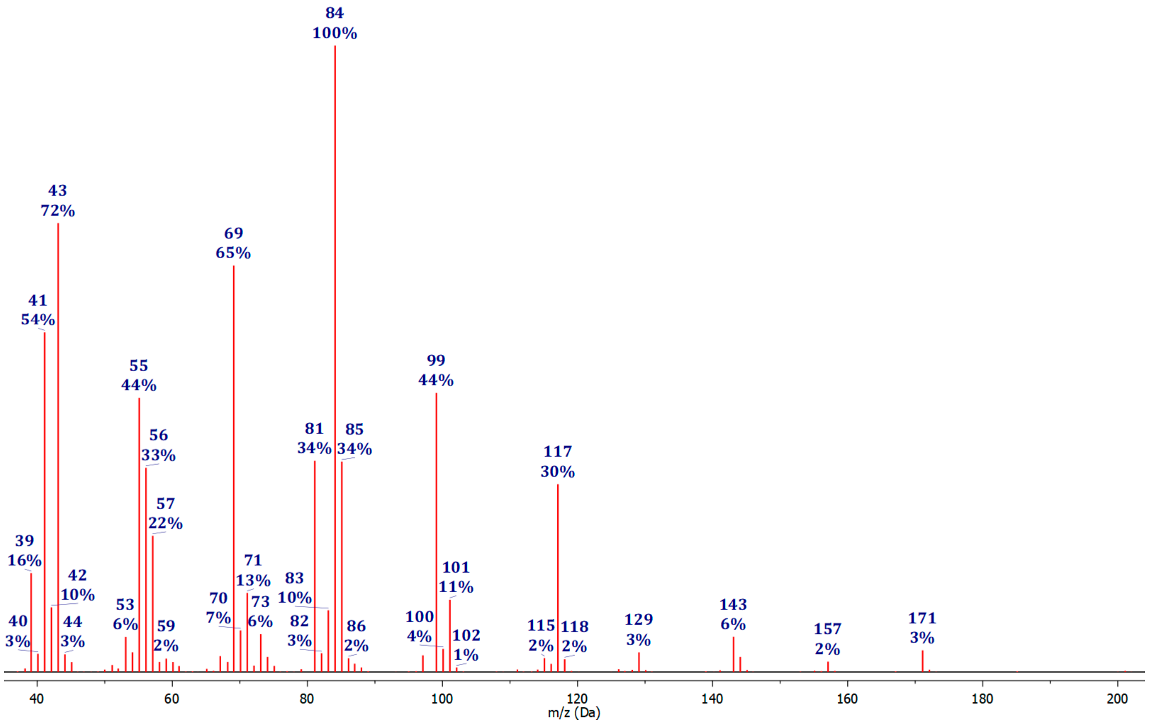

Figure S62a. Mass spectrum of 3-methylpentyl 4-methylpentanoate (**7g**)

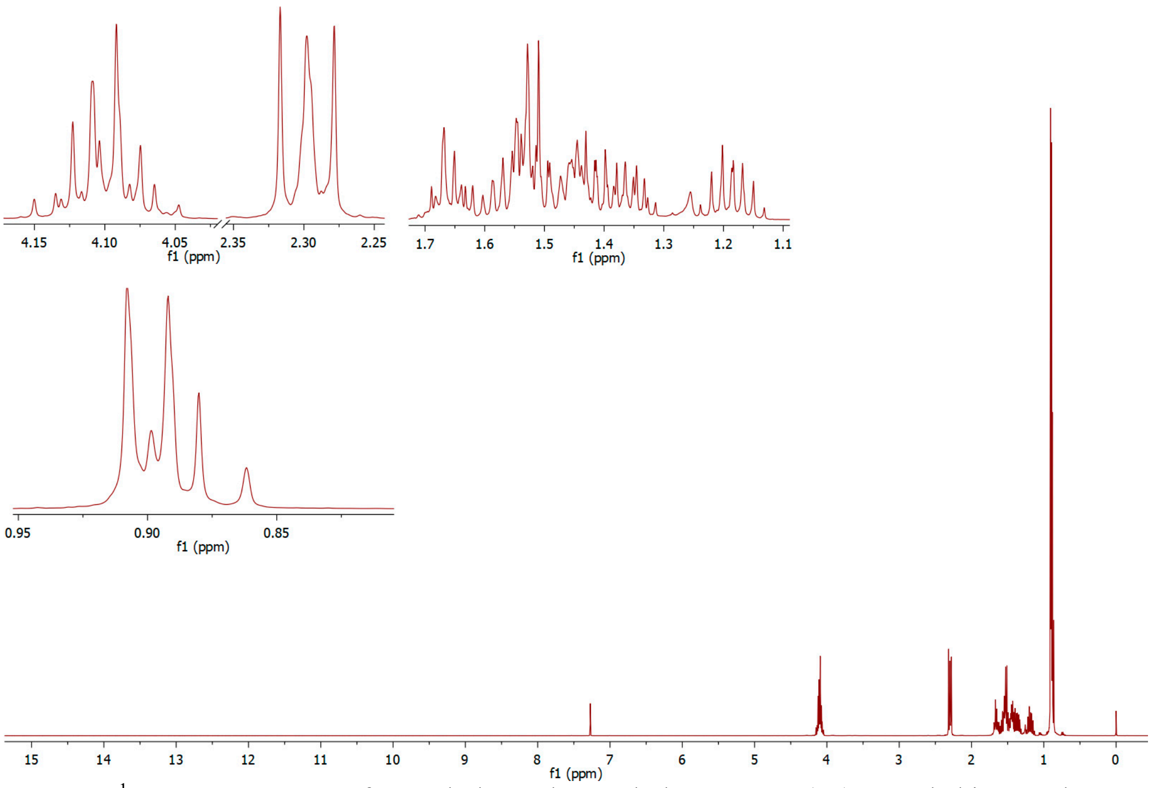

Figure S62b. ^{1}H NMR spectrum of 3-methylpentyl 4-methylpentanoate (**7g**) recorded in $CDCl_3$

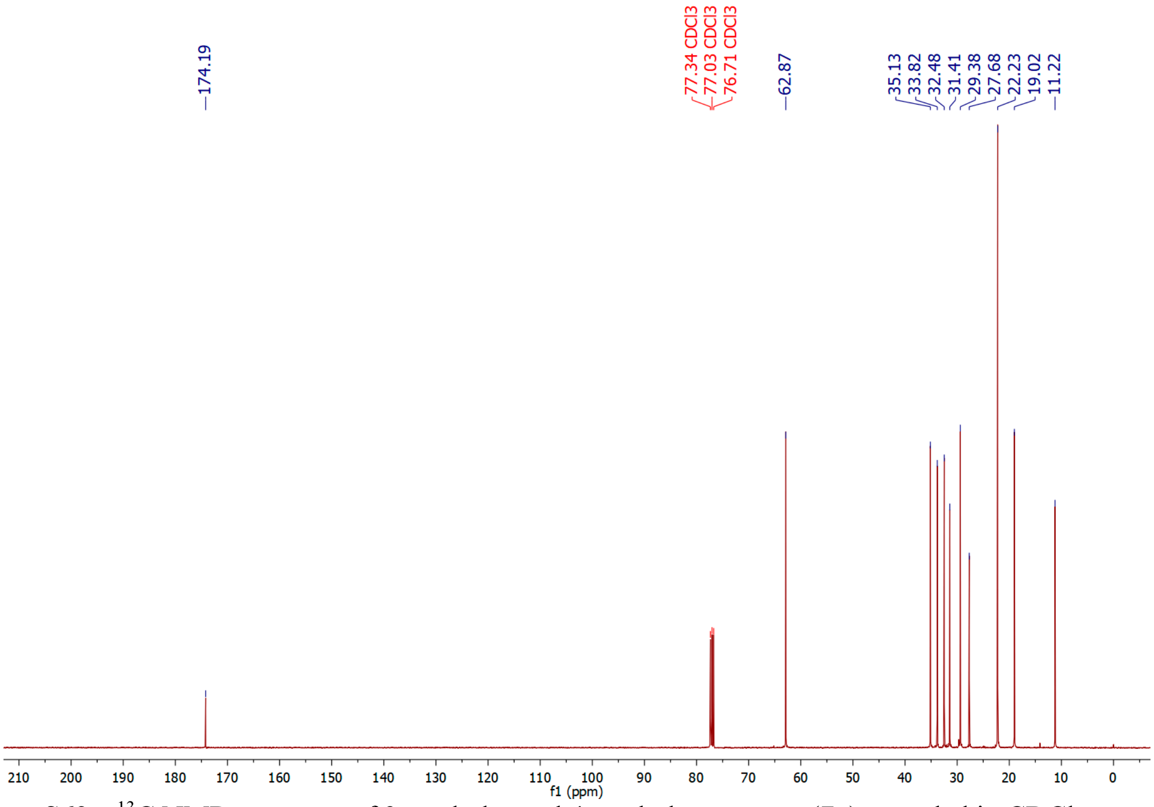

Figure S62c. ^{13}C NMR spectrum of 3-methylpentyl 4-methylpentanoate (**7g**) recorded in $CDCl_3$

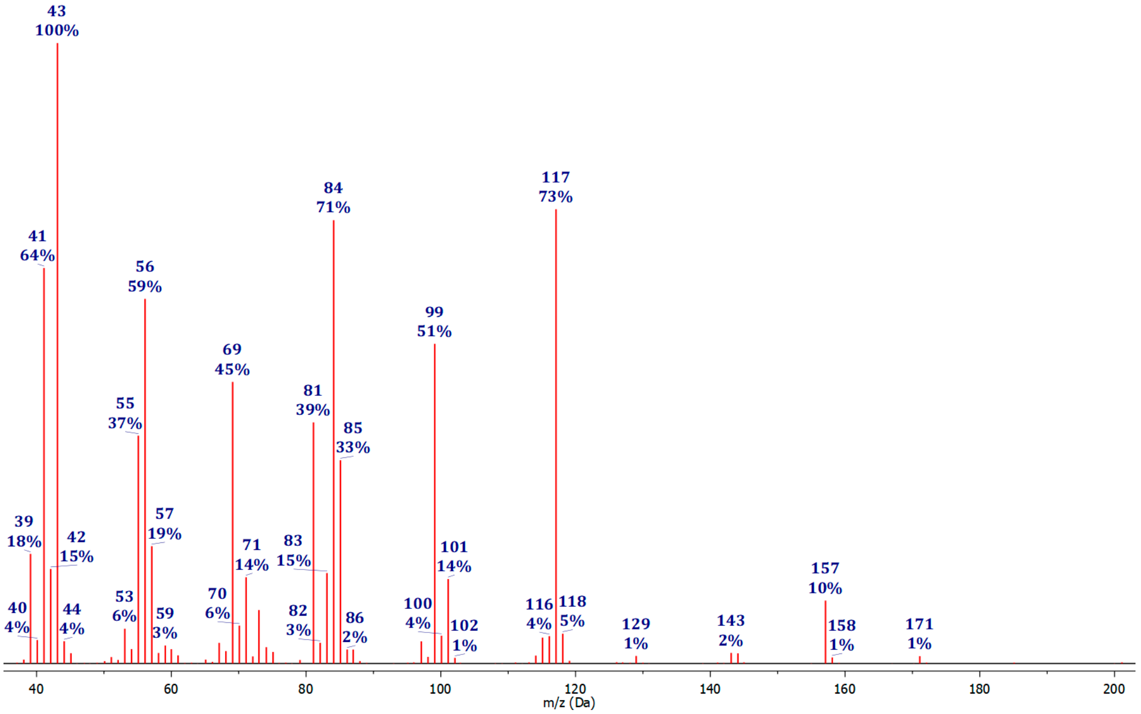

Figure S63. Mass spectrum of 4-methylpentyl 4-methylpentanoate (**8g**)

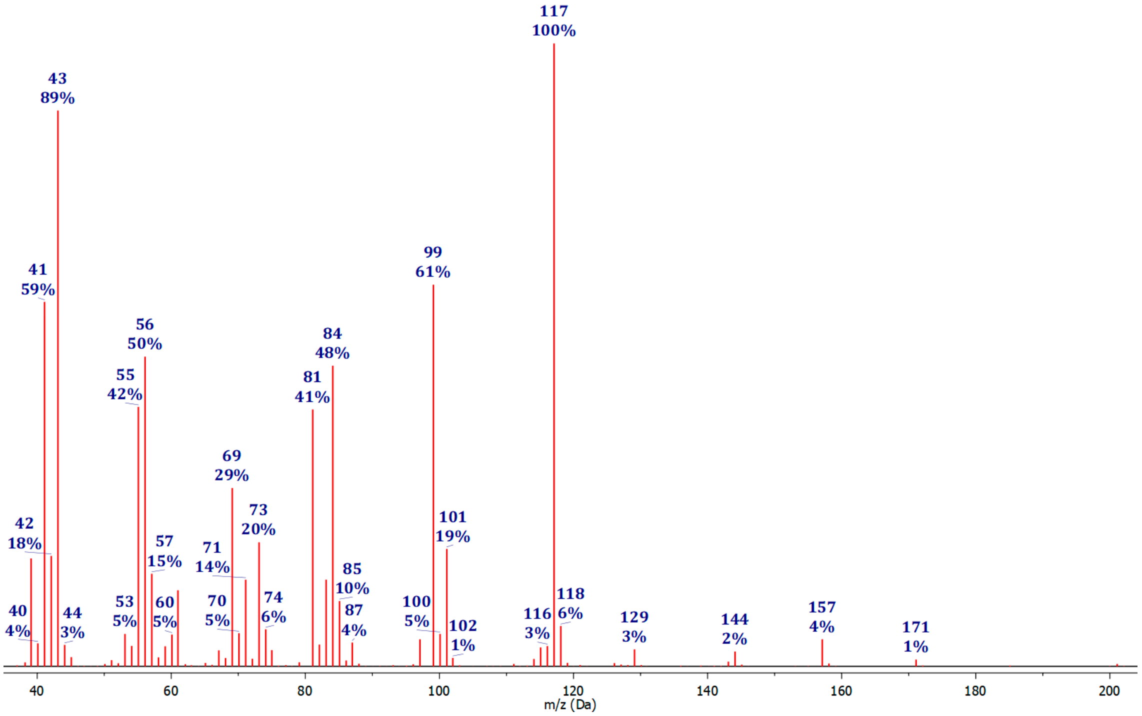

Figure S64. Mass spectrum of hexyl 4-methylpentanoate (**9g**)

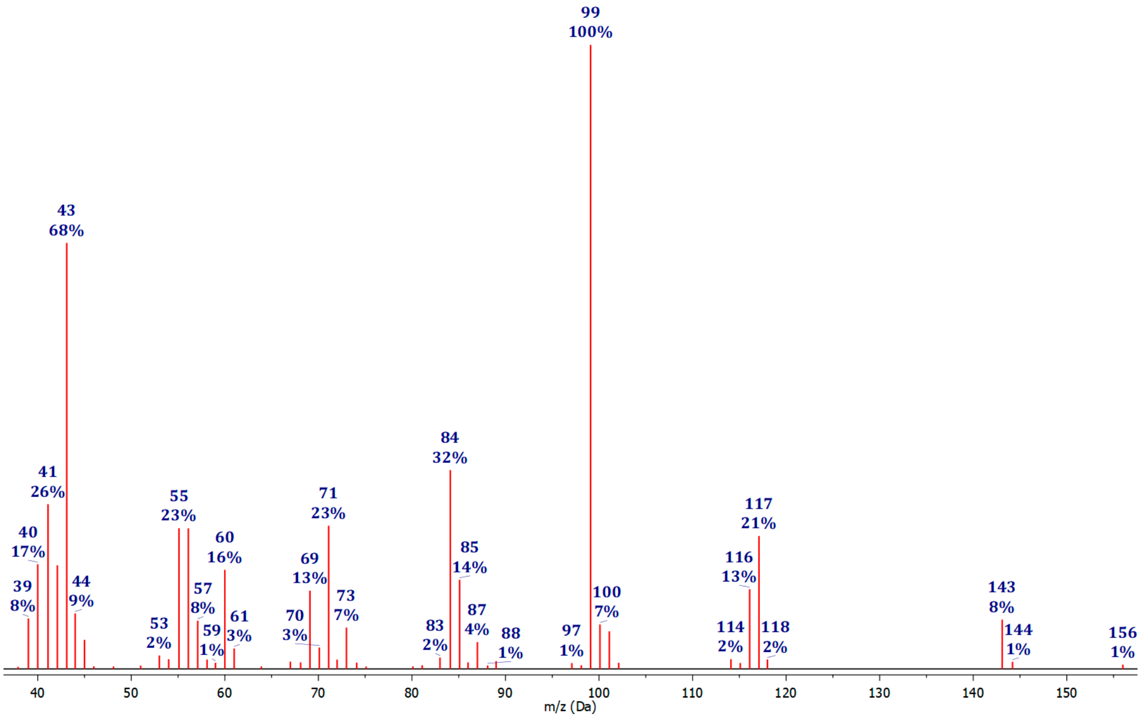

Figure S65. Mass spectrum of 1-methylpentyl hexanoate (**5h**)

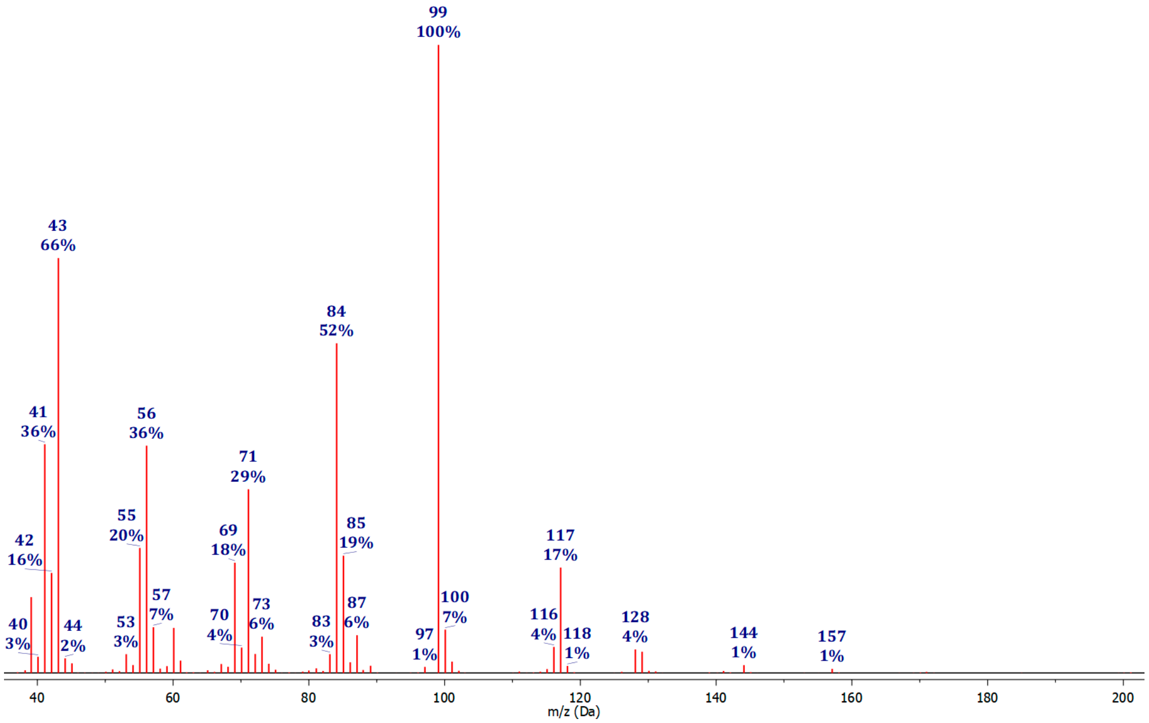

Figure S66. Mass spectrum of 2-methylpentyl hexanoate (**6h**)

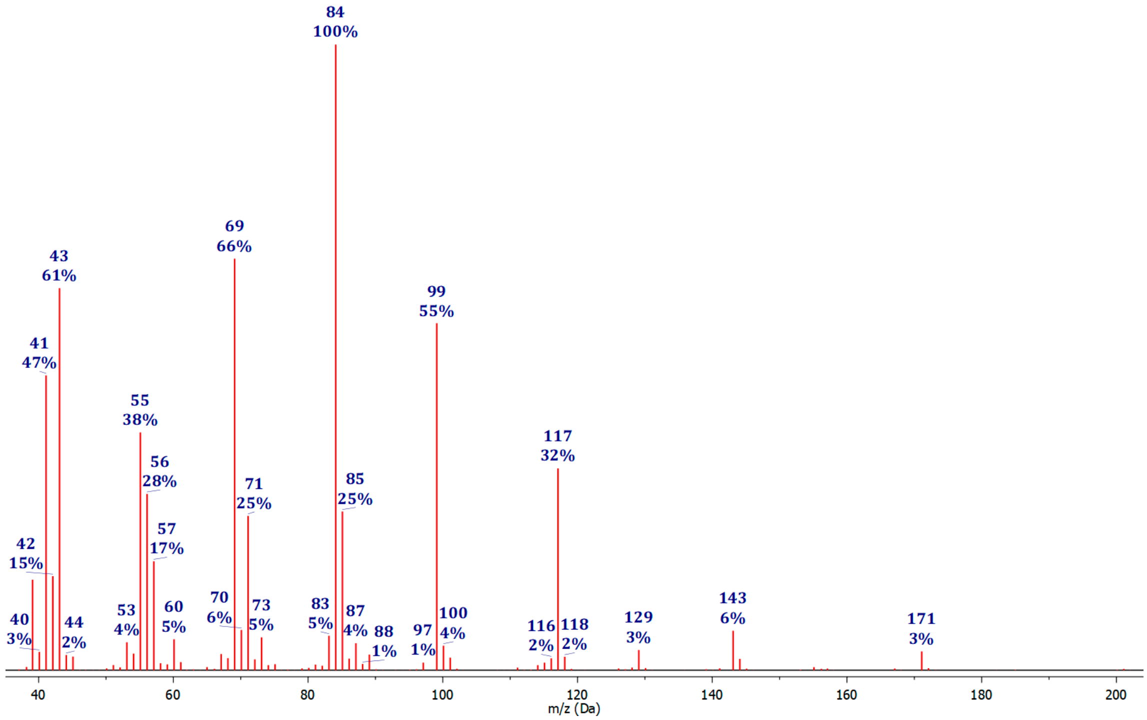

Figure S67. Mass spectrum of 3-methylpentyl hexanoate (**7h**)

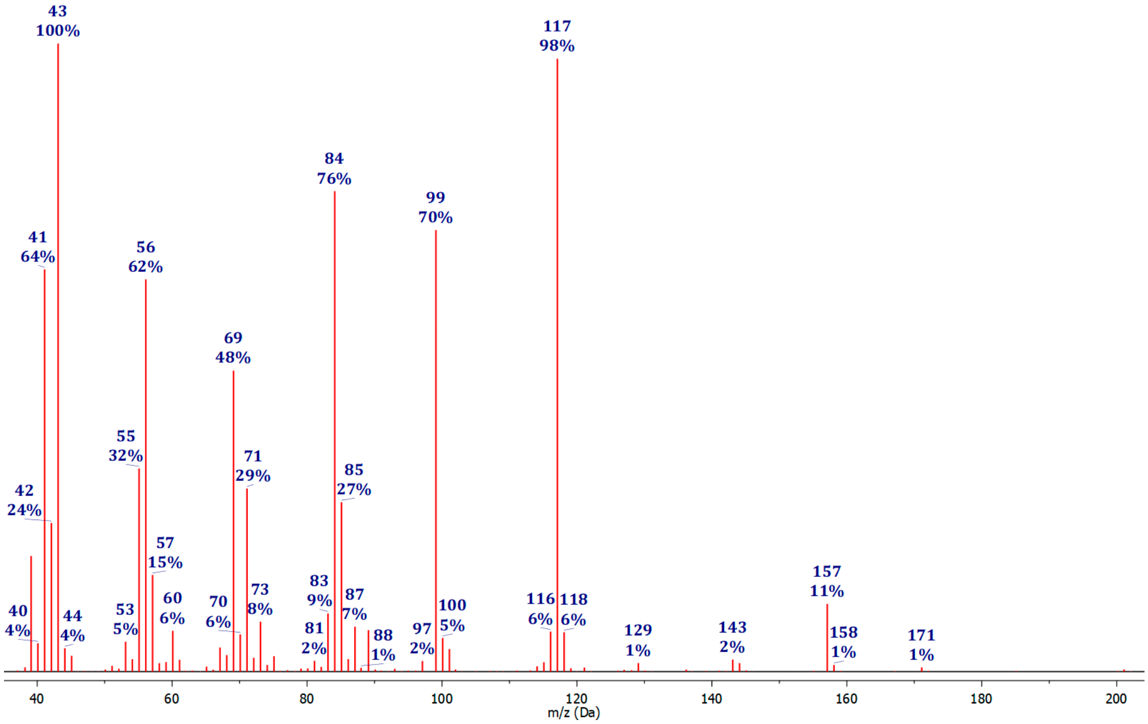

Figure S68. Mass spectrum of 4-methylpentyl hexanoate (**8h**)

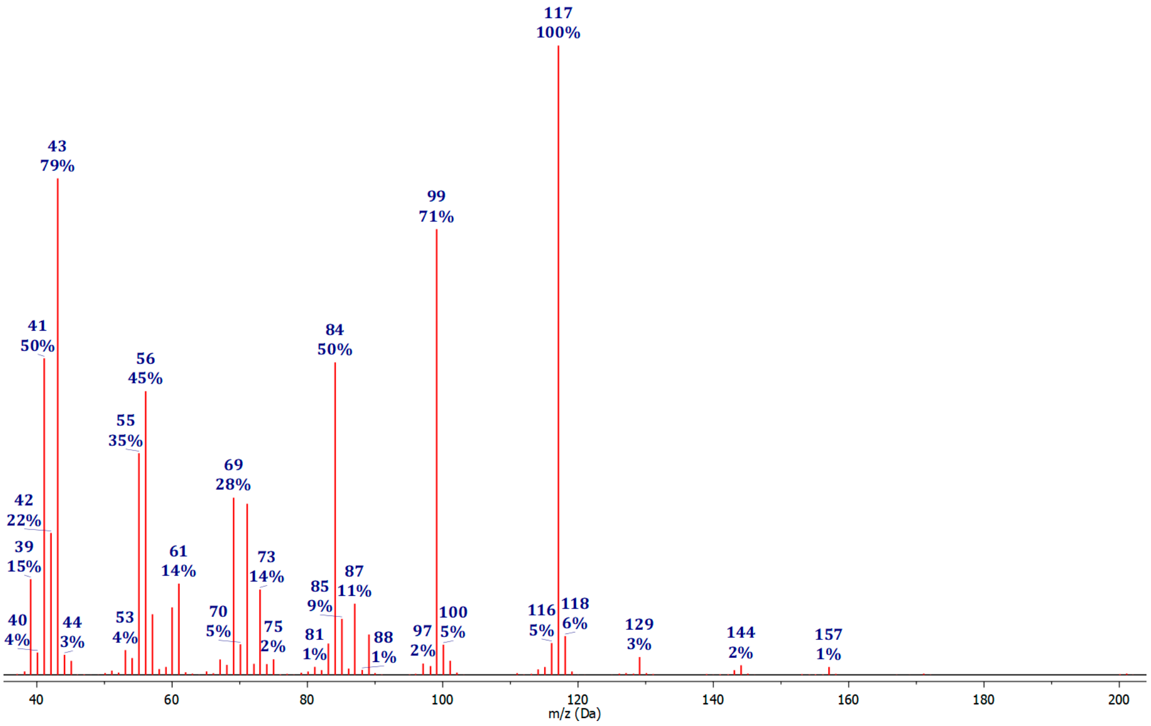

Figure S69. Mass spectrum of hexyl hexanoate (**9h**)

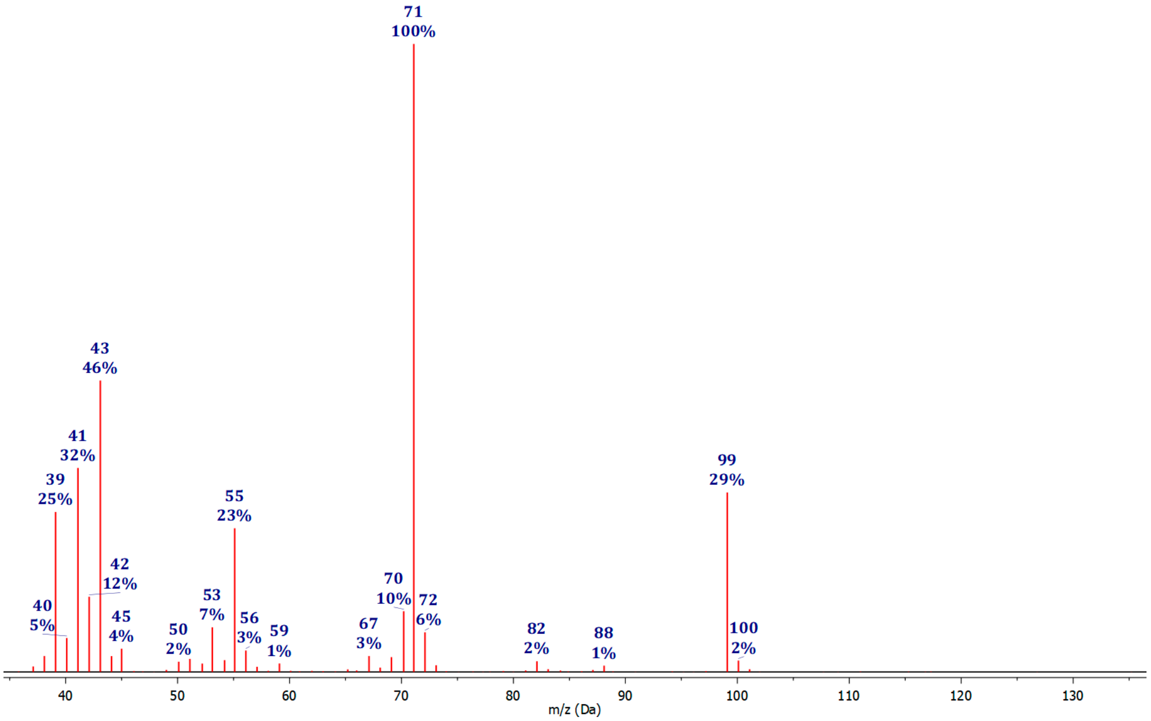

Figure S70. Mass spectrum of (*Z*)-hex-3-en-1-yl 2,2-dimethylbutanoate *(***10b**)

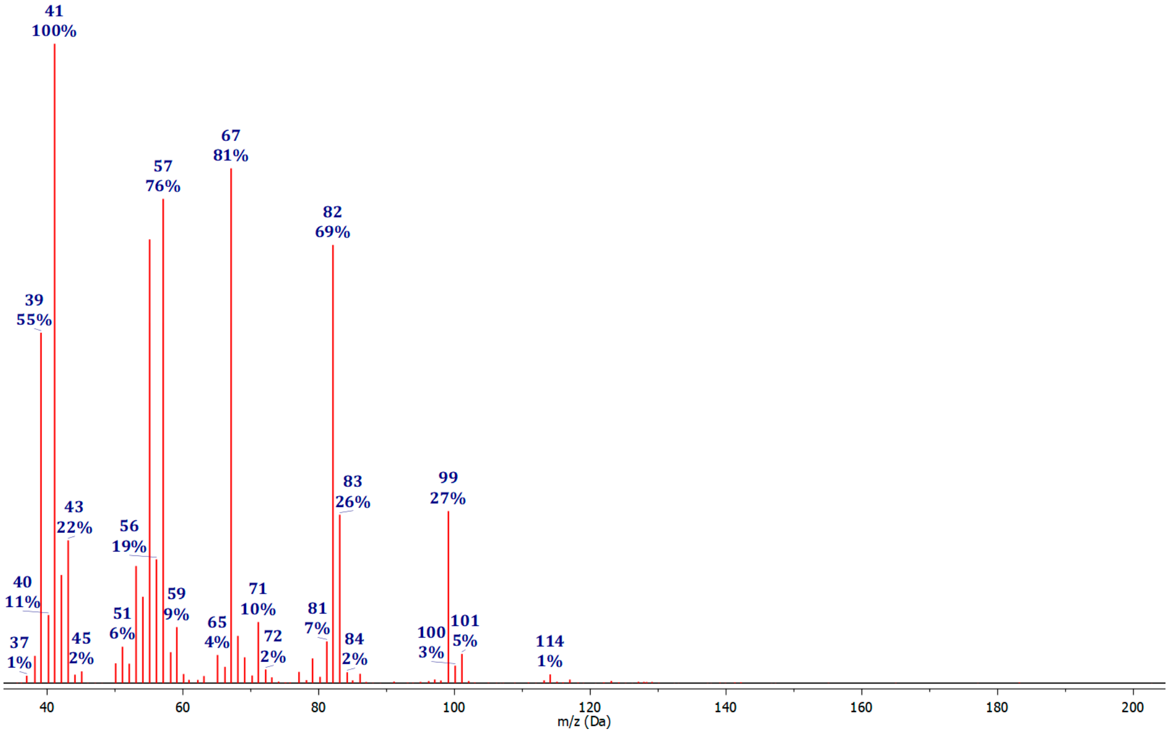

Figure S71. Mass spectrum of (*Z*)-hex-3-en-1-yl 3,3-dimethylbutanoate (**10c**)

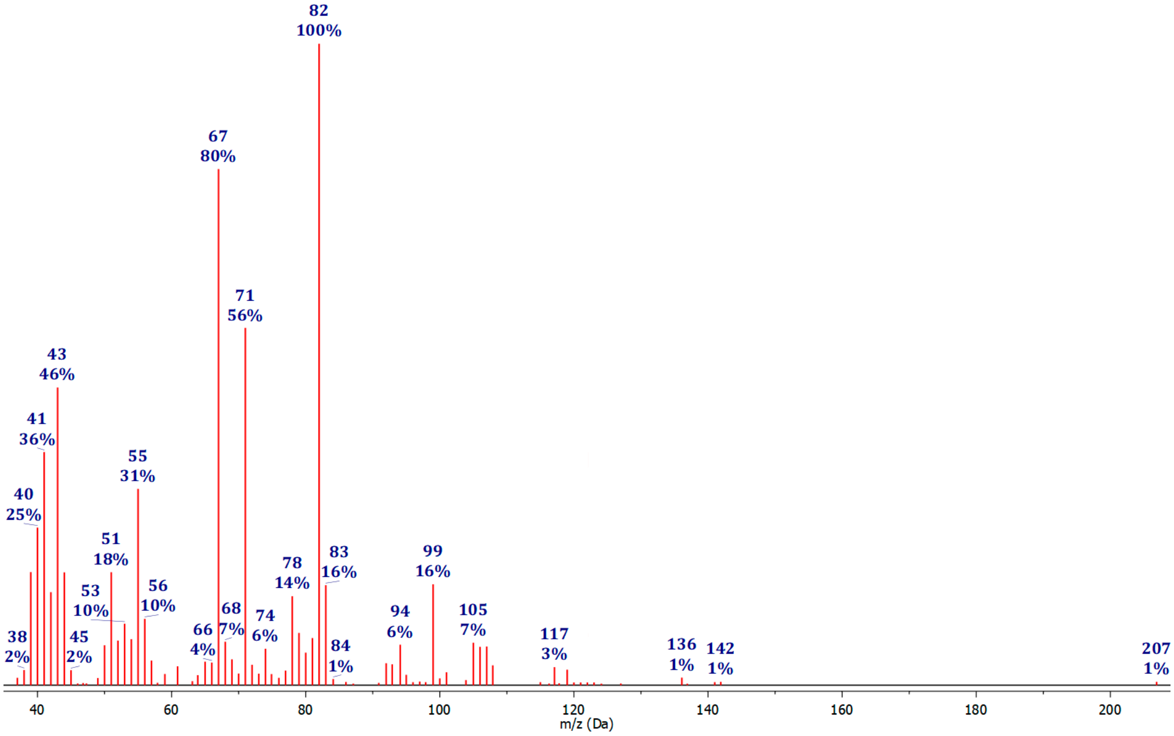

Figure S72. Mass spectrum of (*Z*)-hex-3-en-1-yl 2,3-dimethylbutanoate (**10d**)

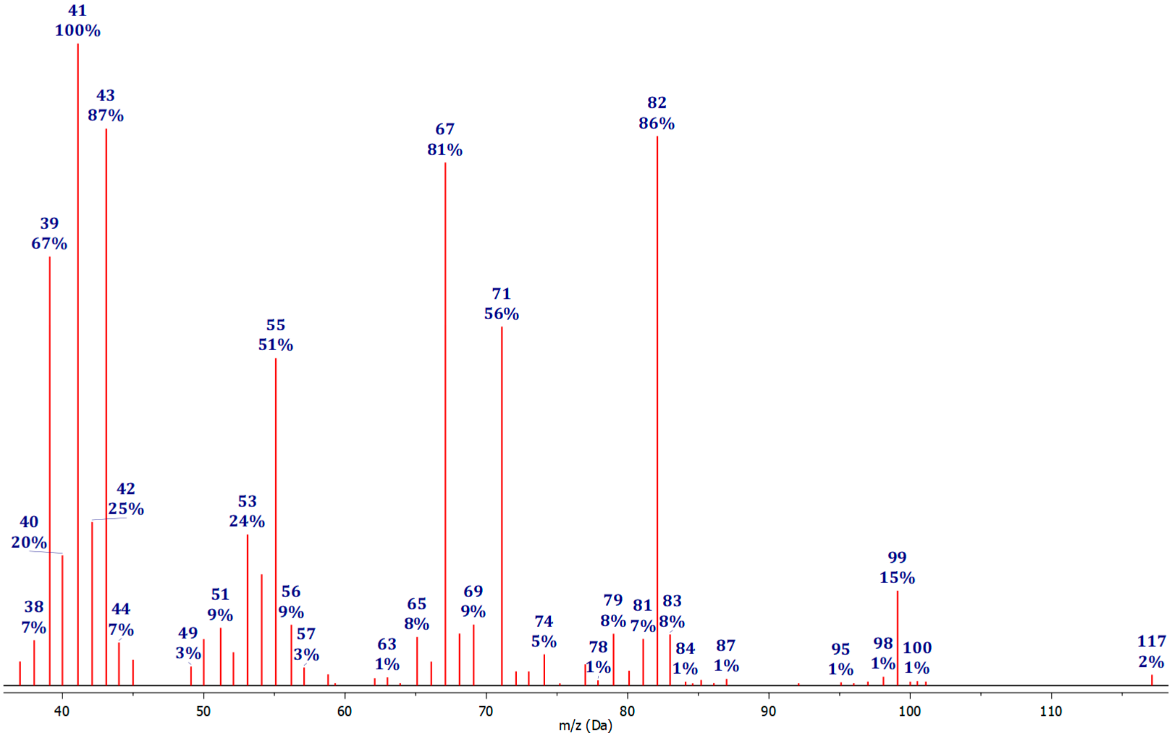

Figure S73. Mass spectrum of (*Z*)-hex-3-en-1-yl 2-methylpentanoate *(***10e**)

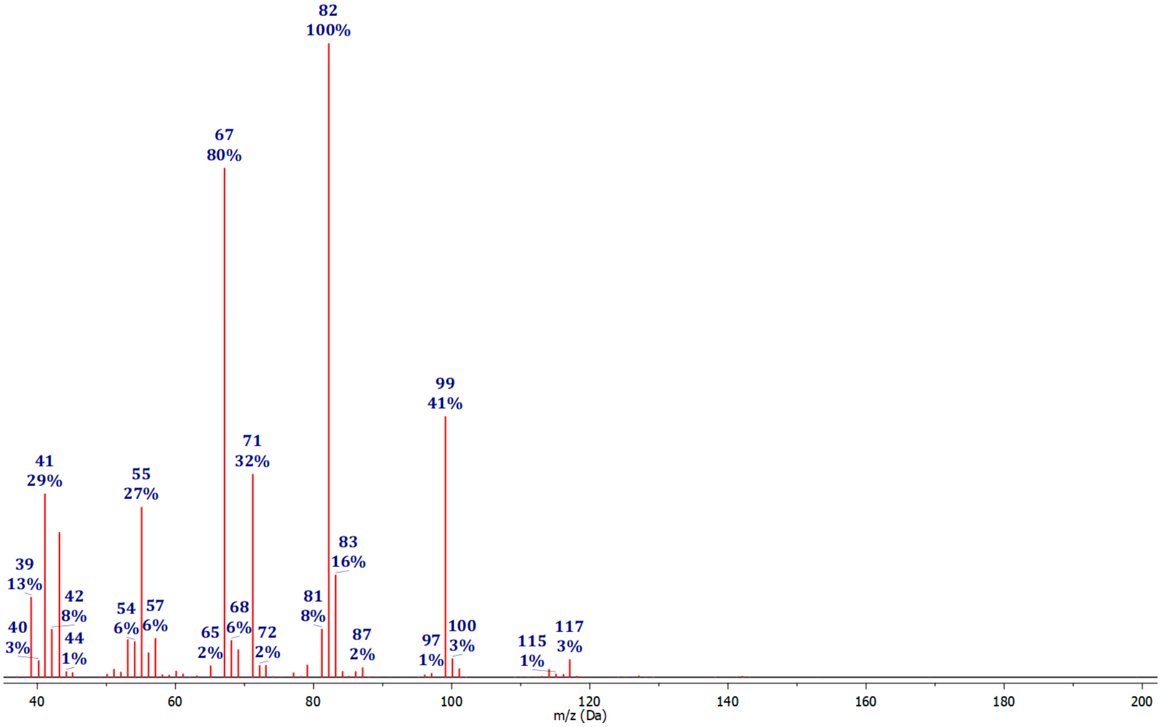

Figure S74a. Mass spectrum of (*Z*)-hex-3-en-1-yl 3-methylpentanoate *(***10f**)

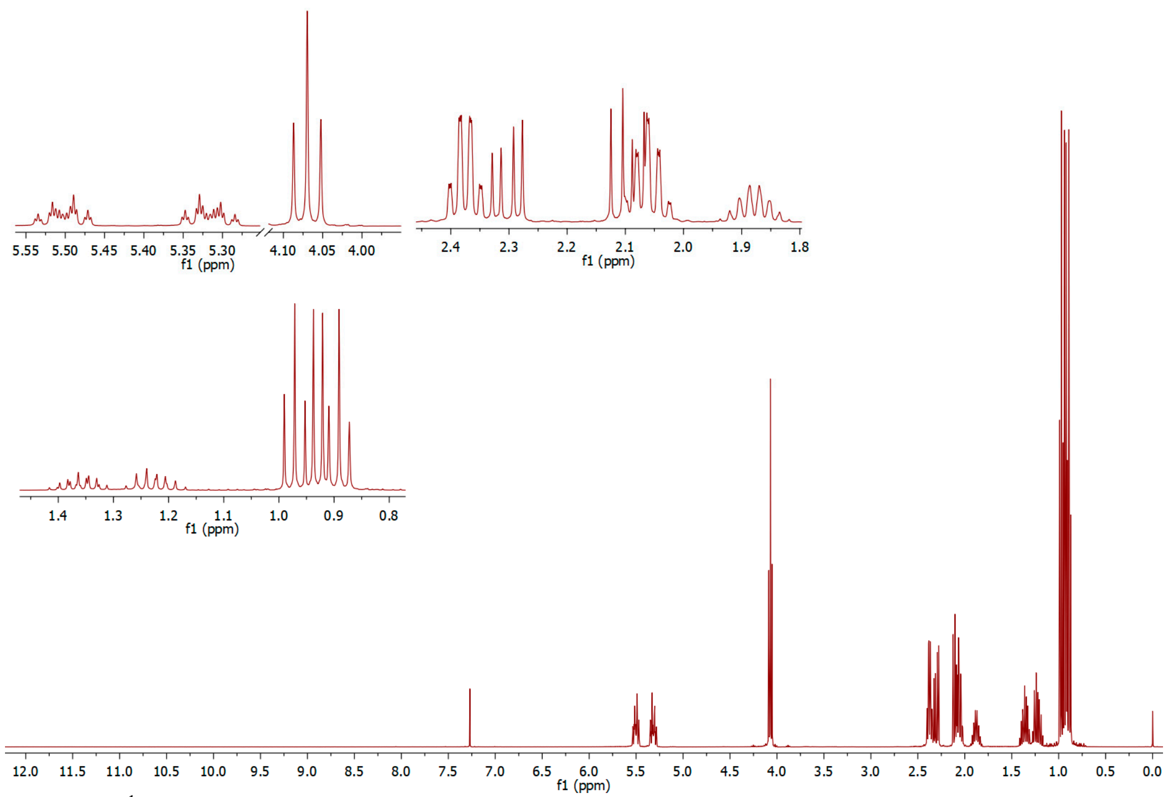

Figure S74b. ^{1}H NMR spectrum of (*Z*)-hex-3-en-1-yl 3-methylpentanoate *(***10f**) recorded in $CDCl_3$

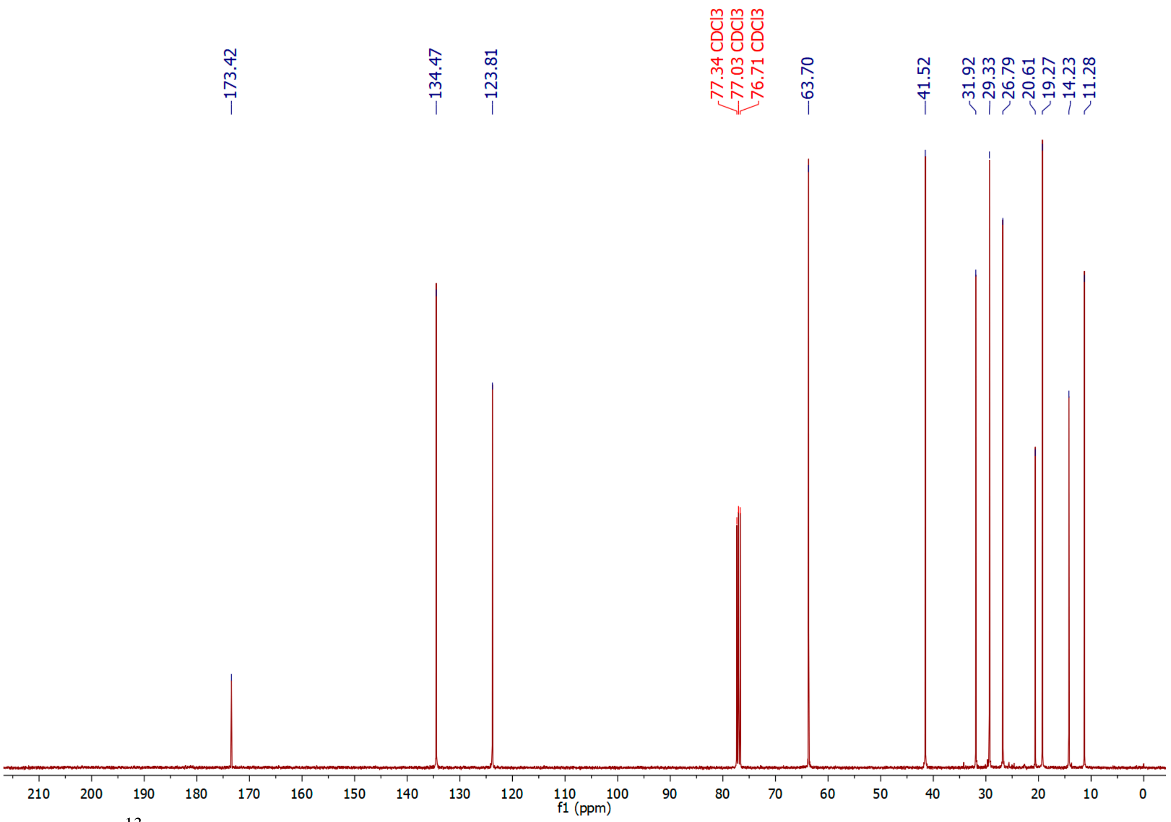

Figure S74c. ^{13}C NMR spectrum of (*Z*)-hex-3-en-1-yl 3-methylpentanoate *(***10f**) recorded in $CDCl_3$

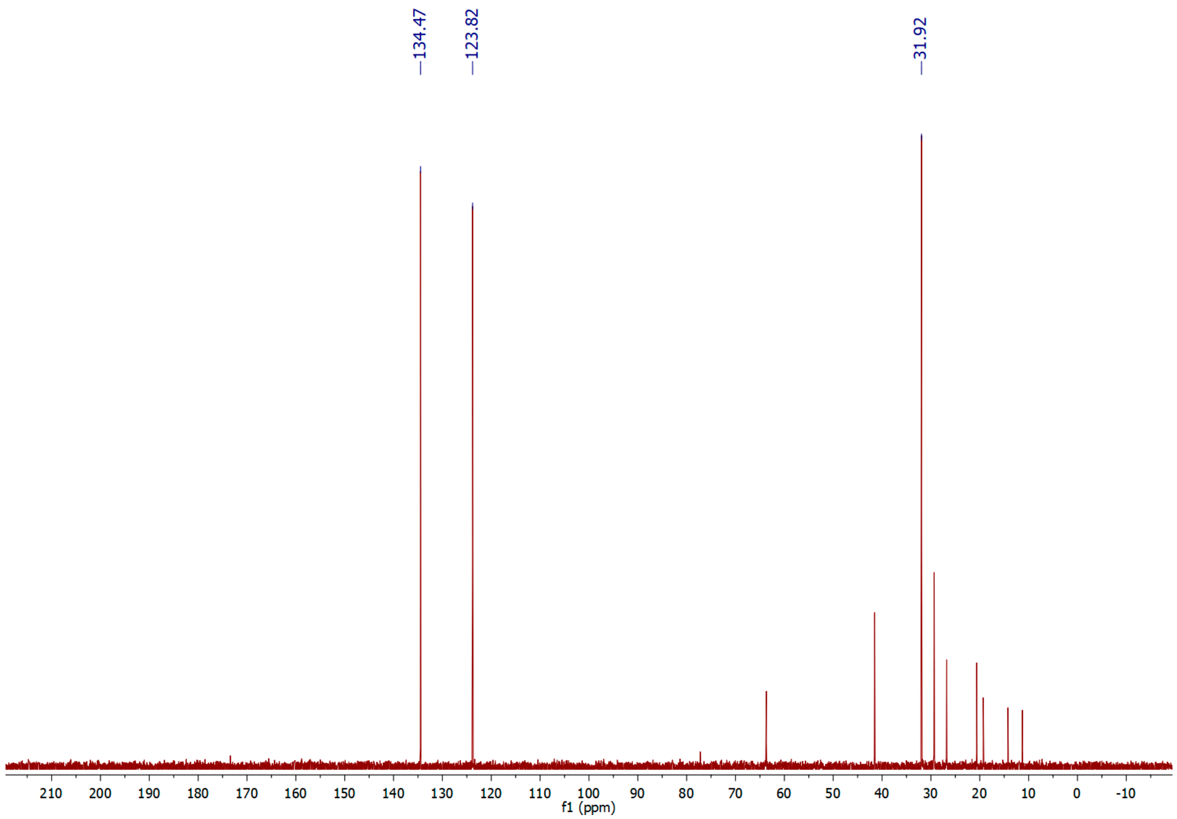

Figure S74d. DEPT 90 spectrum of (*Z*)-hex-3-en-1-yl 3-methylpentanoate *(***10f**) recorded in $CDCl_3$

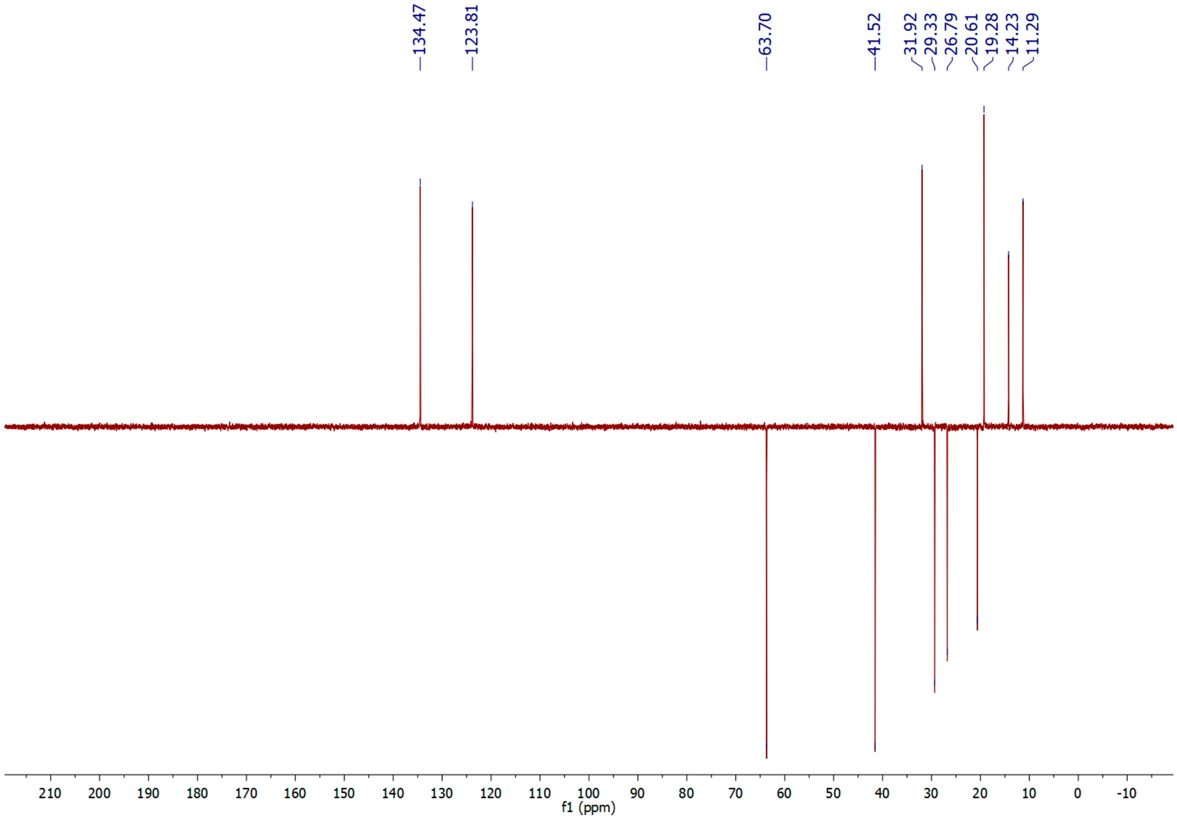

Figure S74e. DEPT 135 spectrum of (*Z*)-hex-3-en-1-yl 3-methylpentanoate *(***10f**) recorded in $CDCl_3$

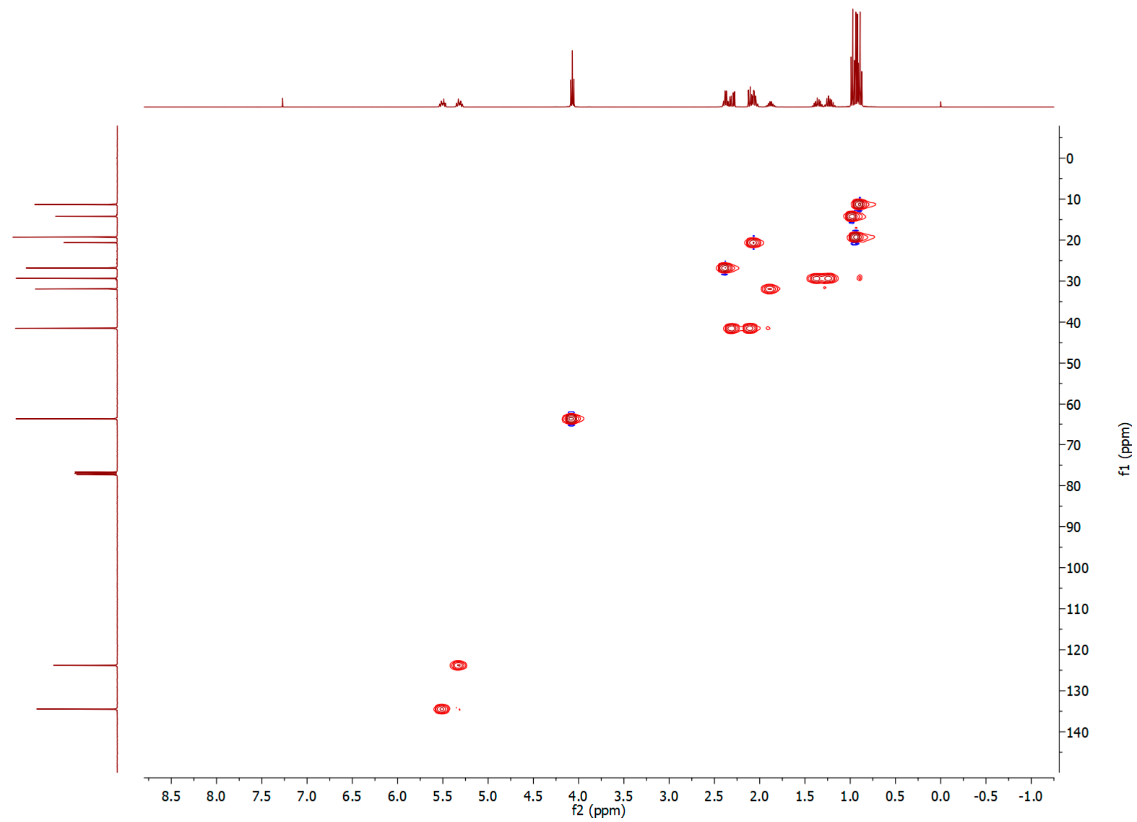

Figure S74f. HSQC spectrum of (*Z*)-hex-3-en-1-yl 3-methylpentanoate *(***10f**) recorded in $CDCl_3$

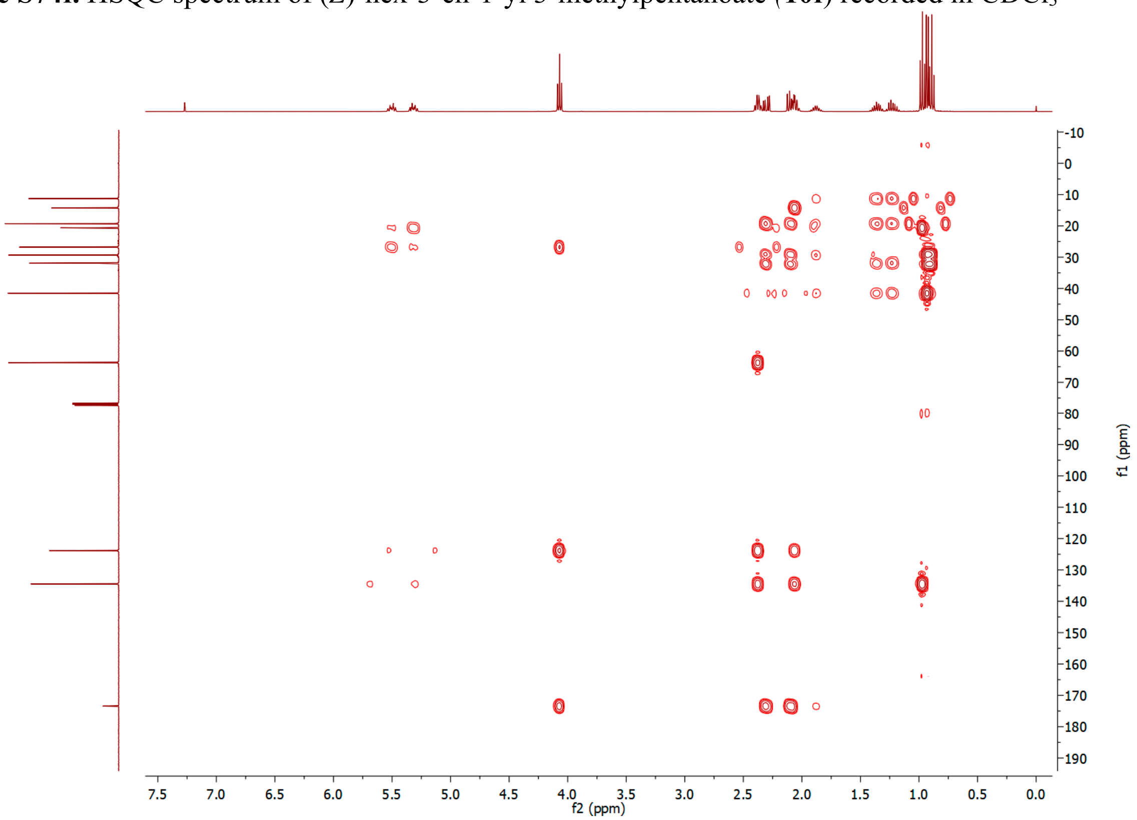

Figure S74g. HMBC spectrum of (*Z*)-hex-3-en-1-yl 3-methylpentanoate *(***10f**) recorded in $CDCl_3$

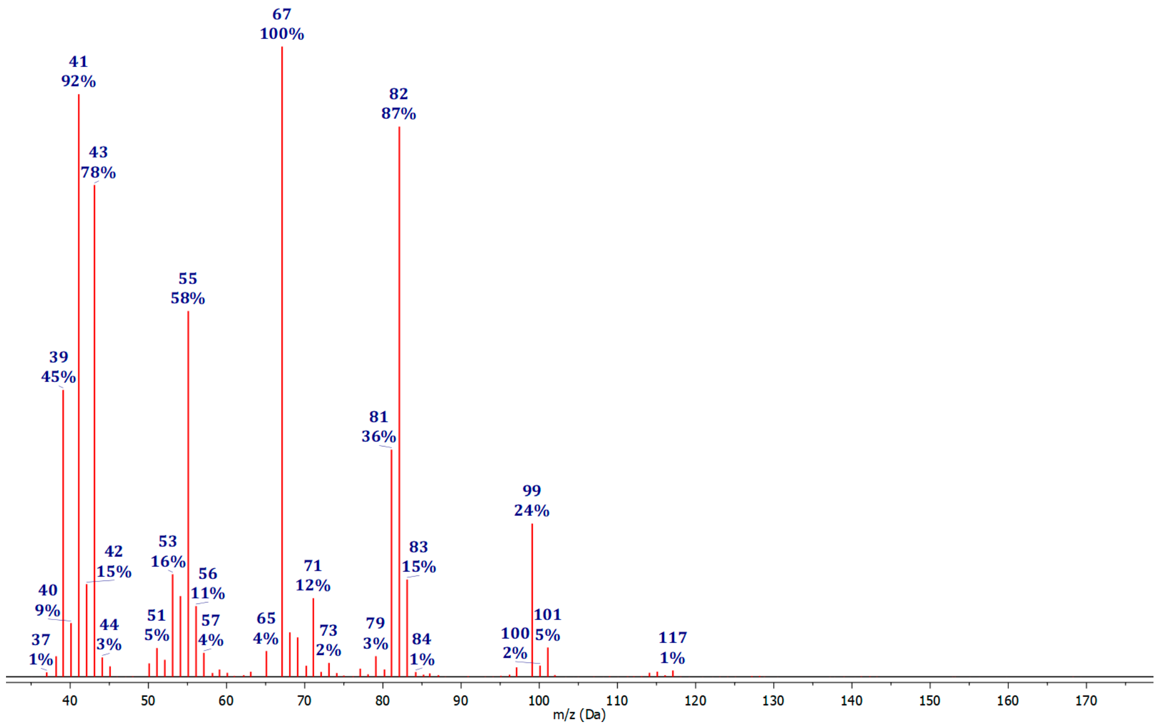

Figure S75. Mass spectrum of (*Z*)-hex-3-en-1-yl 4-methylpentanoate *(***10g**)

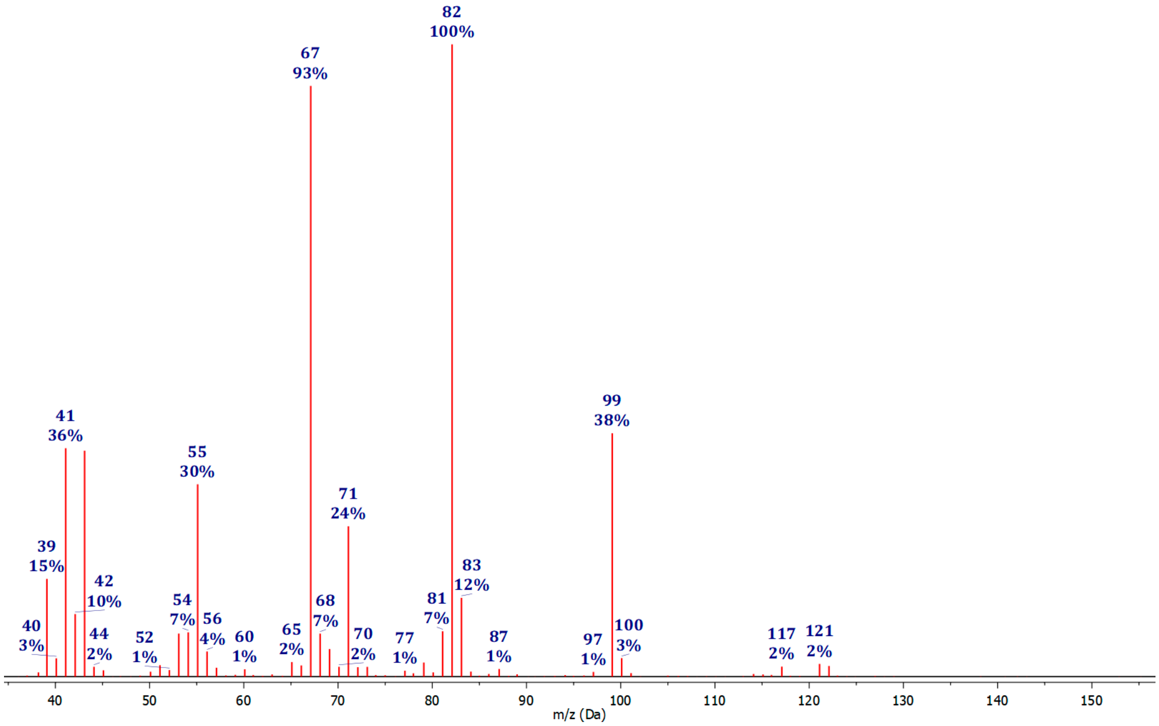

Figure S76. Mass spectrum of (*Z*)-hex-3-en-1-yl hexanoate *(***10h**)

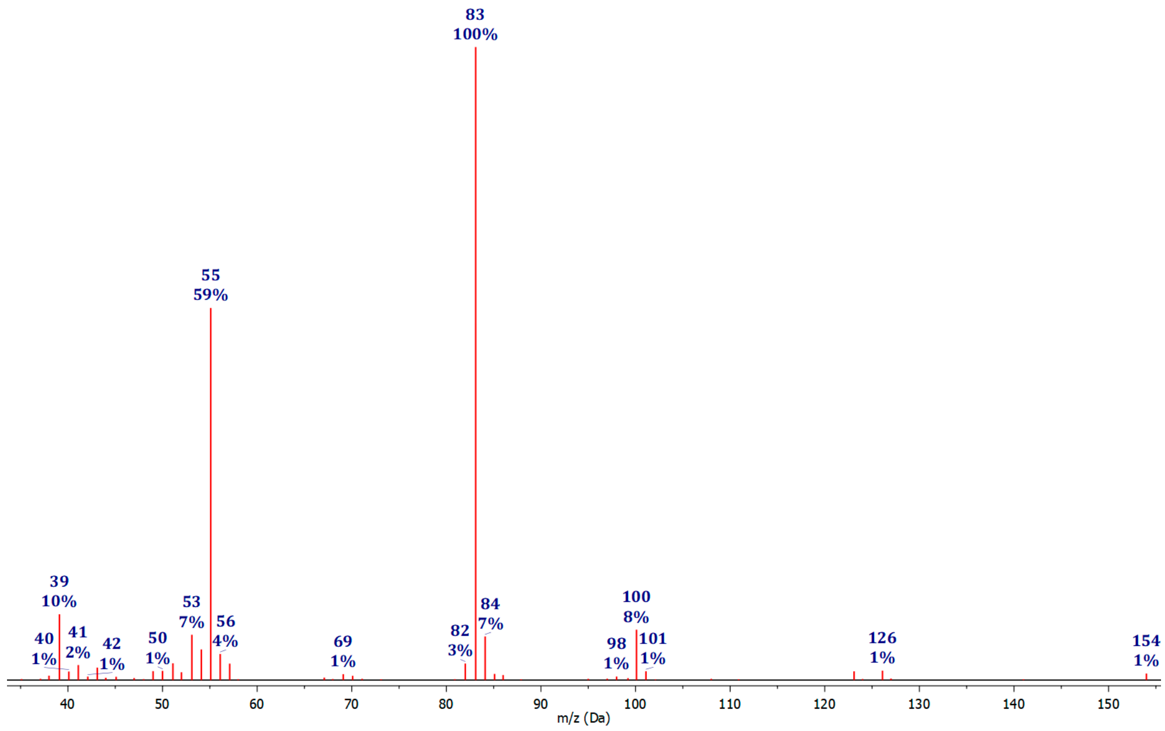

Figure S77. Mass spectrum of 1-methylhexyl angelate (**11o**)

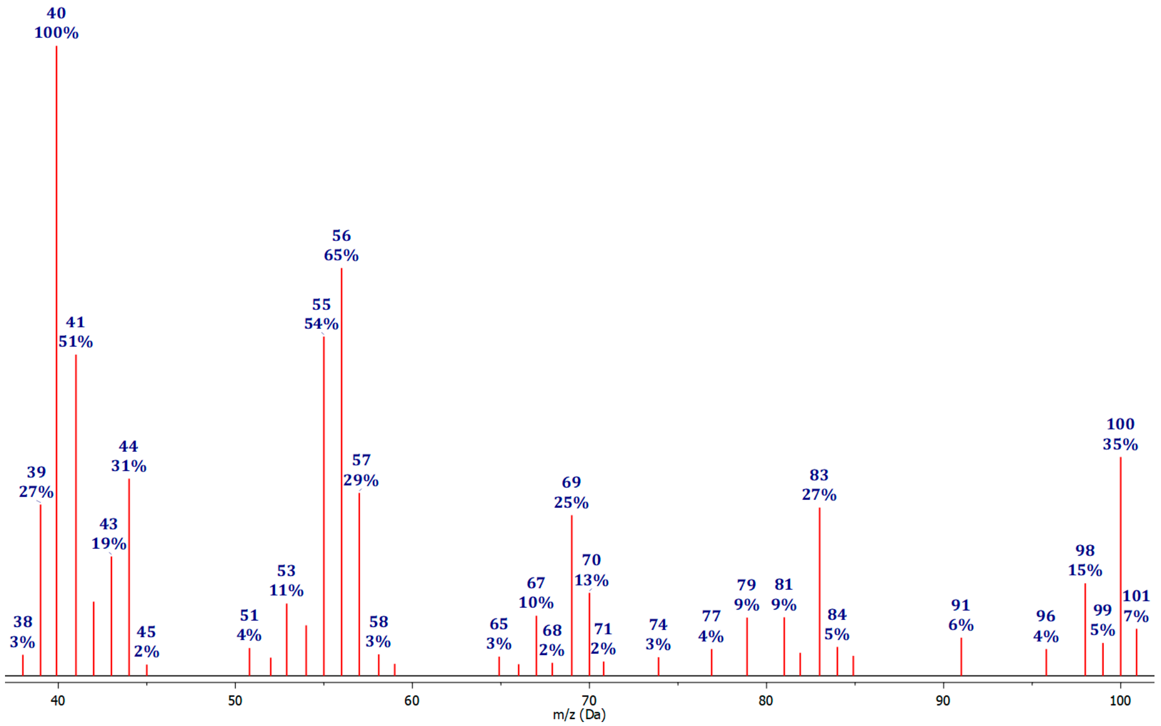

Figure S78. Mass spectrum of 2-methylhexyl angelate (**12o**)

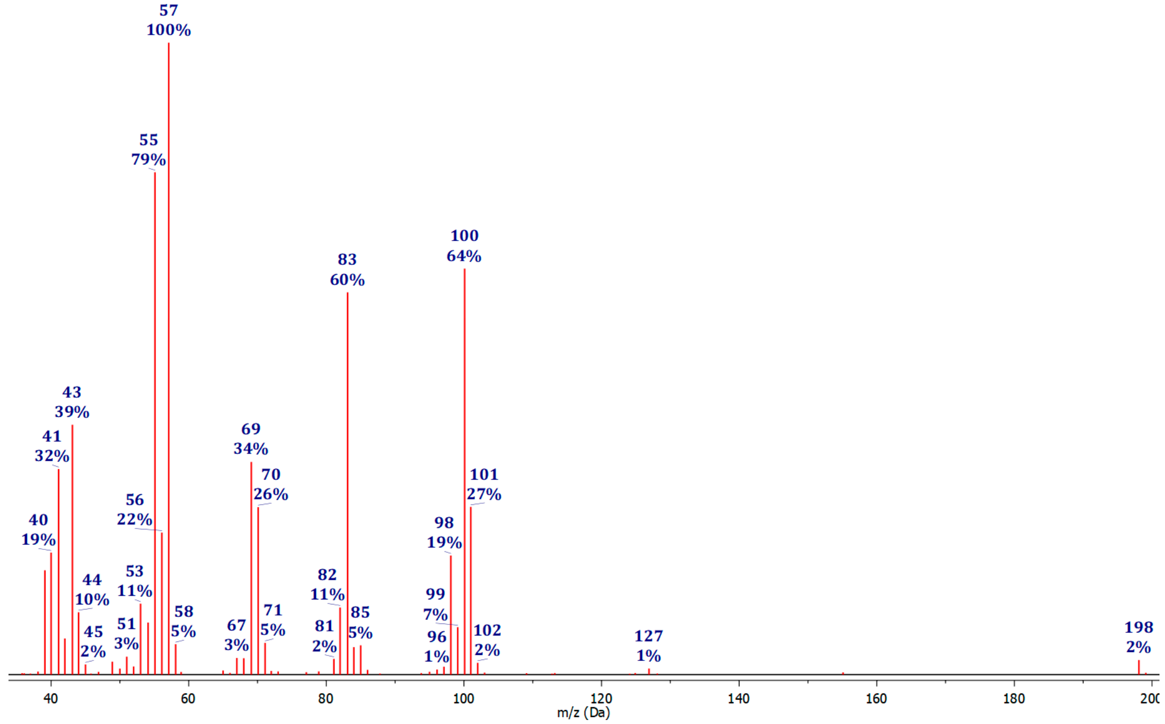

Figure S79. Mass spectrum of 3-methylhexyl angelate (**13o**)

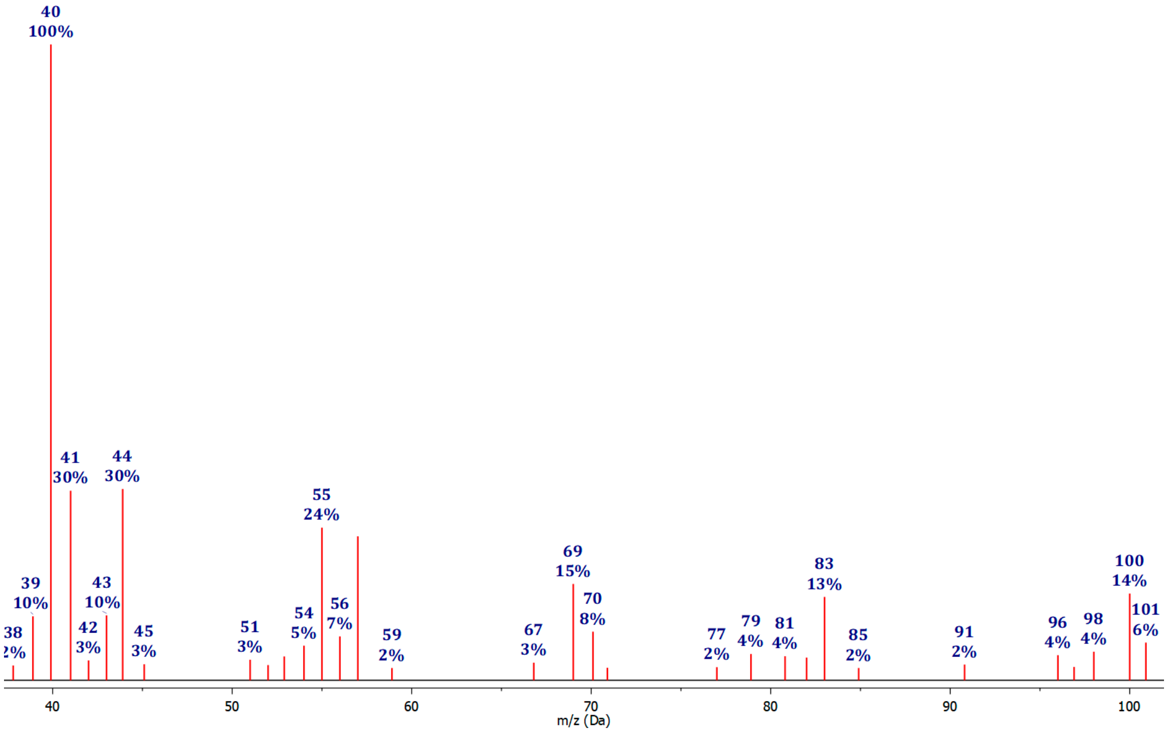

Figure S80. Mass spectrum of 4-methylhexyl angelate (**14o**)

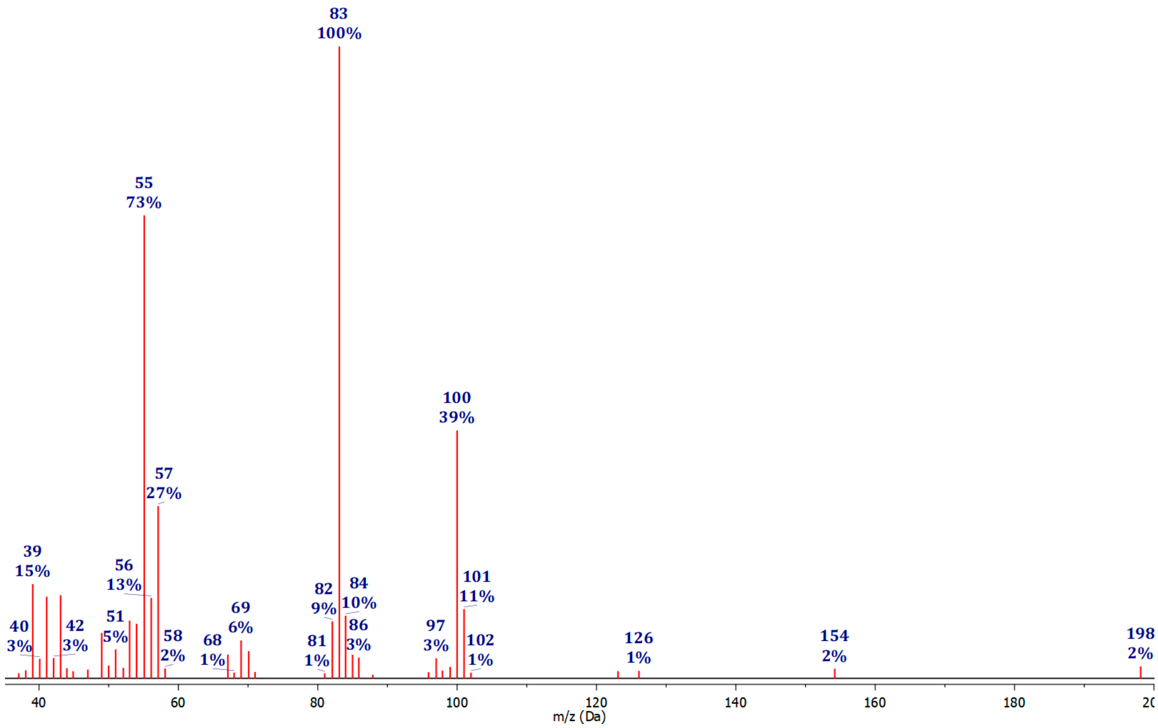

Figure S81. Mass spectrum of 5-methylhexyl angelate (**15o**)

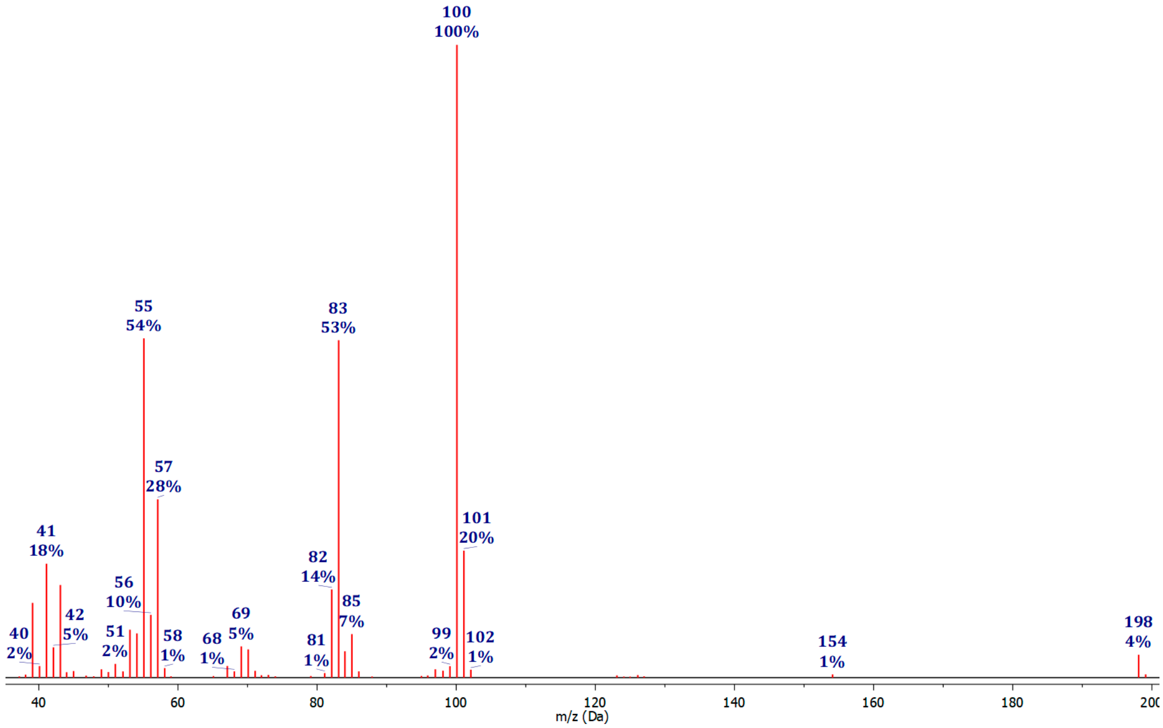

Figure S82. Mass spectrum of heptyl angelate (**16o**)

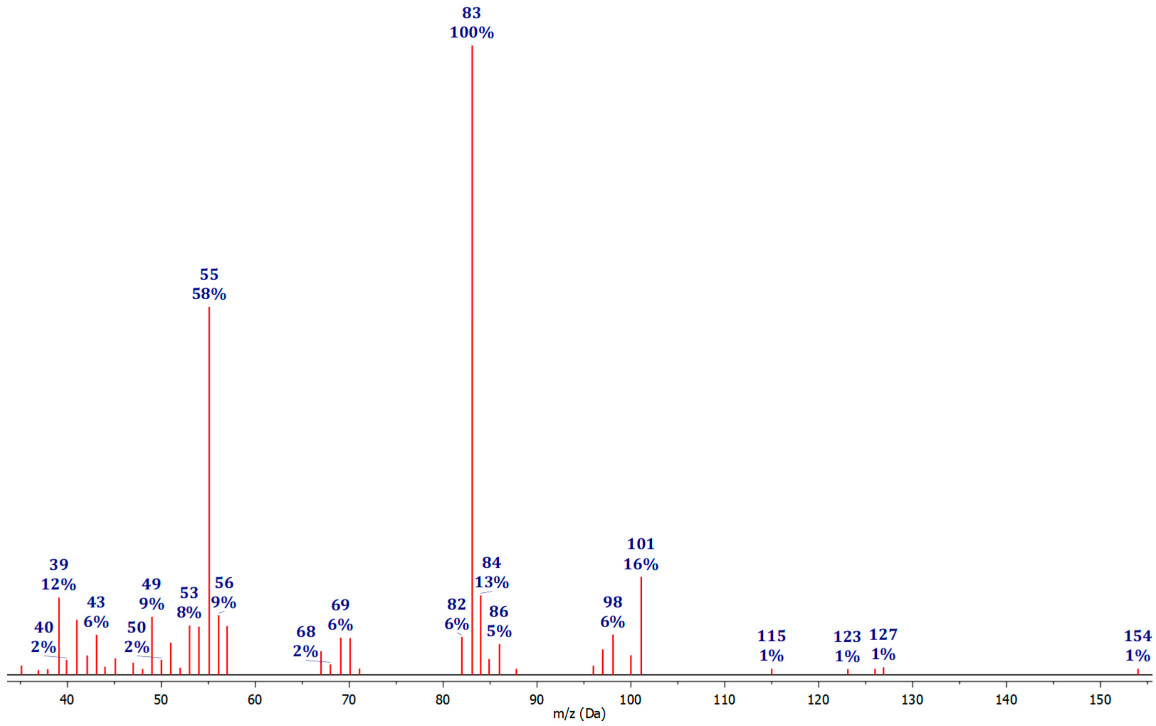

Figure S83. Mass spectrum of 1-methylhexyl tiglate (**11p**)

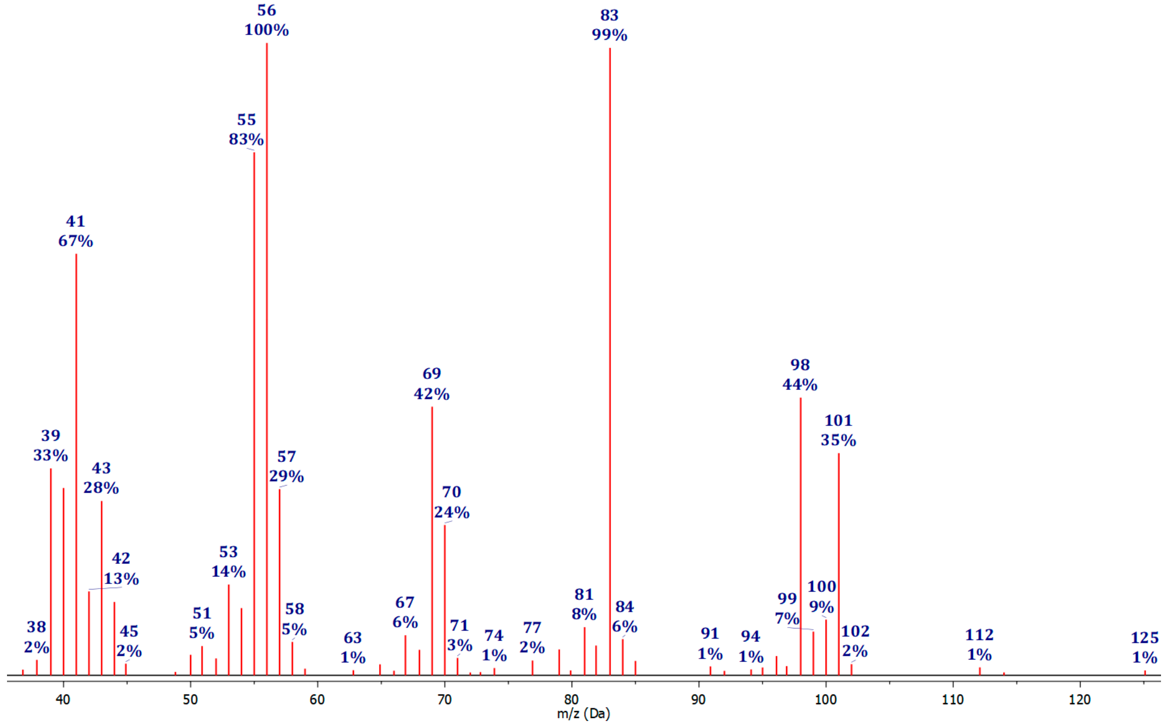

Figure S84. Mass spectrum of 2-methylhexyl tiglate (**12p**)

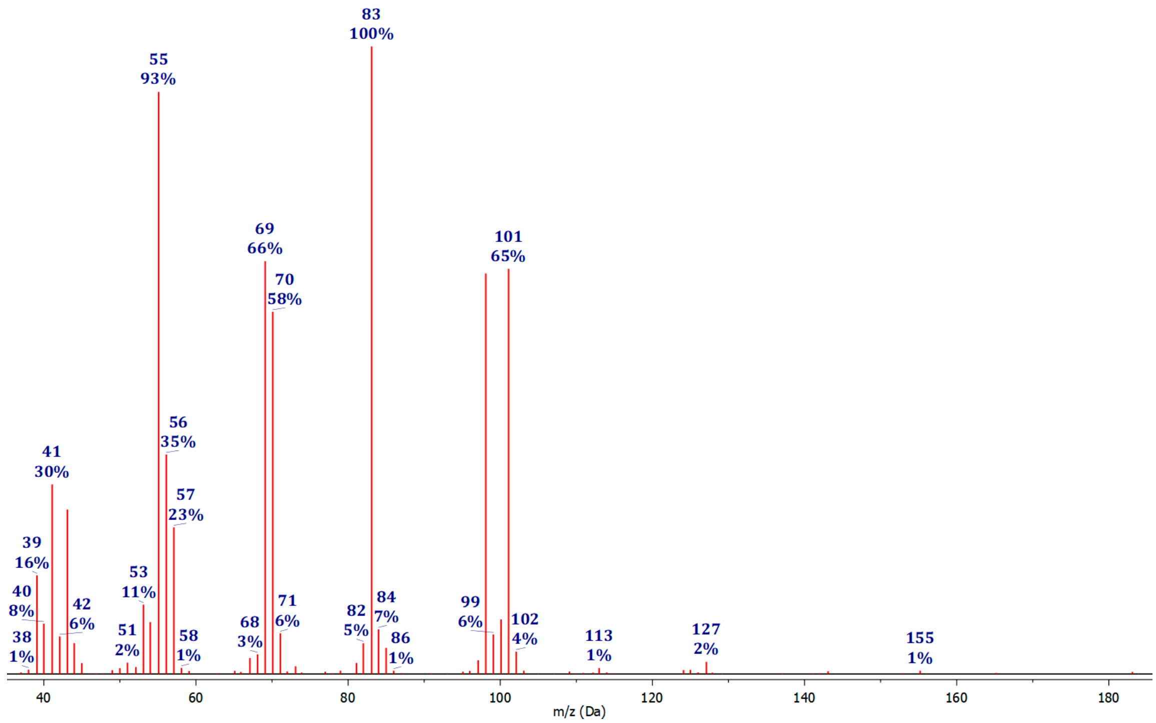

Figure S85. Mass spectrum of 3-methylhexyl tiglate (**13p**)

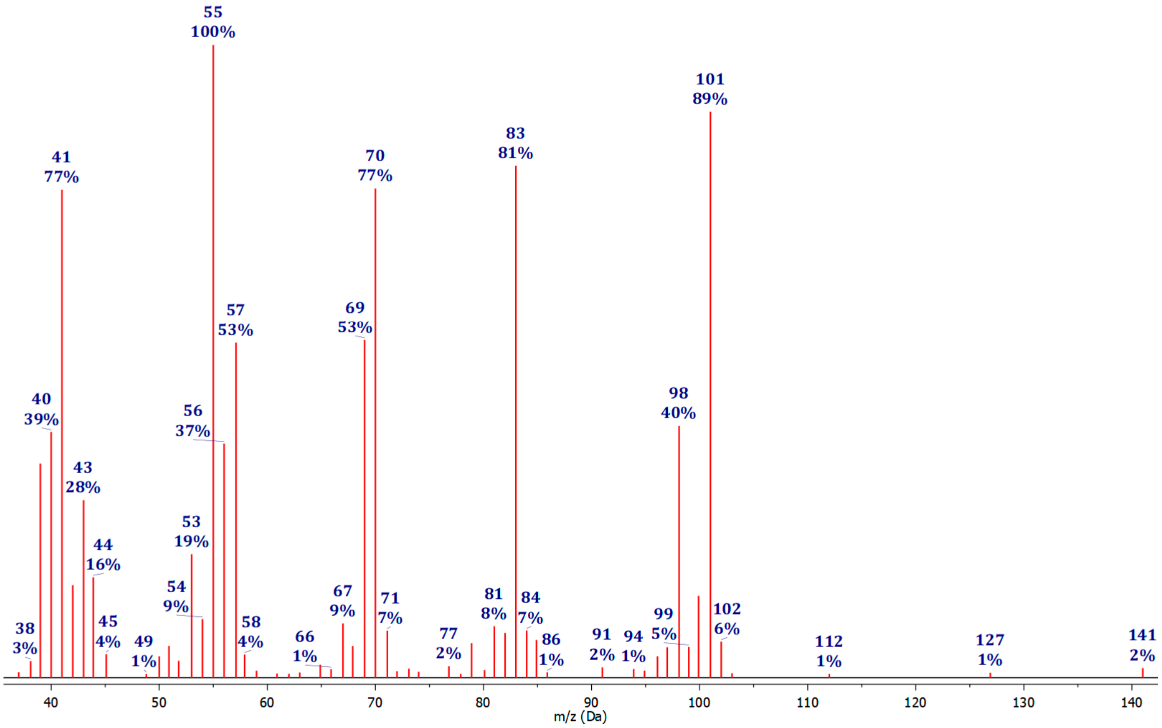

Figure S86. Mass spectrum of 4-methylhexyl tiglate (**14p**)

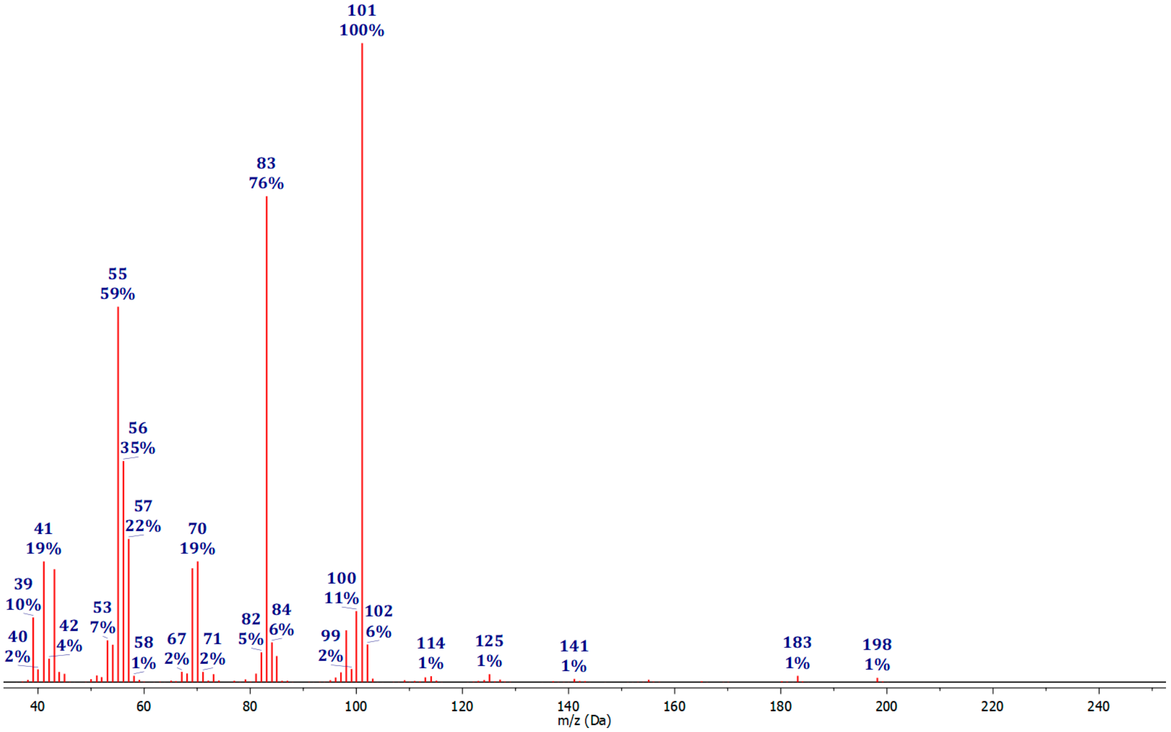

Figure S87a. Mass spectrum of 5-methylhexyl tiglate (**15p**)

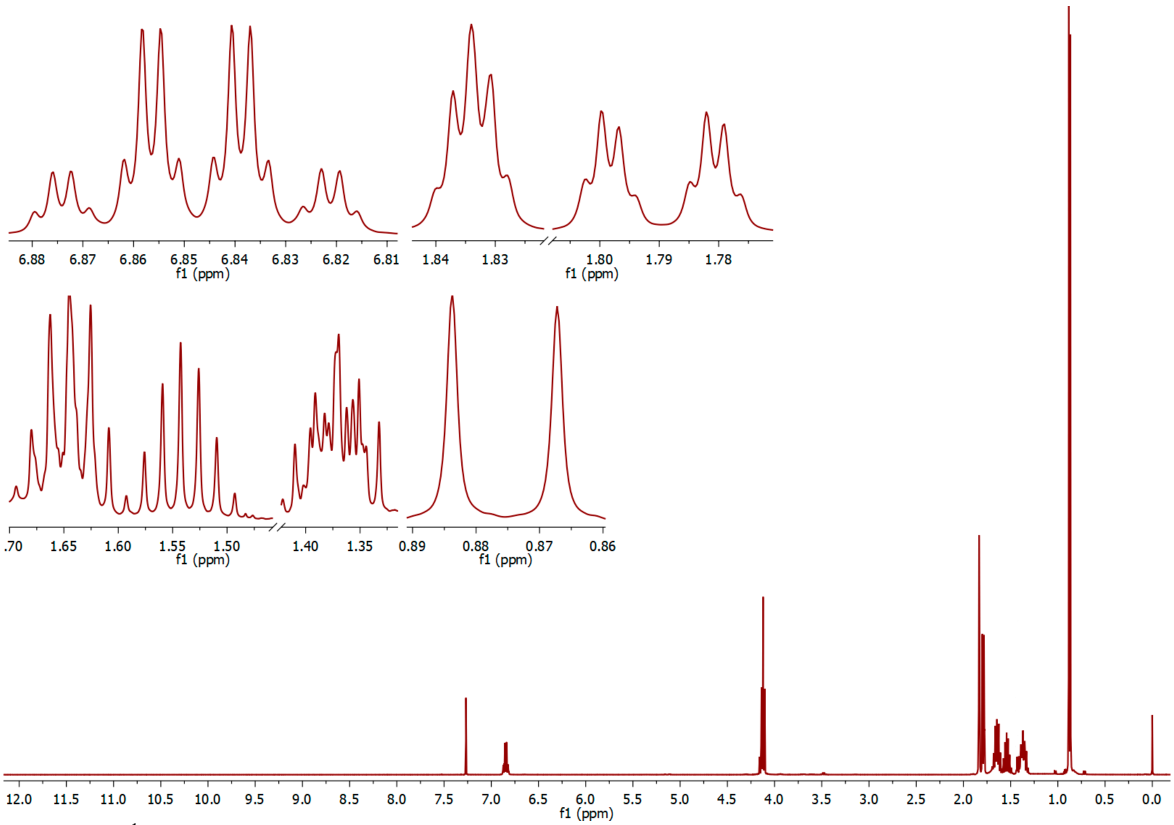

Figure S87b. ^{1}H NMR spectrum of 5-methylhexyl tiglate (**15p**) recorded in $CDCl_3$

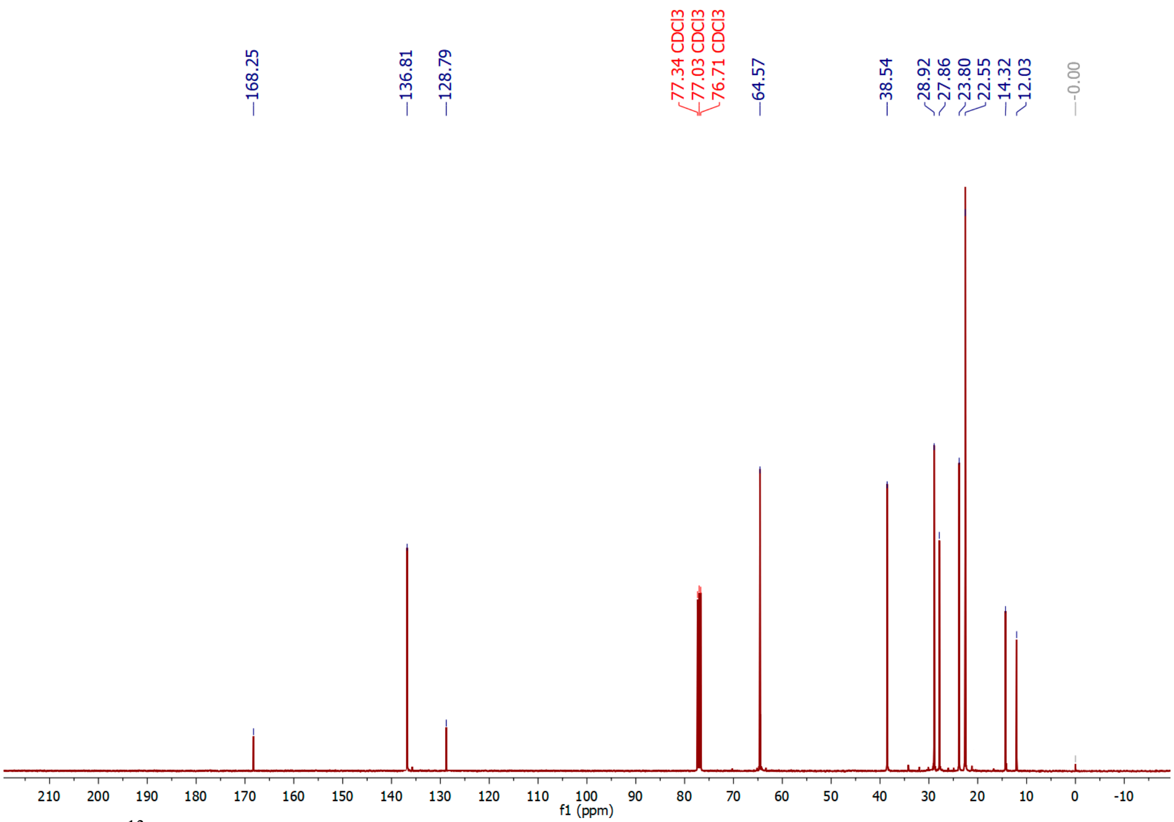

Figure S87c. ^{13}C NMR spectrum of 5-methylhexyl tiglate (**15p**) recorded in $CDCl_3$

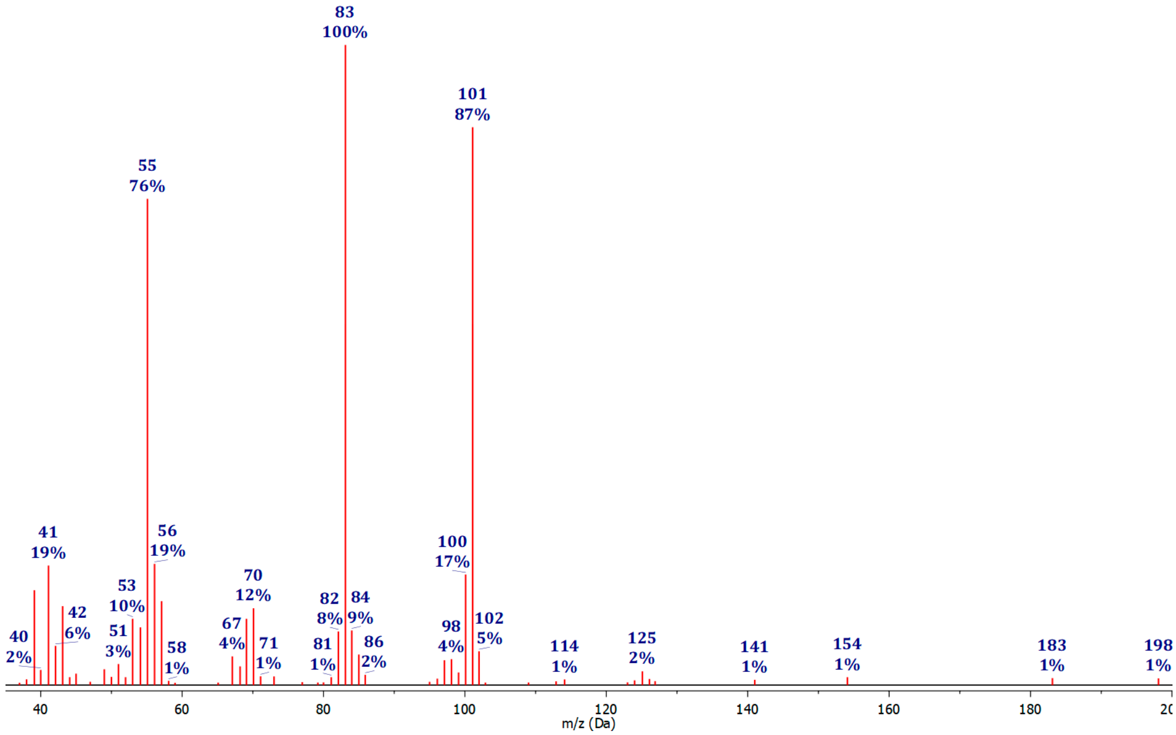

Figure S88. Mass spectrum of heptyl tiglate (**16p**)

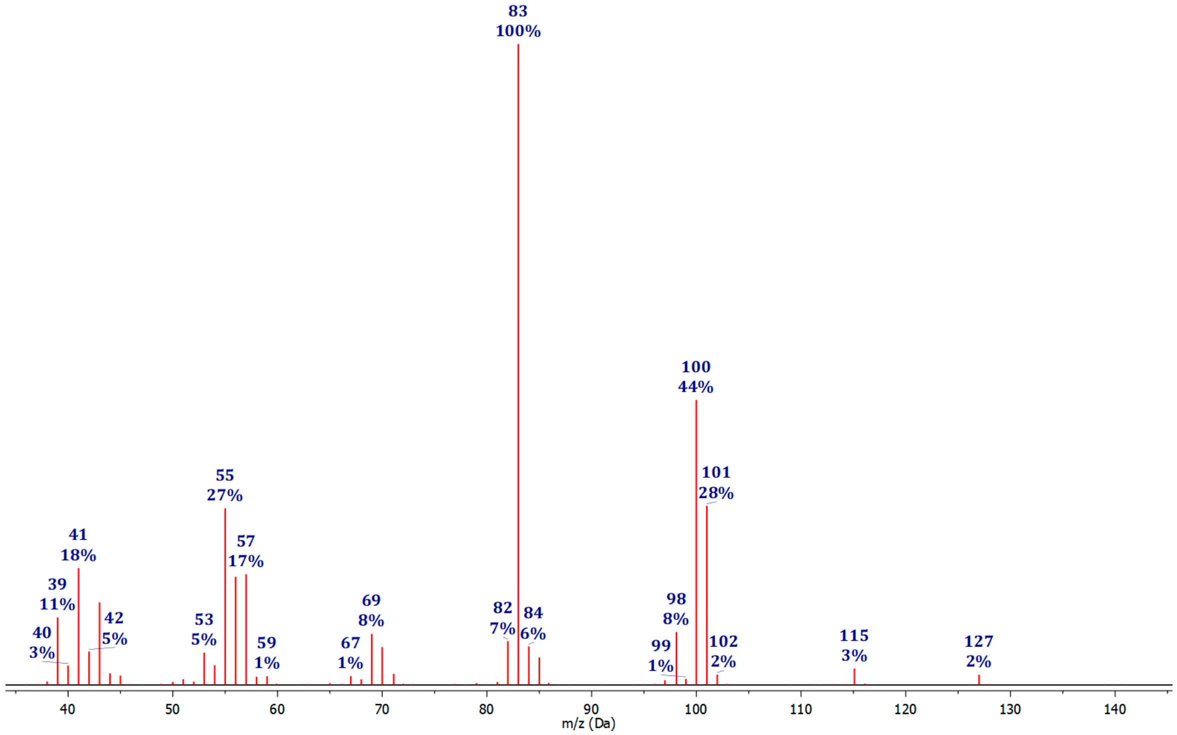

Figure S89. Mass spectrum of 1-methylhexyl senecioate (**11q**)

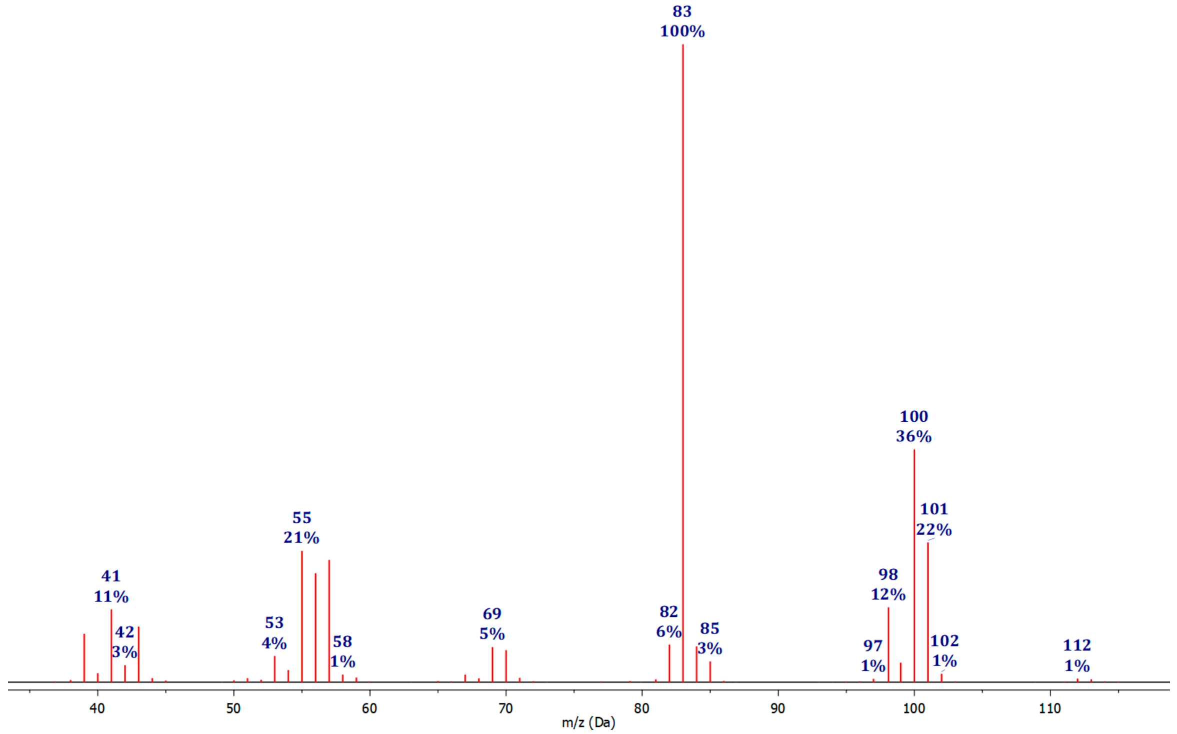

Figure S90. Mass spectrum of 2-methylhexyl senecioate (**12q**)

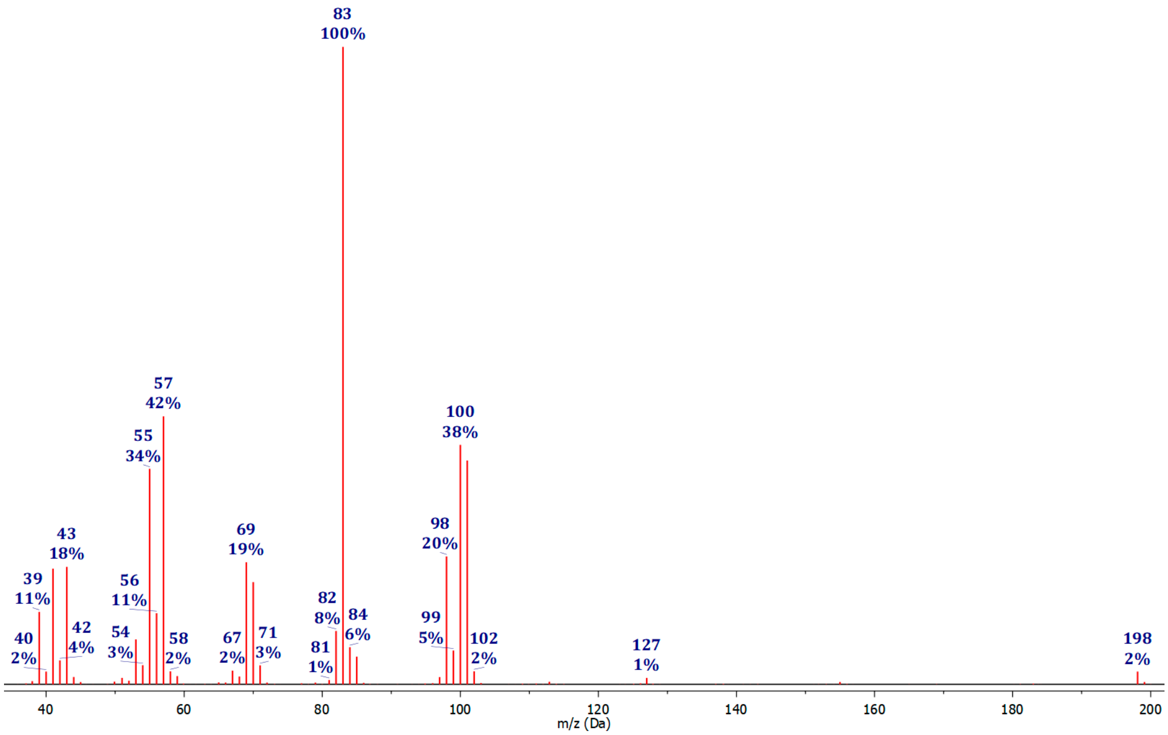

Figure S91. Mass spectrum of 3-methylhexyl senecioate (**13q**)

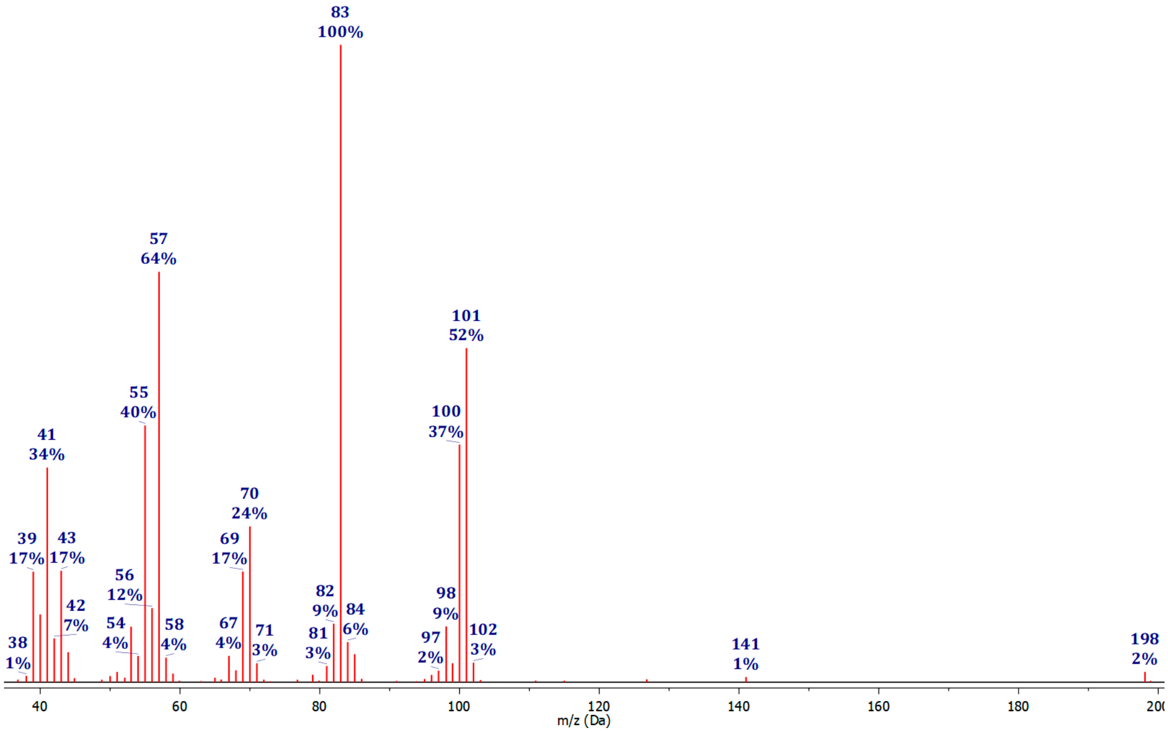

Figure S92. Mass spectrum of 4-methylhexyl senecioate (**14q**)

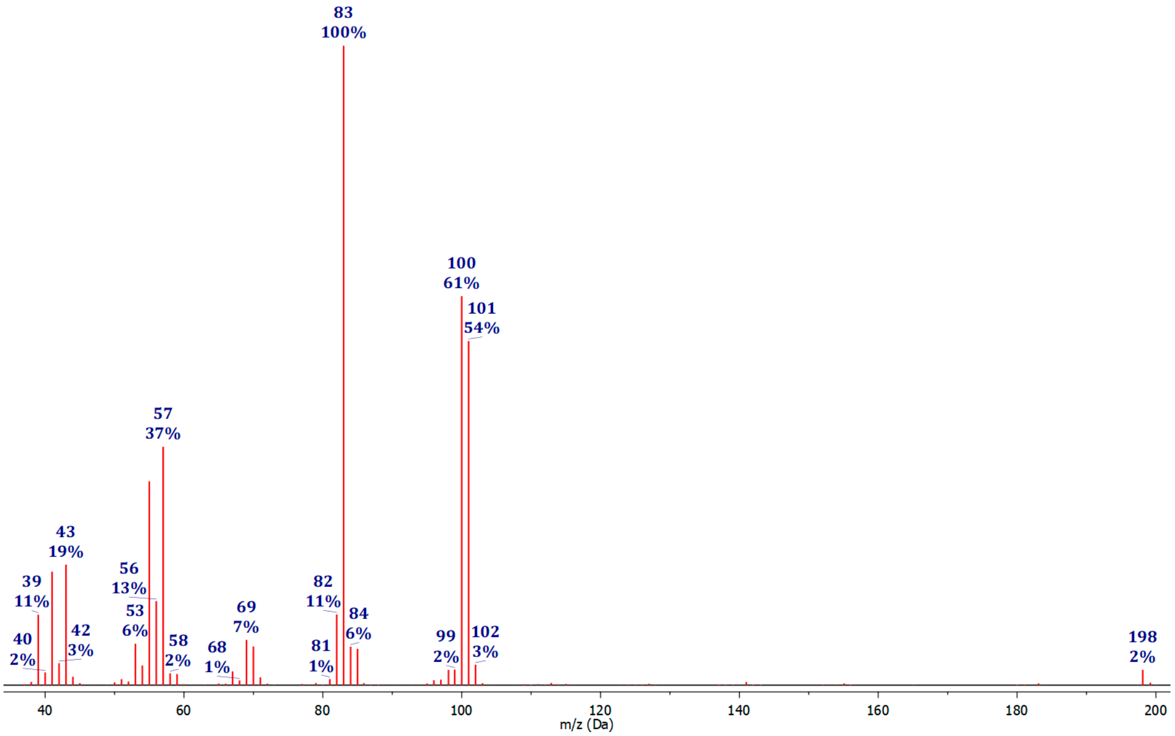

Figure S93. Mass spectrum of 5-methylhexyl senecioate (**15q**)

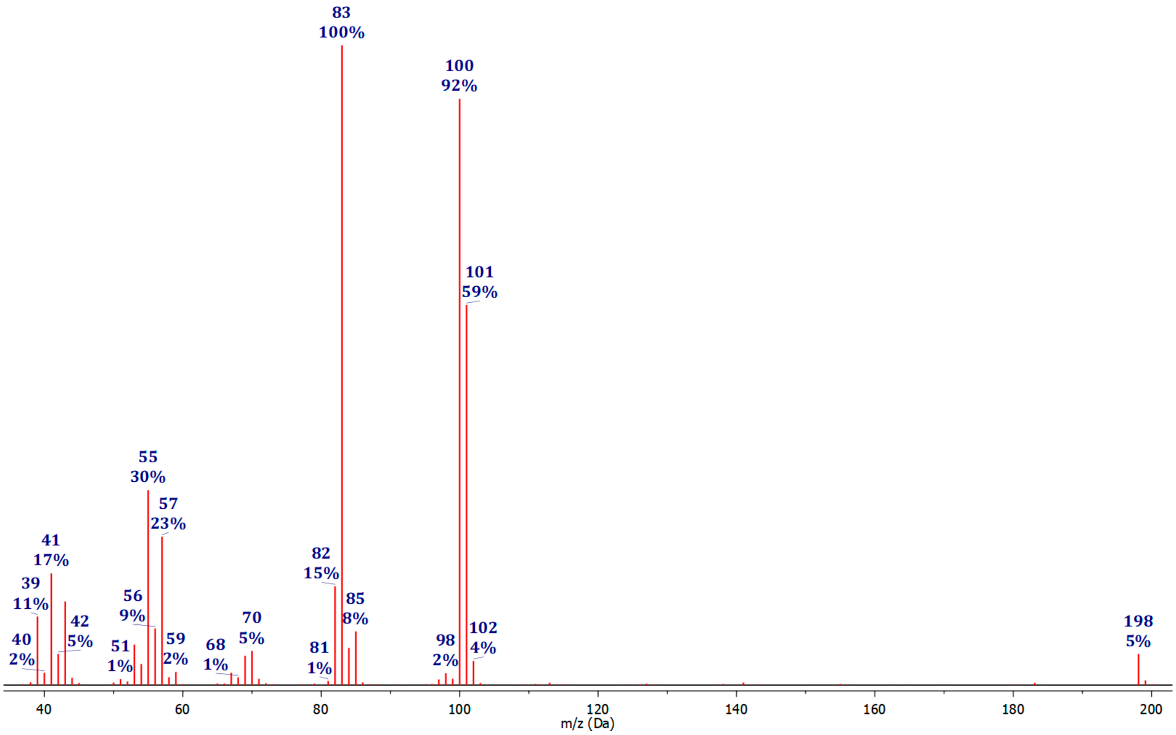

Figure S94. Mass spectrum of heptyl senecioate (**16q**)

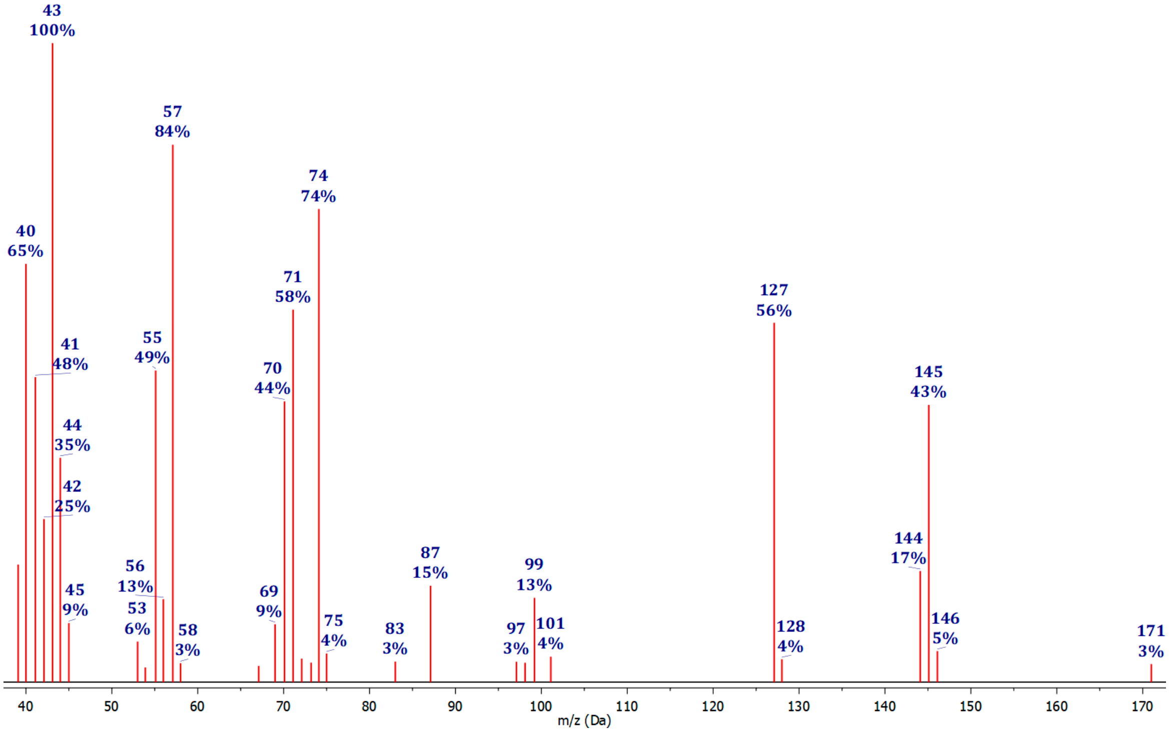

Figure S95a. Mass spectrum of 1-methylbutyl 2-methylheptanoate (**1l epimer I**)

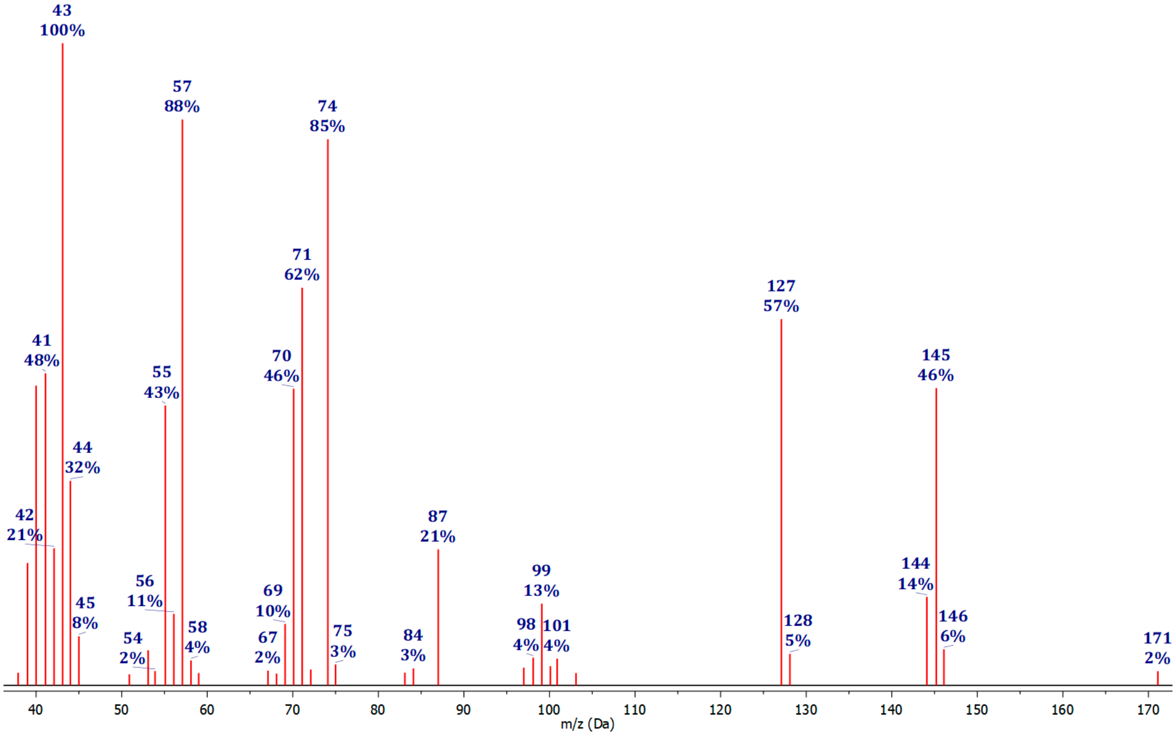

Figure S95b. Mass spectrum of 1-methylbutyl 2-methylheptanoate (**1l epimer II**)

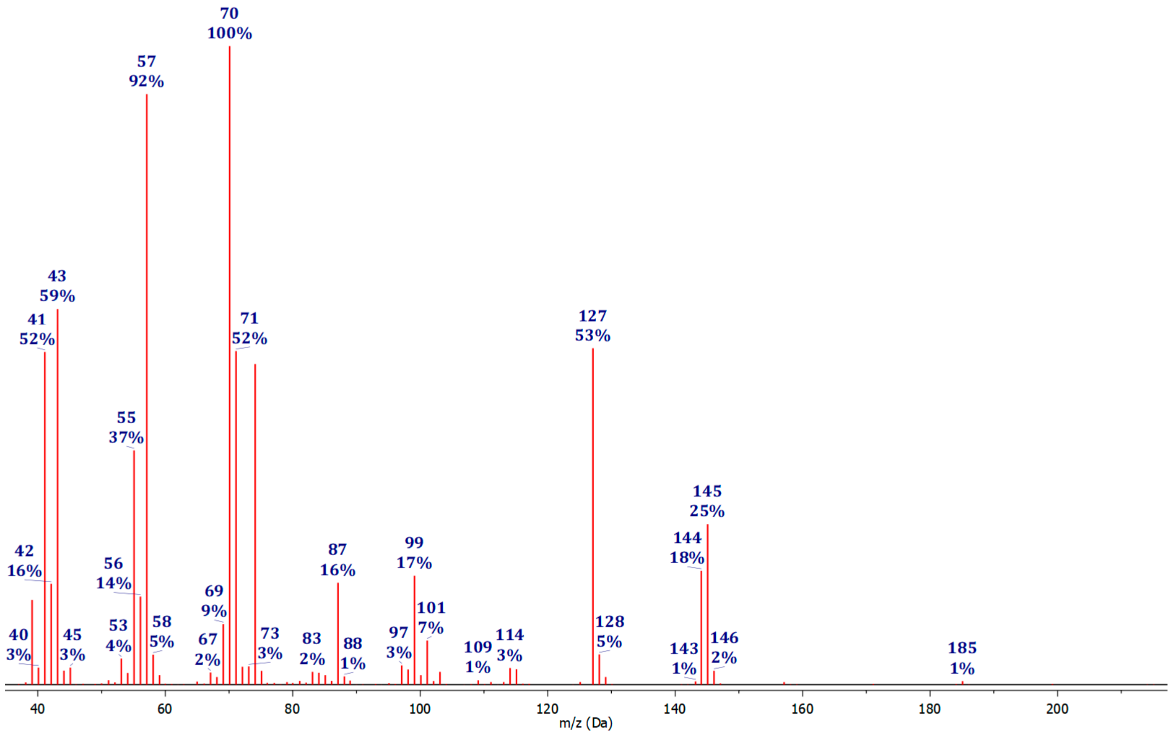

Figure S96. Mass spectrum of 2-methylbutyl 2-methylheptanoate (**2l**)

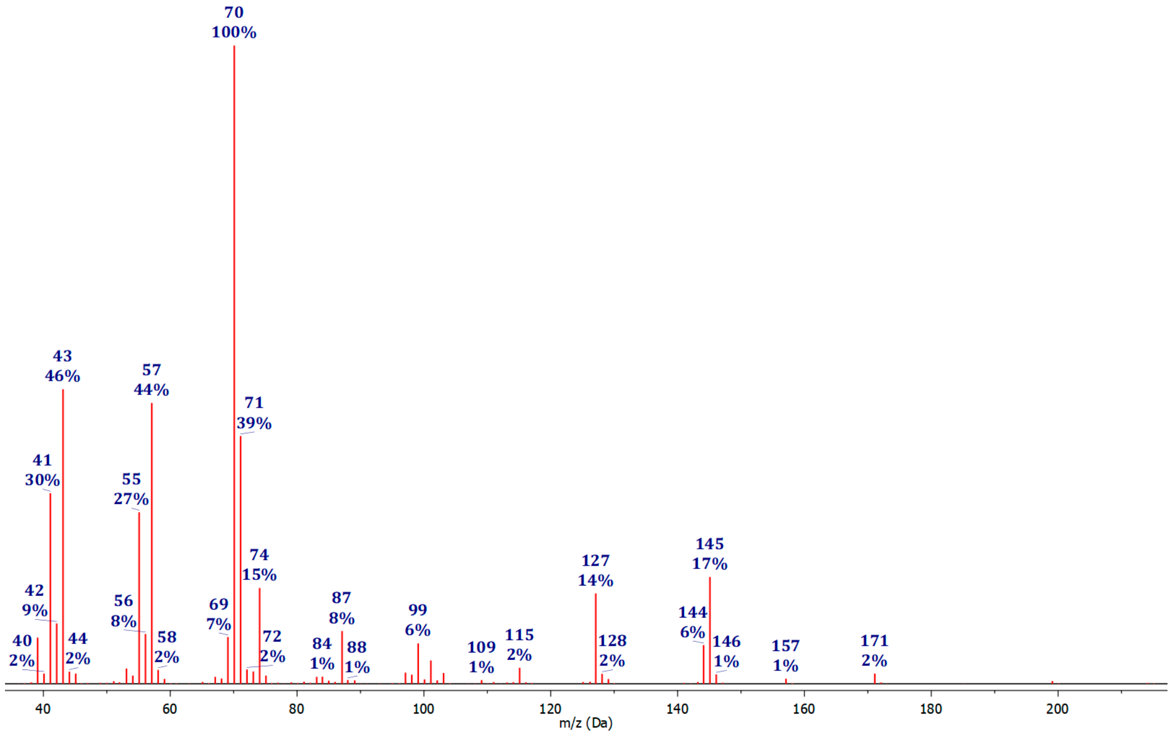

Figure S97. Mass spectrum of 3-methylbutyl 2-methylheptanoate (**3l**)

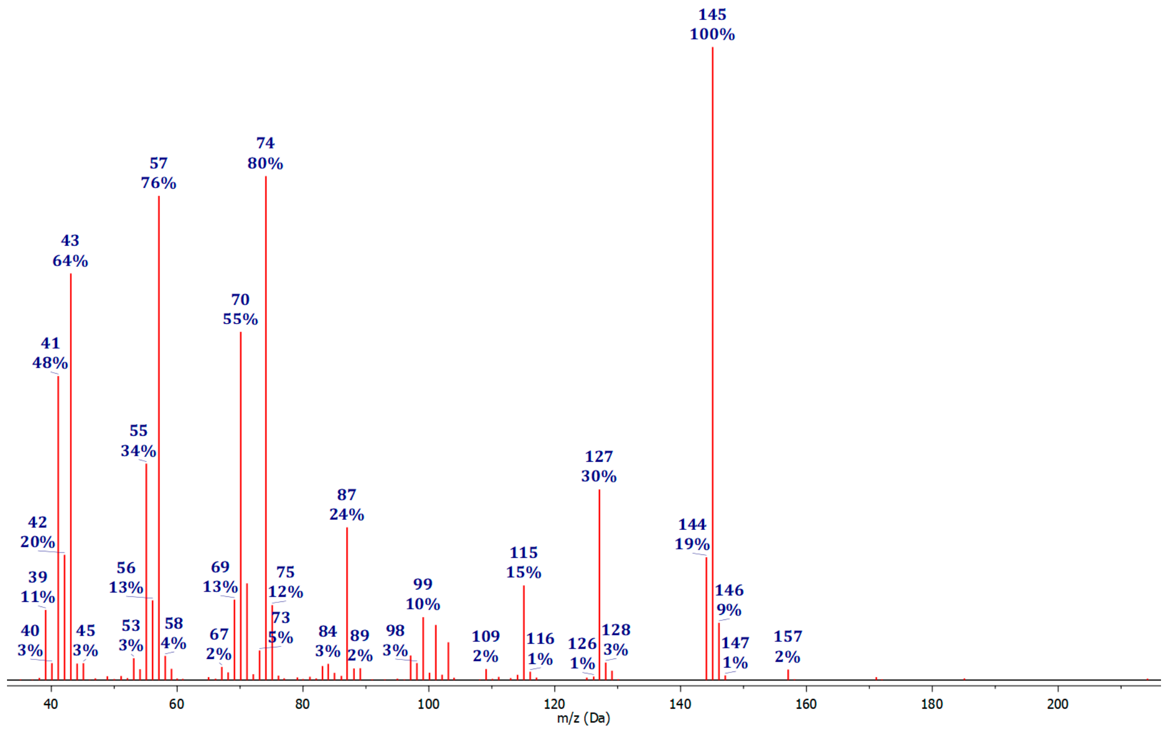

Figure S98. Mass spectrum of pentyl 2-methylheptanoate (**4l**)

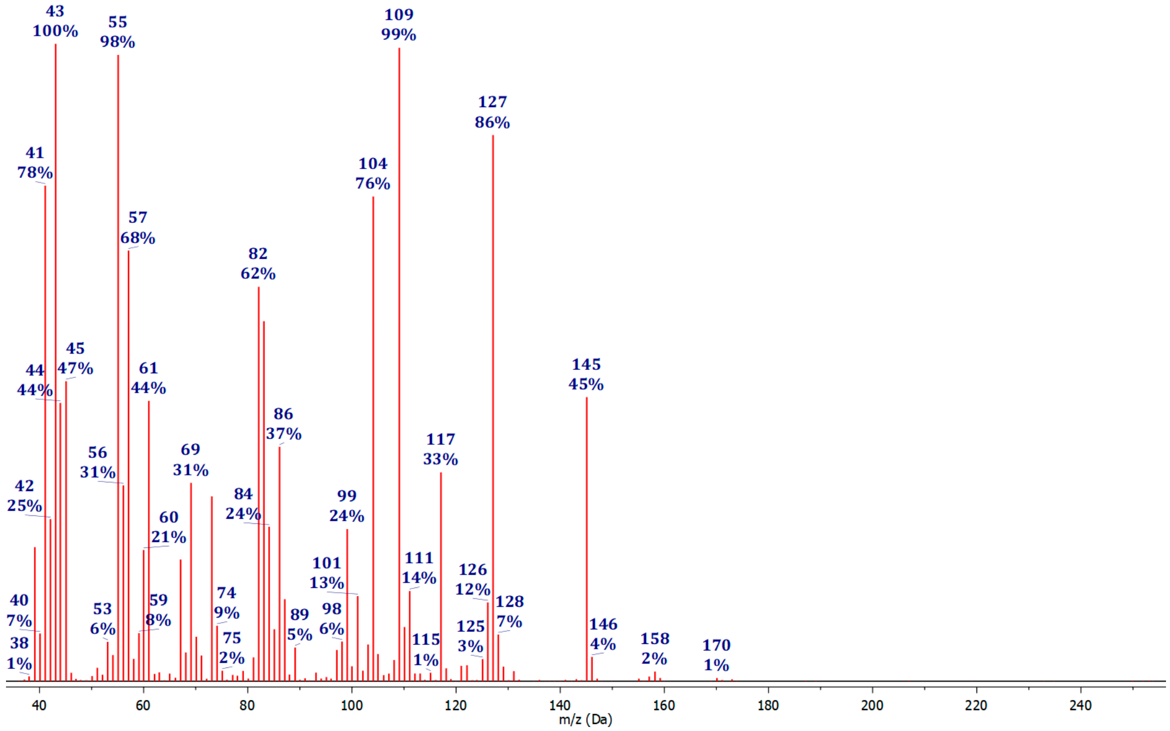

Figure S99. Mass spectrum of 1-methylbutyl 6-methylheptanoate (**1m**)

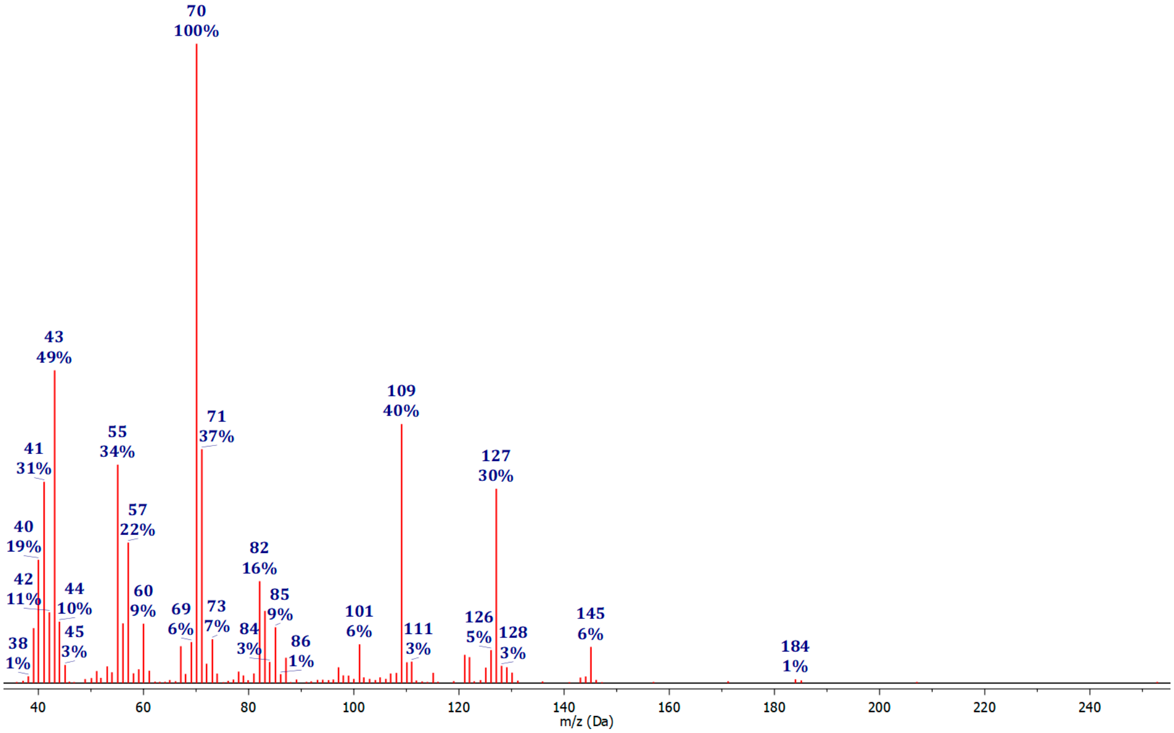

Figure S100. Mass spectrum of 2-methylbutyl 6-methylheptanoate (**2m**)

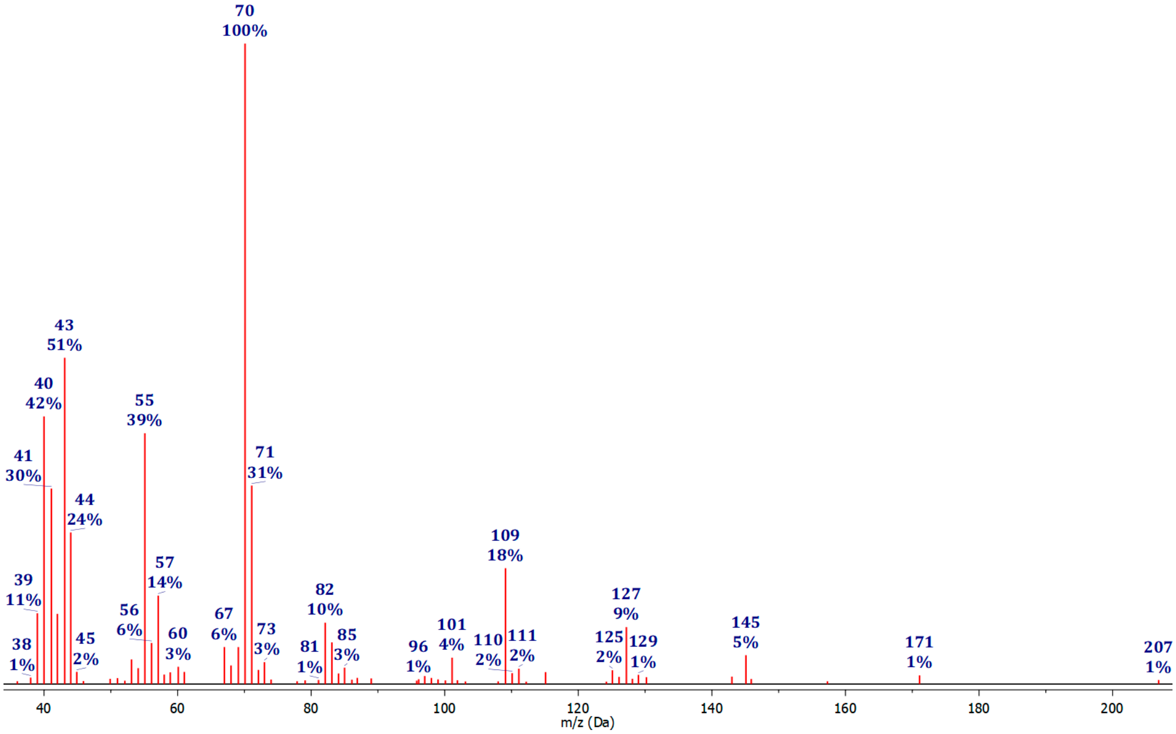

Figure S101. Mass spectrum of 3-methylbutyl 6-methylheptanoate (**3m**)

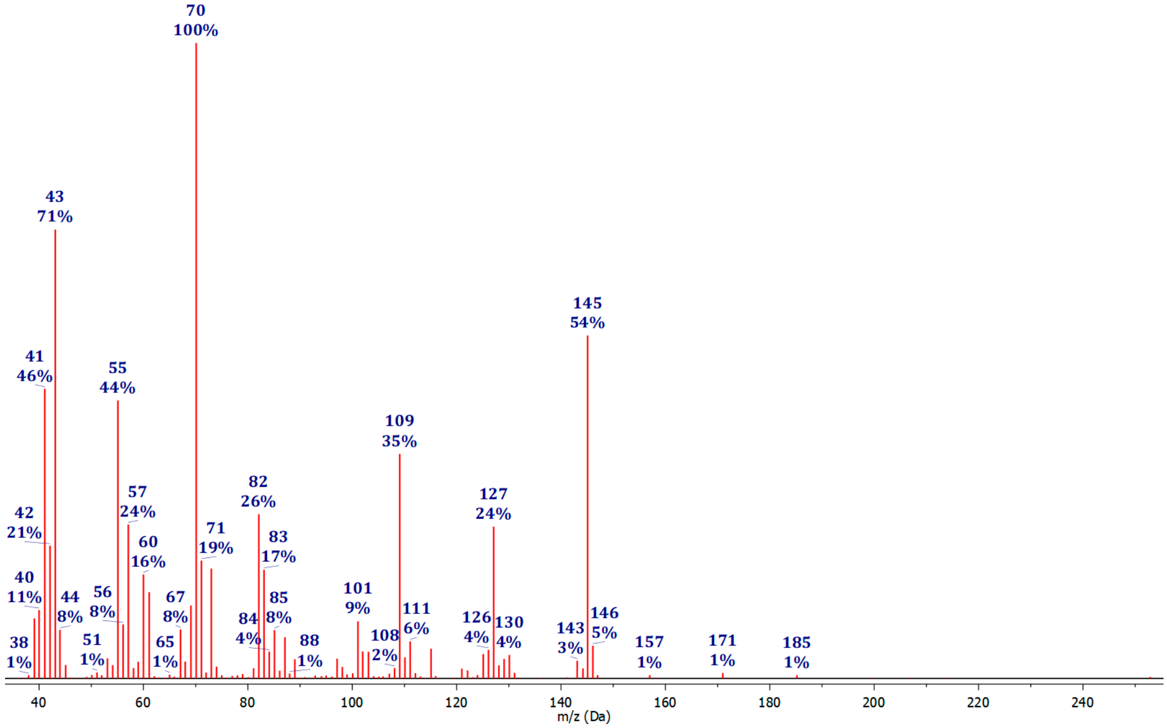

Figure S102. Mass spectrum of pentyl 6-methylheptanoate (**4m**)

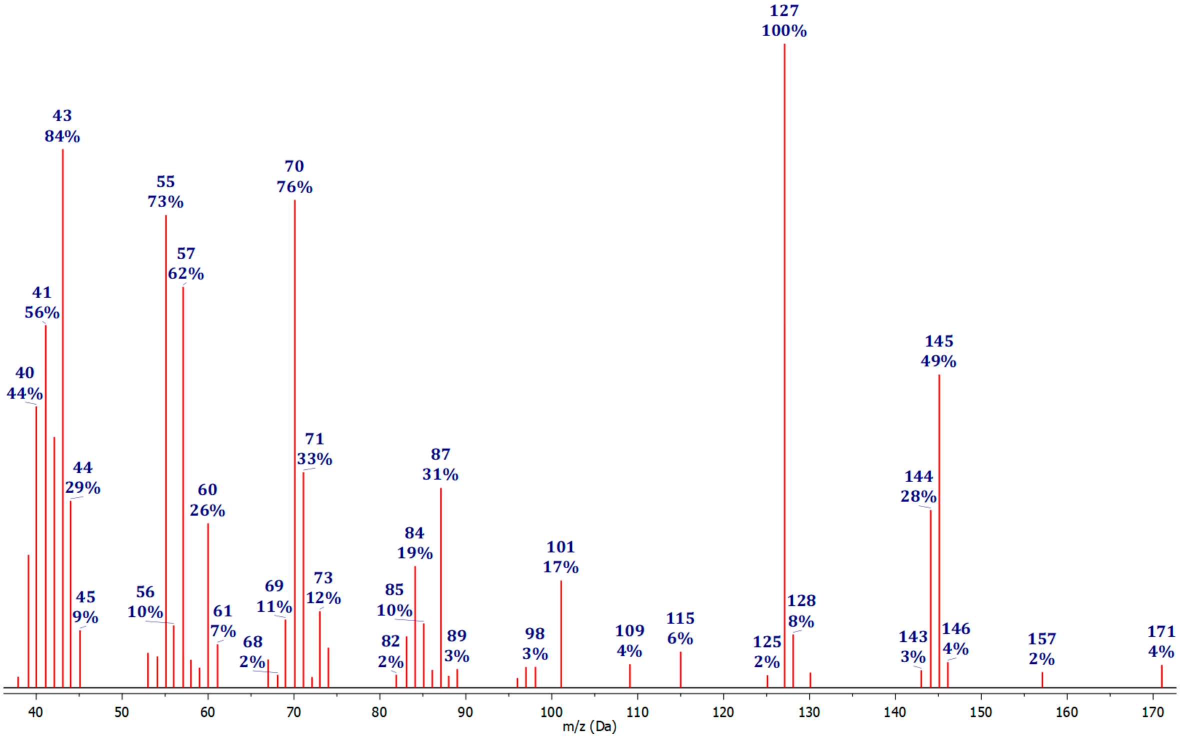

Figure S103. Mass spectrum of 1-methylbutyl octanoate (**1n**)

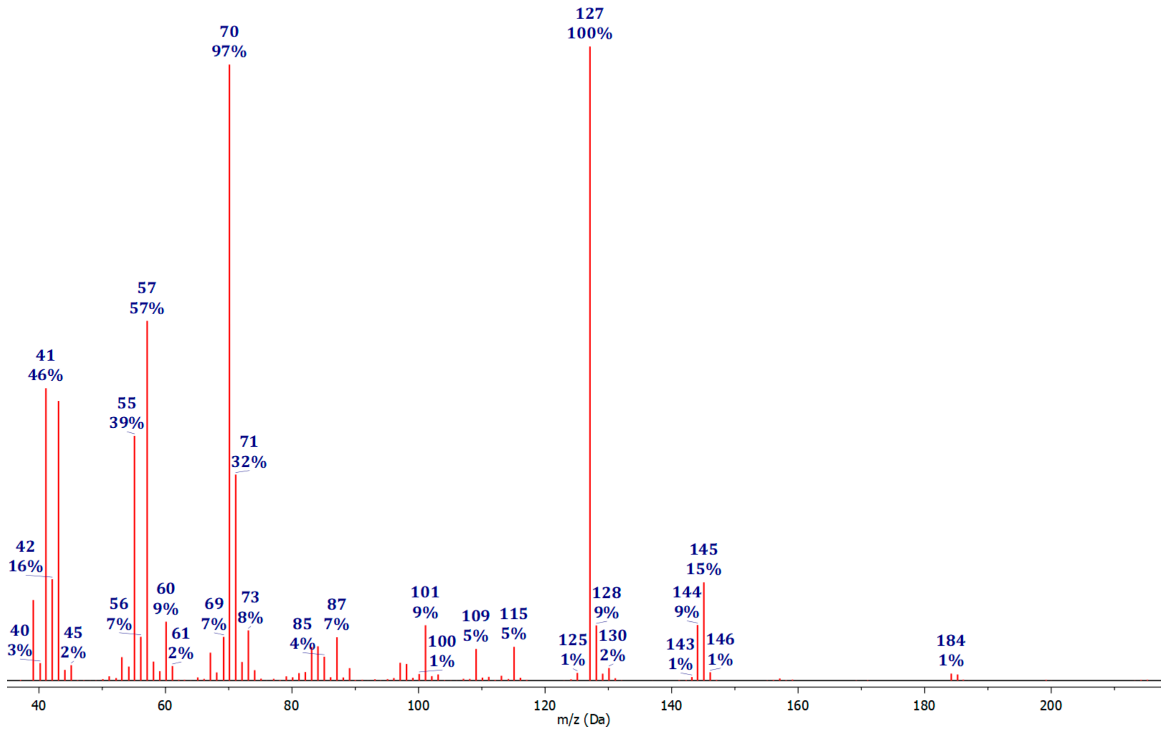

Figure S104. Mass spectrum of 2-methylbutyl octanoate (**2n**)

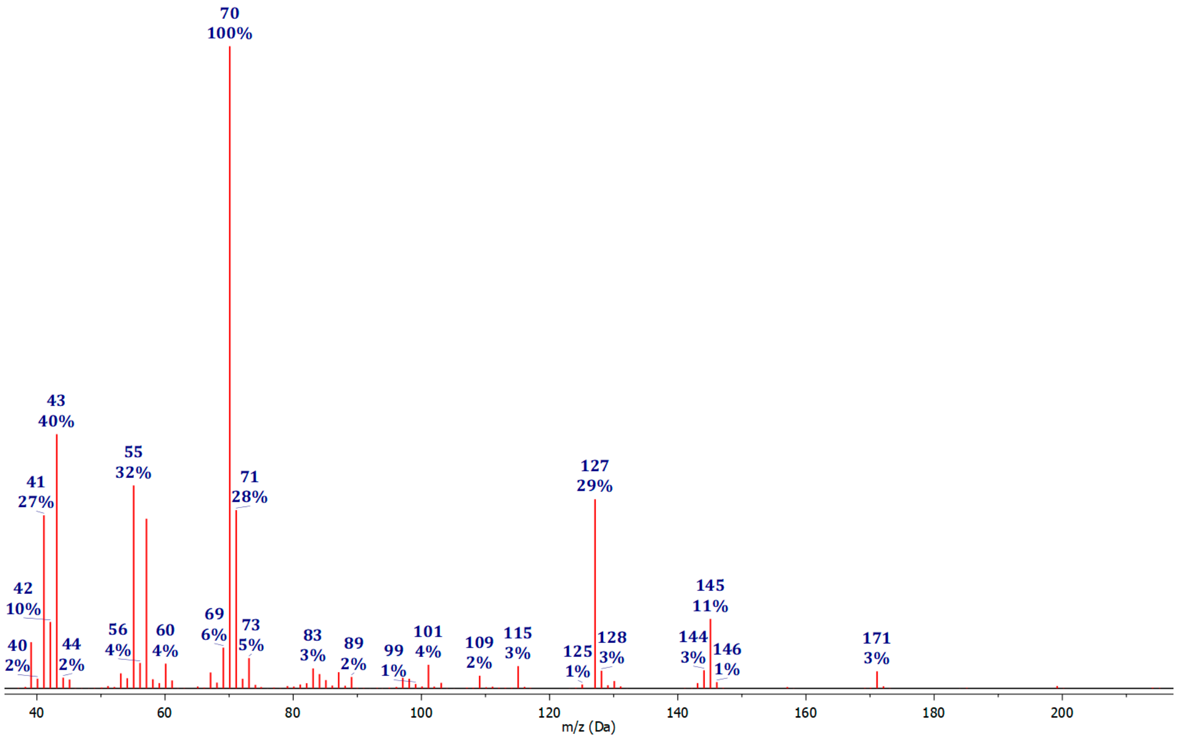

Figure S105. Mass spectrum of 3-methylbutyl octanoate (**3n**)

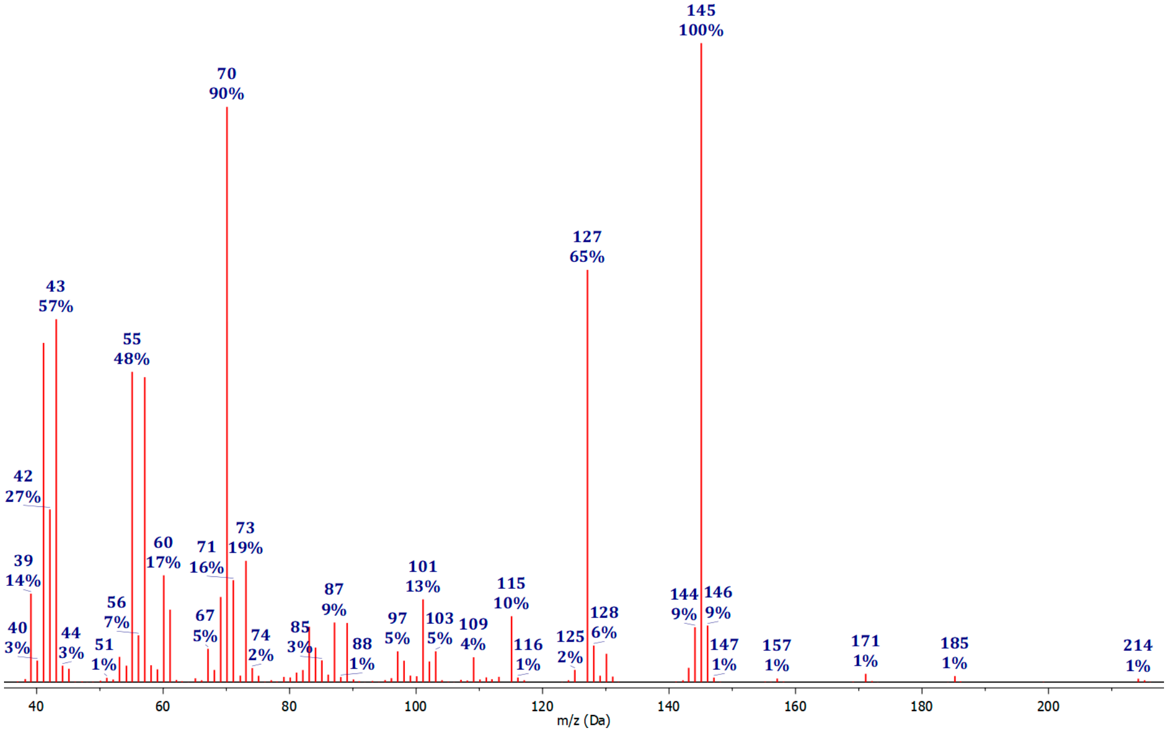

Figure S106. Mass spectrum of pentyl octanoate (**4n**)

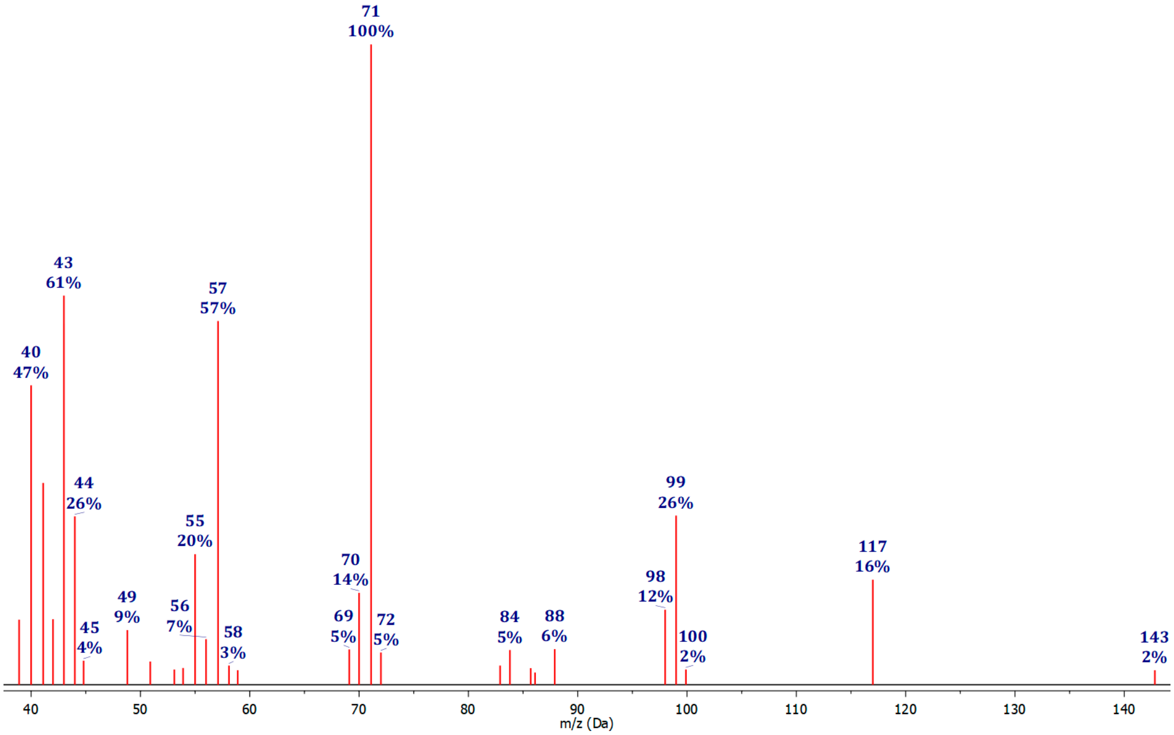

Figure S107. Mass spectrum of 1-methylhexyl 2,2-dimethylbutanoate (**11b**)

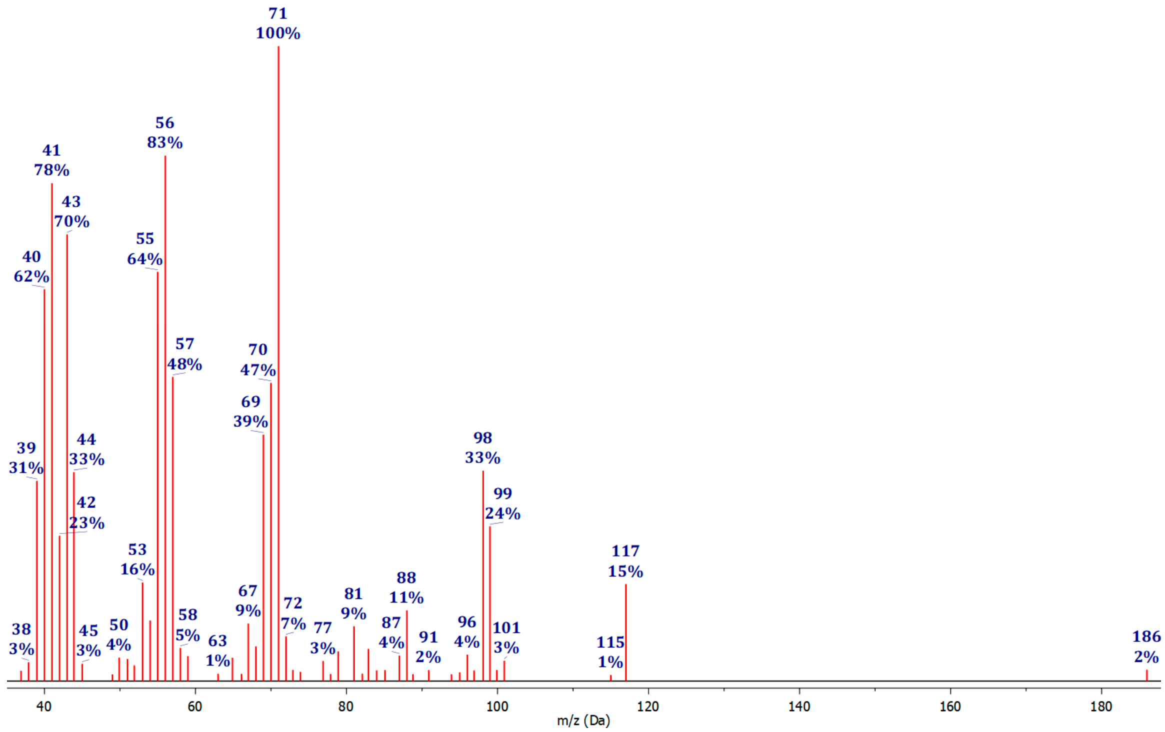

Figure S108. Mass spectrum of 2-methylhexyl 2,2-dimethylbutanoate (**12b**)

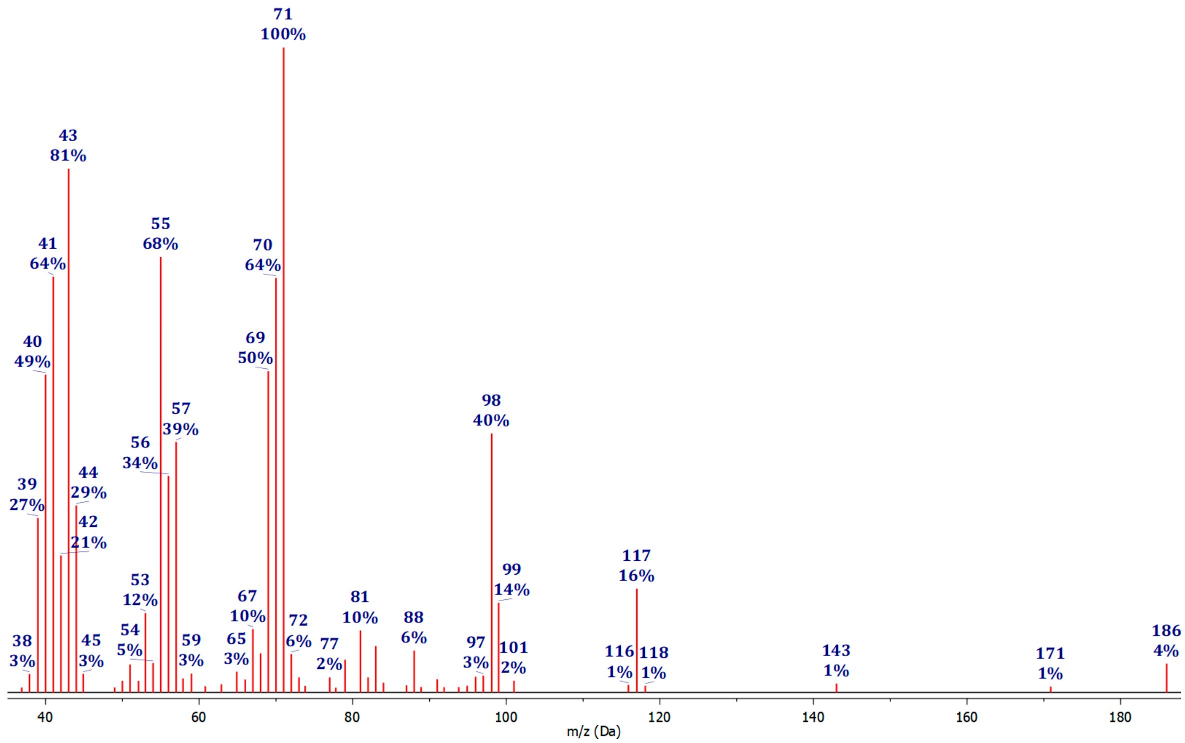

Figure S109. Mass spectrum of 3-methylhexyl 2,2-dimethylbutanoate (**13b**)

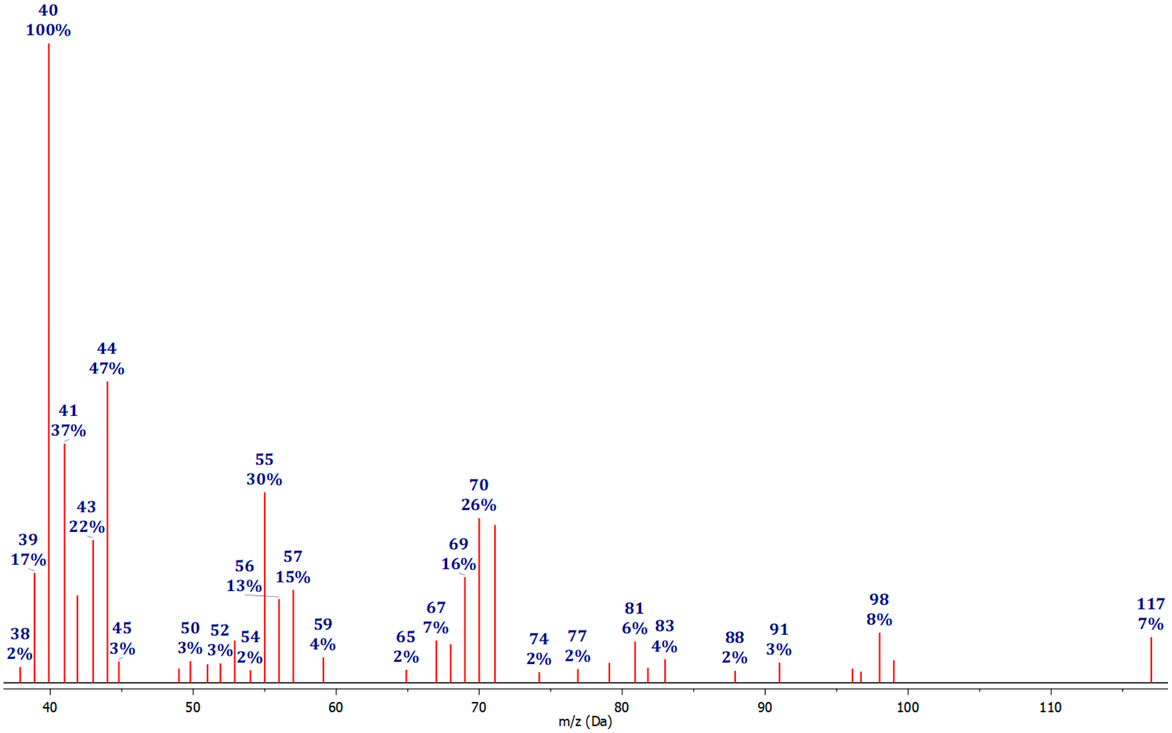

Figure S110. Mass spectrum of 4-methylhexyl 2,2-dimethylbutanoate (**14b**)

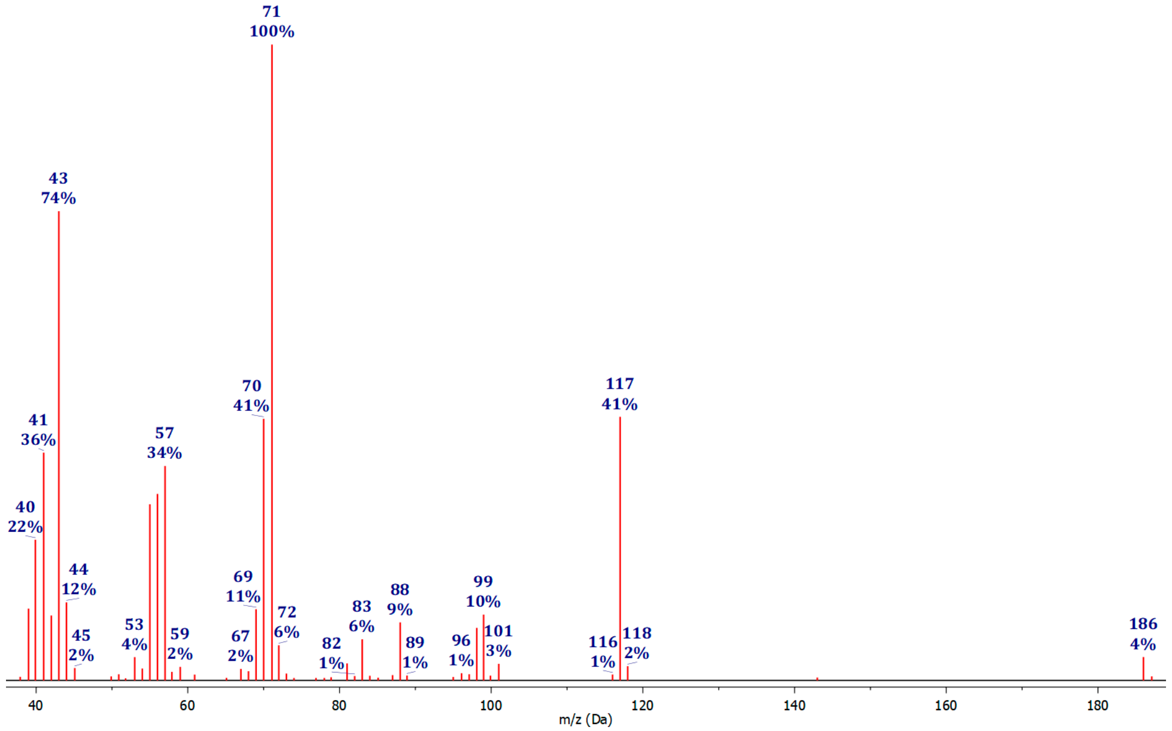

Figure S111. Mass spectrum of 5-methylhexyl 2,2-dimethylbutanoate (**15b**)

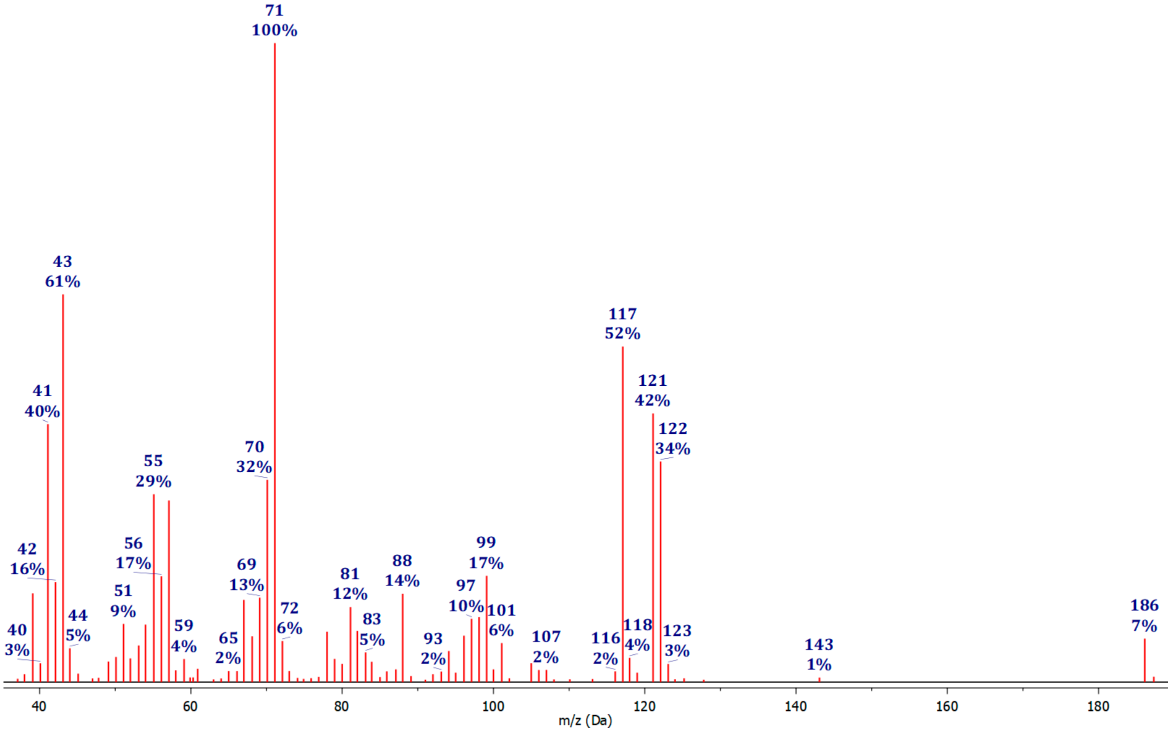

Figure S112. Mass spectrum of heptyl 2,2-dimethylbutanoate (**16b**)

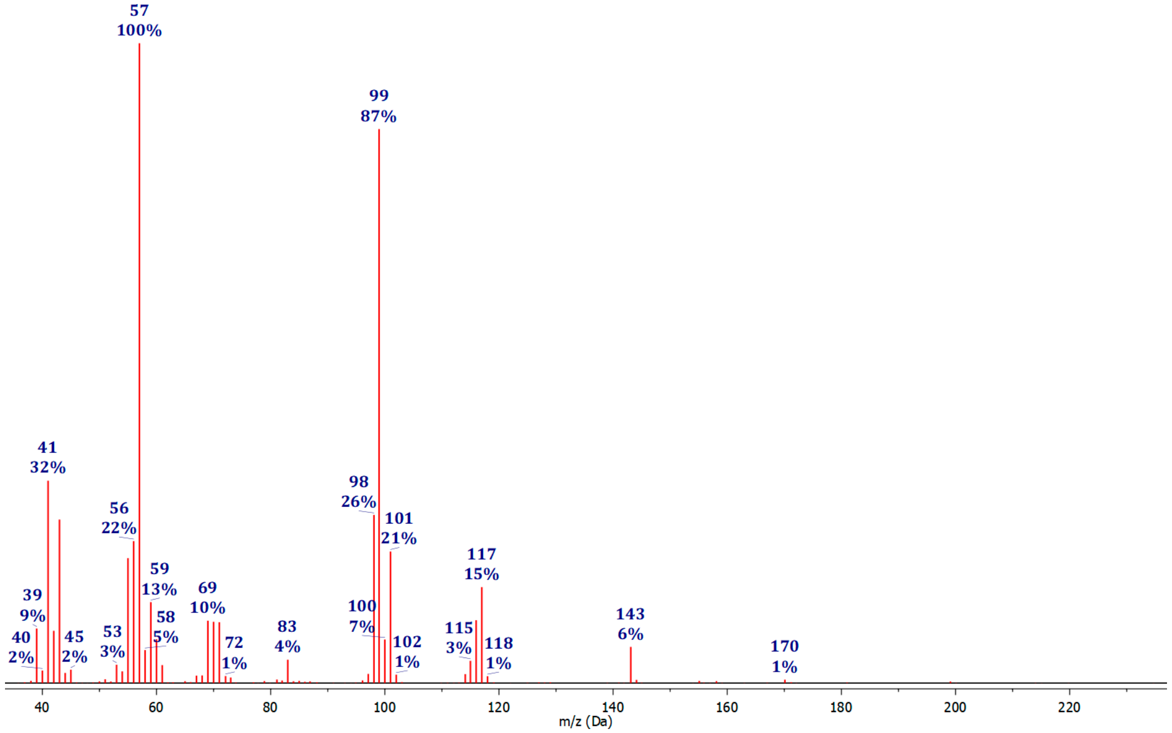

Figure S113. Mass spectrum of 1-methylhexyl 3,3-dimethylbutanoate (**11c**)

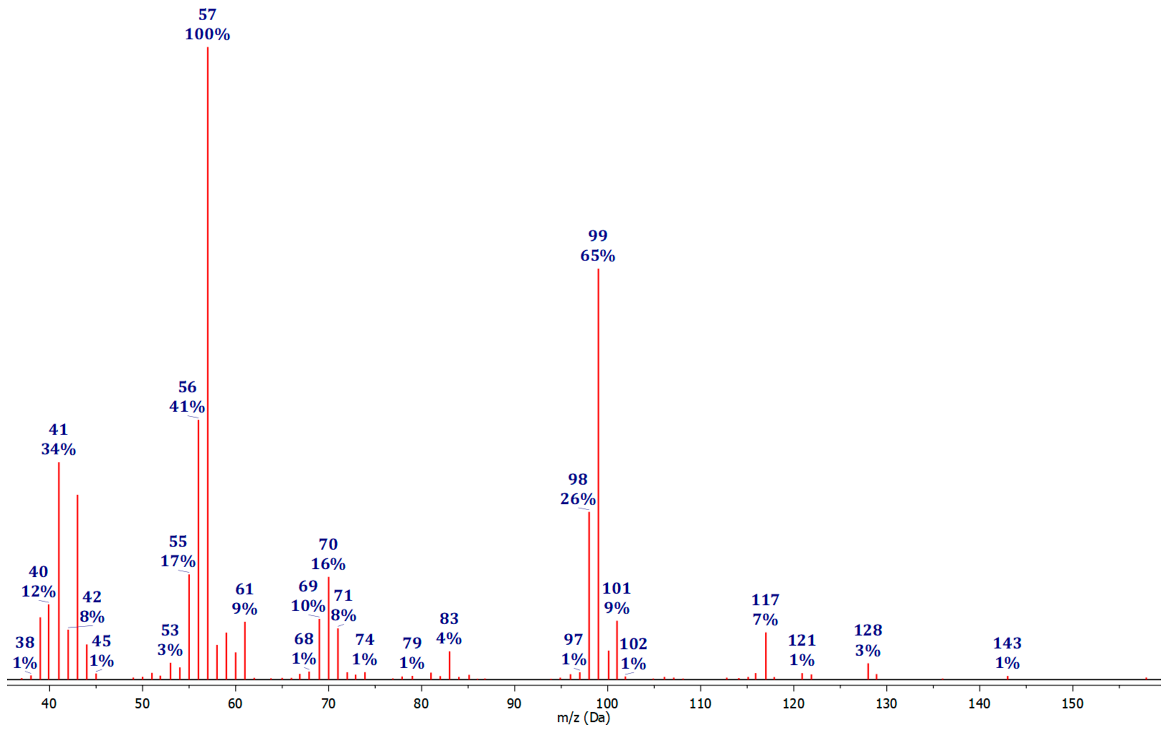

Figure S114. Mass spectrum of 2-methylhexyl 3,3-dimethylbutanoate (**12c**)

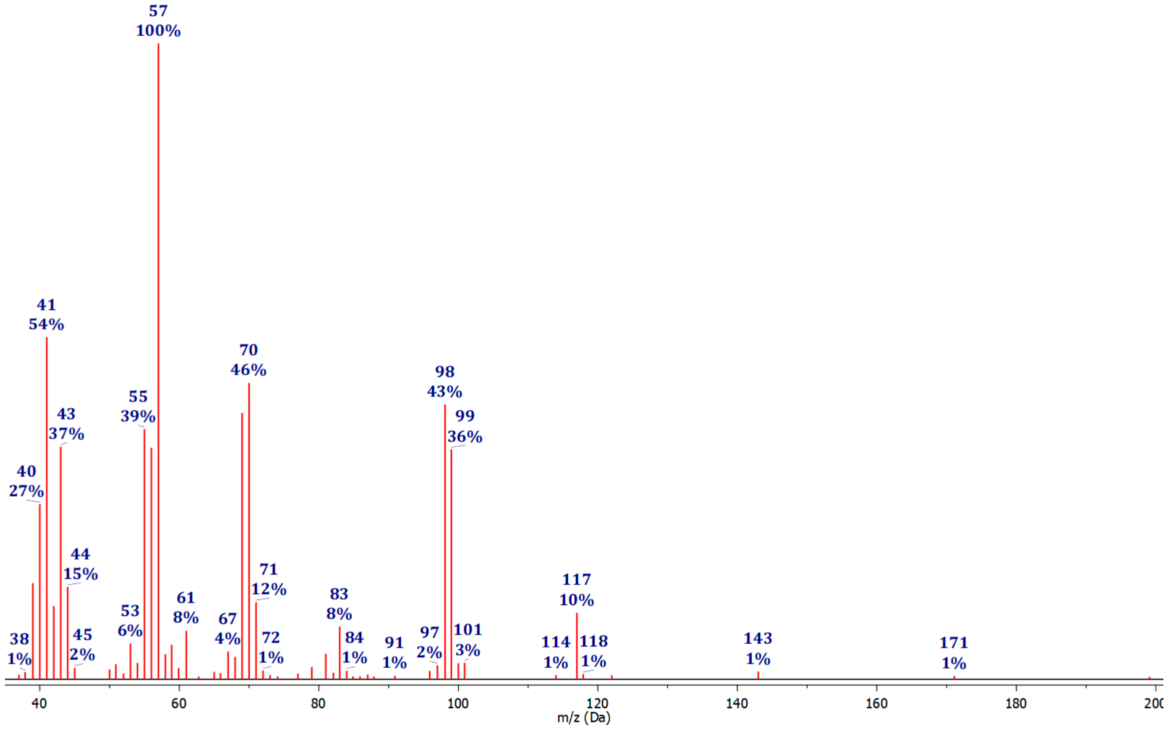

Figure S115. Mass spectrum of 3-methylhexyl 3,3-dimethylbutanoate (**13c**)

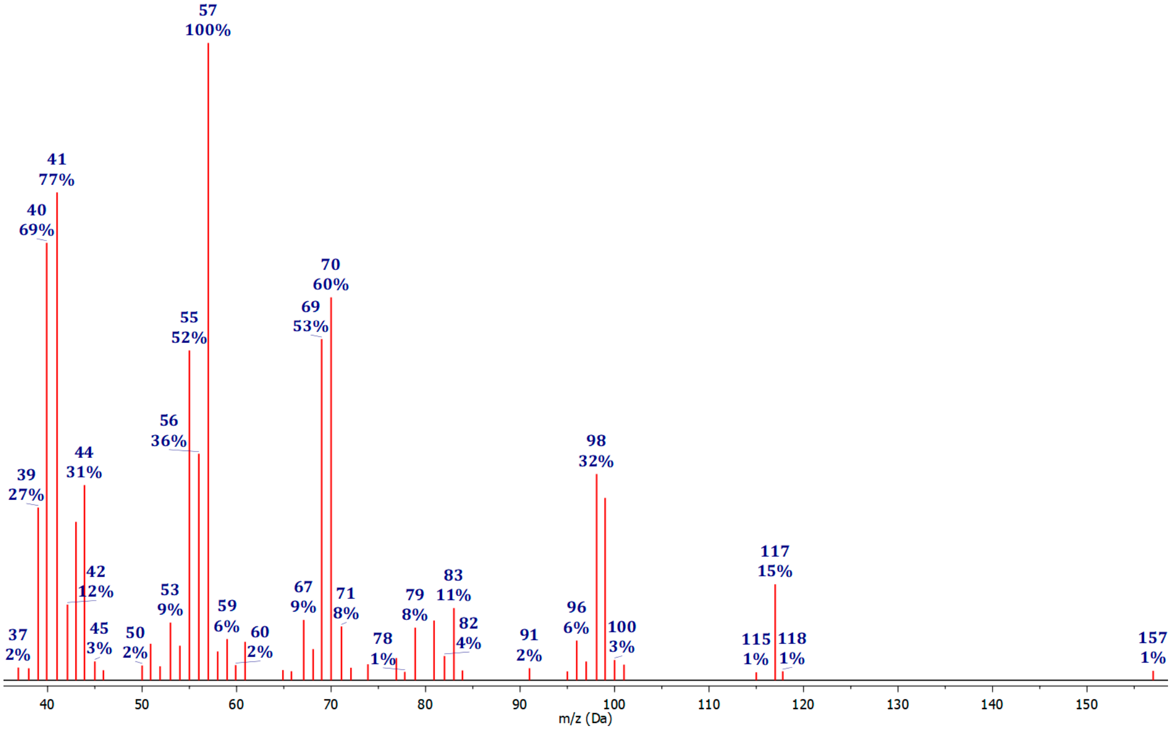

Figure S116. Mass spectrum of 4-methylhexyl 3,3-dimethylbutanoate (**14c**)

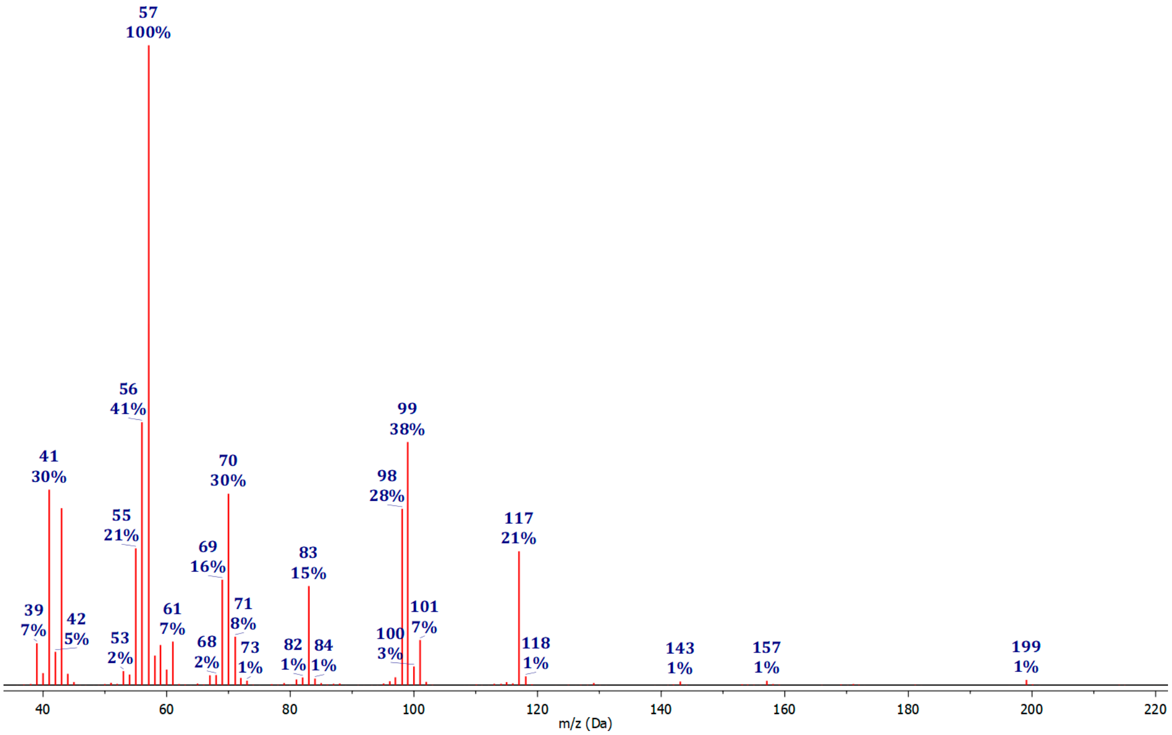

Figure S117. Mass spectrum of 5-methylhexyl 3,3-dimethylbutanoate (**15c**)

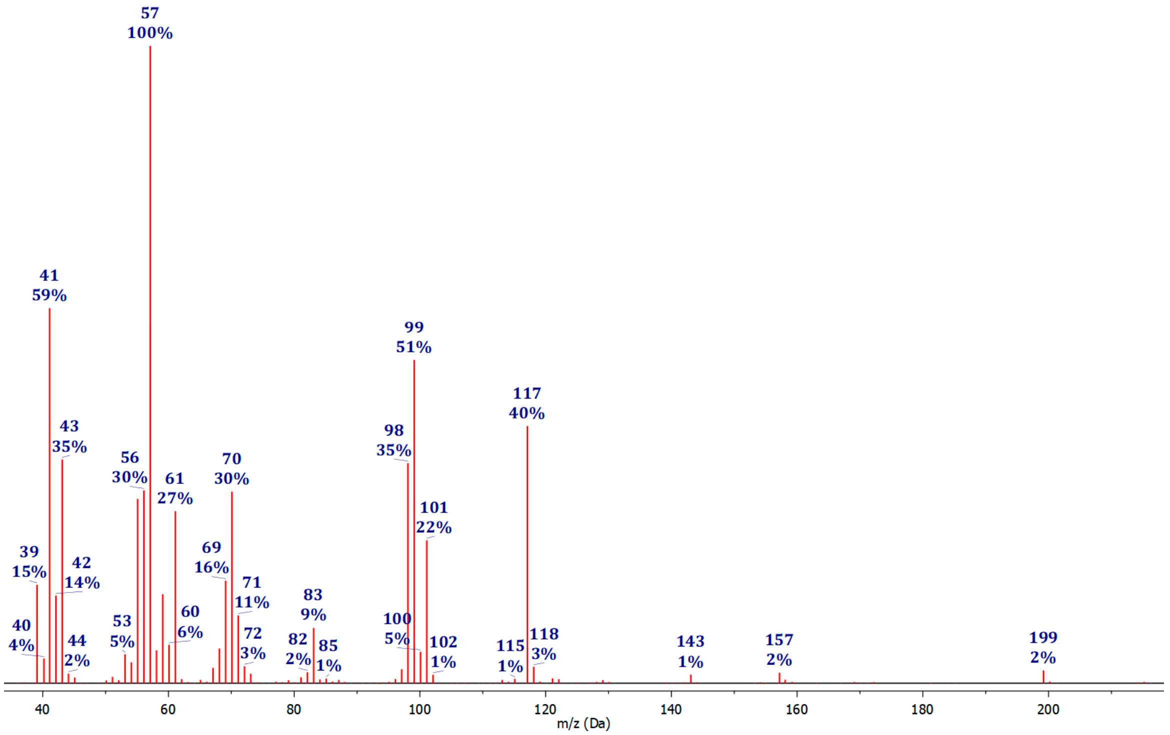

Figure S118. Mass spectrum of heptyl 3,3-dimethylbutanoate (**16c**)

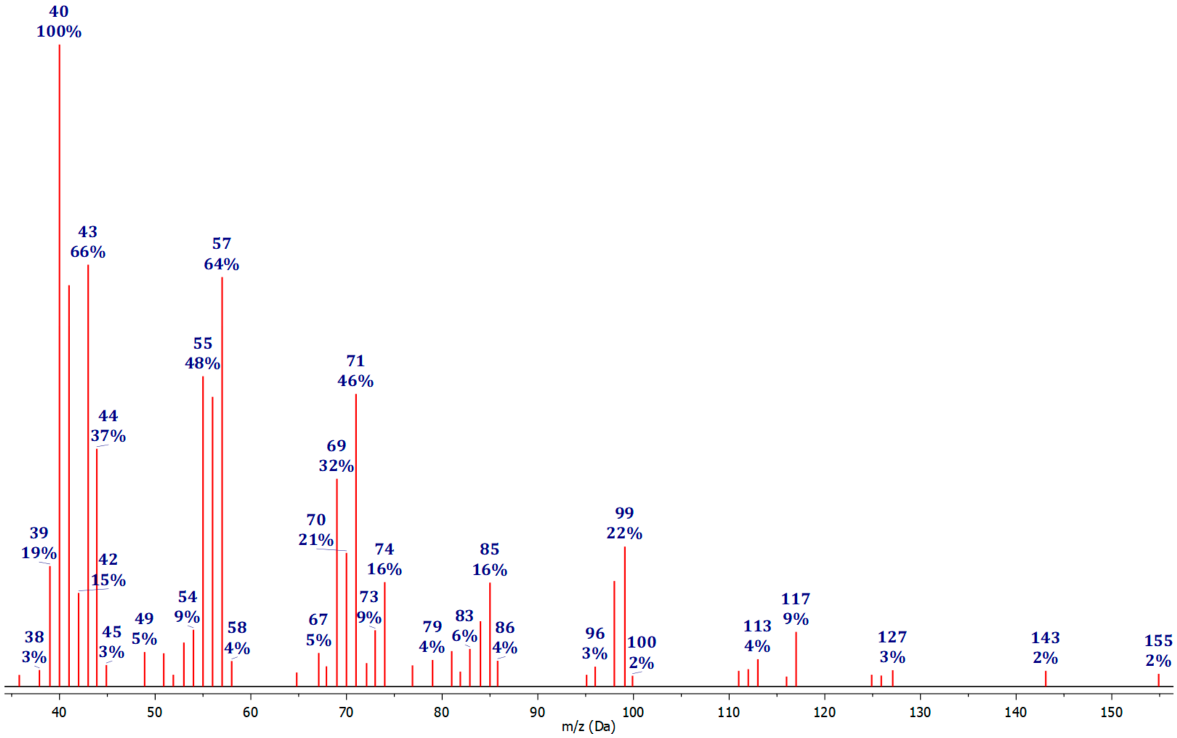

Figure S119. Mass spectrum of 1-methylhexyl 2,3-dimethylbutanoate (**11d**)

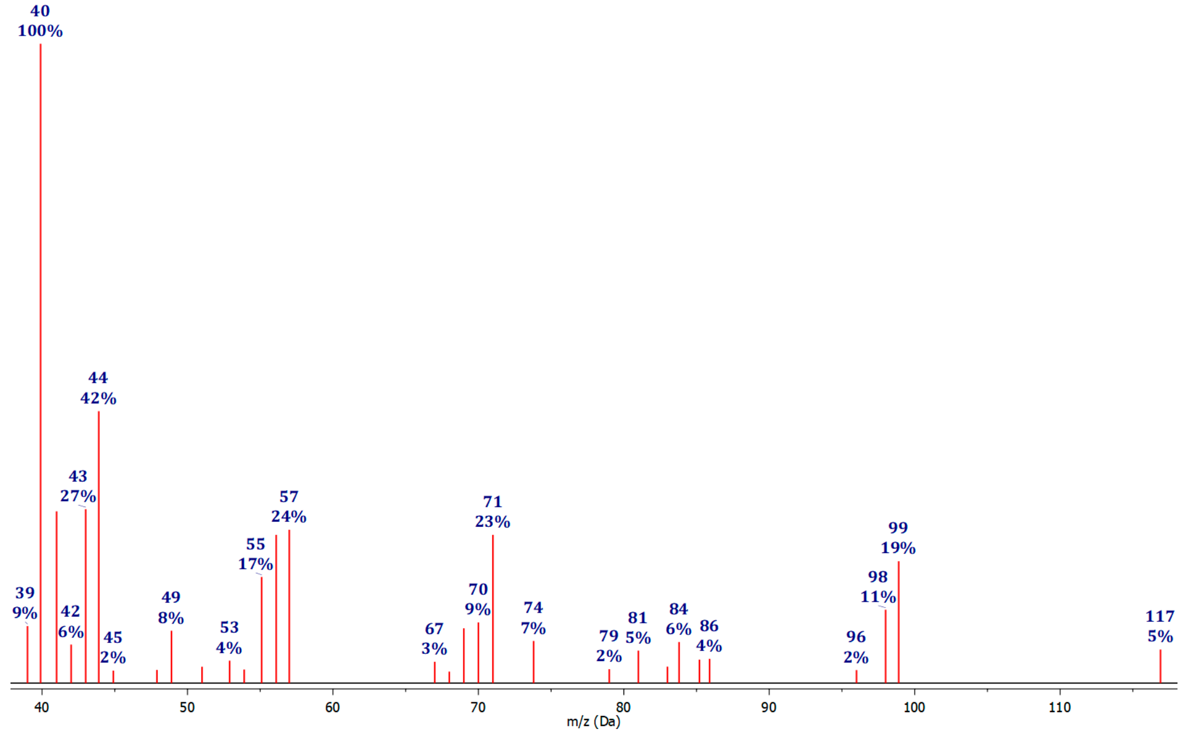

Figure S120. Mass spectrum of 2-methylhexyl 2,3-dimethylbutanoate (**12d**)

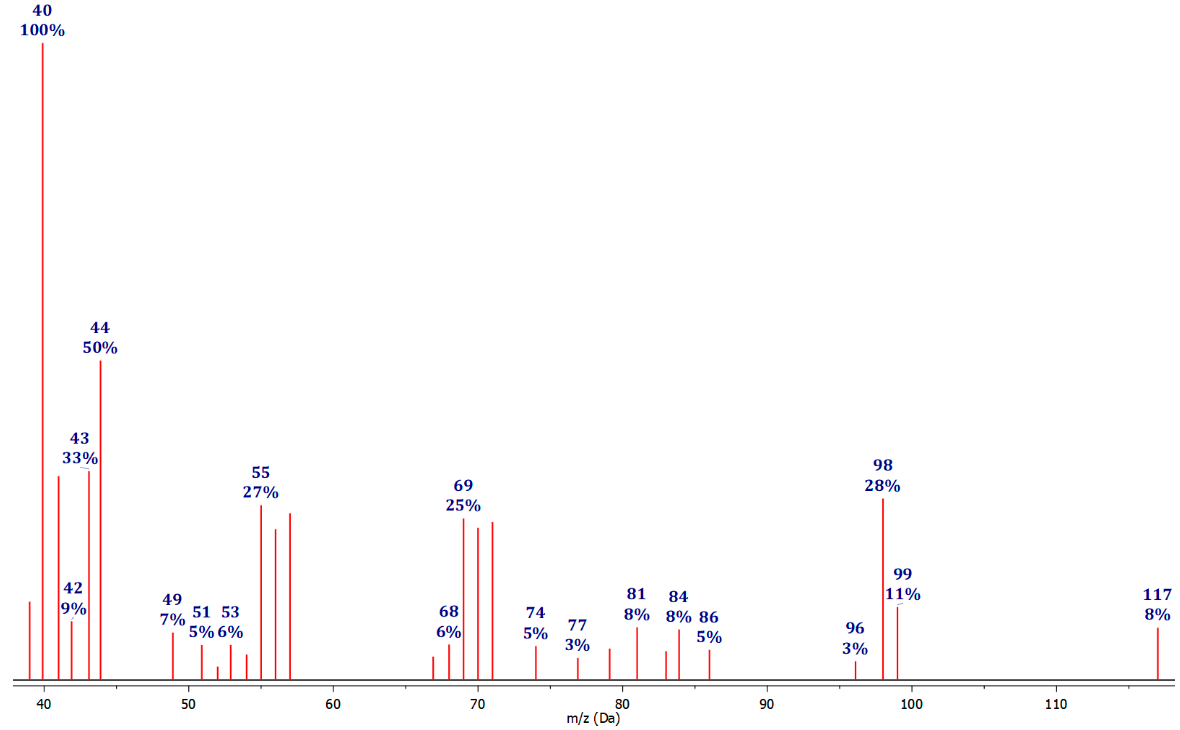

Figure S121. Mass spectrum of 3-methylhexyl 2,3-dimethylbutanoate (**13d**)

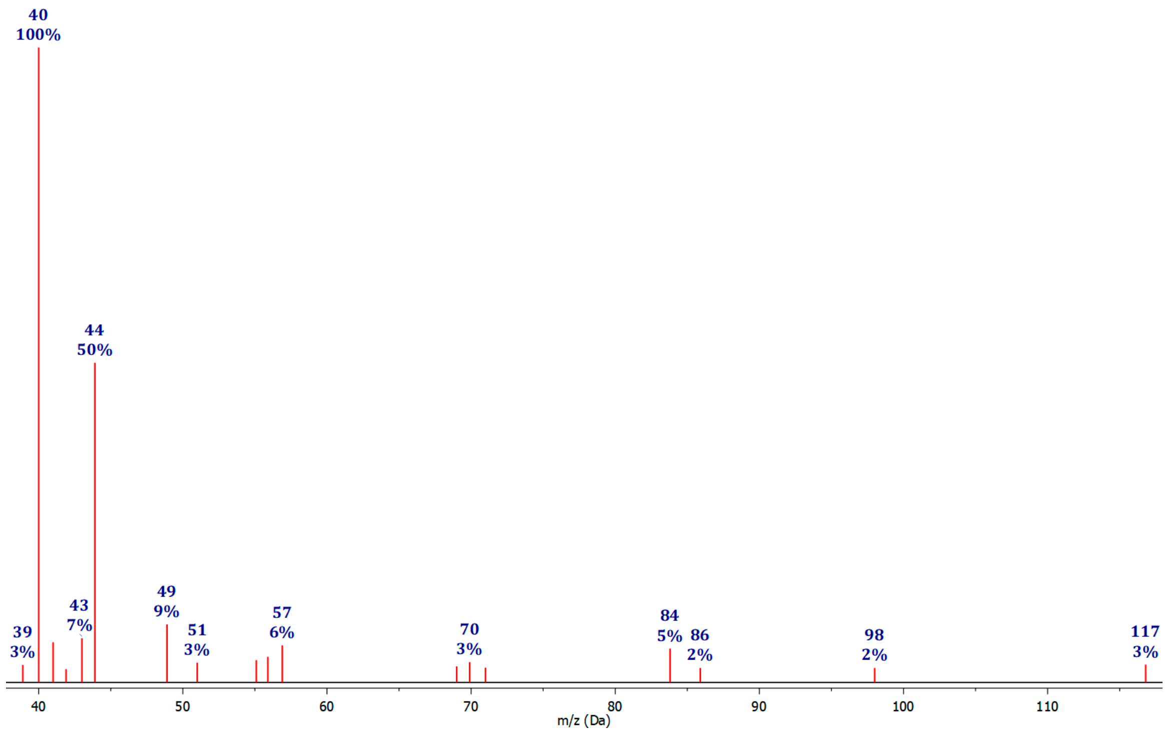

Figure S122. Mass spectrum of 4-methylhexyl 2,3-dimethylbutanoate (**14d**)

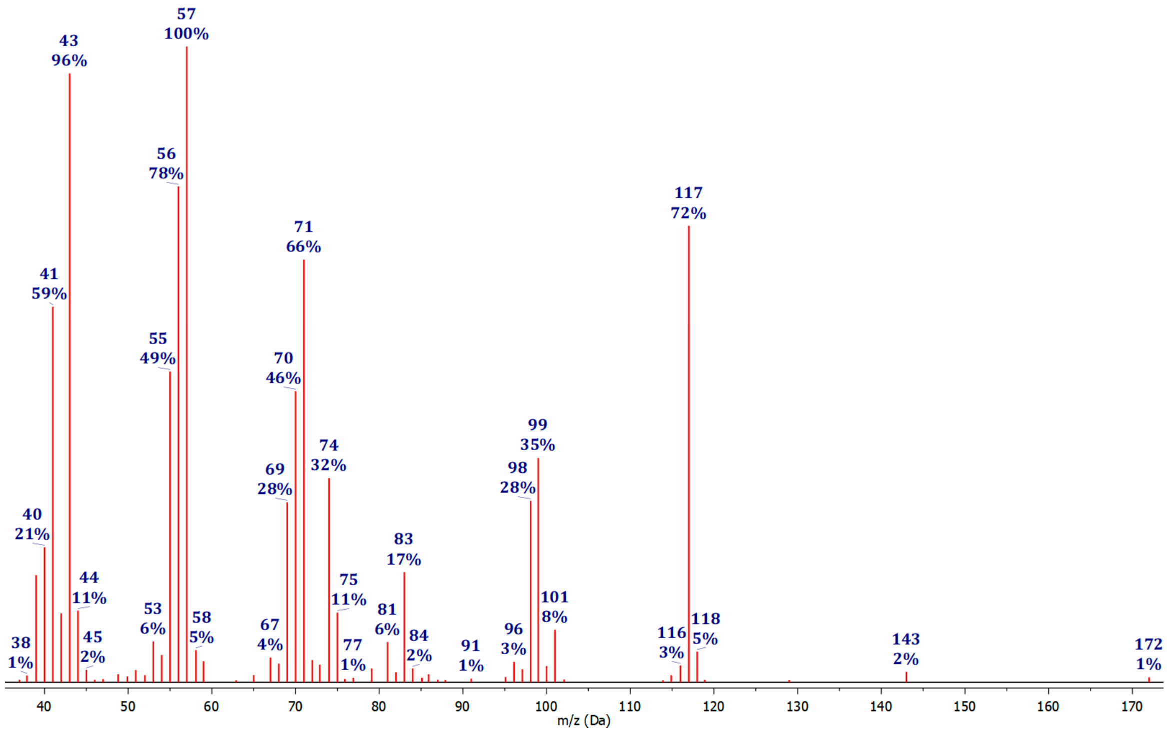

Figure S123. Mass spectrum of 5-methylhexyl 2,3-dimethylbutanoate (**15d**)

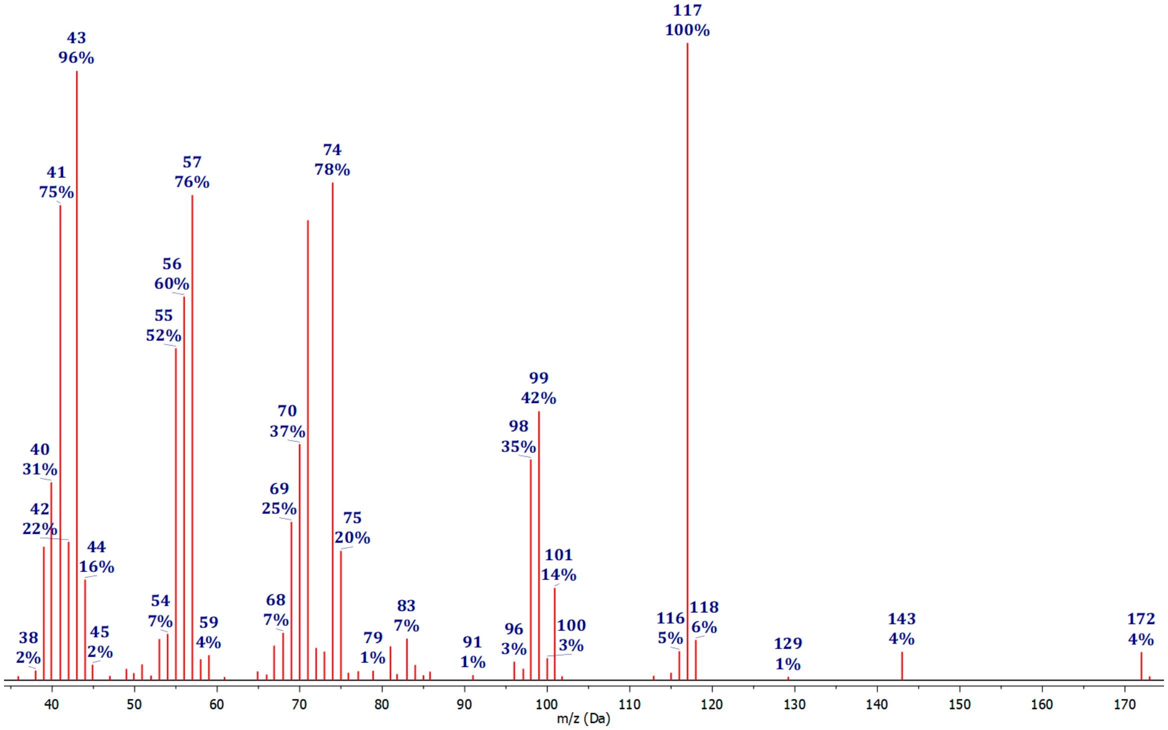

Figure S124. Mass spectrum of heptyl 2,3-dimethylbutanoate (**16d**)

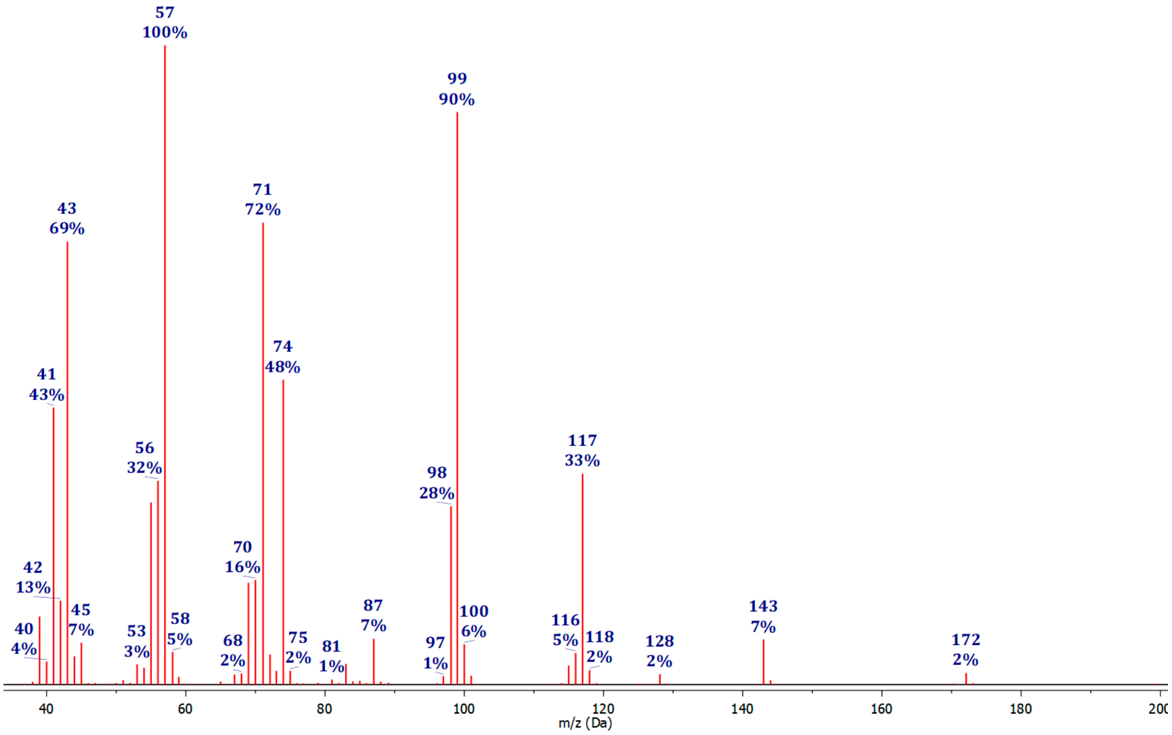

Figure S125a. Mass spectrum of 1-methylhexyl 2-methylpentanoate (**11e epimer I**)

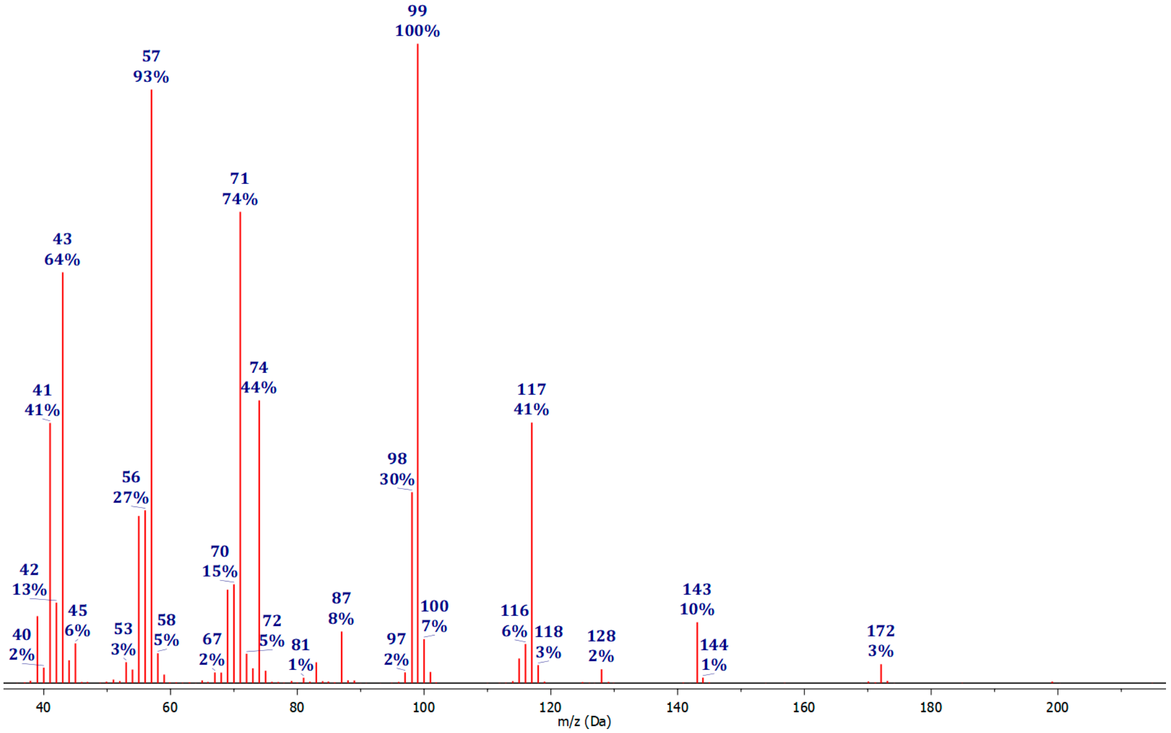

Figure S125b. Mass spectrum of 1-methylhexyl 2-methylpentanoate (**11e epimer II**)

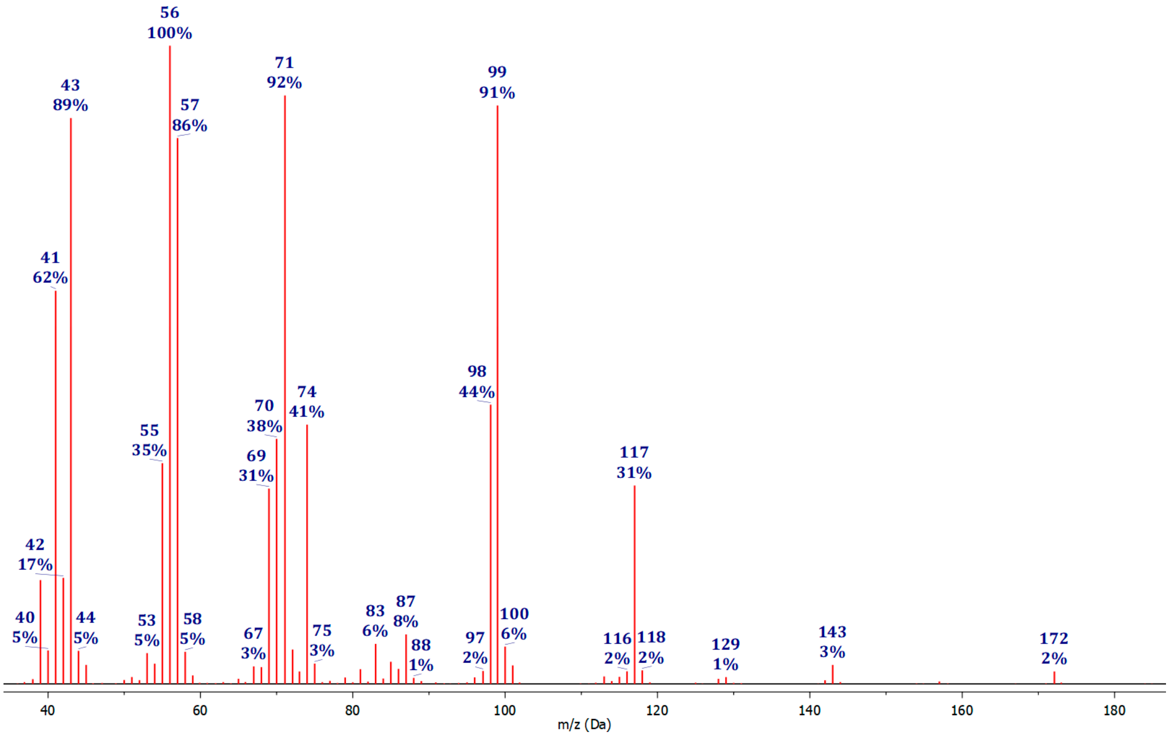

Figure S126. Mass spectrum of 2-methylhexyl 2-methylpentanoate (**12e**)

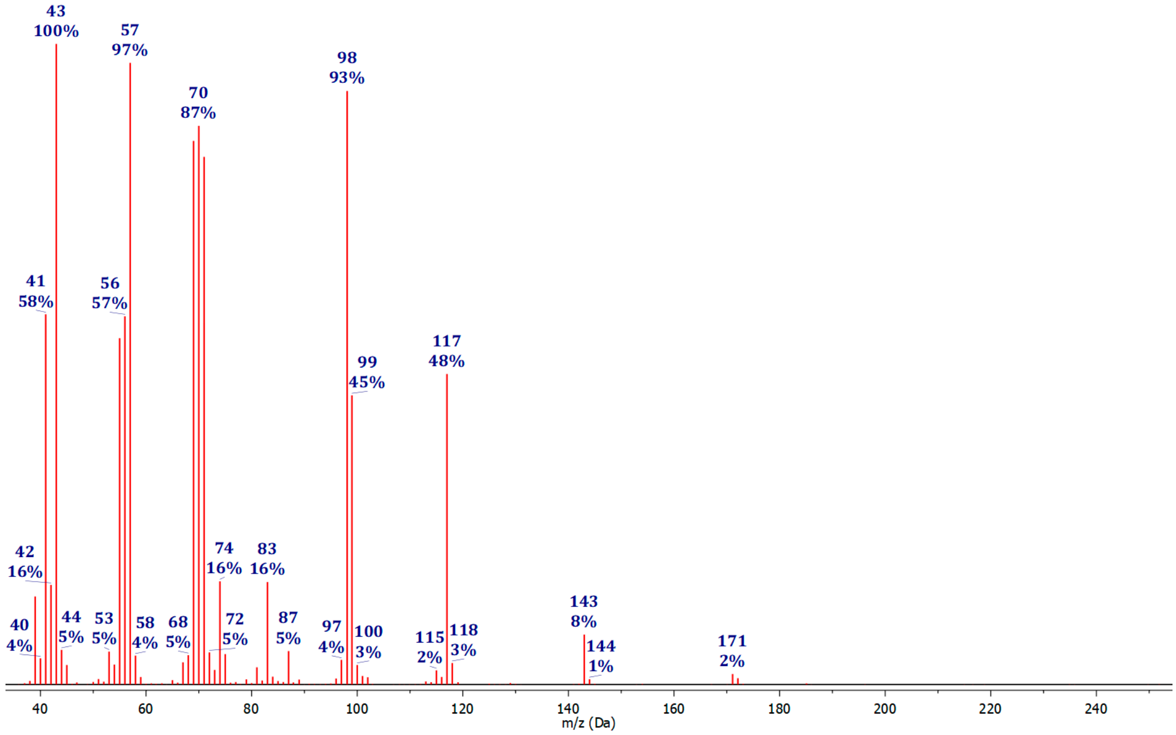

Figure S127. Mass spectrum of 3-methylhexyl 2-methylpentanoate (**13e**)

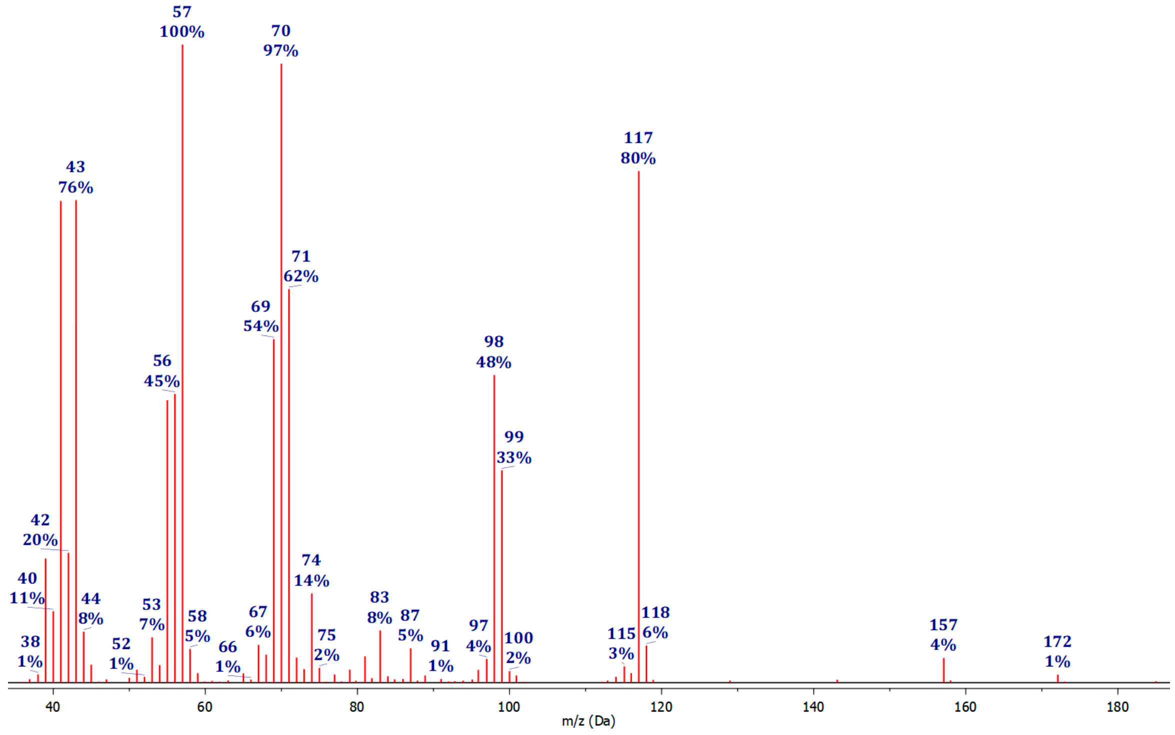

Figure S128. Mass spectrum of 4-methylhexyl 2-methylpentanoate (**14e**)

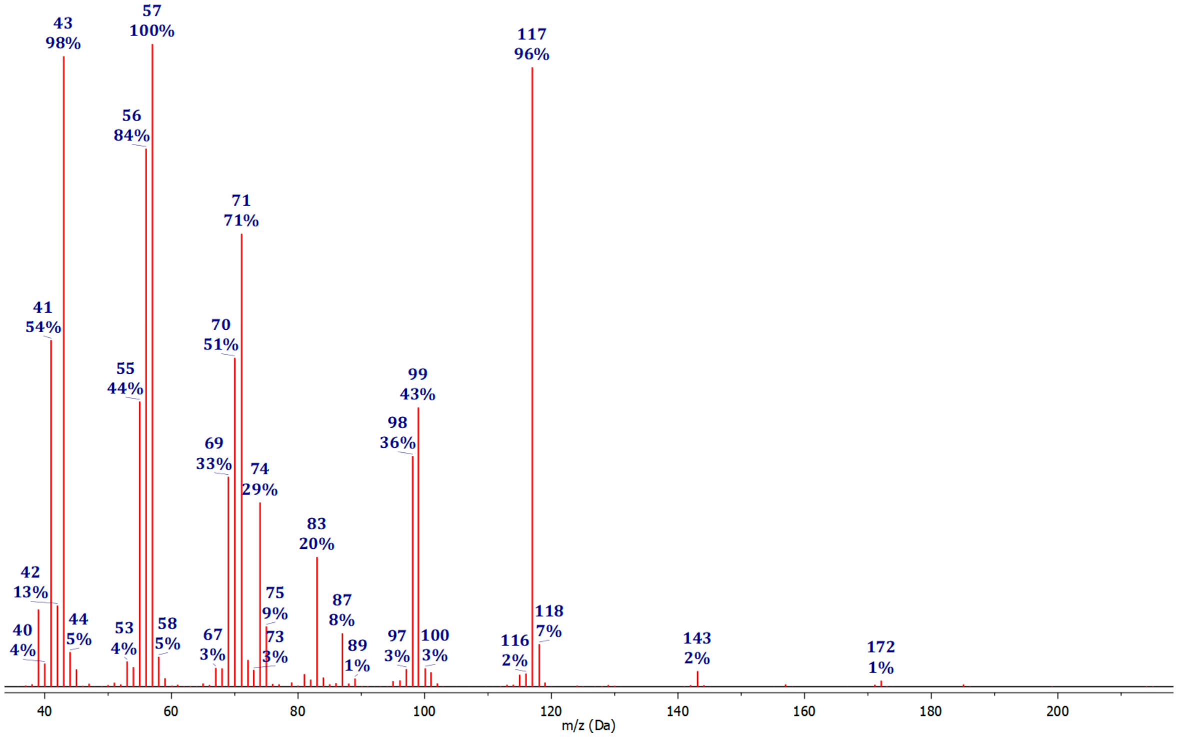

Figure S129. Mass spectrum of 5-methylhexyl 2-methylpentanoate (**15e**)

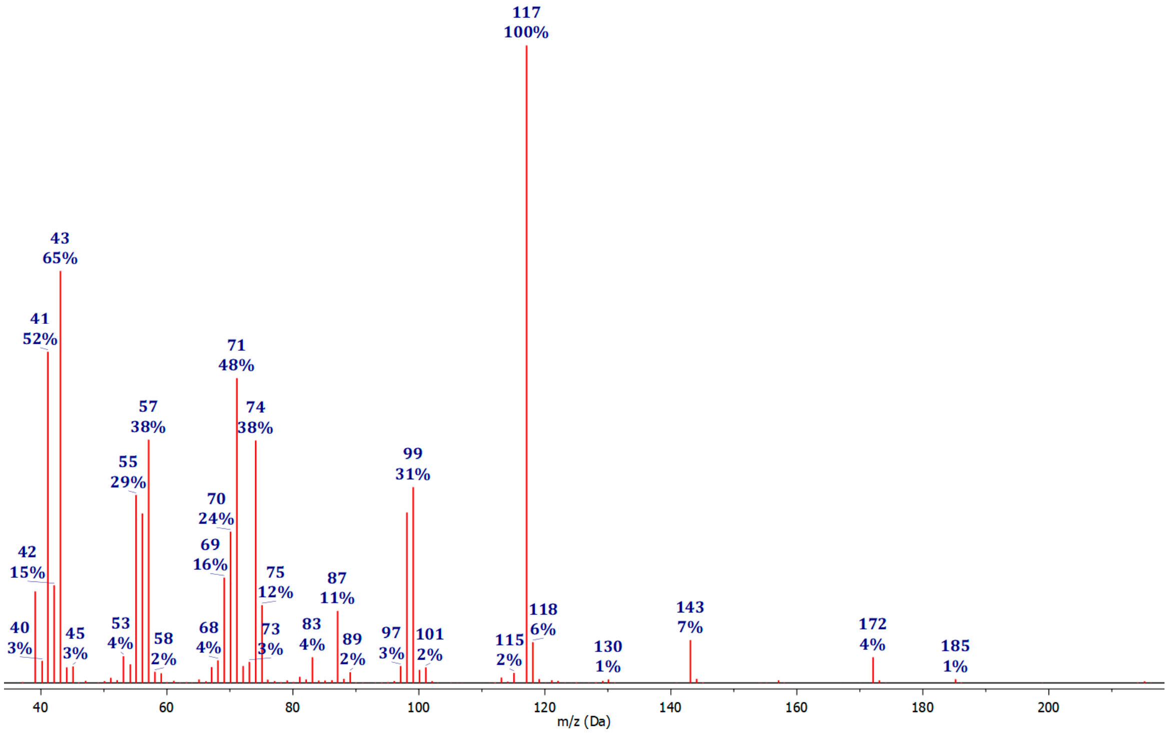

Figure S130. Mass spectrum of heptyl 2-methylpentanoate (**16e**)

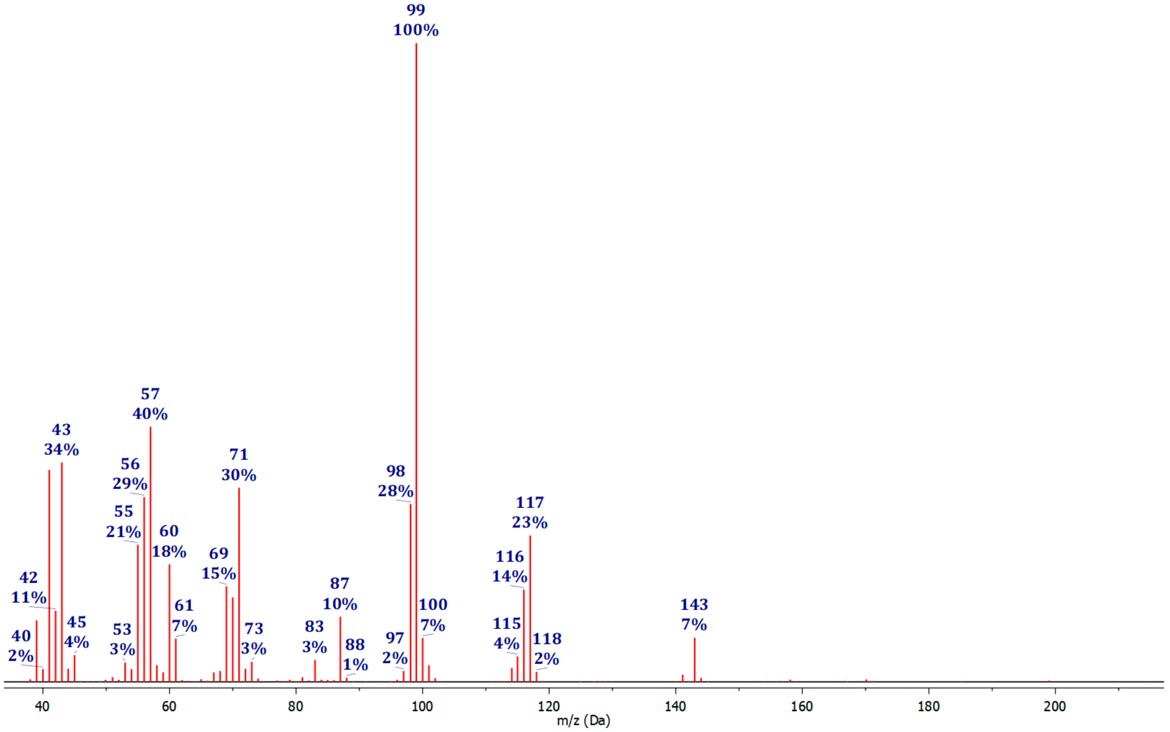

Figure S131. Mass spectrum of 1-methylhexyl 3-methylpentanoate (**11f**)

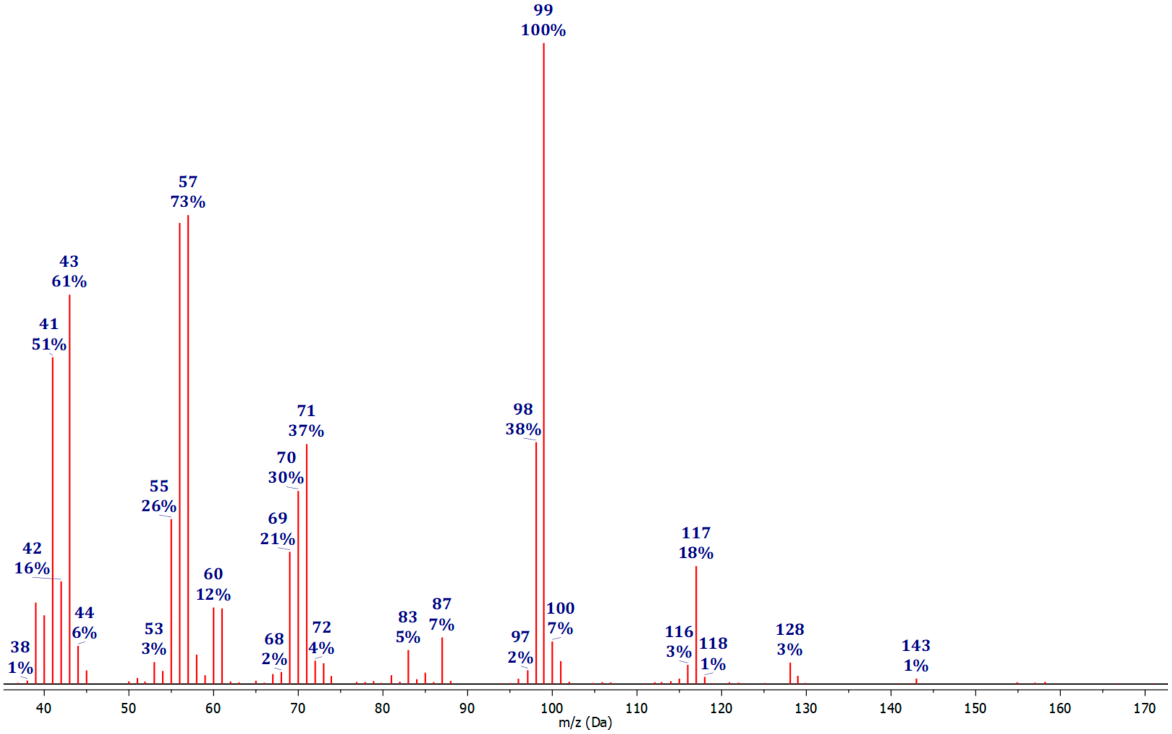

Figure S132. Mass spectrum of 2-methylhexyl 3-methylpentanoate (**12f**)

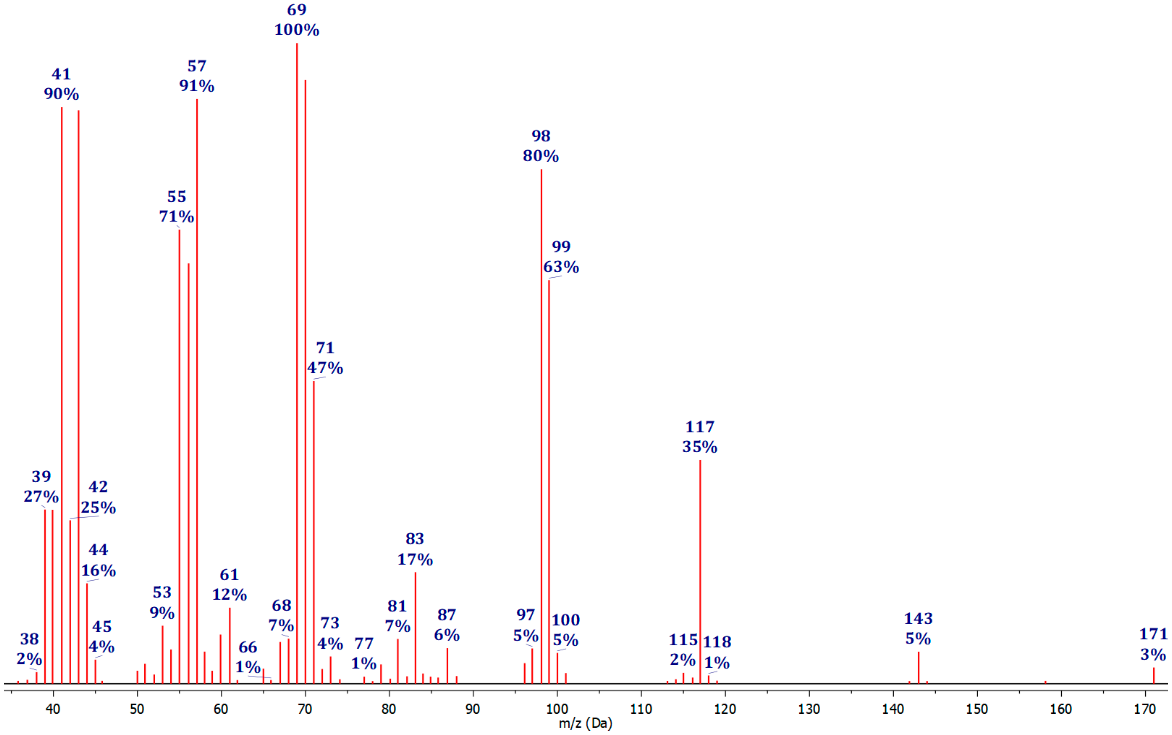

Figure S133. Mass spectrum of 3-methylhexyl 3-methylpentanoate (**13f**)

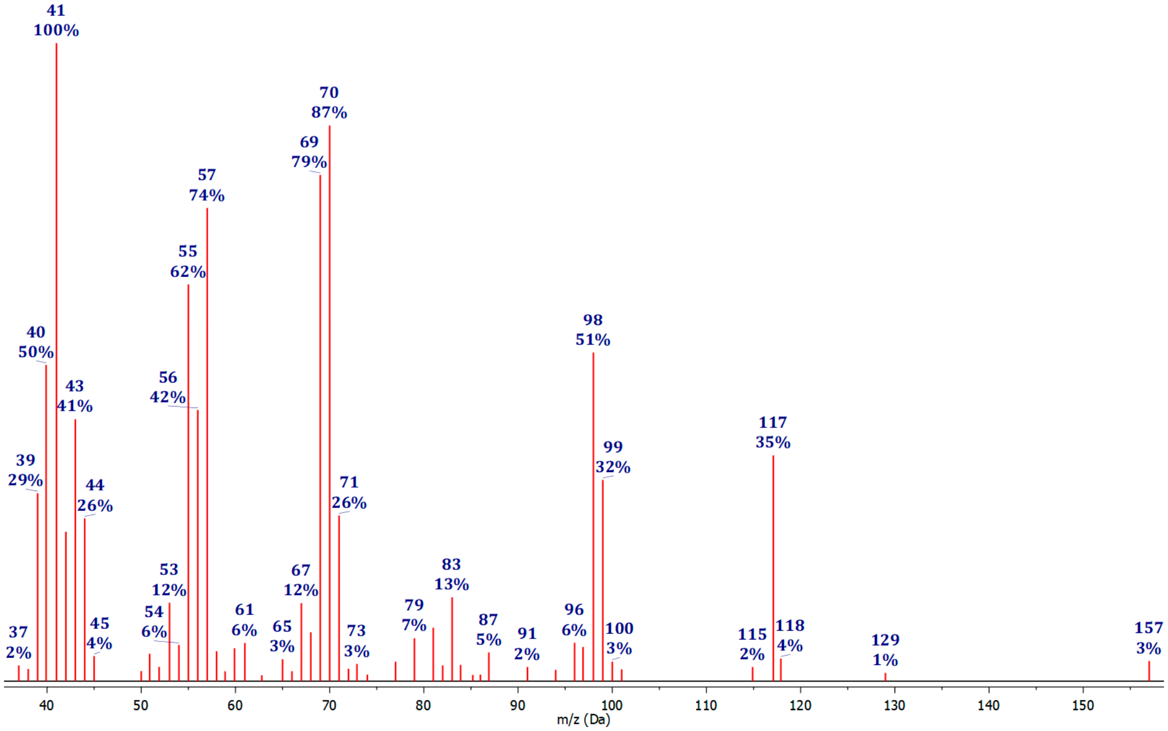

Figure S134. Mass spectrum of 4-methylhexyl 3-methylpentanoate (**14f**)

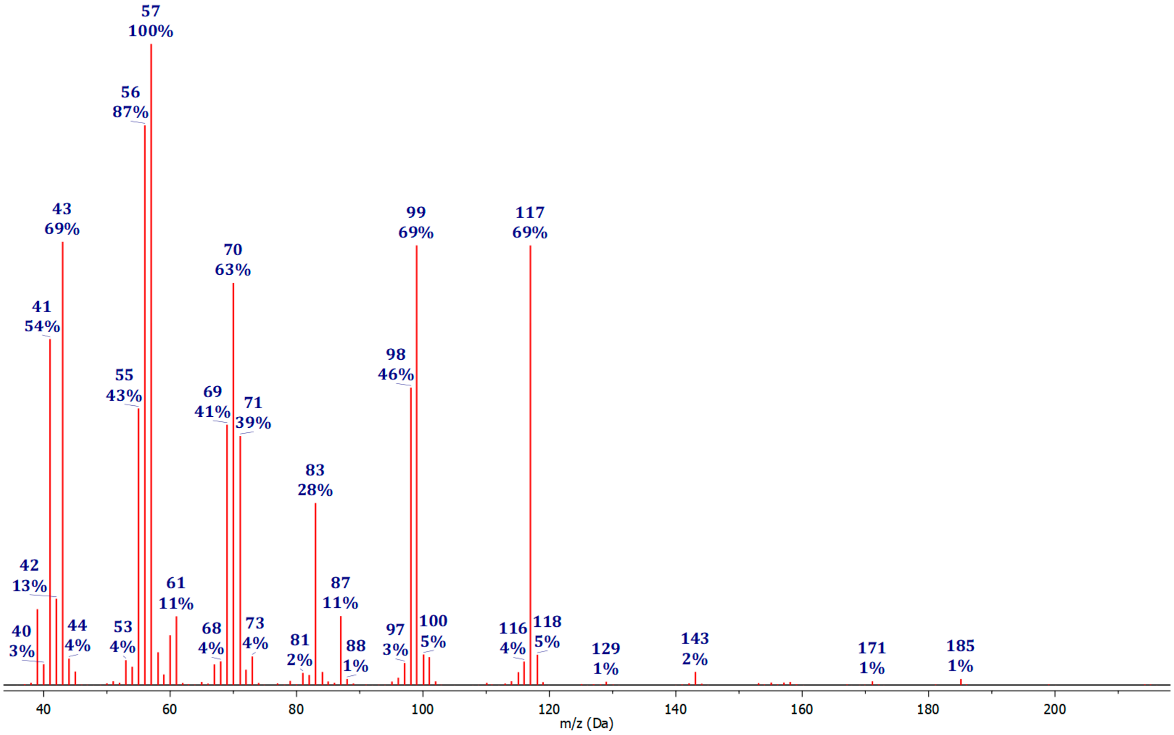

Figure S135. Mass spectrum of 5-methylhexyl 3-methylpentanoate (**15f**)

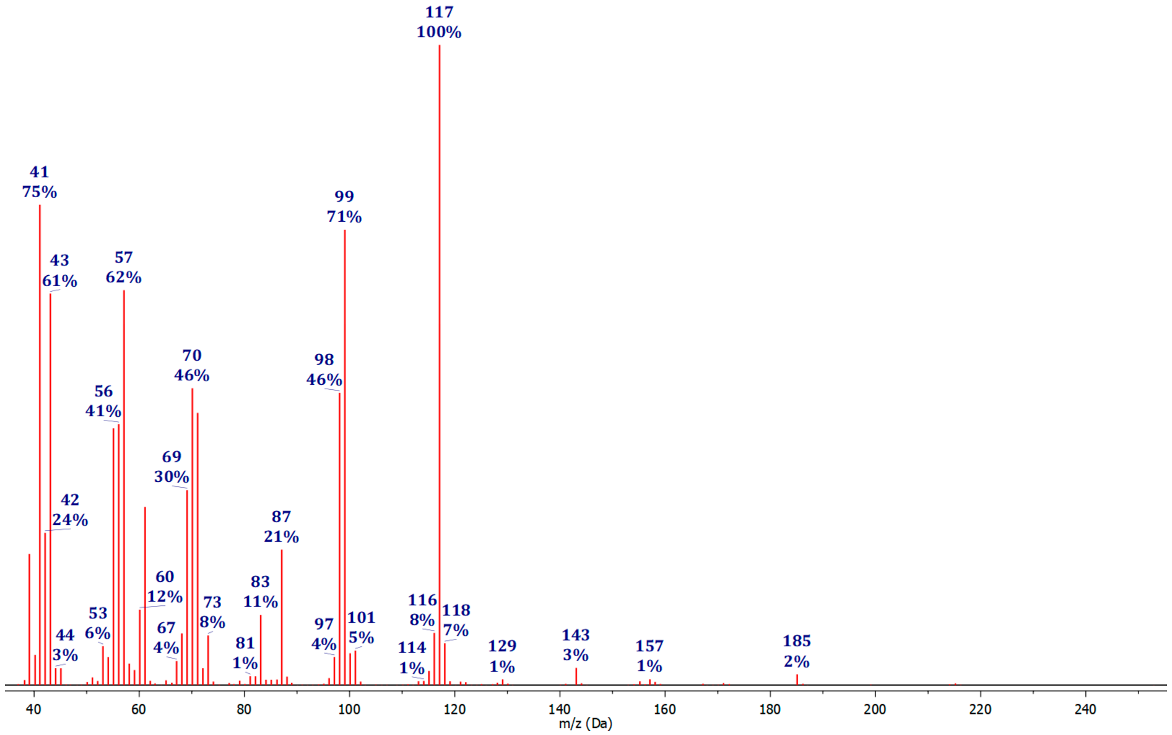

Figure S136. Mass spectrum of heptyl 3-methylpentanoate (**16f**)

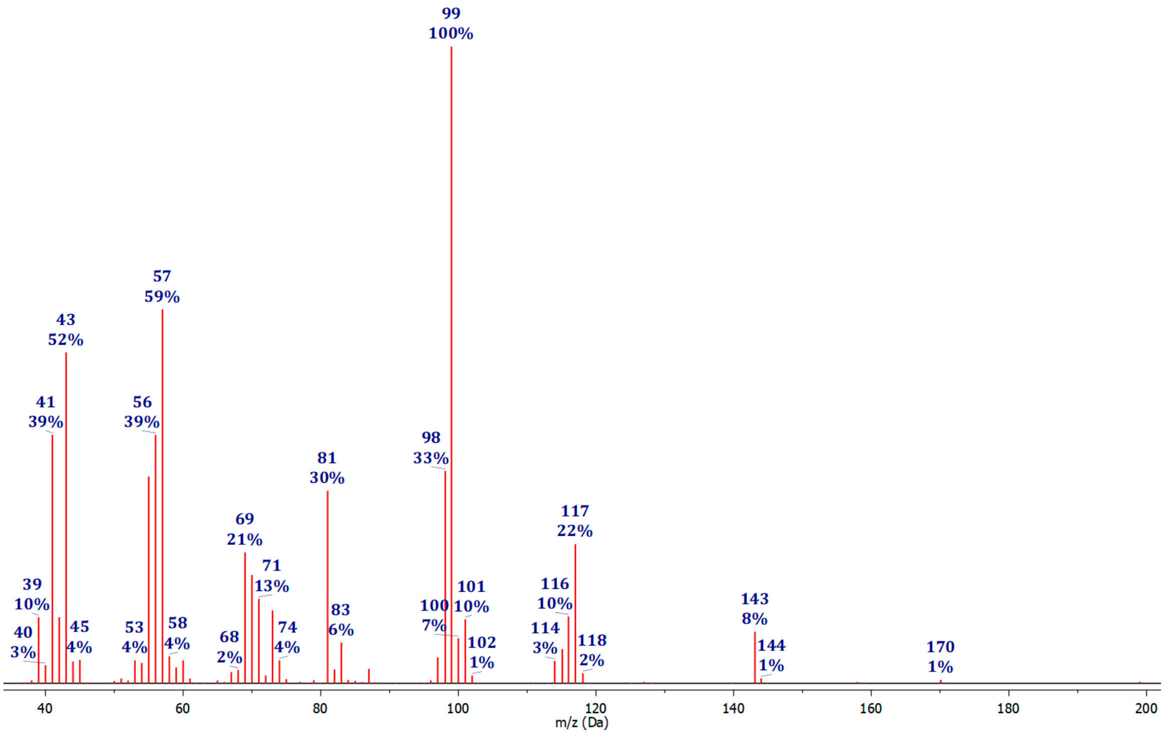

Figure S137. Mass spectrum of 1-methylhexyl 4-methylpentanoate (**11g**)

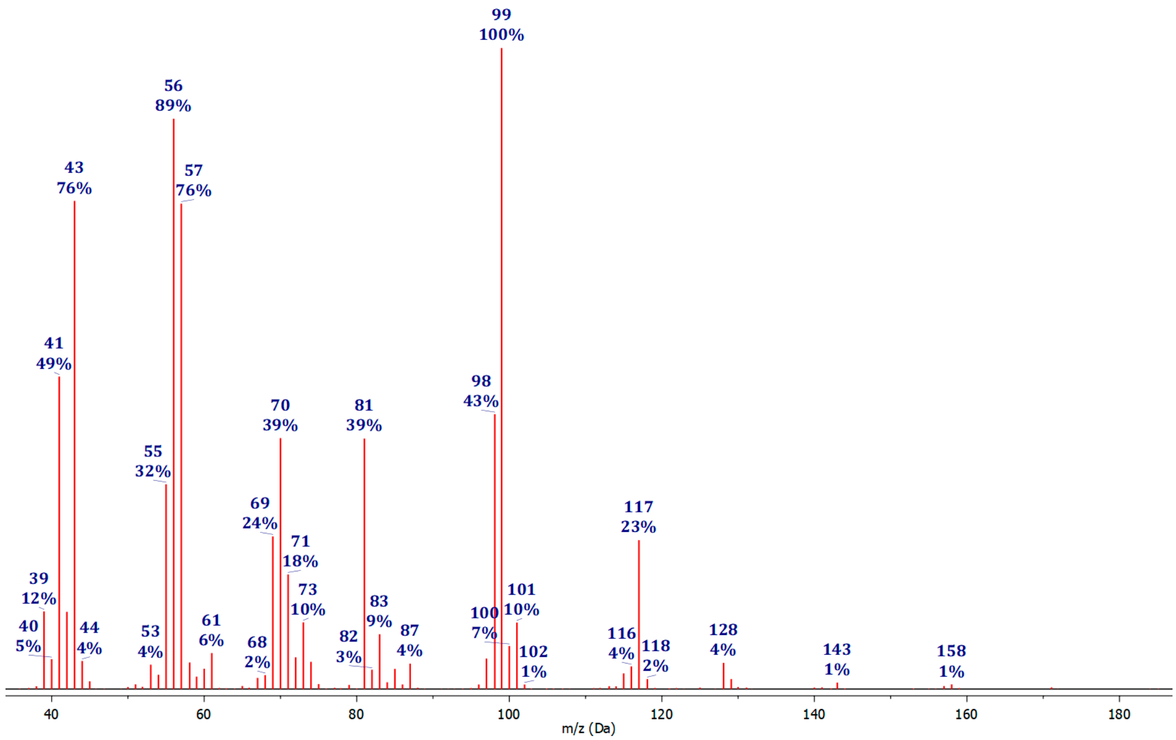

Figure S138. Mass spectrum of 2-methylhexyl 4-methylpentanoate (**12g**)

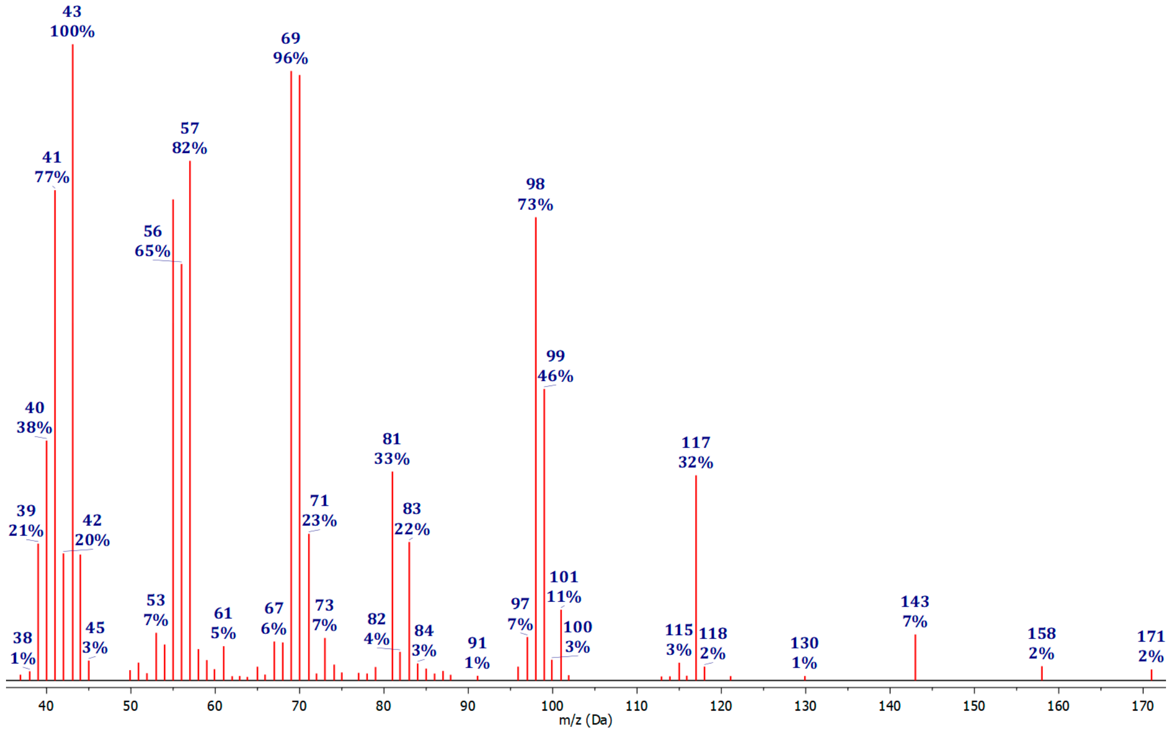

Figure S139. Mass spectrum of 3-methylhexyl 4-methylpentanoate (**13g**)

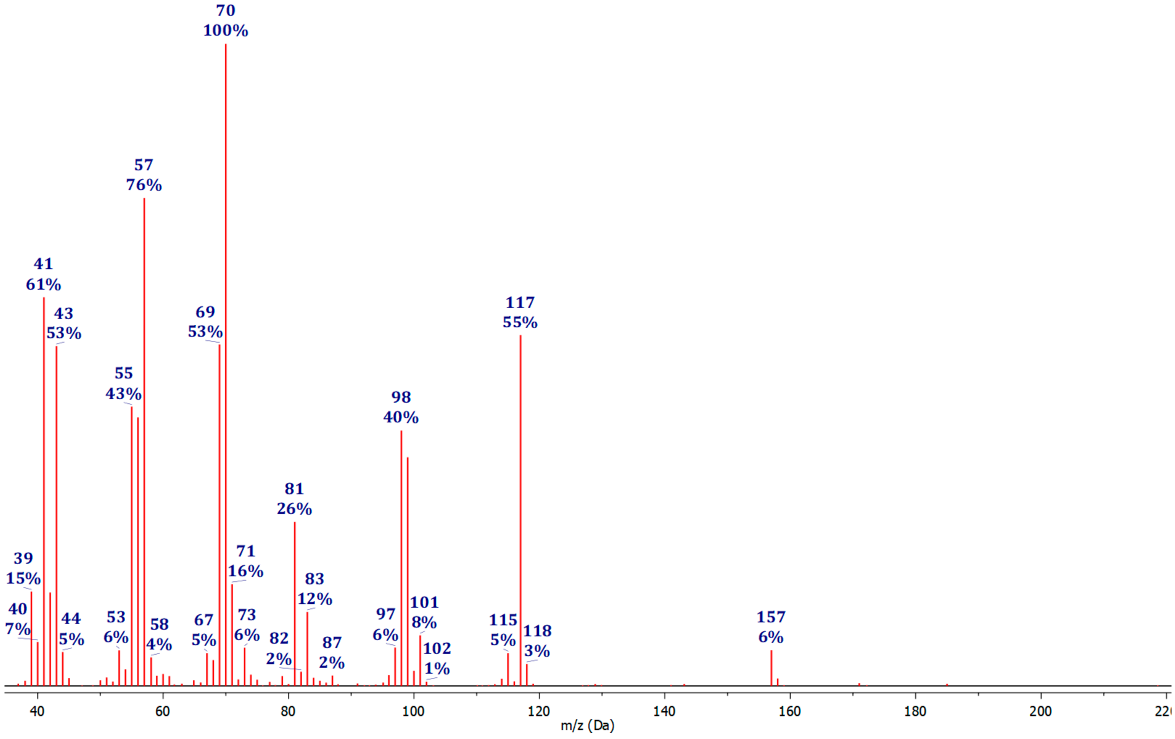

Figure S140. Mass spectrum of 4-methylhexyl 4-methylpentanoate (**14g**)

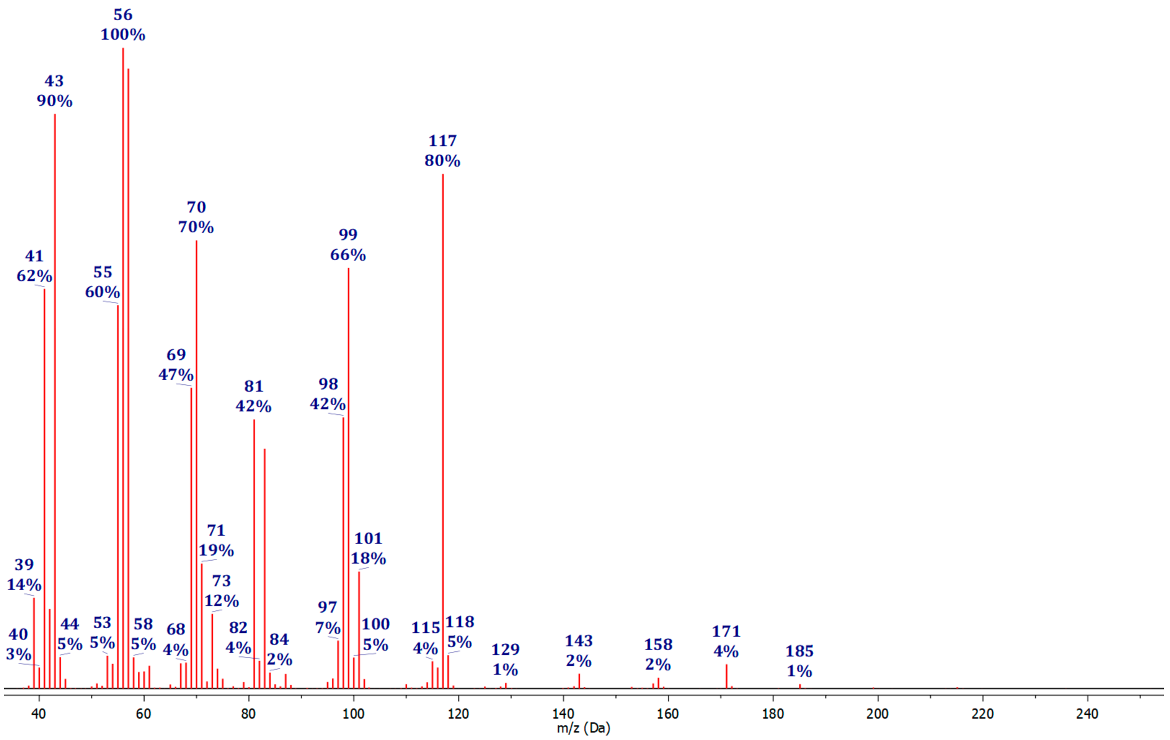

Figure S141. Mass spectrum of 5-methylhexyl 4-methylpentanoate (**15g**)

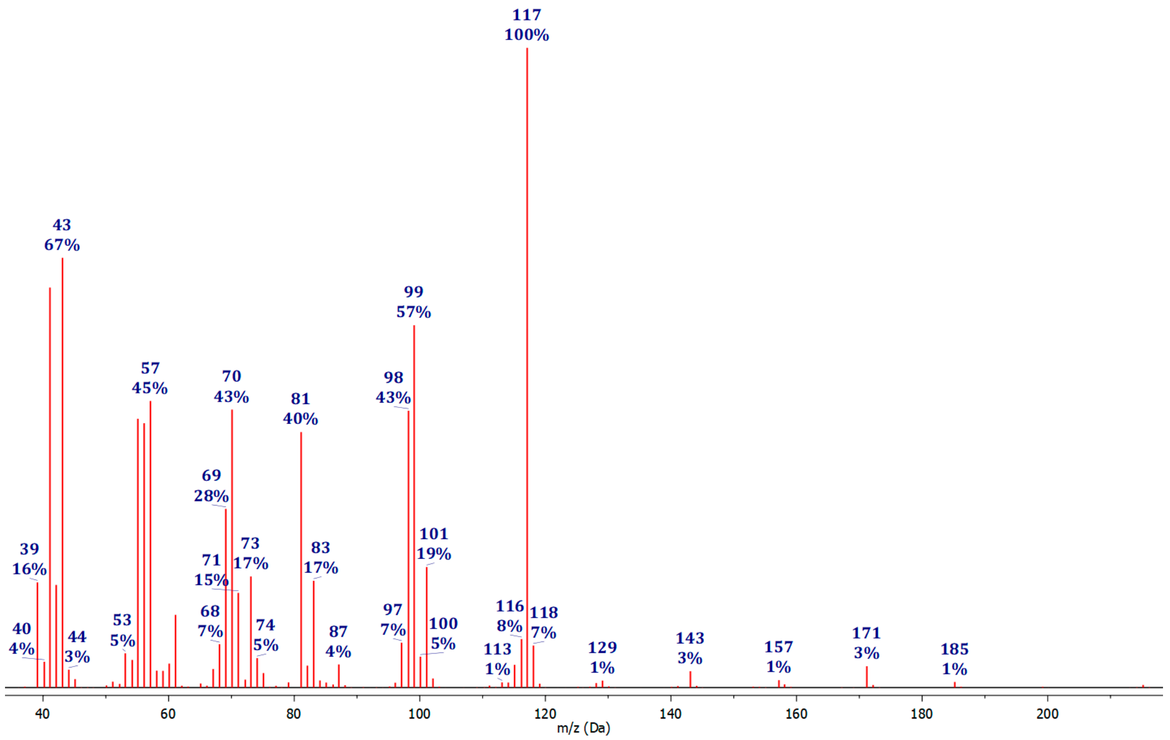

Figure S142. Mass spectrum of heptyl 4-methylpentanoate (**16g**)

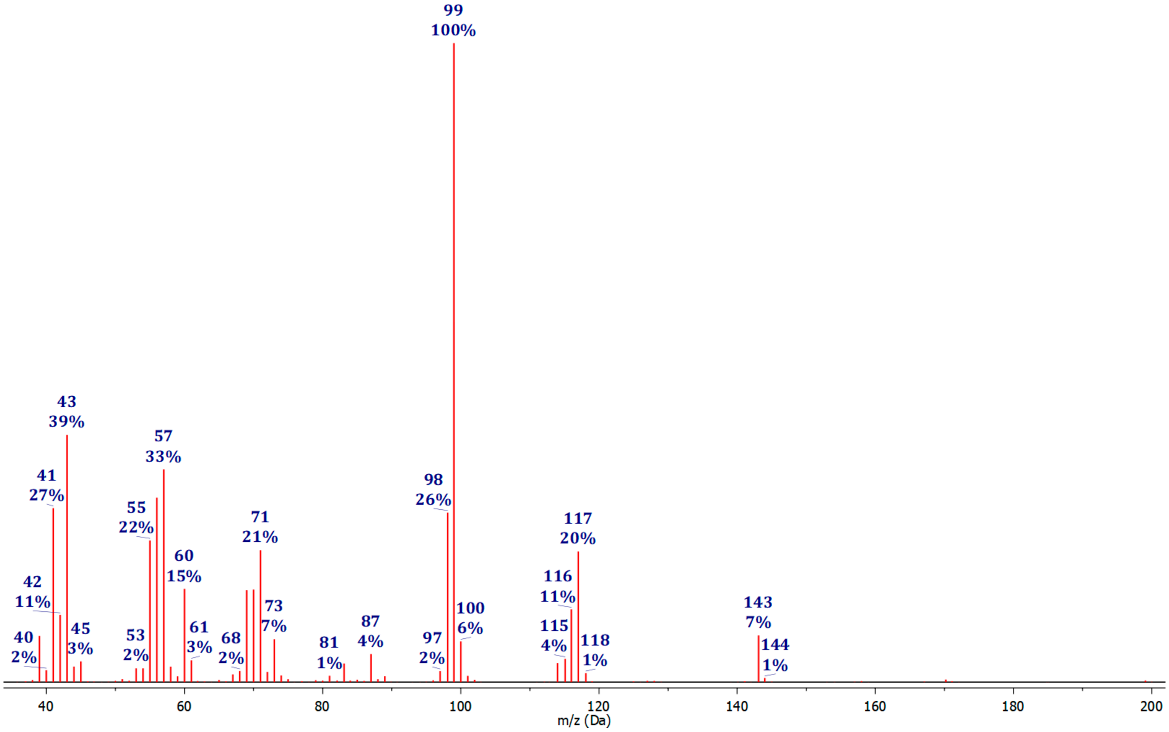

Figure S143. Mass spectrum of 1-methylhexyl hexanoate (**11h**)

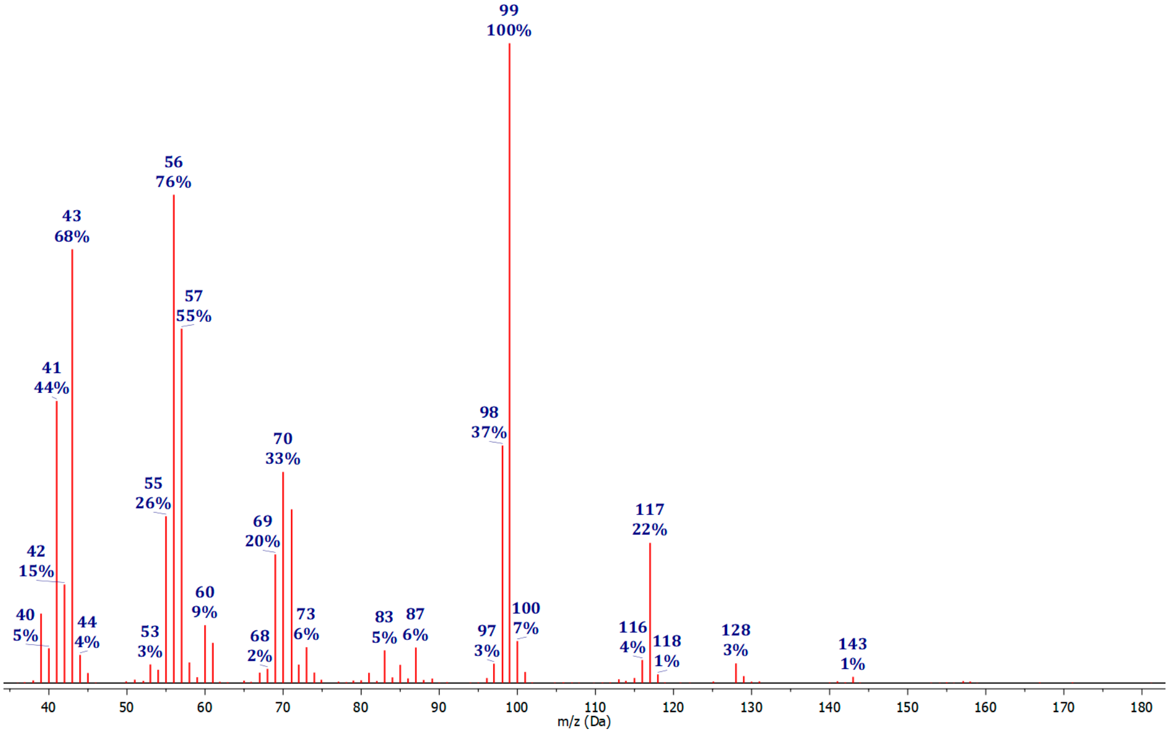

Figure S144. Mass spectrum of 2-methylhexyl hexanoate (**12h**)

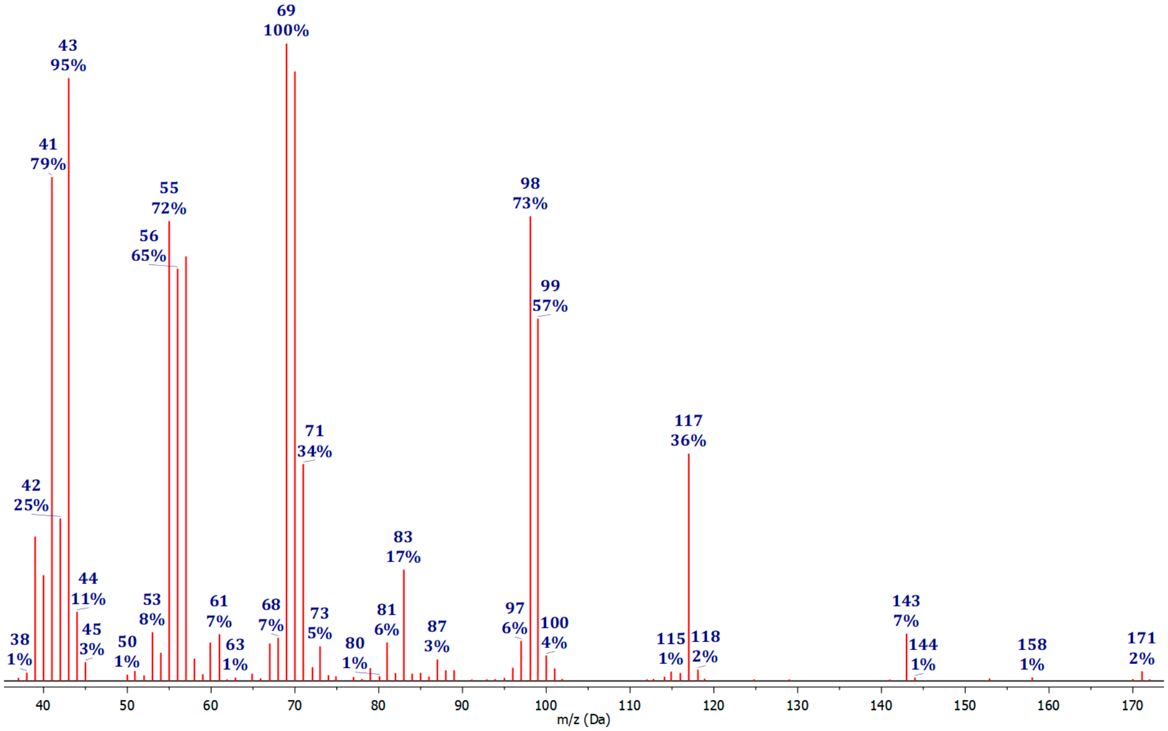

Figure S145. Mass spectrum of 3-methylhexyl hexanoate (**13h**)

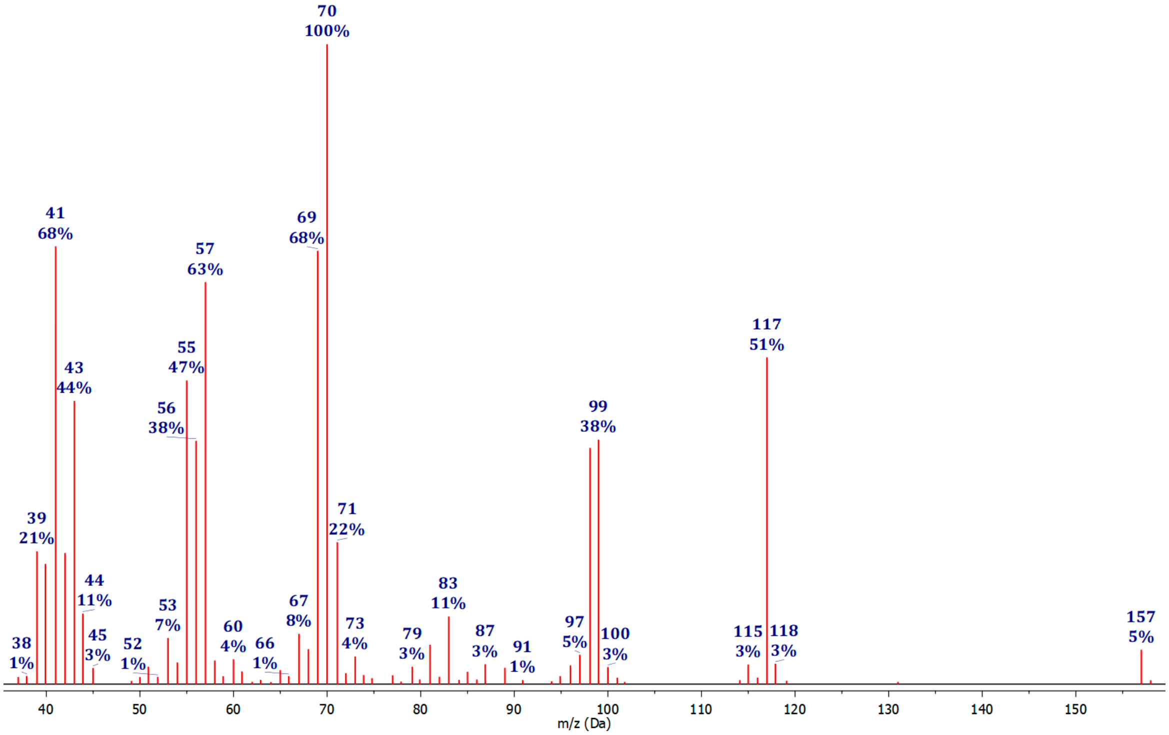

Figure S146. Mass spectrum of 4-methylhexyl hexanoate (**14h**)

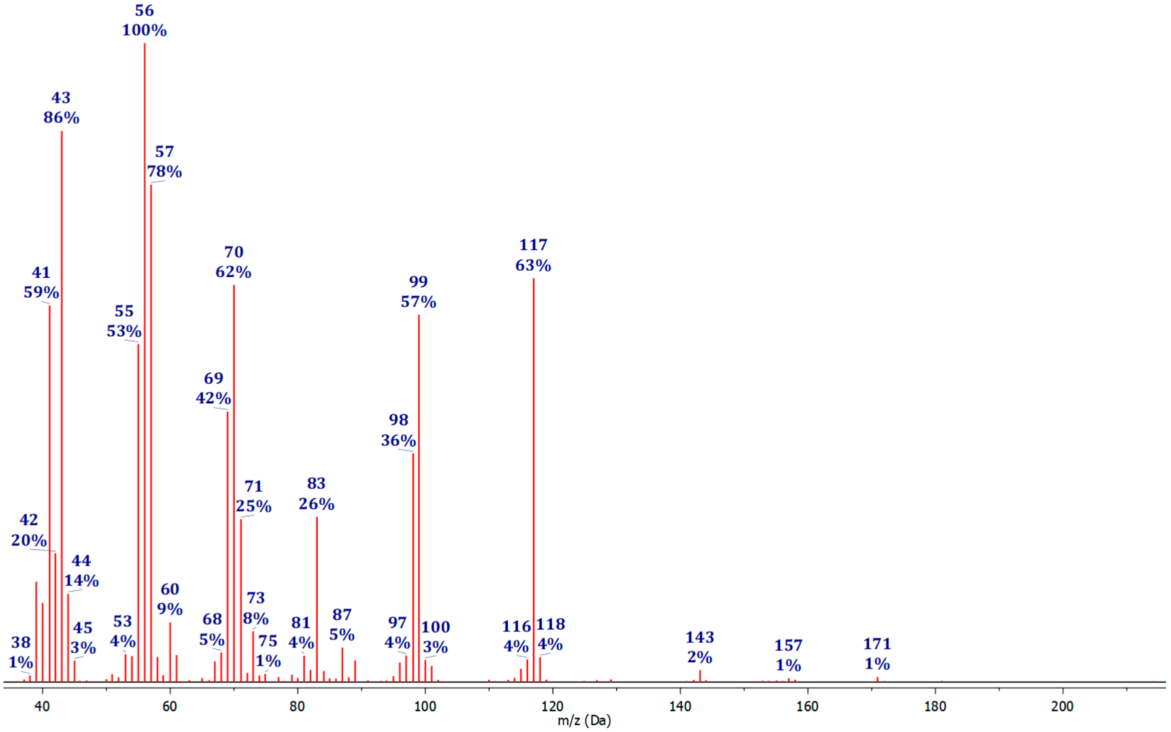

Figure S147a. Mass spectrum of 5-methylhexyl hexanoate (**15h**)

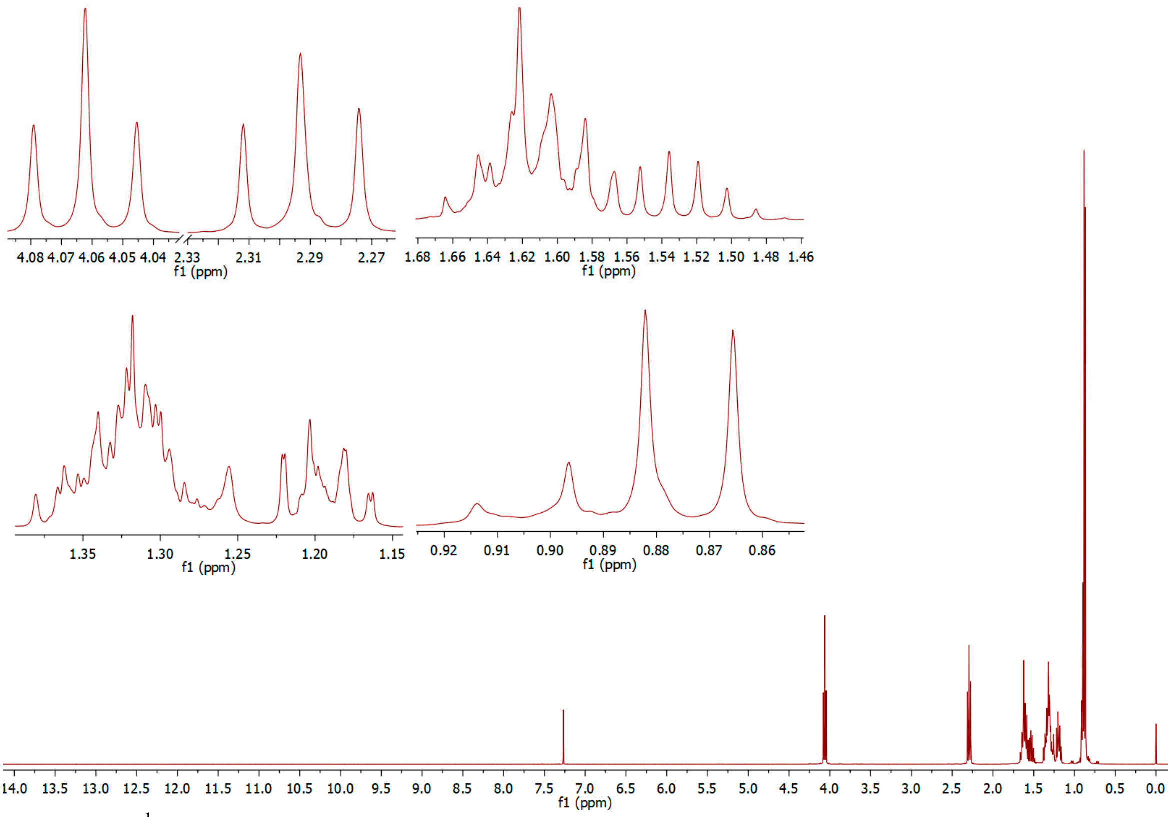

Figure S147b. ^{1}H NMR spectrum of 5-methylhexyl hexanoate (**15h**) recorded in $CDCl_3$

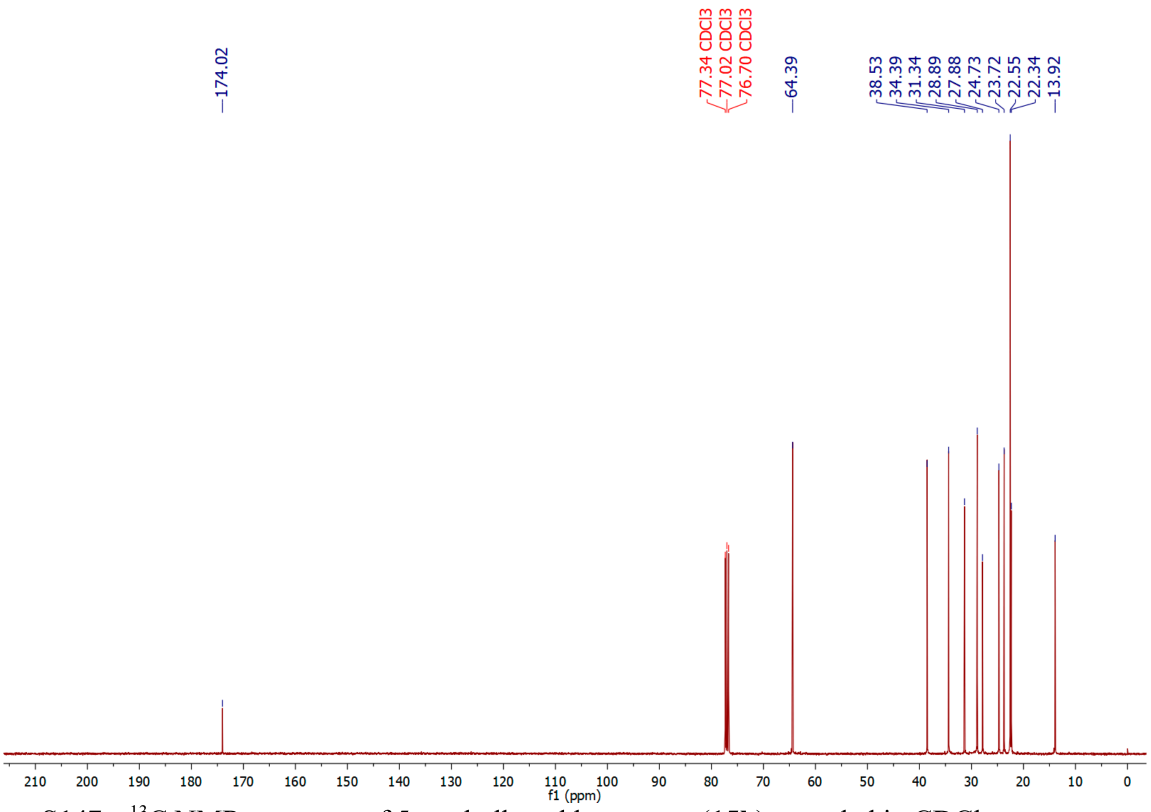

Figure S147c. ^{13}C NMR spectrum of 5-methylhexyl hexanoate (**15h**) recorded in $CDCl_3$

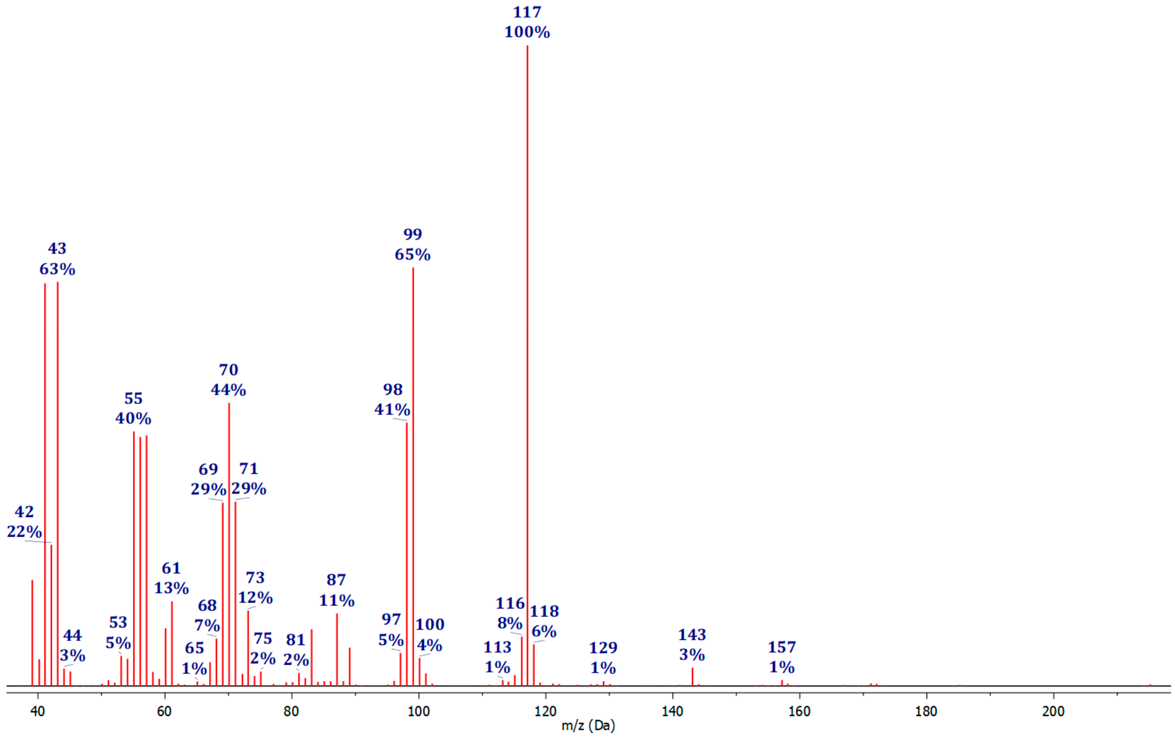

Figure S148. Mass spectrum of heptyl hexanoate (**16h**)

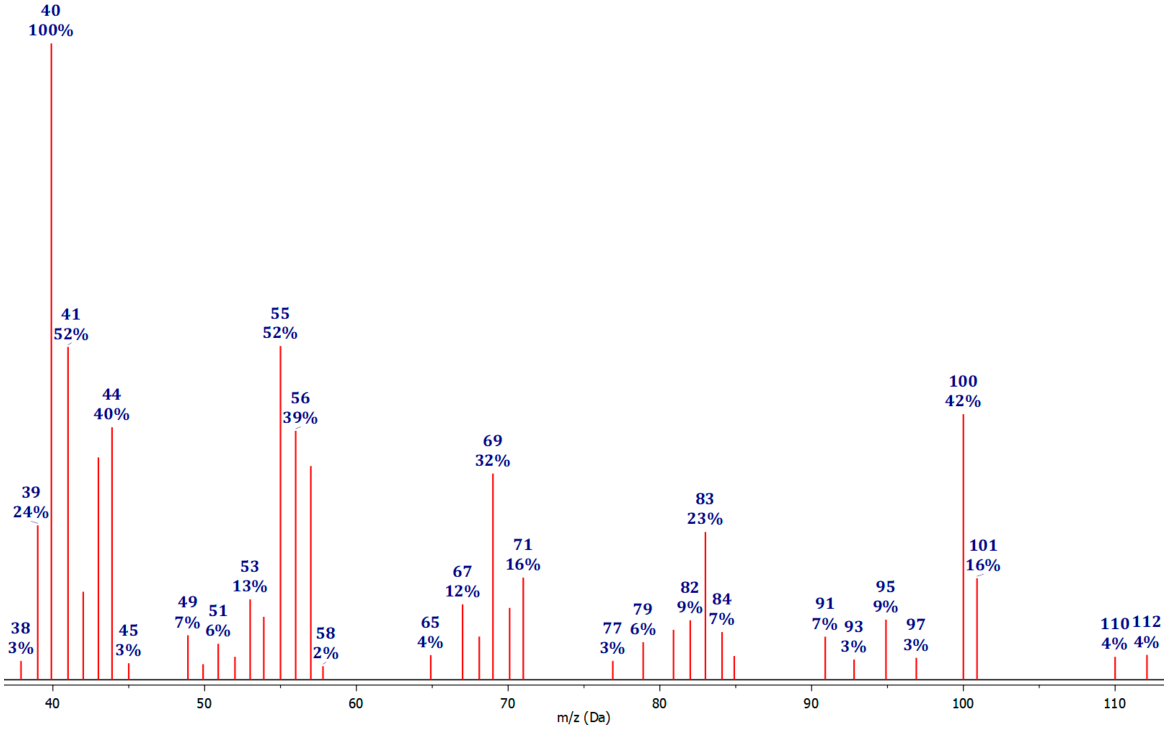

Figure S149. Mass spectrum of 6-methylheptyl angelate (**17o**)

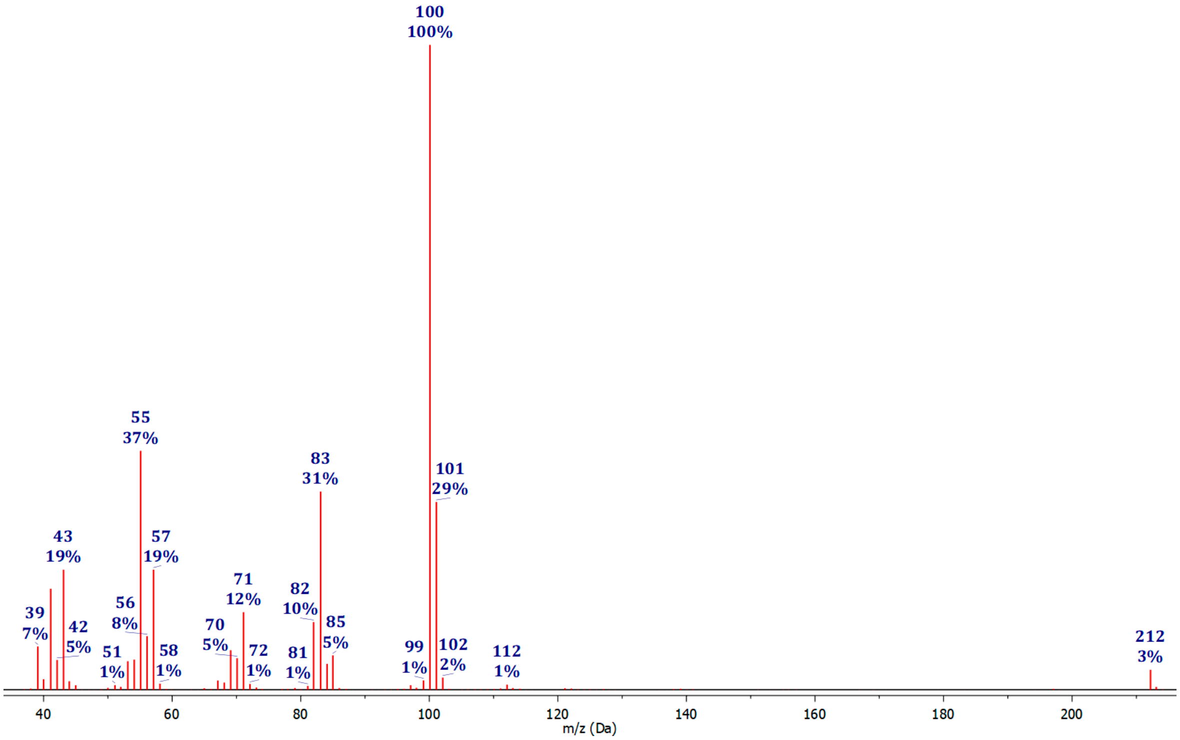

Figure S150. Mass spectrum of octyl angelate (**18o**)

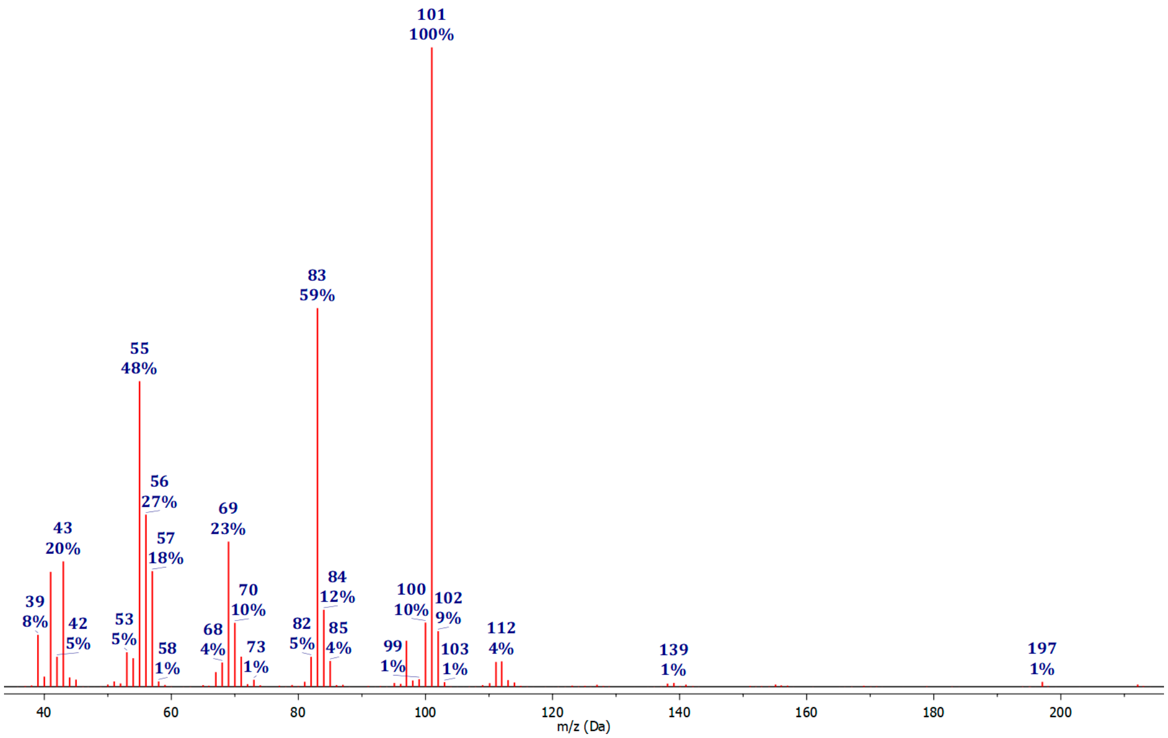

Figure S151. Mass spectrum of 6-methylheptyl tiglate (**17p**)

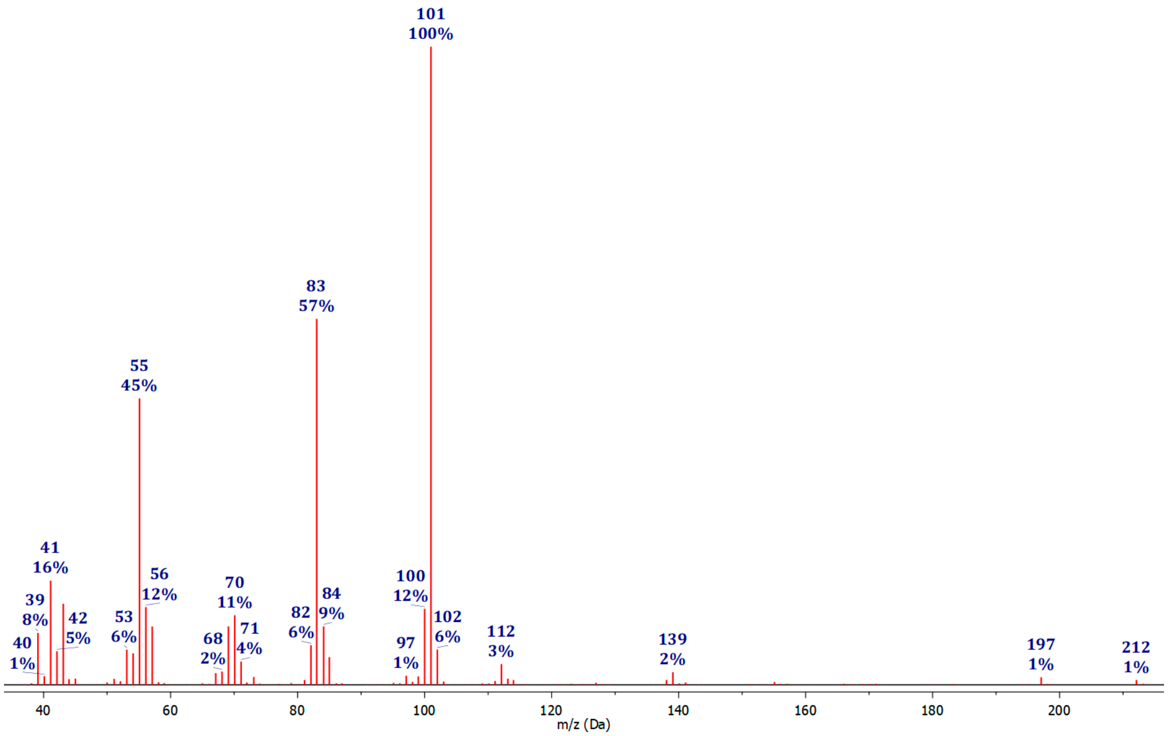

Figure S152. Mass spectrum of octyl tiglate (**18p**)

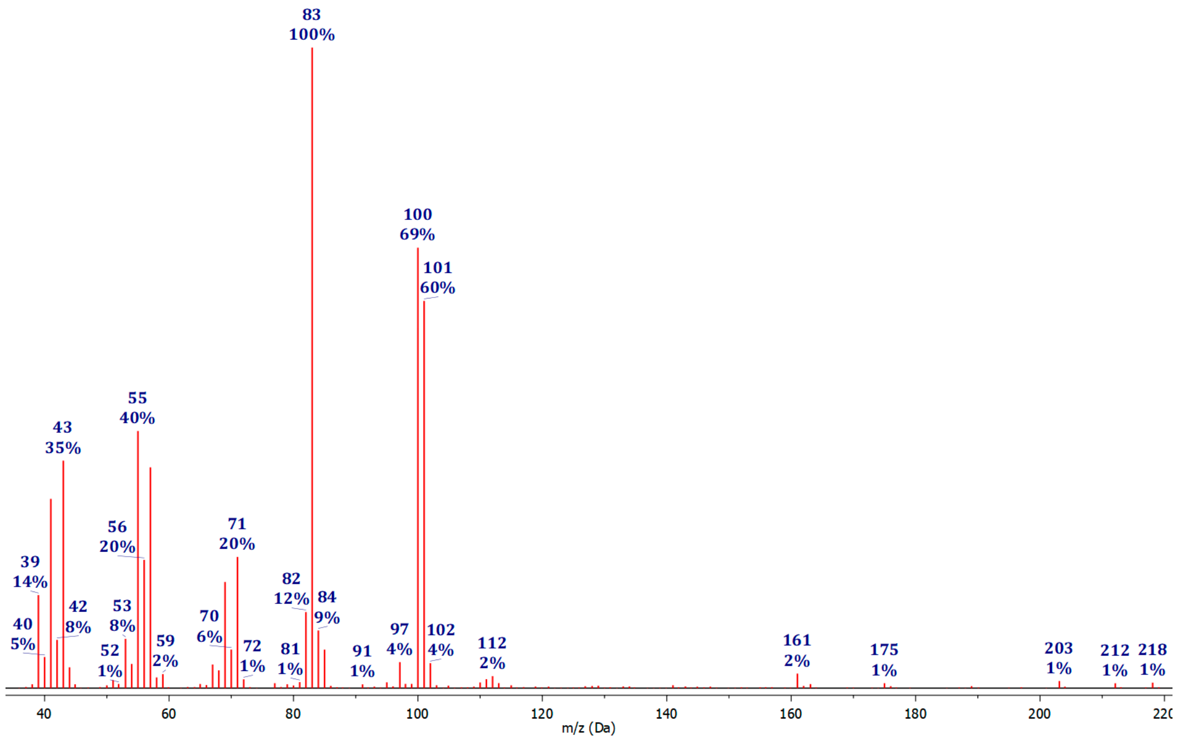

Figure S153. Mass spectrum of 6-methylheptyl senecioate (**17q**)

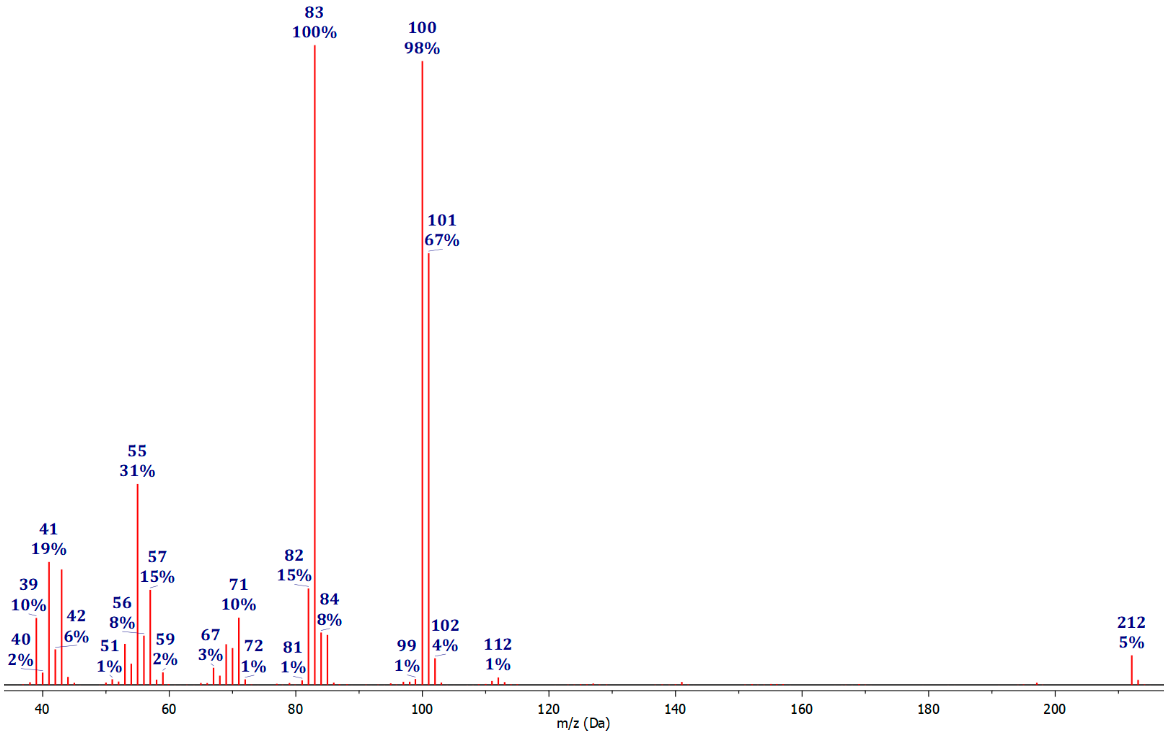

Figure S154. Mass spectrum of octyl senecioate (**18q**)

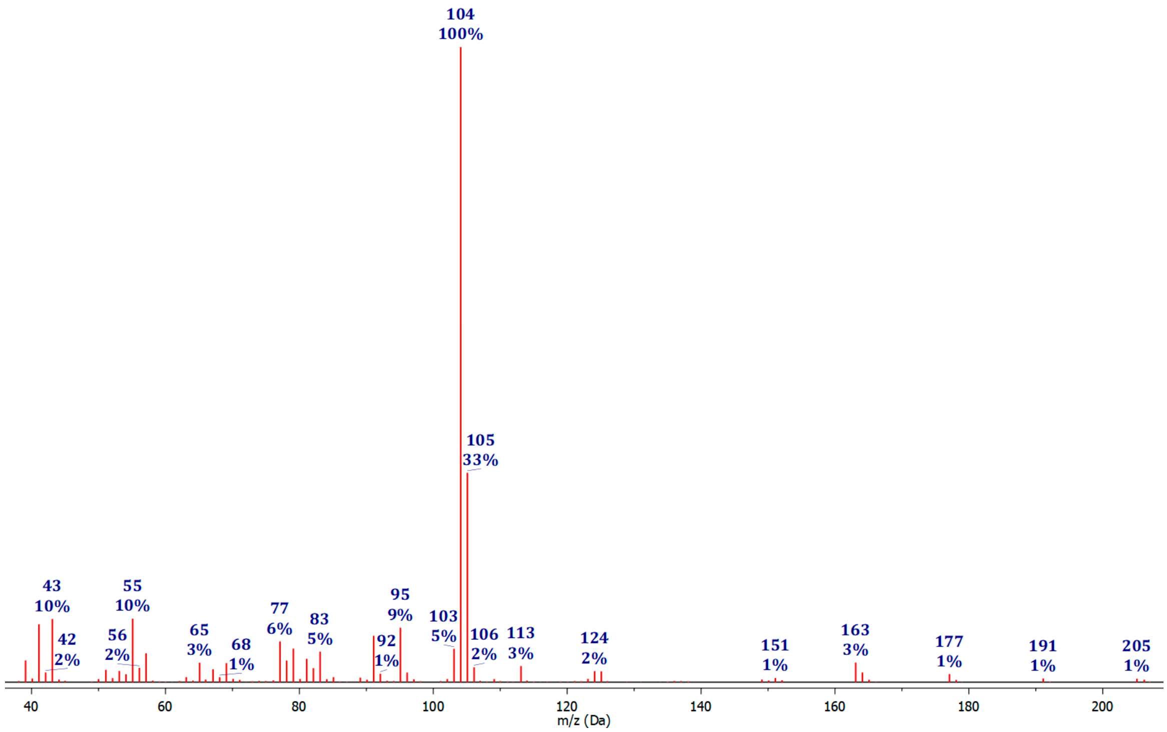

Figure S155a. Mass spectrum of 2-phenylethyl 4-methylhexanoate (**19i**)

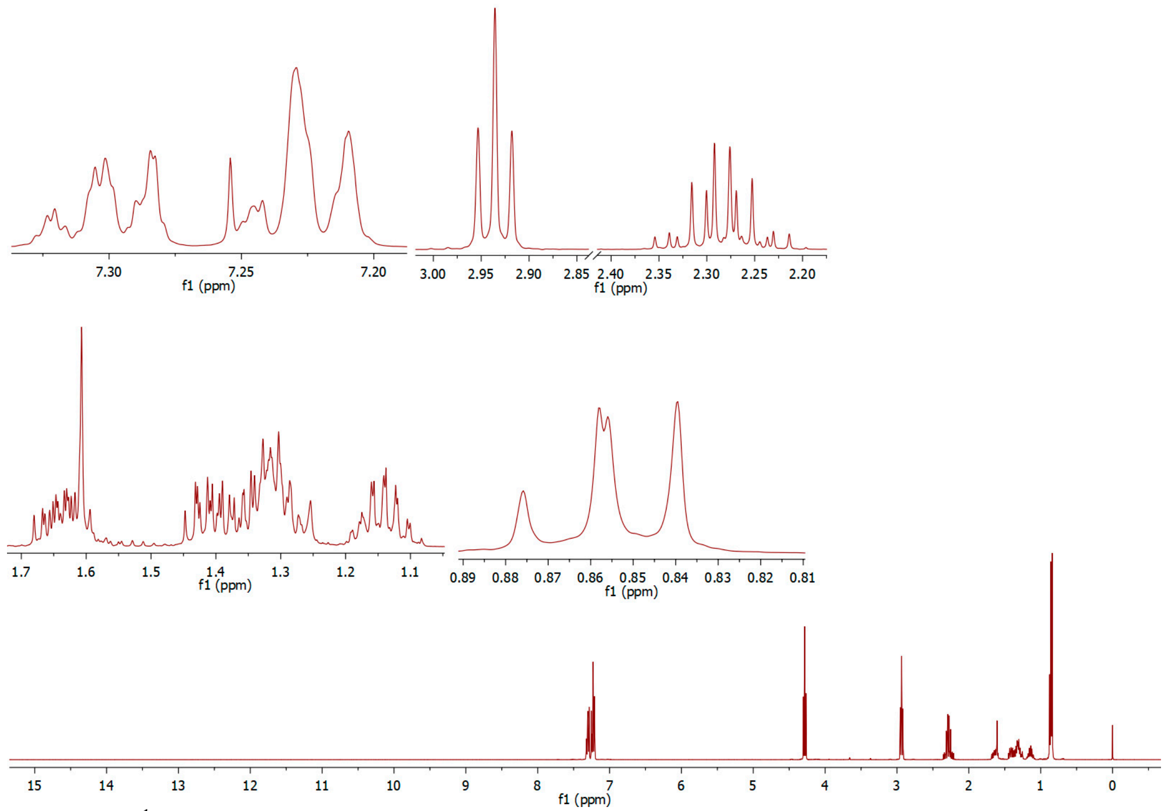

Figure S155b. ^{1}H NMR spectrum of 2-phenylethyl 4-methylhexanoate (**19i**) recorded in $CDCl_3$

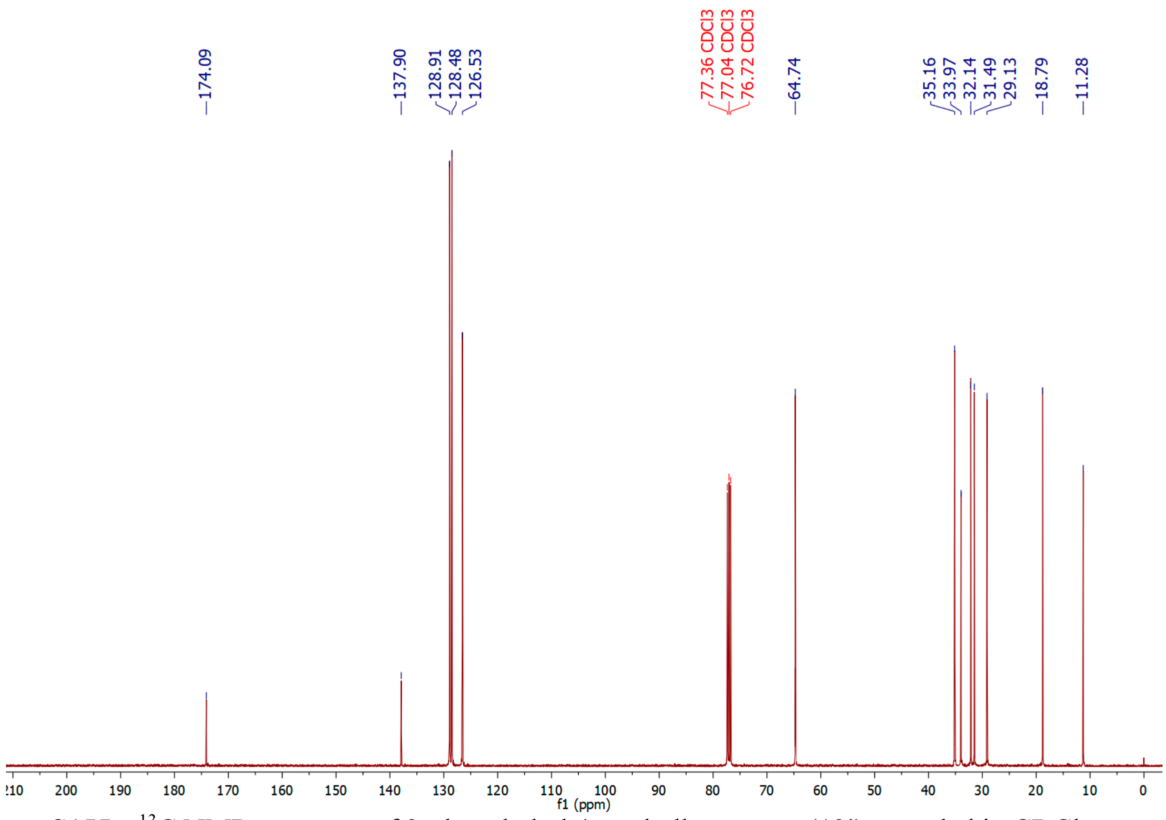

Figure S155c. ^{13}C NMR spectrum of 2-phenylethyl 4-methylhexanoate (**19i**) recorded in $CDCl_3$

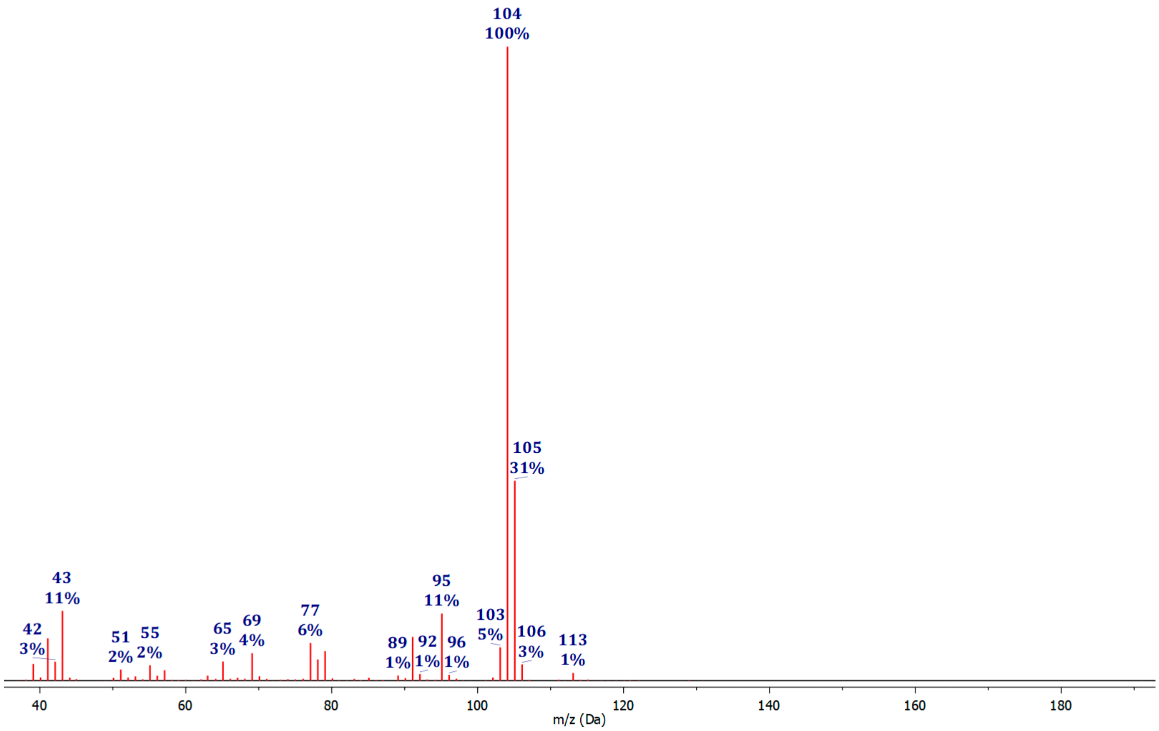

Figure S156a. Mass spectrum of 2-phenylethyl 5-methylhexanoate (**19j**)

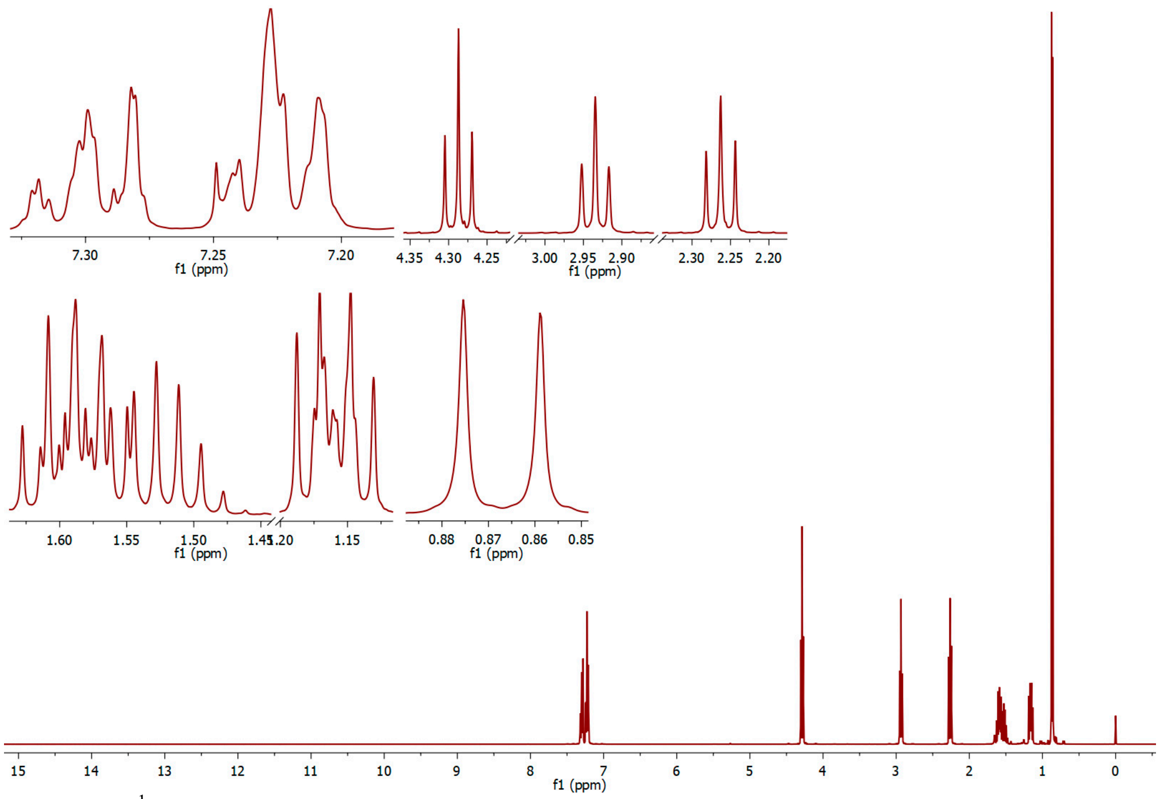

Figure S156b. ^{1}H NMR spectrum of 2-phenylethyl 5-methylhexanoate (**19j**) recorded in $CDCl_3$

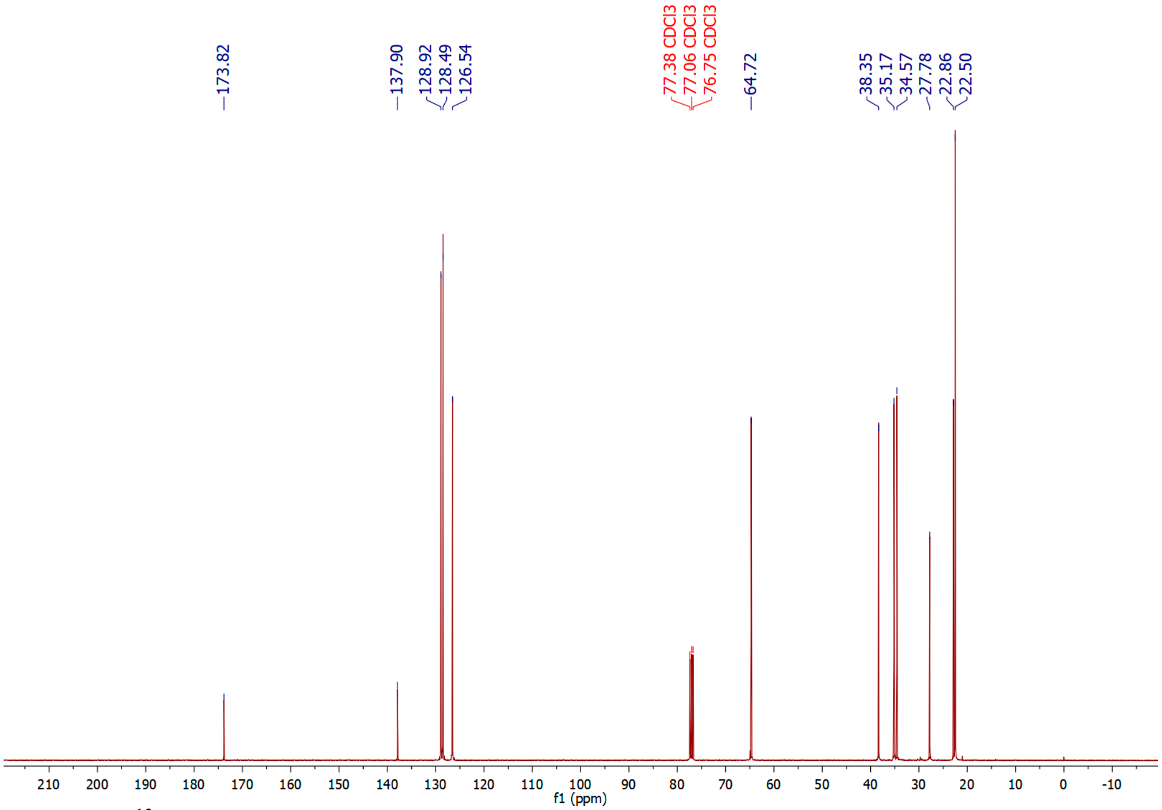

Figure S156c. ^{13}C NMR spectrum of 2-phenylethyl 5-methylhexanoate (**19j**) recorded in $CDCl_3$

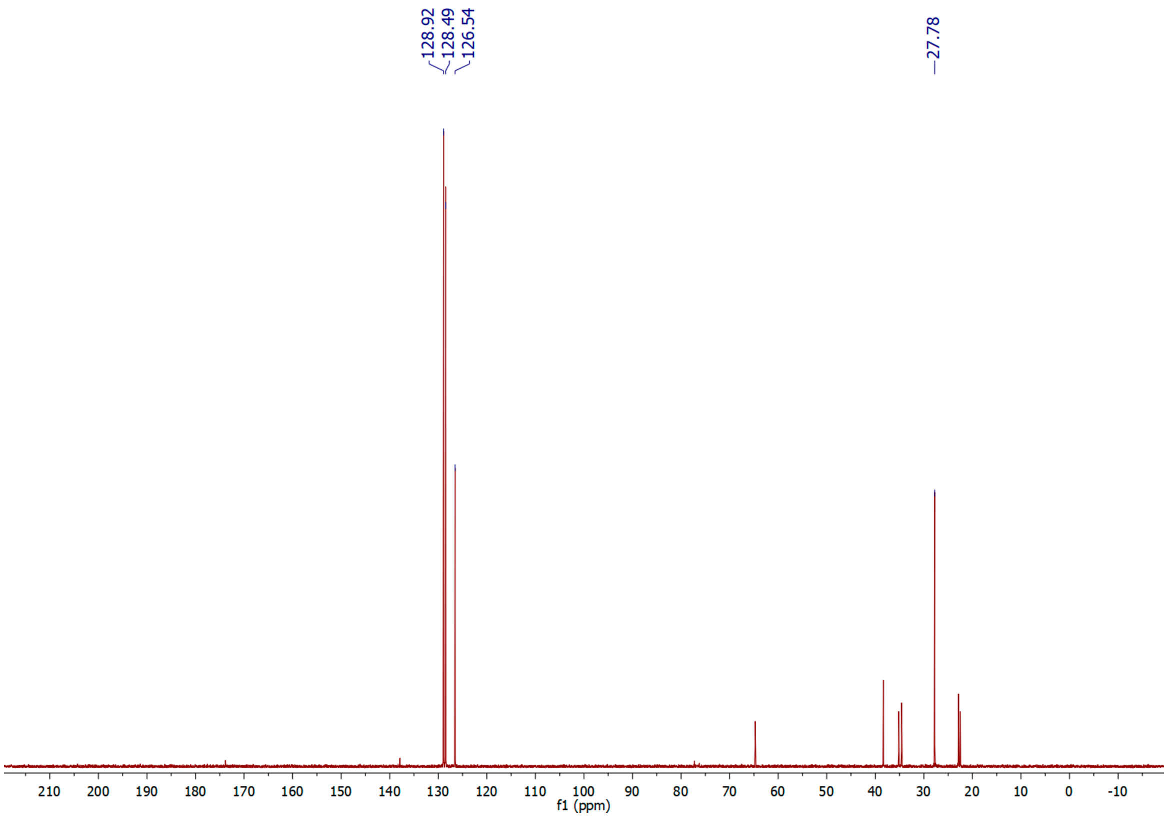

Figure S156d. DEPT 90 spectrum of 2-phenylethyl 5-methylhexanoate (**19j**) recorded in $CDCl_3$

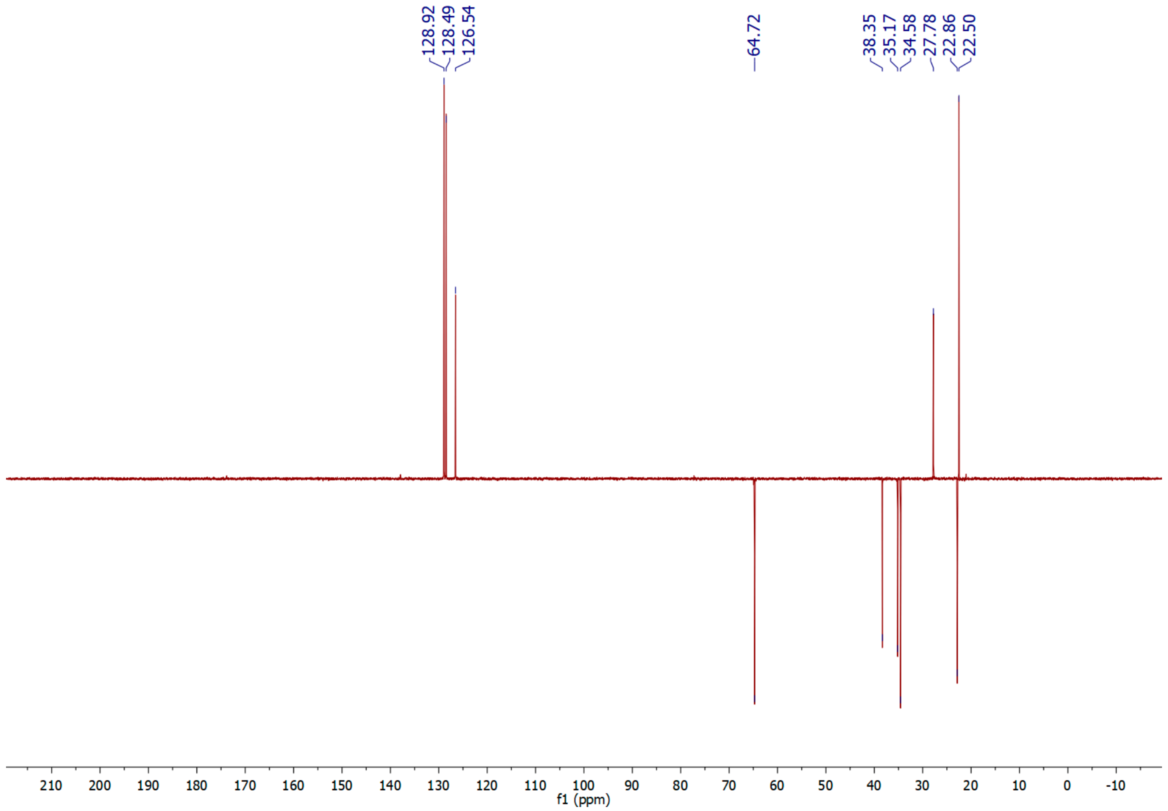

Figure S156e. DEPT 135 spectrum of 2-phenylethyl 5-methylhexanoate (**19j**) recorded in $CDCl_3$

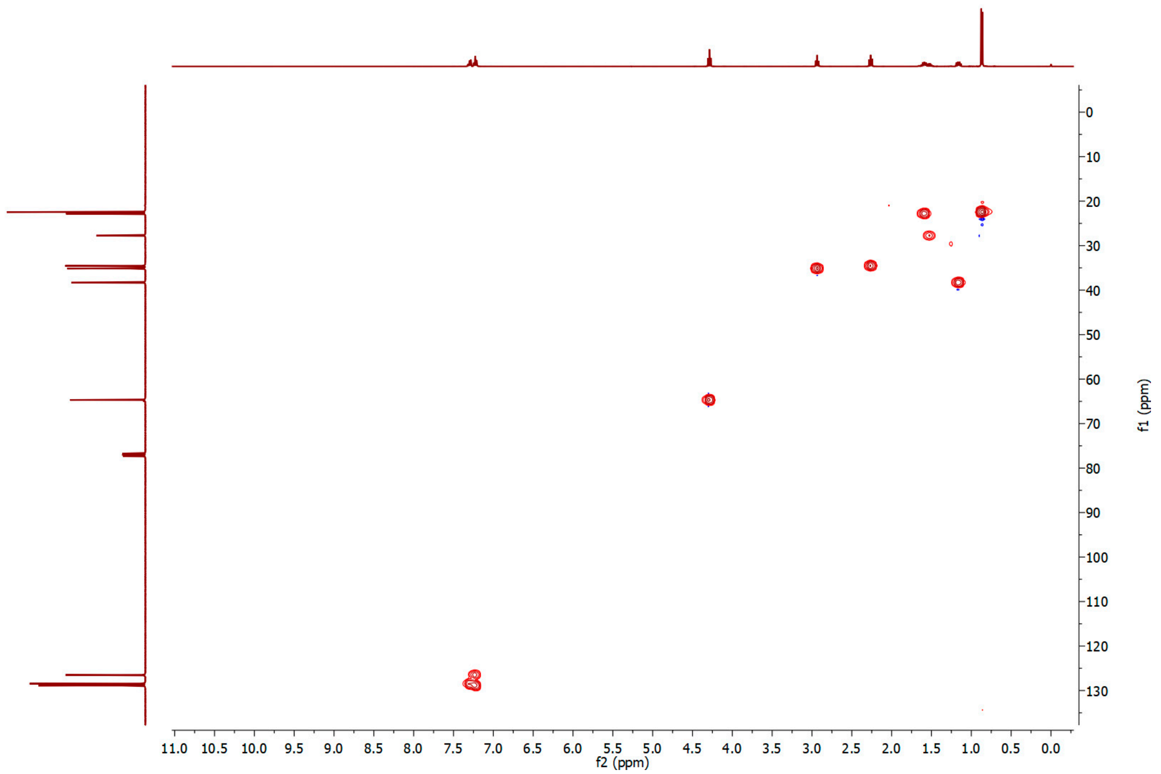

Figure S156f. HSQC spectrum of 2-phenylethyl 5-methylhexanoate (**19j**) recorded in $CDCl_3$

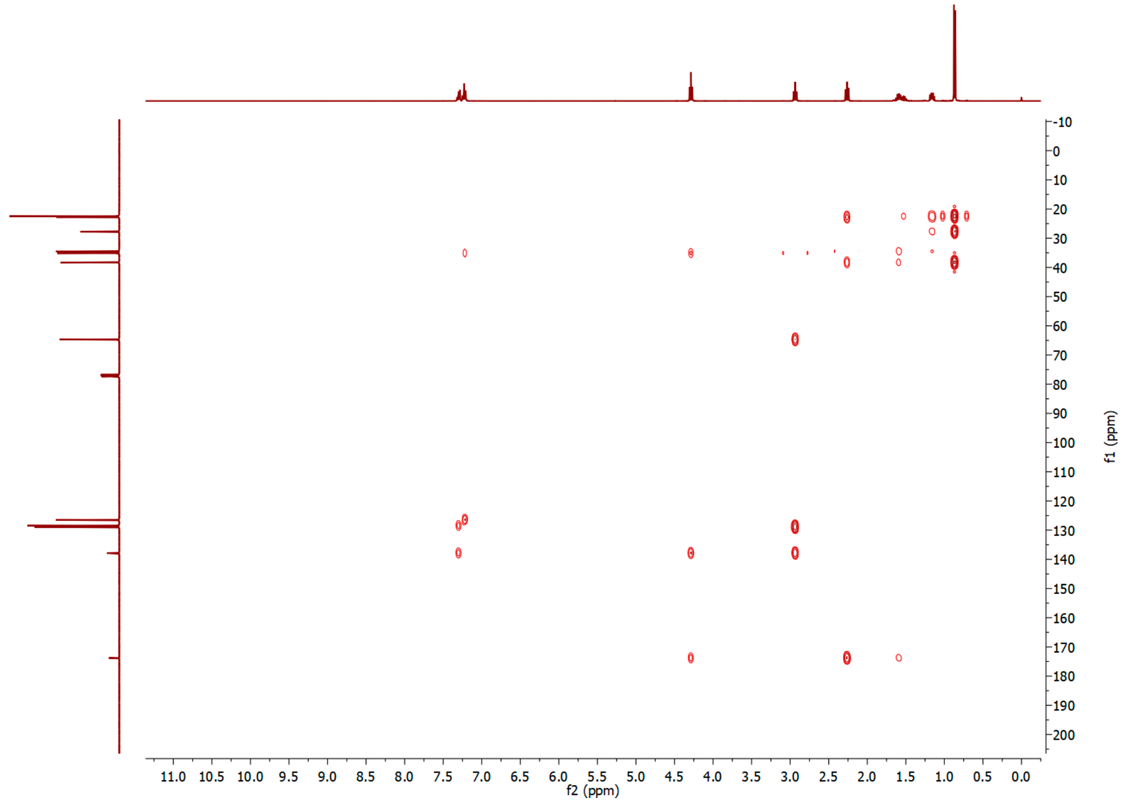

Figure S156g. HMBC spectrum of 2-phenylethyl 5-methylhexanoate (**19j**) recorded in $CDCl_3$

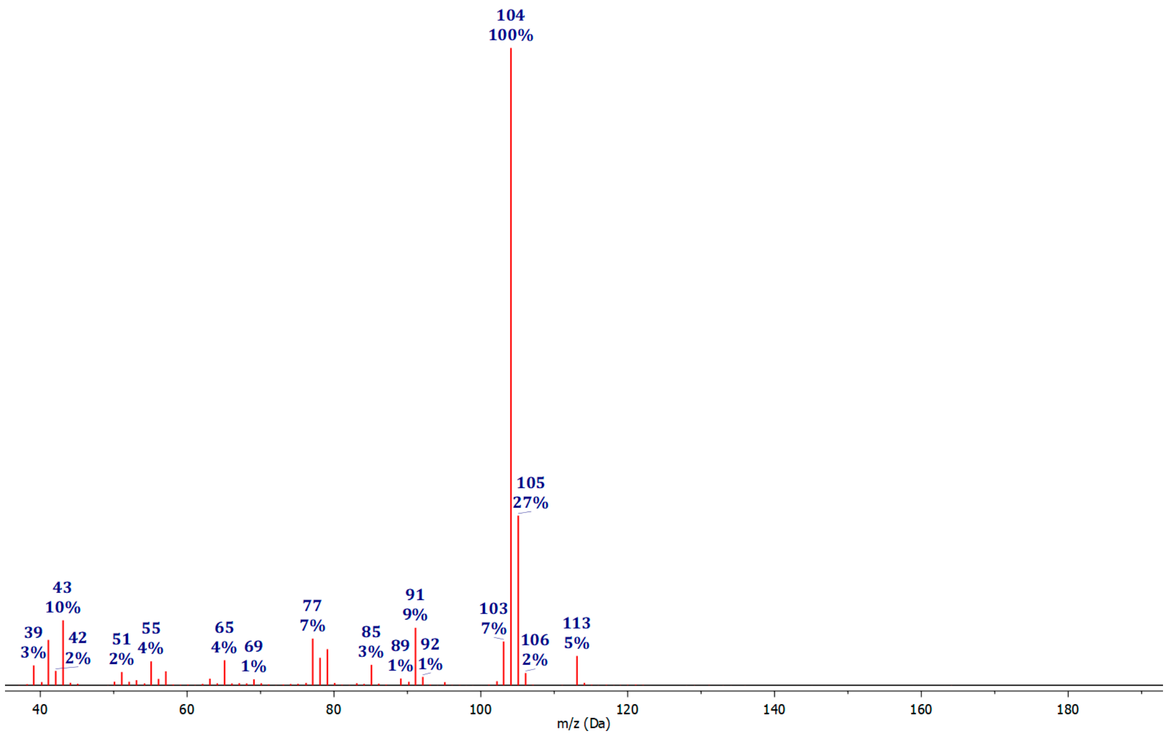

Figure S157. Mass spectrum of 2-phenylethyl heptanoate (**19k**)

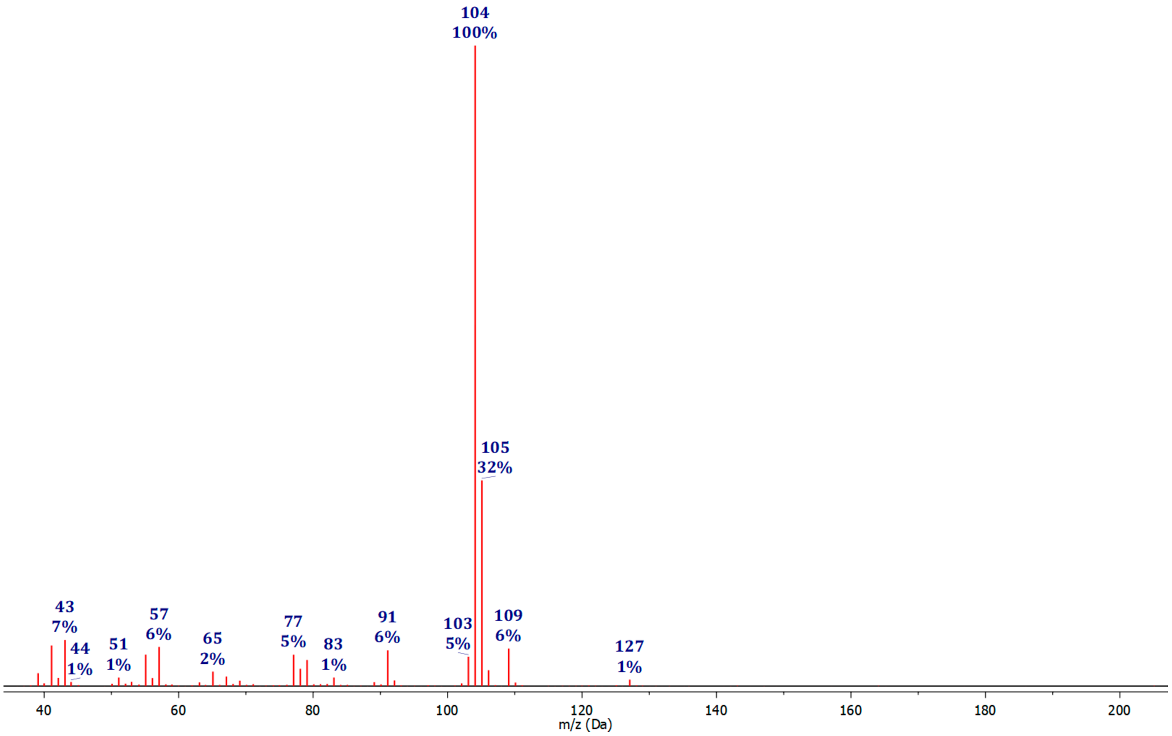

Figure S158. Mass spectrum of 2-phenylethyl 6-methylheptanoate (**19m**)

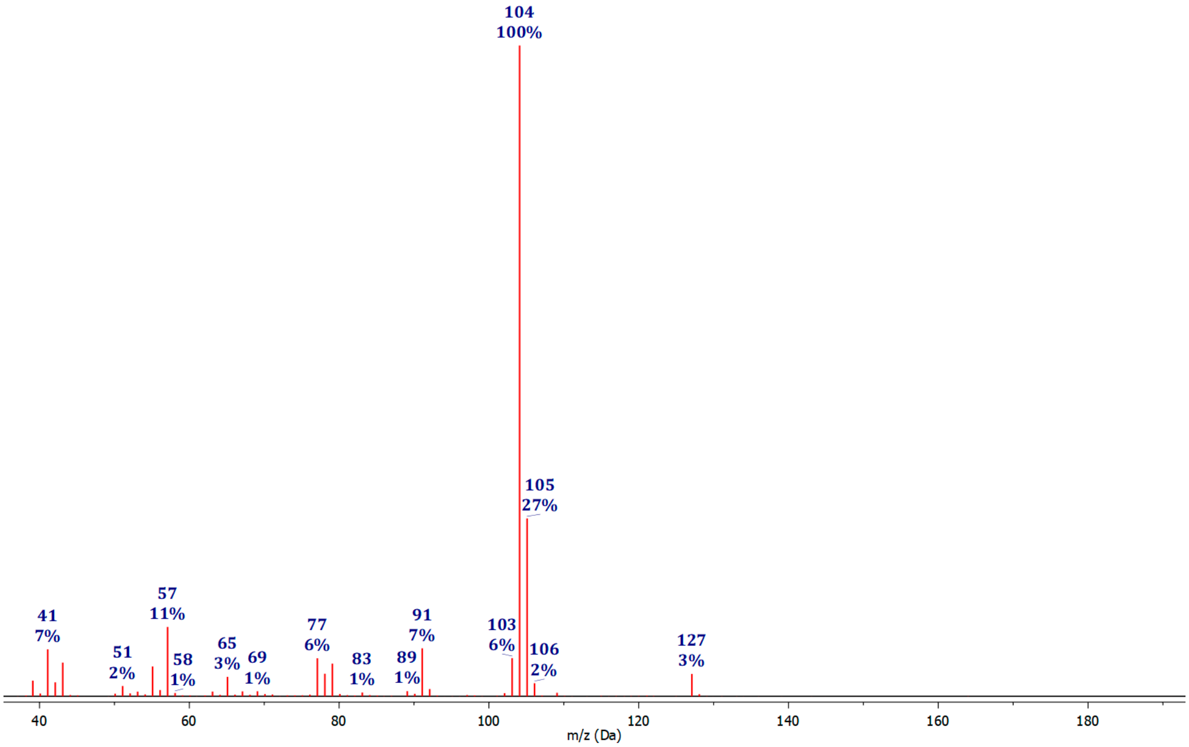

Figure S159. Mass spectrum of 2-phenylethyl octanoate (**19n**)

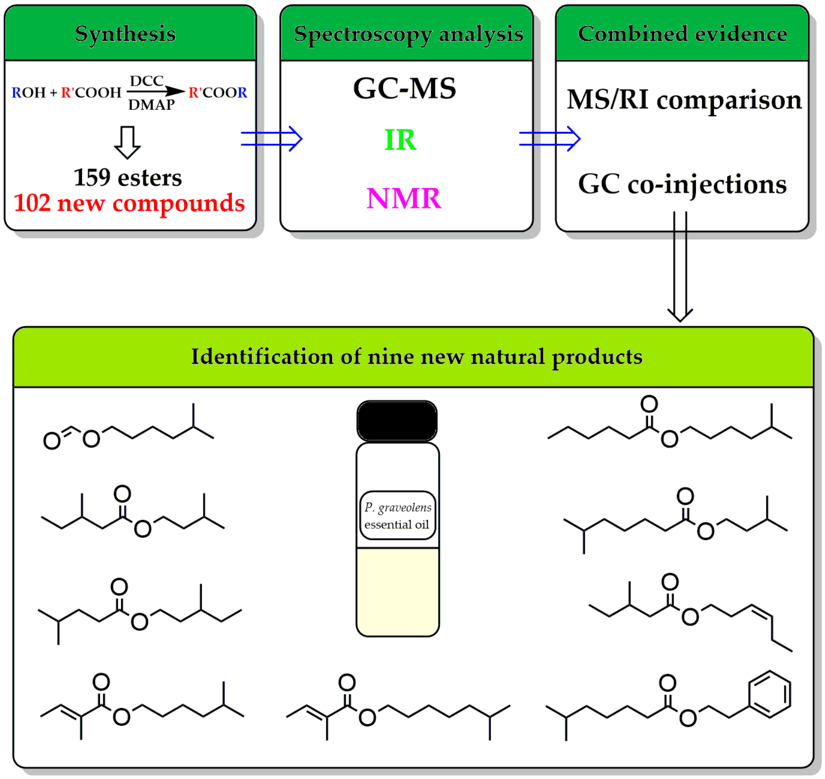

Figure S160. Schematic overview of the identification strategy: synthesis to final confirmation
